# Integrated DFT, molecular docking, and molecular dynamics investigation of some novel 2-thiohydantoin analogues as potent CDK2 inhibitors for anticancer therapy

**DOI:** 10.1038/s41598-026-42330-4

**Published:** 2026-03-26

**Authors:** Nada A. Khaled, Sayed A. Ahmed, Medhat A. Ibrahim, Osama M. Ahmed, Neama A. Mohamed, Nagy M. Khalifa

**Affiliations:** 1 Therapeutic Chemistry Department, Pharmaceutical and Drug Industries Research Institute, National Research Centre, 33 El-Bohouth St., Dokki, Giza, 12622 Egypt; 2 Chemistry Department, Faculty of Science, Beni-Suef University, Beni-Suef, 62521 Egypt; 3 Basic Science Department, Faculty of Engineering, Nahda University Beni-Suef (NUB), Beni Suef, Egypt; 4 Spectroscopy Department, National Research Centre, 33 El-Bohouth St., Dokki, Giza, 12622 Egypt; 5 Molecular Modeling and Spectroscopy Laboratory, Centre of Excellence for Advanced Science, National Research Centre, 33 El-Bohouth St., Dokki, Giza, 12622 Egypt; 6 Center for Converging Sciences and Emerging Technology (CoSET), Benha National University (BNU), Al Obour City, 13518 Egypt; 7 Zoology Department, Faculty of Science, Beni-Suef University, Beni-Suef, 62521 Egypt

**Keywords:** CDK2 inhibitors, 2-Thiohydantoin derivatives, DFT calculations, Molecular docking, Molecular dynamics, MM-PBSA, Anticancer agents, Biochemistry, Cancer, Chemical biology, Chemistry, Computational biology and bioinformatics, Drug discovery

## Abstract

Cancer progression is driven by dysregulation of cyclin-dependent kinase 2 (CDK2), a critical cell cycle regulator. This study employed an integrated computational approach combining Density Functional Theory (DFT), molecular docking, molecular dynamics (MD) simulations, and MM-PBSA calculations to evaluate 2-thiohydantoin derivatives as CDK2 inhibitors. DFT calculations revealed compounds **2b-e** narrowest lowest unoccupied molecular orbital (LUMO)- highest occupied molecular orbital (HOMO) gaps (3.02–3.26 eV in DMSO) and highest electrophilicity indices (> 3.20 eV), indicating enhanced reactivity toward biological targets. QTAIM and Fukui function analyses identified key electrophilic centers (C2, O12, C14) and hydrogen bonding sites essential for protein interactions. Molecular docking against CDK2 (PDB: 1HCK) showed compounds **2c**, **2d**, and **2b** exhibited superior binding affinities (-9.312, -9.303, and − 9.269 kcal/mol) compared to ATP (-8.460 kcal/mol), forming critical hydrogen bonds with Lys33 and Thr14. The 10 ns MD simulations confirmed stable binding, with compound **2f** maintaining highest conformational stability (RMSD ~ 0.05 nm) and robust hydrogen bonding (mean: 2.70 bonds). MM-PBSA analysis revealed compound **2d** achieved optimal binding affinity (ΔG_bind = -34.50 ± 0.42 kcal/mol) through balanced van der Waals interactions (-50.74 kcal/mol) and minimal desolvation penalty (52.40 kcal/mol). Compounds **2b**,** 2c**,** 2d**, and **2f** emerged as lead candidates for experimental validation as next-generation CDK2-targeted anticancer agents.

**Supplementary Information:**

The online version contains supplementary material available at 10.1038/s41598-026-42330-4.

## Introduction

Cancer remains a leading cause of mortality worldwide and is increasingly addressed through mechanism-based targeted therapies rather than non-selective cytotoxic agents. Protein kinases constitute one of the most intensively investigated drug target classes because of their central roles in regulating cell proliferation, survival signaling, and tumor progression. However, despite major advances in kinase-directed therapeutics, achieving isoform selectivity among closely related kinases continues to represent a significant challenge^1–3^. Recent clinical evaluations have emphasized that poor selectivity often leads to dose-limiting toxicities and reduced therapeutic indices^4^, necessitating the development of enhanced drug-target interaction (DTI) prediction models to improve safety profiles^5^. Furthermore, the evolution of delivery systems, such as metal-organic frameworks, highlights the ongoing effort to refine the precision of combinatorial cancer treatments^6^. Cyclin-dependent kinase 2 (CDK2) is a critical regulator of cell-cycle progression, functioning in complex with cyclins E and A to control the G1/S transition and S-phase progression^7–9^. Dysregulation and hyperactivation of CDK2 have been implicated in the pathogenesis of multiple malignancies, including breast cancer, ovarian cancer, and melanoma^10–14^. As the therapeutic landscape broadens to include monoclonal antibodies^15^ and multifaceted biological macromolecules like heparin derivatives^16^, understanding the specific stabilization and activation of CDK family members becomes vital^17^. Consequently, CDK2 has emerged as a validated therapeutic target, and numerous small-molecule inhibitors have entered clinical investigation. Early CDK2-directed agents, including dinaciclib, roscovitine, and milciclib, demonstrated promising antiproliferative activity; however, their clinical utility has been limited by poor selectivity across the CDK family^18–21^. The high sequence and structural homology between CDK2 and other essential CDKs, particularly CDK1, frequently results in off-target toxicities such as myelosuppression and gastrointestinal effects, leading to narrow therapeutic windows and discontinuation in early trials^22–24^. These limitations highlight the continued need for structurally novel scaffolds capable of forming distinct interaction patterns within the CDK2 ATP (Adenosine-5’-Triphosphate)-binding pocket to improve selectivity profiles. Privileged heterocyclic scaffolds provide versatile platforms for kinase inhibitor development due to their structural adaptability and capacity to establish key hydrogen-bonding and π-stacking interactions within catalytic domains. Among these, the 2-thiohydantoin nucleus represents an attractive pharmacophore characterized by dual carbonyl/thiocarbonyl groups and adjacent heteroatoms that enable tunable electronic distribution and diverse substitution patterns. Thiohydantoin derivatives exhibit a wide range of biological activities, including anticonvulsant, antimicrobial, and anticancer properties^25–27^, and have been reported as modulators of enzyme systems, including kinases^28,29^. Nevertheless, despite their favorable physicochemical features, systematic investigation of 2-thiohydantoin derivatives as CDK2 inhibitors remains scarce, and the electronic and structural determinants governing their interaction with CDK2 have not been comprehensively elucidated^30,31^. Modern computer-aided drug design employs multiscale computational techniques to rationalize ligand behavior from electronic structure to dynamic binding stability. Density Functional Theory (DFT) provides molecular-level insight into electronic reactivity and stability^32^, while molecular docking predicts binding modes within target active sites^33^. Molecular Dynamics (MD) simulations further characterize conformational flexibility under near-physiological conditions^34^, and MM-PBSA/GBSA approaches allow estimation of binding free energies^35^. In this study, we report a comprehensive computational investigation of newly designed 2-thiohydantoin derivatives as potential CDK2 inhibitors for targeted anticancer therapy. Unlike previous studies that primarily rely on docking-based screening or isolated computational approaches, the present work integrates density functional theory (DFT), molecular docking, and molecular dynamics (MD) simulations coupled with MM-PBSA free energy calculations within a unified framework. A key novel aspect of this research is the systematic elucidation of how the specific electronic environment and polarizability of the 2-thiohydantoin sulfur atom influence the stability of the ligand-hinge region interactions. This multilevel strategy enables the establishment of a direct link between electronic structure and dynamic binding stability within the CDK2 active site (PDB ID: 1HCK). The findings provide unique mechanistic insights into structure–activity relationships, offering a predictive blueprint for the rational prioritization of lead candidates for subsequent experimental validation.

## Computational methodology

### DFT study

The molecular structures of compounds **1**, **2a–i**, and **3** were initially sketched in ChemDraw and converted into three-dimensional geometries using Chem3D. Conformational analysis was performed through systematic potential energy surface (PES) scans at the PM6 semi-empirical level, with the following key dihedral angles varied: C11–C6–N1–C5 and C3–C14–C15–C20 for compound **1**; C3–C14–C15–C20 and C5–S13–C21–C22 for compounds **2a–e** and **2i**; C3–C14–C15–C20 and C5–S13–C21–C26 for compounds **2f–g**; C3–C14–C15–C20 and N1–C5–S13–C21 for compound **2h**; and C3–N2–C6–C9 and C15–C14–C16–C22 for compound **3**. The lowest-energy conformers identified from these scans were subjected to full geometry optimization using DFT as implemented in Gaussian 09 W^36^. Calculations were performed using the APFD functional^37^ in combination with the 6–311 + + G(d, p) basis set^38^ to ensure accurate electronic and structural characterization. The selection of the APFD functional, which incorporates the Austin-Frisch-Petersson dispersion model, was necessitated by the need to accurately capture the non-covalent interactions and electronic stabilization inherent in sulfur-containing heterocyclic systems, which standard functionals often fail to describe^37^. Coupling this with the 6−311 + + G(d, p) basis set provides a high-level treatment of the valence shells; specifically, the inclusion of diffuse functions (++) is essential for the sulfur and nitrogen atoms to properly account for lone-pair distributions, while polarization functions (d, p) are vital for describing the “soft” electron density of the 2-thiohydantoin ring^38^.To replicate experimental conditions, given that biological assays were conducted in dimethylsulphoxide (DMSO), all optimized geometries were further refined using the SMD implicit solvation model^39^. Geometry optimizations proceeded until all standard convergence criteria were satisfied, including thresholds for maximum force, root mean square (RMS) force, maximum displacement, and RMS displacement. The predicted energy changes in the final optimization steps ranged from − 4.75 × 10⁻¹¹ to − 4.58 × 10⁻¹² Hartree in the gas phase and from − 1.92 × 10⁻¹¹ to − 2.65 × 10⁻¹³ Hartree in DMSO, confirming tight convergence. Vibrational frequency analysis confirmed that all optimized structures corresponded to true energy minima on the potential energy surface, with zero imaginary frequencies observed for all compounds in both gas phase and DMSO environments. Following geometry optimization, comprehensive electronic structure analyses were conducted. Frontier molecular orbital (FMO) analysis was performed to identify the highest occupied molecular orbital (HOMO) and lowest unoccupied molecular orbital (LUMO), and the HOMO–LUMO energy gap (ΔE = E_LUMO_ − E_HOMO_) was calculated^40^. Molecular electrostatic potential (MESP) surfaces were mapped to visualize charge distribution and identify potential binding sites^41^. Global reactivity descriptors were evaluated using Multiwfn^42^, including ionization potential (I), electron affinity (A), chemical potential (µ ≈ −χ ≈ −(I + A)/2), Mulliken electronegativity (χ = (I + A)/2), chemical hardness (η ≈ I − A), softness (S = 1/η), electrophilicity index (ω = χ²/2η)^43^, and nucleophilicity (ε = 1/ω)^44^. Local reactivity indices were derived from Fukui functions calculated using Hirshfeld population analysis. The condensed Fukui functions were computed as **f**_**k**_^**+**^ = q^k^ (*N* + 1) – q^k^ (N) for sites susceptible to nucleophilic attack, **f**_**k**_^**−**^ = q^k^ (N) − q^k^ (*N* − 1) for sites susceptible to electrophilic attack, and **f**_**k**_^**0**^ = q^k^(*N* + 1) – q^k^(*N* − 1) for radical attack^43^, where q^k^​ represents the electron population at atom k in the neutral (N), anionic (*N* + 1), or cationic (*N* − 1) states. The condensed dual descriptor (CDD = f^+^ − f^−^)^45^ was employed to distinguish nucleophilic and electrophilic regions within each molecule. Additionally, quantum theory of atoms in molecules (QTAIM)^46^ and noncovalent interaction (NCI)^47^ analyses were performed using Multiwfn to probe the electronic structure and characterize intermolecular interactions. All molecular visualizations and figures were prepared using Gauss View 6^48^, VMD (Visual Molecular Dynamics)^49^, and Gnuplot^50^ to ensure publication-quality representations.

### Molecular docking 

The three-dimensional crystal structure of CDK2 complexed with ATP (PDB ID: 1HCK) was retrieved from the RCSB Protein Data Bank^51^. The protein was prepared using UCSF Chimera 1.16^52^ and AutoDockTools 1.5.7^53^ by removing water molecules, heteroatoms, and co-crystallized ligands, adding hydrogen atoms, and assigning Gasteiger charges. The structure was saved in PDBQT format. Ligands were prepared in AutoDockTools with Gasteiger charges, merged non-polar hydrogens, and defined rotatable bonds, and saved as PDBQT files^54^. Molecular docking was performed using AutoDock Vina 1.1.2^55^. The docking protocol was validated by re-docking ATP into the CDK2 active site, with RMSD ≤ 2.0 Å used as the acceptance criterion^56^. The grid box was centered on the ATP-binding pocket (center_x = 103.552 Å, center_y = 97.543 Å, center_z = 82.286 Å) with dimensions of 54 × 52 × 66 Å. Docked complexes were analyzed for hydrogen bonds and hydrophobic interactions using PyMOL(TM) 3.1.5.1^57^. and BIOVIA Discovery Studio Visualizer 2025^58^.

### Molecular dynamics simulation

The protein-ligand complex was prepared using CHARMM-GUI^59^ with the CHARMM36m force field for protein^60^ and CGenFF for ligand^61^ parameters. The system was solvated with TIP3P water^62^ in a rectangular box (minimum 10 Å from protein edges) and neutralized at 0.15 M ionic strength with Na⁺ and Cl⁻ ions. Energy minimization was performed using steepest descent (50,000 steps maximum, convergence 100.0 kJ mol⁻¹ nm⁻¹). Electrostatic interactions were calculated using PME^63^(1.2 nm cutoff, fourth-order interpolation, 0.12 nm Fourier spacing), van der Waals interactions with force-switch modifier (1.0–1.2 nm), and hydrogen bonds constrained with LINCS^64^. The system was equilibrated for 100 ps under NVT conditions at 300 K with V-rescale thermostat (0.1 ps coupling), followed by 100 ps NPT equilibration using V-rescale thermostat (0.1 ps solute, 0.5 ps solvent)^65^ and Parrinello-Rahman barostat (1.0 bar, 2.0 ps coupling, 4.5 × 10⁻⁵ bar⁻¹ compressibility)^66^. Position restraints (400.0 kJ mol⁻¹ nm⁻² backbone, 40.0 kJ mol⁻¹ nm⁻² side chains) were applied during equilibration. Production MD was run for 10 ns with a 2 fs time step using Nosé-Hoover^67,68^ thermostat (300 K, 1.0 ps coupling) and Parrinello-Rahman barostat (1.0 bar). Coordinates and energies were saved every 10 ps and 2 ps, respectively. All simulations were performed using GROMACS 2024.3^69^, with analysis conducted using GROMACS tools, PyMOL^57^, and Python (MDAnalysis^70^, Matplotlib^71^.

### MM-PBSA binding free energy calculations

The binding free energies of the CDK2–ligand complexes were calculated using the Molecular Mechanics Poisson–Boltzmann Surface Area (MM-PBSA) method implemented in gmx_MMPBSA v1.6.4. Energy evaluations were performed on snapshots extracted at regular intervals from the equilibrated portion of the 10 ns molecular dynamics trajectories.

The binding free energy was computed according to the standard thermodynamic cycle:

$${\Delta}{\mathrm{G}}_{\mathrm{bind}}=\mathrm{G}\_\mathrm{c}\mathrm{o}\mathrm{m}\mathrm{p}\mathrm{l}\mathrm{e}\mathrm{x}-(\mathrm{G}\_\mathrm{r}\mathrm{e}\mathrm{c}\mathrm{e}\mathrm{p}\mathrm{t}\mathrm{o}\mathrm{r}+\mathrm{G}\_\mathrm{l}\mathrm{i}\mathrm{g}\mathrm{a}\mathrm{n}\mathrm{d})$$

Each free energy term was decomposed into gas-phase and solvation contributions:

$$\mathrm{G}=\mathrm{G}\_\mathrm{g}\mathrm{a}\mathrm{s}+\mathrm{G}\_\mathrm{s}\mathrm{o}\mathrm{l}\mathrm{v}$$

Accordingly, the MM-PBSA binding free energy can be expressed as:

$${\Delta}\mathrm{G}\_\mathrm{b}\mathrm{i}\mathrm{n}\mathrm{d}={\Delta}\mathrm{G}\_\mathrm{g}\mathrm{a}\mathrm{s}+{\Delta}\mathrm{G}\_\mathrm{s}\mathrm{o}\mathrm{l}\mathrm{v}$$

where the gas-phase interaction energy is the sum of van der Waals and electrostatic components:

$${\Delta}\mathrm{G}\_\mathrm{g}\mathrm{a}\mathrm{s}={\Delta}\mathrm{E}\_\mathrm{V}\mathrm{D}\mathrm{W}\mathrm{A}\mathrm{A}\mathrm{L}\mathrm{S}+{\Delta}\mathrm{E}\_\mathrm{E}\mathrm{E}\mathrm{L}$$

The solvation free energy consists of polar and non-polar contributions:

$${\Delta}\mathrm{G}\_\mathrm{s}\mathrm{o}\mathrm{l}\mathrm{v}={\Delta}\mathrm{E}\_\mathrm{P}\mathrm{B}+{\Delta}\mathrm{E}\_\mathrm{N}\mathrm{P}\mathrm{O}\mathrm{L}\mathrm{A}\mathrm{R}$$

Polar solvation energies (ΔE_PB) were calculated using the Poisson–Boltzmann equation with dielectric constants of 1 (solute) and 80 (solvent). Non-polar solvation energies (ΔE_NPOLAR) were estimated from solvent-accessible surface area (SASA) calculations. Entropic contributions were excluded due to the high computational cost and limited reliability of normal-mode analysis. Standard errors of the mean (SEM) were computed to assess the statistical robustness of the binding free energy values^72–74^.

## Results and discussion

### DFT study

#### Conformational analysis and geometry optimization

The conformational analysis of the studied imidazolidinone and imidazolone derivatives, performed through systematic potential energy surface exploration of 1369 conformers generated by incremental dihedral rotations, revealed a high degree of conformational flexibility (Fig. S1). Geometry optimization of the lowest-energy conformers in gas and DMSO (Fig. S2) confirmed that all reported structures represent true minima, ensuring that the derived parameters accurately reflect thermodynamically stable configurations. Across the series (Fig. S3; Table 1), the C = O bond lengths were highly consistent, ranging from 1.203 to 1.219 Å in the gas phase and 1.206–1.218 Å in DMSO, values characteristic of sp²-hybridized carbonyl groups. The minimal variation across derivatives highlights the electronic robustness of this moiety, with solvent-induced elongation of ~ 0.005 Å reflecting stabilization of charge-separated resonance forms by DMSO. The thiocarbonyl group in the parent compound **1** retained the expected double-bond character, while its transformation into thioether substituents in compounds **2a**–**2i** was marked by elongation of C–S bonds by approximately 0.10 Å, consistent with a transition from double to single bond character. Notably, compound **3**, which maintains the thioxoimidazolidinone core, exhibited bond lengths nearly identical to compound **1**, confirming preservation of thiocarbonyl electronic structure despite substitution at the benzylidene position. Analysis of C–S–C bond angles in thioether derivatives further emphasized the influence of substituent environment, with values ranging from ~ 97° to 109°. The compressed angles in compounds **2f** and **2g** suggest steric congestion introduced by carboxylic and ester substituents, while the wide angle of **2i** (109.4°) indicates steric and electronic effects associated with the chloroacetyl group. Solvent effects on geometries were modest (< 0.02 Å in bond lengths and 0.5–2.0° in angles) but consistently indicated enhanced delocalization in DMSO.

**Table 1:** Key bond lengths (Å) and bond angles (°) for the carbonyl (C = O), thiocarbonyl/thioether (C = S/C-S), and substituent linkages (C-S-C, S-CH₂-CO) in compounds **1**, **2a**–**2i**, and **3** in gas and DMSO phase.

	C = O (Bond lengths) , (Å)		C = S or C-S (Bond lengths), (Å)		Bond angle for (C-S-C), (°)		Bond angle of (S-CH2-CO), (°)	
	DMSO	Gas	DMSO	Gas	DMSO	Gas	DMSO	Gas
**1**	1.21130	1.20632	1.66271	1.64869	-	-	-	-
**2a**	1.21541	1.21311	1.74663	1.74966	99.78797	98.20848	-	-
**2b**	1.21824	1.21179	1.76450	1.75383	99.81538	100.07528	107.39017	108.94844
**2c**	1.21812	1.21151	1.76667	1.75449	98.11597	100.05259	107.30692	108.78481
**2d**	1.21904	1.21147	1.76808	1.75454	97.69227	100.03866	108.24194	108.88897
**2e**	1.21797	1.21189	1.75052	1.75302	99.67452	98.72518	112.77824	112.29178
**2f**	1.21272	1.20684	1.74656	1.74825	97.98592	97.37953	115.32810	115.14106
**2g**	1.21381	1.20739	1.74381	1.74672	97.72867	96.79265	113.21082	112.37122
**2h**	1.21434	1.20815	1.74116	1.74565	99.46845	98.34244	-	-
**2i**	1.21348	1.20701	1.76261	1.76205	109.40726	109.44718	-	-
**3**	1.20585	1.20332	1.66262	1.64633	-	-		

#### Electronic properties: dipole moment and polarizability

The dipole moment and molecular polarizability analyses (Table 2**)** provided key insights into the electronic behavior of the 2-thiohydantoin derivatives and aligned well with the structural variations. Dipole moments varied significantly across the series, ranging from 2.34 to 6.07 D in the gas phase and 2.85–8.49 D in DMSO, reflecting substituent-dependent electronic redistribution. The parent scaffold **1**, which retains the intact thioxoimidazolidinone moiety without extended thioether substitution, exhibited the highest polarity in both phases, consistent with strong charge separation within its conjugated heterocyclic core. In contrast, **2a**, bearing a simple benzylthio substituent, showed a pronounced drop in dipole moment, indicative of lower electronegativity contrast and reduced intramolecular polarization. Introduction of electron-withdrawing or π-extended substituents in compounds **2b**–**2e** (e.g., phenacyl, nitrobenzyl, and halogenated phenyl groups) restored or increased dipolar character, confirming that resonance-assisted and inductive effects enhance charge delocalization across the thiohydantoin–thioether linkage. Notably, compound **3**, despite containing strongly electron-withdrawing malononitrile and aromatic rings, demonstrated an unexpectedly low dipole moment, which can be attributed to conformational folding that partially cancels individual bond dipoles. Across all compounds, solvation in DMSO increased dipole moments by approximately 40%, highlighting stabilization of charge-separated states in polar media. Polarizability trends further supported the influence of structural features on electronic flexibility. The most π-extended derivatives **2a**–**2e** displayed the highest polarizabilities (545–584 a.u. in DMSO), a direct consequence of their enlarged aromatic systems and enhanced electron delocalization. In contrast, compounds incorporating carboxylic **2f**, ester **2g**, aliphatic **2h**, or sterically constrained **2i** substituents exhibited lower polarizabilities due to reduced conjugation and limited electronic mobility. Compound **3**, despite possessing potent electron-withdrawing groups, showed the lowest polarizability, reinforcing the notion that its geometry restricts effective conjugation. Solvation again increased polarizability by ~ 45%, signifying solvent-facilitated electronic dispersion. From a medicinal chemistry standpoint, derivatives with both high dipole moments and high polarizabilities particularly **2b**–**2e** are expected to exhibit stronger noncovalent interactions (hydrogen bonding, dipole–dipole, π–π, and dispersion forces) with biological targets, potentially enhancing their anticancer activity compared to less polarizable analogues.

**Table 2:** Total dipole moment (TDM, Debye) and polarizability (a.u.) of compounds **1**, **2a**–**2i**, and **3** in gas and DMSO phase.

	TDM (Debye)		Poarizability (a.u.)	
	Gas	DMSO	Gas	DMSO
**1**	6.065195	8.493078	297.952333	426.900333
**2a**	2.344491	2.845015	390.906000	544.926000
**2b**	4.729037	7.707679	392.962667	566.809667
**2c**	4.129357	6.356844	406.346333	575.203667
**2d**	4.139901	6.826145	414.825000	583.901333
**2e**	4.940255	5.744489	398.512333	570.599667
**2f**	4.457764	6.355199	316.819000	449.312667
**2g**	4.704591	6.708660	346.262333	494.148333
**2h**	3.521442	4.870226	310.165667	443.582000
**2i**	4.191847	5.592133	337.852333	478.053000
**3**	3.942256	2.836104	282.989667	414.438667

#### Thermodynamic stability and solvation energies

Thermodynamic evaluation further clarified the stability of these compounds in different environments (Table 3**)**. The corrected electronic energies (E + ZPE) and Gibbs free energies (ΔG) confirmed that all compounds adopt energetically favorable conformations in both gas and solvent phases, with solvent inclusion consistently lowering the total free energy. The solvation free energies (ΔG_solv_) were uniformly negative, ranging from − 17.84 to − 24.66 kcal/mol, thereby confirming that polar solvation stabilizes the series. The degree of stabilization was strongly dependent on the nature of substituents. Compound **3** exhibited the most favorable solvation energy (–24.66 kcal/mol), attributable to its malononitrile group, which enhances dipolar interactions with polar solvents. Halogenated derivatives such as **2c** and **2d** also showed high stabilization (–22.7 and − 23.0 kcal/mol, respectively), highlighting the contribution of polarizable halogen atoms to solvent compatibility. By contrast, compound **2h**, bearing a nonpolar methyl substituent, displayed the weakest stabilization (–17.84 kcal/mol), consistent with its reduced ability to interact with the solvent. Taken together, the thermodynamic and solvation analyses indicate that electron-withdrawing or conjugated substituents enhance stability in polar media, whereas simple aliphatic groups reduce solvent compatibility. These results confirm that solvation effects considerably contribute to the thermodynamic stability of the designed molecules and must be considered when interpreting their reactivity and potential biological activity.

**Table 3:** Corrected electronic energies (E + ZPE), Gibbs free energies (ΔG), and solvation free energies (ΔG_solv_) of compounds **1**, **2a–2i**, and **3** calculated in the gas and DMSO phase.

1	(E + ZPE)_DMSO_ (Ha)	(EE + ZPE)_gas_ (Ha)	Δ G_DMSO_ (Ha)	Δ G_Gas_ (Ha)	Δ G_solvation_ (Ha)	Δ G_solv_ (K/mol)
	−1427.989238	−1427.958363	−1428.042262	−1428.010512	−0.03175	−19.92342662
**2a**	−1698.058768	−1698.026282	−1698.123679	−1698.089972	−0.033707	−21.15146272
**2b**	−1811.327820	−1811.291384	−1811.392079	−1811.357298	−0.034781	−21.82540792
**2c**	−2270.849709	−2270.809766	−2270.914275	−2270.878032	−0.036243	−22.74282681
**2d**	−4384.691946	−4384.651454	−4384.757123	−4384.72054	−0.036583	−22.95618004
**2e**	−1902.474793	−1902.439594	−1902.541633	−1902.507199	−0.034434	−21.60766212
**2f**	−1655.711981	−1655.677950	−1655.768697	−1655.734681	−0.034016	−21.34536315
**2g**	−1734.224858	−1734.191716	−1734.287914	−1734.253701	−0.034213	−21.46898252
**2h**	−1467.227334	−1467.200403	−1467.28416	−1467.255728	−0.028432	−17.8413501
**2i**	−2040.005813	−2039.972887	−2040.066569	−2040.034103	−0.032466	−20.37272343
**3**	−1652.825798	−1652.787509	−1652.884652	−1652.845356	−0.039296	−24.65861331

#### Molecular Electrostatic Potential Analysis

**Fig. 1:**
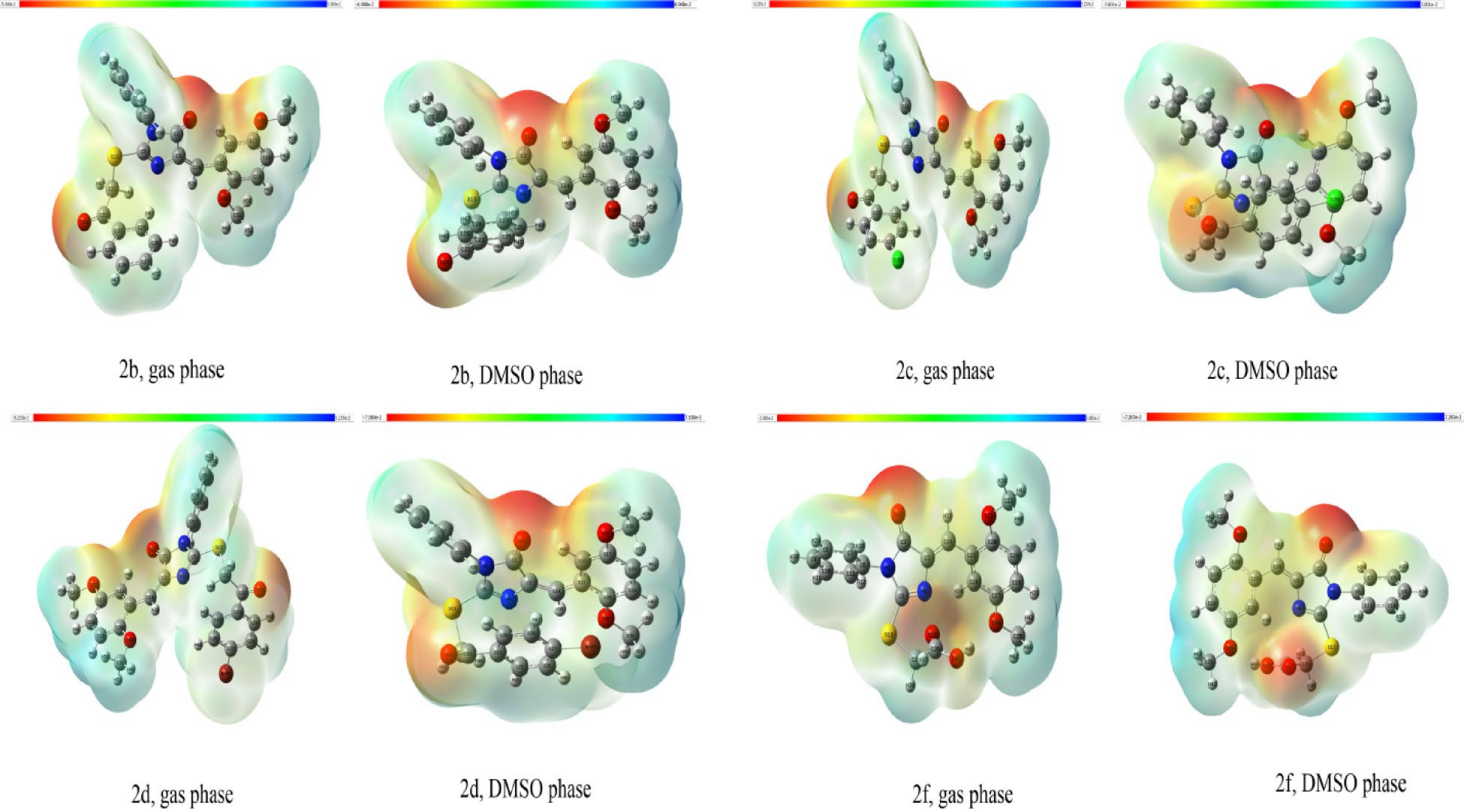
Molecular electrostatic potential (MESP) surfaces of compounds **2b**,** 2c**,** 2d**, and **2f** in gas and DMSO phase.

The molecular electrostatic potential (MESP) surfaces of compounds **1**, **2a-2i**, and **3** were computed in both DMSO and gas phases to elucidate the electronic distribution and identify potential reactive sites for intermolecular interactions (Fig. 1 and S4**)**. The MESP maps, displayed using a color-coded scale ranging from red (electron-rich, negative potential) through yellow and green (neutral) to blue (electron-deficient, positive potential), reveal distinct regional charge distributions across the molecular architectures. The electronegative regions (red) are predominantly localized around oxygen and sulfur heteroatoms, representing potential hydrogen bond acceptor sites, while electropositive regions (blue) are observed proximal to nitrogen-bound hydrogens and aromatic hydrogens, indicating hydrogen bond donor capabilities. In both phases, the carbonyl oxygen atoms and thiocarbonyl sulfur atoms exhibit intense negative potential, serving as primary hydrogen bond acceptors, whereas N-H groups in the thiohydantoin moiety display positive potential characteristic of hydrogen bond donors. Comparative analysis between gas and DMSO phases demonstrates that solvent effects significantly modulate the electrostatic potential distribution, with DMSO solvation generally diminishing the intensity of both negative and positive potential extrema due to enhanced charge stabilization through solute-solvent interactions. The thiohydantoin ring system consistently exhibits characteristic potential patterns, with the sulfur and carbonyl oxygen atoms serving as prominent electron-rich domains and potential acceptor sites. These MESP profiles provide crucial insights into the hydrogen bonding capabilities, molecular recognition patterns, and binding affinities of these compounds, facilitating rational predictions of their pharmacological behavior and supramolecular assembly patterns.

#### Frontier molecular orbital (FMO) analysis

**Fig. 2:**
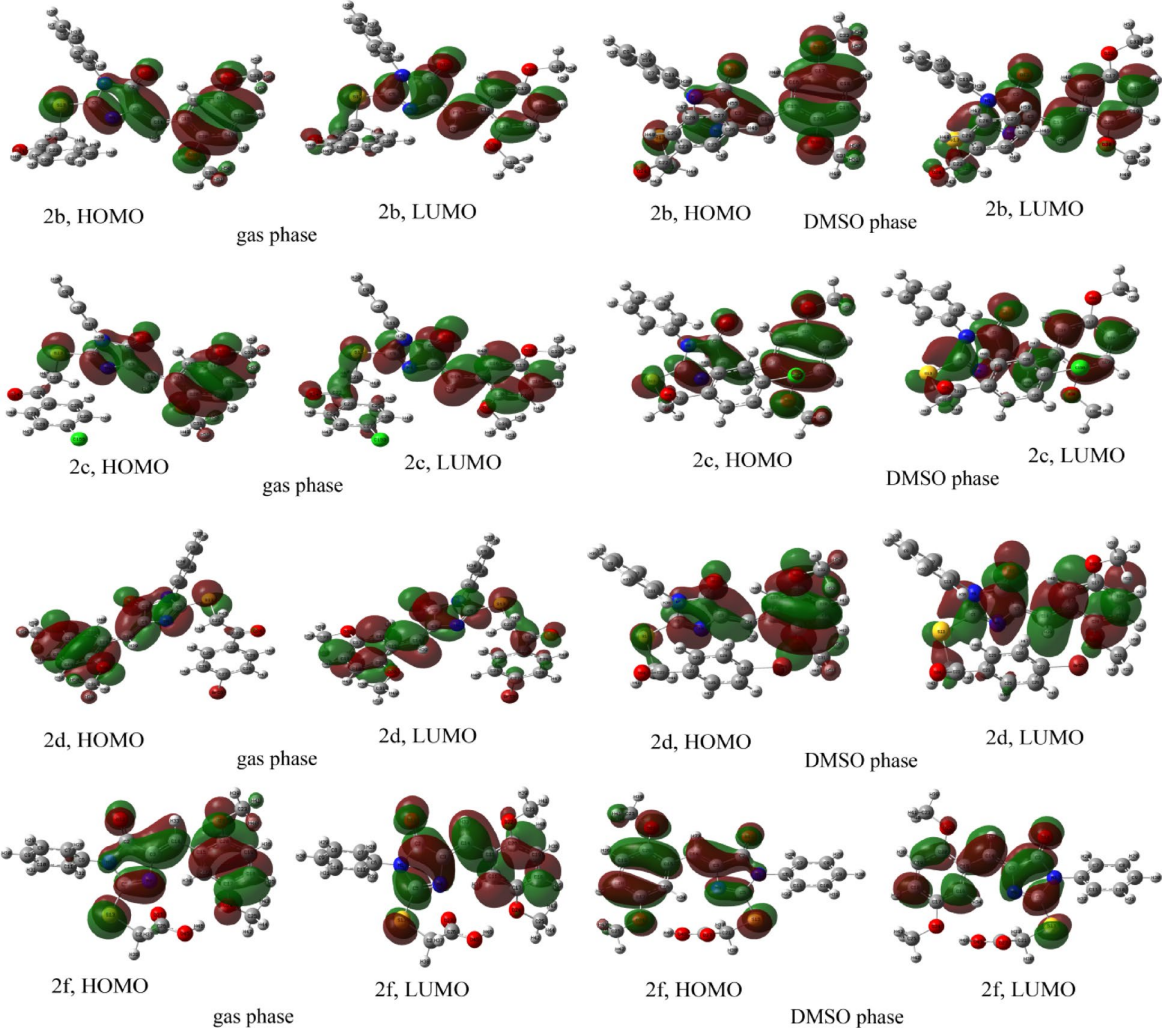
Frontier molecular orbitals (HOMO and LUMO) of compounds **2b**, **2c**, **2d**, and **2f** calculated in gas and DMSO phase.

Density functional theory calculations revealed clear trends in FMO distributions across the compound series in both the gas phase and DMSO, reflecting systematic substituent-dependent electronic effects (Fig. 2 and S5; Table 4). In the gas phase, the HOMOs were predominantly localized on the central aromatic scaffold, with electron density distributed across extended π-systems (− 5.62 to − 6.06 eV for compounds **1** and **2a**–**2i**), whereas the LUMOs were more diffusely distributed toward peripheral aromatic rings and heteroatomic regions (− 2.14 to − 2.65 eV). Introducing DMSO as a polar solvent induced measurable reorganization of the orbital contours: HOMO energies were destabilized by 0.04–0.35 eV, while LUMO energies were stabilized by 0.02–0.23 eV relative to the gas phase. This differential solvation effect consistently narrowed the HOMO–LUMO energy gaps, reducing the band-gap range from 3.18 to 3.68 eV in the gas phase to 3.02–3.55 eV in DMSO. The extent of gap reduction correlated strongly with the electron-withdrawing character of the substituents. Among the derivatives, compound **2e**, bearing a strongly electron-withdrawing nitro group, showed the greatest band-gap contraction (to 3.02 eV), driven by marked LUMO stabilization (− 2.73 eV in DMSO) and enhanced charge delocalization into the nitro-substituted phenylthio moiety. Compound **2b** also exhibited significant narrowing (3.24 eV), while the halogenated derivatives **2c** (p-Cl, 3.26 eV) and **2d** (p-Br, 3.22 eV) showed comparable effects attributable to the combined inductive and resonance contributions of halogen substituents. In contrast, derivatives bearing electron-donating or weakly withdrawing groups, including **2f** (carboxylic acid, 3.55 eV), **2g** (ethyl ester, 3.39 eV), **2h** (methylthio, 3.39 eV), and **2a** (benzylthio, 3.45 eV), retained comparatively wider band gaps. The unusually large gap of **2f** may arise from disrupted conjugation or intramolecular hydrogen bonding that limits orbital overlap, whereas the similar gaps of **2g** and **2h** reflect the modest electronic influence of their substituents. Compound **2i** (chloroacetyl) showed a modest yet notable reduction from 3.42 to 3.29 eV upon solvation, likely reflecting enhanced dipolar stabilization of its frontier orbitals. The parent scaffold **1** maintained a moderate gap of 3.52 eV, while compound **3**, bearing highly electron-withdrawing dicyano groups, displayed the largest gaps (4.68 eV in gas phase and 4.56 eV in DMSO) due to substantial HOMO stabilization. The frontier orbital analysis reveals several favorable electronic features: narrowed HOMO–LUMO gaps in electron-withdrawing derivatives, enhanced LUMO delocalization, and solvent-induced orbital stabilization. These characteristics suggest improved reactivity and charge-transfer capability that may facilitate stronger interactions with the CDK2 active site. Reduced band gaps typically correlate with increased molecular reactivity and enhanced electron mobility, properties that can promote more efficient target engagement in biological environments. Among the studied compounds, derivatives **2a-2i** exhibit the most pronounced orbital energy modulations and may therefore possess comparatively higher inhibitory potency. These computational trends provide a strong foundation for structure–activity relationship studies and warrant experimental validation.

**Table 4:** HOMO and LUMO energies and band gap values for compounds **1**, **2a-2i**, and **3** calculated in gas and DMSO phase.

Co. no	Phase	E-HOMO, eV	E-LUMO, eV	Band gap, eV
**1**	Gas	−6.0645	−2.3804	3.6841
	DMSO	−5.9278	−2.4076	3.5202
**2a**	Gas	−5.6193	−2.148	3.4713
	DMSO	−5.7579	−2.3089	3.449
**2b**	Gas	−5.8115	−2.3959	3.4156
	DMSO	−5.8288	−2.5924	3.2364
**2c**	Gas	−5.8608	−2.4618	3.399
	DMSO	−5.7846	−2.5276	3.257
**2d**	Gas	−5.862	−2.464	3.398
	DMSO	−5.769	−2.5448	3.2242
**2e**	Gas	−5.8279	−2.6525	3.1754
	DMSO	−5.7573	−2.7333	3.024
**2f**	Gas	−5.8857	−2.2161	3.6696
	DMSO	−5.8907	−2.3407	3.55
**2g**	Gas	−5.7348	−2.1992	3.5356
	DMSO	−5.7539	−2.3606	3.3933
**2h**	Gas	−5.6622	−2.1429	3.5193
	DMSO	−5.7299	−2.3404	3.3895
**2i**	Gas	−5.9769	−2.5616	3.4153
	DMSO	−5.8306	−2.5399	3.2907
**3**	Gas	−6.4521	−1.7766	4.6755
	DMSO	−5.9856	−1.421	4.5646

#### DFT-based evaluation of electronic properties and molecular reactivity parameters

The global reactivity indices calculated using density functional theory reveal significant insights into the electronic properties and potential anticancer activity of the investigated compounds (Table 5). In the gas phase, the ionization potential (I) ranges from 6.85 to 7.86 eV, while electron affinity (A) varies between 0.46 and 1.42 eV, indicating moderate electron-donating and electron-accepting capabilities. The chemical hardness (η) values spanning 5.65–7.39 eV suggest relatively stable molecular frameworks resistant to electronic perturbations. Upon transitioning to the DMSO phase, which better mimics the biological environment, notable changes are observed: ionization potentials decrease to 5.50–5.78 eV, electron affinities increase substantially to 1.68–2.90 eV, and chemical hardness decreases to 2.66–4.10 eV, collectively indicating enhanced reactivity in polar media. Particularly significant is the electrophilicity index (ω), which increases from 1.17 to 1.60 eV in gas phase to 1.69–3.35 eV in DMSO, with compounds **2b**, **2c**, **2d**, **2e**, and **2i** exhibiting the highest electrophilicity values (> 3.20 eV). This enhanced electrophilic character is crucial for anticancer activity, as it facilitates interactions with nucleophilic biomolecular targets such as DNA bases and amino acid residues in proteins, potentially leading to cytotoxic effects. The inverse relationship between chemical hardness and biological activity, as postulated by Pearson’s hard-soft acid-base principle, suggests that compounds with lower η and higher ω values in DMSO phase may demonstrate superior anticancer efficacy through more favorable interactions with cellular nucleophiles, disrupting critical biochemical pathways in cancer cells.

**Table 5:** Global reactivity descriptors calculated at the DFT level for compounds **1**, **2a-2i**, and **3** in gas and DMSO phase. All energies are expressed in Hartrees (HA), while reactivity indices are reported in electron volts (eV).

Parameter	Phase	1	2a	2b	2c	2d	2e	2f	2 g	2 h	2i	3
E(N), HA	Gas	−1428.27	−1698.44	−1811.72	−2271.23	−4385.07	−1902.86	−1656.03	−1734.60	−1467.54	−2040.31	−1653.15
	DMSO	−1428.30	−1698.48	−1811.76	−2271.27	−4385.11	−1902.89	−1656.06	−1734.63	−1467.56	−2040.34	−1653.19
E(N + 1), HA	Gas	−1428.31	−1698.48	−1811.76	−2271.28	−4385.12	−1902.91	−1656.06	−1734.63	−1467.57	−2040.36	−1653.16
	DMSO	−1428.40	−1698.57	−1811.86	−2271.37	−4385.21	−1903.00	−1656.16	−1734.73	−1467.66	−2040.45	−1653.25
E(N-1), HA	Gas	−1428.00	−1698.19	−1811.46	−2270.97	−4384.81	−1902.60	−1655.77	−1734.34	−1467.28	−2040.04	−1652.86
	DMSO	−1428.09	−1698.27	−1811.55	−2271.06	−4384.91	−1902.69	−1655.86	−1734.43	−1467.36	−2040.14	−1652.97
I, eV	Gas	7.36	6.85	7.04	7.09	7.09	7.07	7.16	7.00	6.95	7.27	7.86
	DMSO	5.67	5.50	5.57	5.55	5.55	5.50	5.64	5.50	5.47	5.57	5.78
A, eV	Gas	1.09	0.90	1.22	1.31	1.32	1.42	0.93	0.94	0.86	1.31	0.46
	DMSO	2.70	2.62	2.90	2.81	2.82	2.84	2.64	2.66	2.65	2.84	1.68
χ, eV	Gas	4.22	3.88	4.13	4.20	4.21	4.24	4.04	3.97	3.90	4.29	4.16
	DMSO	4.18	4.06	4.23	4.18	4.19	4.17	4.14	4.08	4.06	4.21	3.73
µ, eV	Gas	−4.22	−3.88	−4.13	−4.20	−4.21	−4.24	−4.04	−3.97	−3.90	−4.29	−4.16
	DMSO	−4.18	−4.06	−4.23	−4.18	−4.19	−4.17	−4.14	−4.08	−4.06	−4.21	−3.73
η, eV	Gas	6.28	5.95	5.82	5.78	5.77	5.65	6.23	6.06	6.09	5.95	7.39
	DMSO	2.97	2.89	2.68	2.74	2.73	2.66	3.00	2.84	2.82	2.73	4.10
S, eV⁻¹	Gas	0.16	0.17	0.17	0.17	0.17	0.18	0.16	0.17	0.16	0.17	0.14
	DMSO	0.34	0.35	0.37	0.37	0.37	0.38	0.33	0.35	0.35	0.37	0.24
ω, eV	Gas	1.42	1.26	1.46	1.53	1.53	1.60	1.31	1.30	1.25	1.55	1.17
	DMSO	2.94	2.85	3.35	3.20	3.21	3.27	2.85	2.93	2.92	3.24	1.69
ε, eV	Gas	0.70	0.79	0.68	0.66	0.65	0.63	0.76	0.77	0.80	0.65	0.85
	DMSO	0.34	0.35	0.30	0.31	0.31	0.31	0.35	0.34	0.34	0.31	0.59

E(N) = ground state energy; E(*N* + 1) = energy of anionic species; E(N-1) = energy of cationic species; I = ionization potential; A = electron affinity; χ = electronegativity; µ = chemical potential; η = global hardness; S = global softness; ω = electrophilicity index; ε = nucleophilicity index.

#### Fukui function and condensed-to-atom dual descriptor analysis

**Fig. 3:**
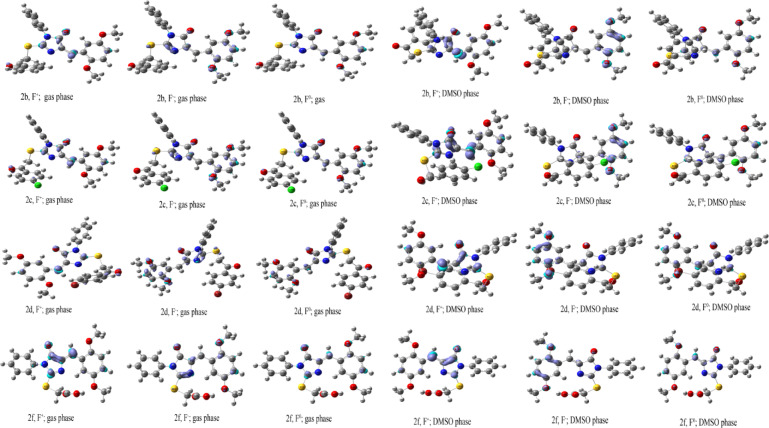
Fukui functions and local reactivity indices for compounds **2b**, **2c**, **2d**, and **2f** in gas and DMSO phase. Site-specific reactivity descriptors for electrophilic (f⁻), nucleophilic (f⁺), and radical (f⁰) attacks derived from Hirshfeld charges.

The condensed-to-atom dual descriptor (CDD) values derived from Fukui function analysis reveal distinct electrophilic and nucleophilic centers with notable solvent-dependent modulations. Positive CDD values indicate electrophilic centers (susceptible to nucleophilic attack), while negative values indicate nucleophilic centers (susceptible to electrophilic attack)^45^ (Tables S1-S22, Fig. 3 and S6). For compound **1**, dominant electrophilic centers were C2 (CDD = + 0.0531 gas, + 0.0634 DMSO), O12 (+ 0.0473 gas, + 0.0631 DMSO), and C14 (+ 0.0276 gas, + 0.0790 DMSO). S13 exhibited solvent-driven inversion from nucleophilic (− 0.0359 gas) to electrophilic (+ 0.0612 DMSO). N4 remained the principal nucleophilic site (− 0.0331 gas, − 0.0091 DMSO), with C17, C20, O21, and O23 showing enhanced nucleophilicity in DMSO. Compound **2a** showed moderate gas-phase electrophilicity at C2 (+ 0.0413), O12 (+ 0.0282), and C8 (+ 0.0017), with N4 (− 0.0284) as the primary nucleophilic center. DMSO dramatically amplified reactivity: C2 (+ 0.2300), C6 (+ 0.2164), and C8 (+ 0.2228) became highly electrophilic, while N4 (− 0.2151) and O12 (− 0.2309) became strongly nucleophilic. For compounds **2b–d**, similar patterns emerged with C14, C2, and O12 as main electrophilic sites, while S13 consistently underwent solvent-induced inversion from nucleophilic (gas phase) to electrophilic (DMSO). Nucleophilic centers C17, C20, O30, and O32 intensified in DMSO. Halogen substituents showed moderate electrophilicity: Cl55 in 2c (+ 0.0186 gas, − 0.0012 DMSO) and Br55 in 2 d (+ 0.0234 gas, + 0.0003 DMSO). Compound **2e** displayed unique nitro group-dominated electrophilicity: O34 (+ 0.0693 gas, + 0.1664 DMSO), O33 (+ 0.0655 gas, + 0.1662 DMSO), and N32 (+ 0.0465 gas, + 0.1167 DMSO). S13 served as the dominant nucleophilic site (− 0.0411 gas, − 0.0284 DMSO), with C20 showing intensified nucleophilicity in DMSO (− 0.0712). Compounds **2f–h** exhibited C14, C2, and O12 as primary electrophilic centers with characteristic S13 inversion in DMSO. Nucleophilic sites C17, C20, and oxygen atoms (O21–O24) showed enhanced polarization in polar solvent. Compound **2i** displayed electrophilic centers at C14 (+ 0.0700 gas, + 0.0980 DMSO), C2(+ 0.0313 gas, + 0.0466 DMSO), and O12 (+ 0.0179 gas, + 0.0424 DMSO), with S13 shifting from nearly neutral (− 0.0028 gas) to clearly electrophilic (+ 0.0219 DMSO). Nucleophilic centers O21, O23, C17, and C20 intensified in DMSO. The chloroacetyl moiety showed moderate electrophilicity at C40 and Cl45. Compound **3** showed dominant electrophilic sites at O7 (+ 0.0675 gas, + 0.0985 DMSO), C1 (+ 0.0651 gas, + 0.0914 DMSO), C3 (+ 0.0628 gas, + 0.0993 DMSO), and S8 (+ 0.0357 gas, + 0.2028 DMSO). S8 exhibited remarkable solvent-dependent amplification (nearly sixfold increase). Nucleophilic centers O28, O26, C17, and C20 intensified significantly in DMSO, demonstrating pronounced charge separation in the polar environment. These analyses reveal critical site-specific reactivity patterns with systematic solvent-dependent modulation of electrophilic and nucleophilic centers, particularly the characteristic sulfur inversion behavior, providing essential insights for optimizing biological interactions in anticancer drug development.

#### QTAIM topological parameters

##### Nuclear critical points

**Fig. 4:**
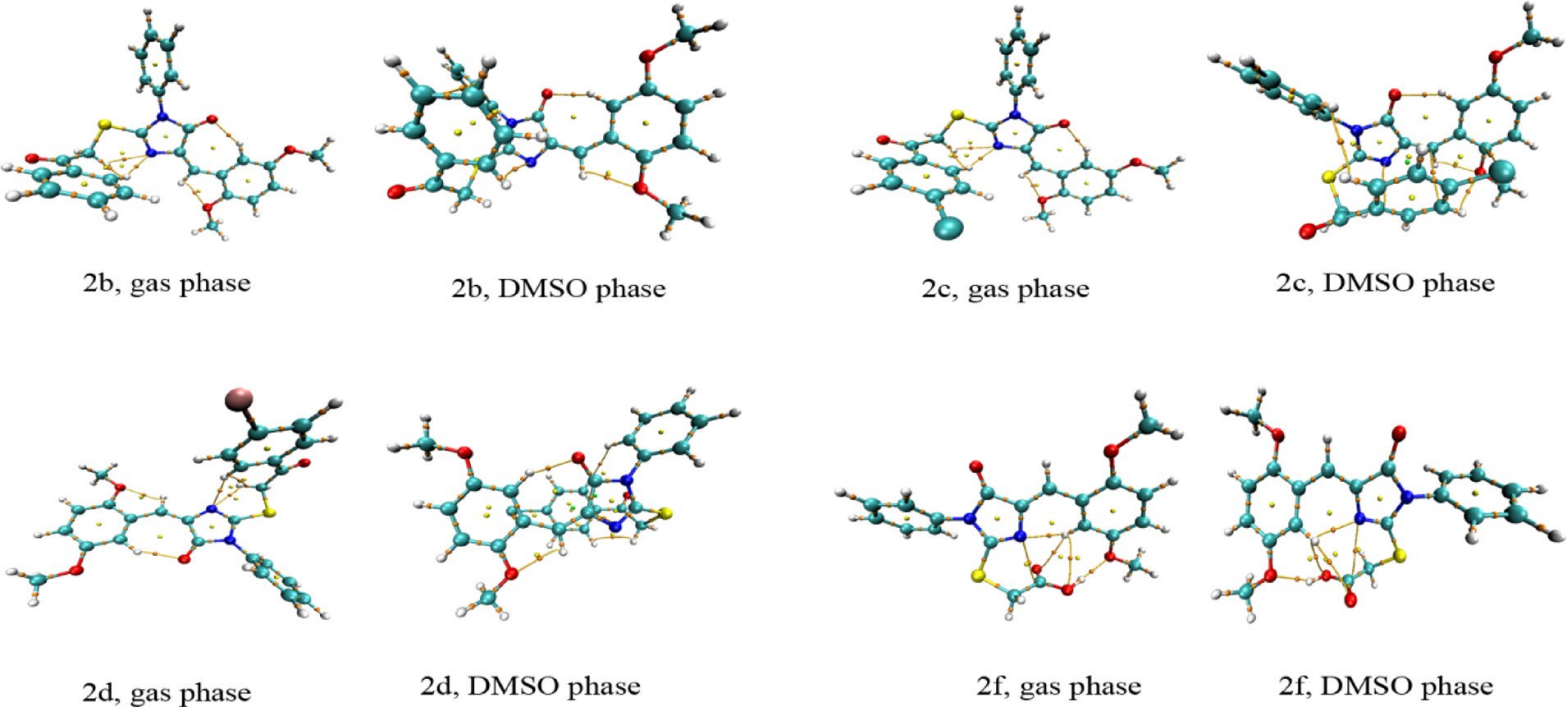
Molecular graphs illustrating the nuclear critical points (NCPs), bond critical points (BCPs), and associated bond paths for compounds **2b**, **2c**, **2d**, and **2f** (Gas and DMSO phase).

The Quantum Theory of Atoms in Molecules (QTAIM) analysis performed at nuclear critical points (NCPs) provides fundamental insight into the intrinsic electronic structure characteristics of compounds **1**, **2a–2i**, and **3** in both gas and DMSO phases^75–77^(**Tables S23-S44**, Fig. 4 and S7). The electron density (ρ) values at NCPs follow periodic trends, with heavy atoms exhibiting substantially higher electron density than light atoms. Sulfur centers consistently present the highest electron densities (~ 2588 a.u.) and strongly negative Laplacian values (∇²ρ ≈ − 1.44 × 10⁸ a.u.), reflecting pronounced nuclear charge concentration and core electron effects. Oxygen atoms display intermediate ρ values (~ 295 a.u.) with Laplacians around − 2.46 × 10⁶ a.u., while nitrogen atoms exhibit slightly lower densities (~ 194 a.u.; ∇²ρ ≈ − 1.21 × 10⁶ a.u.), consistent with their reduced core electron contributions. Carbon atoms maintain relatively uniform electron densities (119.43–119.94 a.u.) and Laplacians (–5.44 × 10⁵ to − 5.46 × 10⁵ a.u.) across all compounds, highlighting the conserved electronic environment within the aromatic and conjugated frameworks. This uniformity supports structural stabilization and suggests favorable π–π stacking potential in biological settings. Hydrogen atoms, as expected, show the lowest electron densities (0.39–0.44 a.u.) with modestly negative Laplacians (–23 to − 26 a.u.). The average local ionization energy (ALIE) values further elucidate atom-specific reactivity patterns. Sulfur atoms exhibit exceptionally high ALIE values (~ 82.6 a.u.), indicating limited susceptibility to electrophilic attack and a primarily structural role. Oxygen atoms show ALIE values around 18.3 a.u., establishing them as primary hydrogen-bond acceptors, particularly those with the highest ALIE values [O(23) and O(21) in compound **1**, O(28) and O(30) in compound **2a**, and O(26) in compound **3**] which represent the most favorable H-bonding sites. Carbonyl oxygens such as O(12), with slightly lower ALIE values (18.29–18.32 a.u.), serve as important secondary acceptor centers. Nitrogen atoms exhibit dual reactivity: N(4) centers (ALIE ≈ 13.76–13.79 a.u.) represent the most nucleophilic and reactive nitrogen sites across the series, while N(1) atoms (ALIE ≈ 13.87–13.89 a.u.) are more prone to protonation and ionic interactions. Unique nitrogen centers in compound **3** (N(23) and N(25)) show slightly enhanced nucleophilicity, suggesting potential involvement in covalent or coordination-driven interactions. Hydrogen atoms with unusually elevated ALIE values (0.63–0.67 a.u.), such as H(34) in compound **1** and H(46) in compound **2f**, act as strong hydrogen-bond donors and may participate in proton-transfer mechanisms. Halogen-substituted derivatives introduce distinctive electronic features: chlorine atoms in compounds **2c** and **2i** (ALIE ≈ 94.2 a.u.) and bromine in compound **2d** (ALIE ≈ 438.1 a.u.) act as σ-hole donors capable of forming halogen bonds, while their high electron density (ρ ≈ 3122 a.u. for Cl and 28,736 a.u. for Br) contributes to increased lipophilicity and directional interactions with electron-rich protein residues. The nitro substituent in compound **2e** also introduces characteristic electron density patterns at the nitrogen center (ρ ≈ 194.3 a.u.; ALIE ≈ 14.0 a.u.), reflecting its electron-withdrawing nature and potential influence on overall molecular reactivity. Comparison between gas and DMSO phases reveals only minor variations in QTAIM parameters, indicating that solvation does not fundamentally alter the electronic character of nuclear sites. Slight shifts in Laplacian values reflect solvent-induced polarization, which may modulate intermolecular interactions without affecting intrinsic electronic stability. Collectively, the QTAIM–NCP analysis confirms the structural integrity of the designed scaffolds and identifies the principal reactive centers, high-ALIE oxygen atoms, nucleophilic N(4) sites, acidic hydrogens, halogen substituents, and aromatic carbons, as key contributors to biological recognition. These features are expected to enhance binding affinity through a combination of hydrogen bonding, halogen bonding, π–π stacking, and protonation-dependent interactions, supporting the potential of these compounds as promising anticancer candidates.

##### Bond critical points

The quantum theory of atoms in molecules (QTAIM) analysis at bond critical points (BCPs) provides crucial insights into the electronic structure and potential biological reactivity of the synthesized compounds^75–77^ (Tables S45-S66, Fig. 4 and S7). The electron density ρ(r) values for covalent bonds range from 0.17 to 0.51 a.u., with C-C, C-N, and C-O bonds displaying typical values between 0.27 and 0.33 a.u., indicative of stable σ-bonding interactions. Notably, the carbonyl groups (C = O) exhibit significantly higher electron densities (0.40–0.43 a.u.) and more negative total energy densities H(r) (−0.68 to −0.72 a.u.), confirming their electrophilic character and potential as hydrogen bond acceptors in biological environments. The presence of intramolecular hydrogen bonds is evident from specific BCPs with characteristic low electron densities (0.01–0.03 a.u.) and positive Laplacian values (0.04–0.12 a.u.), particularly between N-H···O and O-H···O moieties. These interactions contribute to conformational stability and preorganization for target binding. For instance, compound **1** displays an intramolecular H-bond between 21(O) and 25(H) with ρ(r) = 0.03 a.u. and ∇²ρ(r) = 0.12 a.u. in the gas phase, which is maintained in DMSO, suggesting structural persistence across different biological microenvironments. The C-S bonds connecting the thiazole ring to adjacent moieties show electron densities of 0.17–0.22 a.u. with slightly positive Laplacian values, indicating polarized covalent character. This polarization enhances the nucleophilicity of sulfur atoms, potentially facilitating coordination with metal centers in metalloproteins or interaction with cysteine-rich domains of biological targets. The ellipticity values (ε) for aromatic C-C bonds range from 0.18 to 0.32, confirming π-electron delocalization, which is crucial for π-π stacking interactions with aromatic amino acids (Phe, Tyr, Trp) in protein binding sites. Solvent effects, as evidenced by comparing gas and DMSO phase data, reveal systematic changes in electron density and energy descriptors. Polar DMSO generally reduces electron densities at BCPs by 0.01–0.02 a.u. and makes total energy densities less negative, reflecting enhanced solvation and weakening of certain interactions. This suggests that the compounds maintain sufficient structural integrity while exhibiting flexibility for induced-fit binding to biological targets. The substitution patterns in compounds **2a-2i** introduce diverse electronic effects. Halogen substituents (Cl, Br) exhibit characteristic C-X bonds with ρ(r) = 0.16–0.20 a.u. and serve as σ-hole donors for halogen bonding, as evidenced by weak interactions with oxygen atoms (ρ(r) ≈ 0.00–0.01.00.01 a.u.). The nitro-substituted compound **2e** shows enhanced electron-withdrawing effects, with N-O bonds displaying ρ(r) = 0.50–0.51 a.u. and highly negative H(r) values (−0.66 to −0.67 a.u.), significantly increasing the electrophilicity of adjacent positions and potentially enhancing Michael acceptor reactivity. Mechanistically, these compounds can exert anticancer activity through complementary interactions, including hydrogen bonding via N–H and C = O groups, π–π stacking through their conjugated aromatic systems, metal coordination through nitrogen and sulfur lone pairs, and halogen bonding that enhances binding selectivity. Their electrophilic centers may also enable covalent modification of nucleophilic residues such as Cys, Lys, or Ser. Together, these features create a versatile scaffold capable of engaging diverse cancer-related targets.

#### Intramolecular non-covalent interaction analysis and conformational stability

**Fig. 5:**
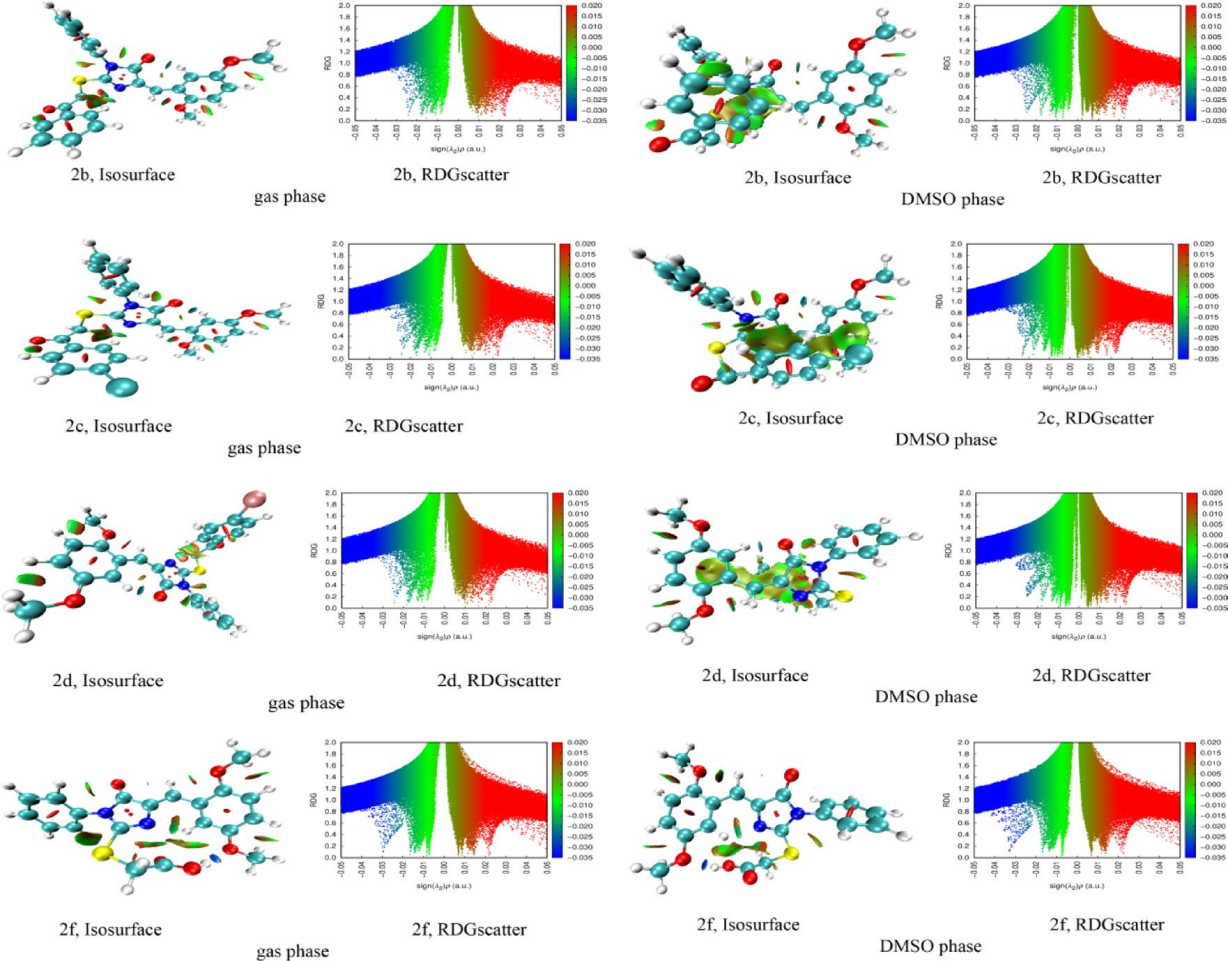
NCI isosurfaces and RDG scatter plots for compounds **2b**, **2c**, **2d**, and **2f** in the gas and DMSO phase.

The intramolecular non-covalent interaction (NCI) analysis, conducted using reduced density gradient (RDG) methodology in both gas and DMSO phases, provided critical insights into the conformational stability and three-dimensional structural features of compounds **1**, **2a-2i**, and **3** that are essential prerequisites for optimal receptor binding (Fig. 5 and S8**)**. The NCI isosurfaces, color-coded according to the sign(λ2)ρ descriptor, revealed extensive intramolecular stabilization patterns with green surfaces predominating around the molecular scaffolds, indicating favorable weak van der Waals interactions and dispersion forces that maintain the preferred bioactive conformations. Blue isosurfaces, observed particularly in compounds **1** and **2a-f**, correspond to strong intramolecular hydrogen bonding between proximate carbonyl, hydroxyl, amino, and aromatic nitrogen functionalities, which rigidify the molecular architecture and pre-organize pharmacophoric elements for target recognition^47^. These intramolecular H-bonds effectively lock the molecules into spatially defined geometries, reducing the entropic penalty upon binding to anticancer protein targets. The yellow-green isosurfaces localized between aromatic rings and aliphatic chains suggest stabilizing CH-π and π-π stacking interactions that contribute to the overall conformational preference. Red regions, indicative of steric repulsion, were minimal across the series, confirming energetically favorable intramolecular arrangements. Notably, the RDG scatter plots exhibited characteristic distributions with sign(λ2)ρ values ranging from − 0.035 to + 0.020 a.u., where the blue spike region (negative sign(λ2)ρ) corresponds to attractive interactions and the red region (positive sign(λ2)ρ) represents repulsive contacts. Comparison between gas and DMSO phases revealed that polar solvation moderately modulated the strength of intramolecular interactions, particularly attenuating hydrogen bonding intensities as evidenced by reduced blue isosurface volumes in DMSO, though the overall conformational integrity remained preserved. These intramolecular NCI features collectively ensure that the designed compounds maintain conformationally optimized pharmacophoric presentations, minimizing conformational flexibility and maximizing shape complementarity with biological targets, critical determinants for potent anticancer activity. The pre-organized three-dimensional architectures revealed by this analysis rationalize the anticipated binding efficiency and provide a molecular basis for structure-activity relationships in the anticancer evaluation of this compound series.

### Molecular docking analysis of designed compounds aganist target CDK2

**Fig. 6:**
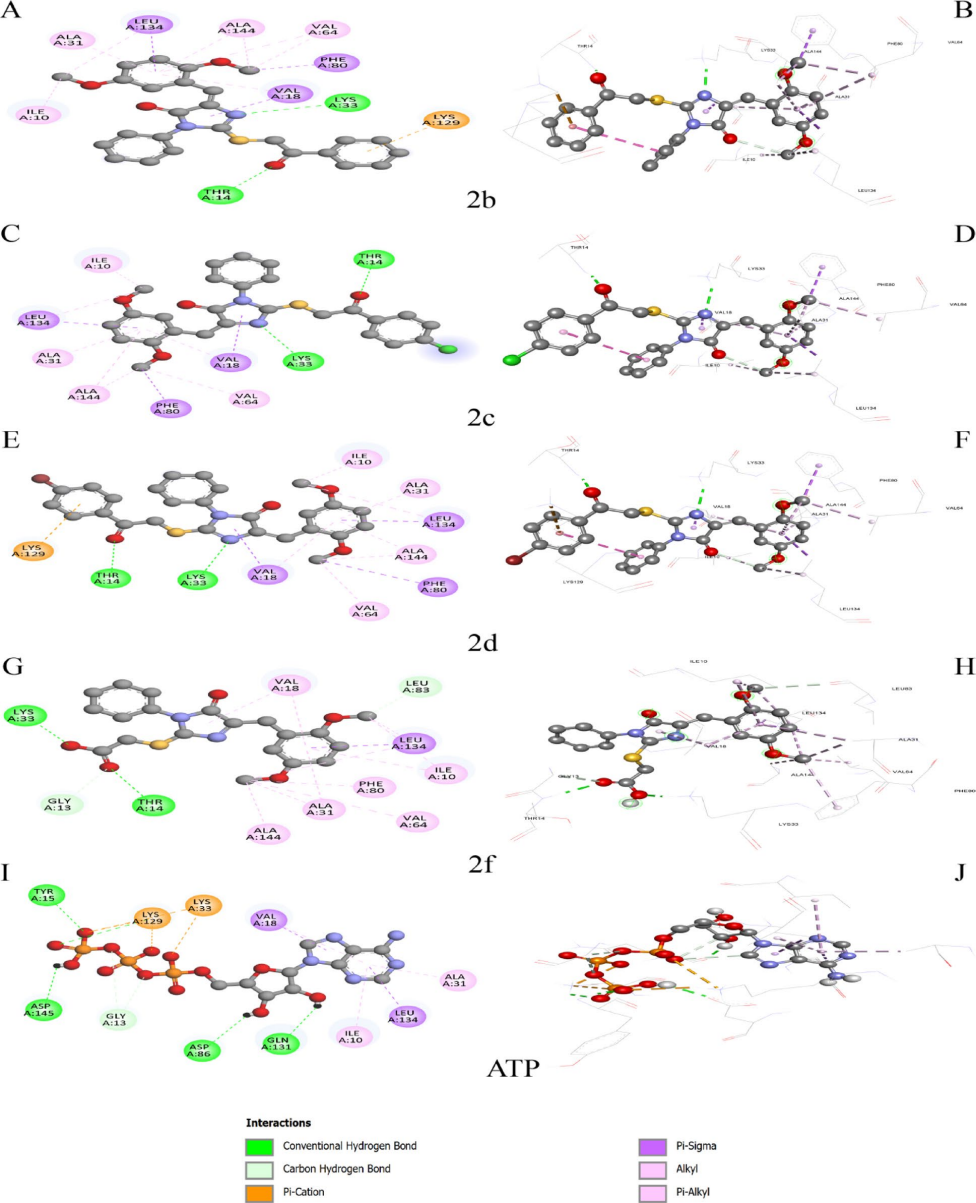
2D and 3D binding interaction diagrams of compounds **2b**, **2c**, **2d**, **2f**, and **ATP** with CDK2 (PDB: 1HCK).

**Fig. 7:**
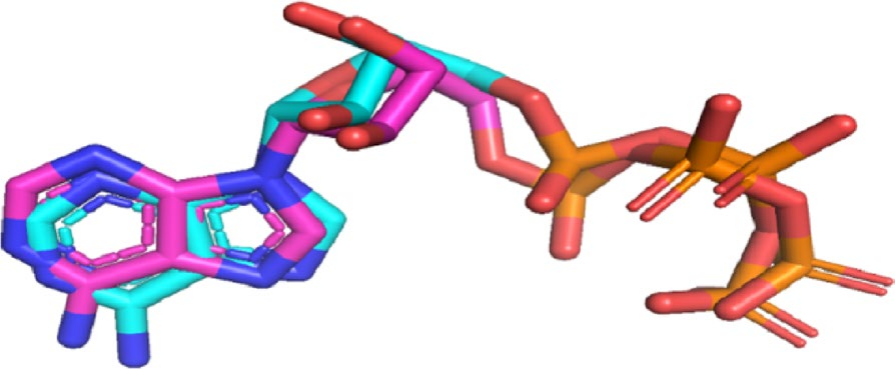
Validation of the molecular docking protocol. Superimposition of the co-crystallized ATP (magenta) and re-docked ATP (cyan) in the CDK2 active site, showing excellent alignment with RMSD = **1.024** Å.

Molecular docking was performed to evaluate the binding affinities and interaction patterns of the designed compounds (**1**, **2a-2i**, and **3**) within the ATP-binding site of cyclin-dependent kinase 2 (CDK2, PDB ID: 1HCK), using ATP (Adenosine-5’-Triphosphate) as a reference ligand (Fig. 6, S9 and S10). The docking protocol was validated by redocking ATP, yielding an RMSD of 1.024 Å (Fig. 7), confirming the reliability and reproducibility of the methodology. The compounds exhibited binding energies ranging from − 6.664 to − 9.312 kcal/mol, with several derivatives showing stronger predicted affinity than ATP (− 8.460 kcal/mol) (Table 6). Compounds **2c**, **2d**, and **2b** emerged as the most potent candidates, displaying binding energies of − 9.312, − 9.303, and − 9.269 kcal/mol, respectively. These ligands adopted highly similar binding poses and consistently formed two key hydrogen bonds with Lys33 and Thr14, critical residues within the ATP-binding pocket. In addition, these compounds established extensive hydrophobic interactions with conserved residues, including Ile10, Val18, Ala31, Val64, Phe80, Leu134, and Ala144, providing substantial stabilization of the ligand–protein complexes. Compounds **2b** and **2d** also engaged Lys129 through additional hydrophobic contacts, potentially enhancing complex stability. Compound **2e** showed the fourth-highest affinity (− 9.090 kcal/mol), retaining the essential Lys33 hydrogen bond and forming an additional carbon–hydrogen interaction with Val164, while maintaining a hydrophobic network comparable to the top-ranked compounds. Compound **2a** exhibited a lower affinity (− 8.595 kcal/mol) and formed only a single hydrogen bond with Lys33, suggesting that loss of the secondary interaction with Thr14 contributed to its reduced binding. Compounds **2f** and **2g** displayed intermediate affinities (− 8.175 and − 8.462 kcal/mol, respectively) with distinct interaction profiles: **2f** formed hydrogen bonds with Lys33 and Thr14 and additional carbon–hydrogen contacts with Gly13 and Leu83, whereas **2g** interacted with Lys33 and Tyr15 and formed carbon–hydrogen bonds with Leu83 and Asn132, but exhibited fewer hydrophobic contacts, consistent with its moderate affinity. The parent compound **1** showed weak binding (− 7.792 kcal/mol) and lacked hydrogen bond interactions, relying exclusively on hydrophobic contacts, emphasizing the importance of directed hydrogen bonding for potent CDK2 inhibition. Similarly, compound **3** demonstrated the lowest affinity (− 6.664 kcal/mol), with hydrogen bonding limited to Glu12 rather than Lys33, indicating a suboptimal binding orientation. Compounds **2h** and **2i** displayed moderate affinities (− 7.949 and − 8.105 kcal/mol) and formed carbon–hydrogen bonds with His84, with **2i** additionally interacting with Glu12, deviating from the optimal Lys33/Thr14 interaction motif. Collectively, the docking data identify compounds **2c**, **2d**, and **2b** as the most promising CDK2 inhibitors, characterized by superior binding affinities, strategic hydrogen bonding with key catalytic residues, and extensive hydrophobic stabilization within the ATP-binding cleft. Among the evaluated compounds, compound **2c** is predicted to be the most potent CDK2 inhibitor based on its superior binding affinity and optimal interaction profile. The improved binding affinity of the lead compounds relative to the parent scaffold (Δ binding affinity ≈ − 1.52 kcal/mol) supports the effectiveness of the rational design strategy, warranting further in vitro enzymatic and cell-based validation.

**Table 6:** Binding affinity and interaction profile of test compounds with CDK2.

Compound	Binding Affinity (kcal/mol)	H-Bond Interactions	Hydrophobic Interactions	Number of H-bonds
**1**	−7.792	None	Ile10, Val18, Ala31, Lys33, Val64, Phe80, Leu134	0
**2a**	−8.595	Lys33	Ile10, Val18, Ala31, Val64, Phe80, Leu134, Ala144	1
**2b**	**−9.269**	Lys33, Thr14	Ile10, Val18, Ala31, Val64, Phe80, Lys129, Leu134, Ala144	2
**2c**	**−9.312**	Lys33, Thr14	Ile10, Val18, Ala31, Val64, Phe80, Leu134, Ala144	2
**2d**	**−9.303**	Lys33, Thr14	Ile10, Val18, Ala31, Val64, Phe80, Lys129, Leu134, Ala144	2
**2e**	**−9.090**	Lys33, Val164ᵃ	Ile10, Val18, Ala31, Val64, Phe80, Lys129, Leu134, Ala144	2
**2f**	−8.175	Lys33, Thr14, Gly13ᵃ, Leu83ᵃ	Ile10, Val18, Ala31, Val64, Phe80, Leu134, Ala144	4
**2g**	−8.462	Lys33, Tyr15, Leu83ᵃ, Asn132ᵃ	Ile10, Val18, Ala31, Val64, Phe80, Leu134	4
**2h**	−7.949	Glu81ᵃ, His84ᵃ	Ile10, Val18, Ala31, Lys33, Val64, Phe80, Leu134	2
**2i**	−8.105	Glu12, His84ᵃ	Ile10, Val18, Ala31, Lys33, Val64, Phe80, Leu134	2
**3**	−6.664	Glu12, Asn132ᵃ, Glu162ᵃ	Val18, Ala144	3
**ATP**	−8.460	Tyr15, Asp86, Gln131, Asp145, Gly13ᵃ	Ile10, Val18, Ala31, Lys33, Lys129, Leu134	5

ᵃ Carbon hydrogen bond.

**RMSD: **1.024 Å.

### Molecular dynamics simulation analysis

**Fig. 8:**
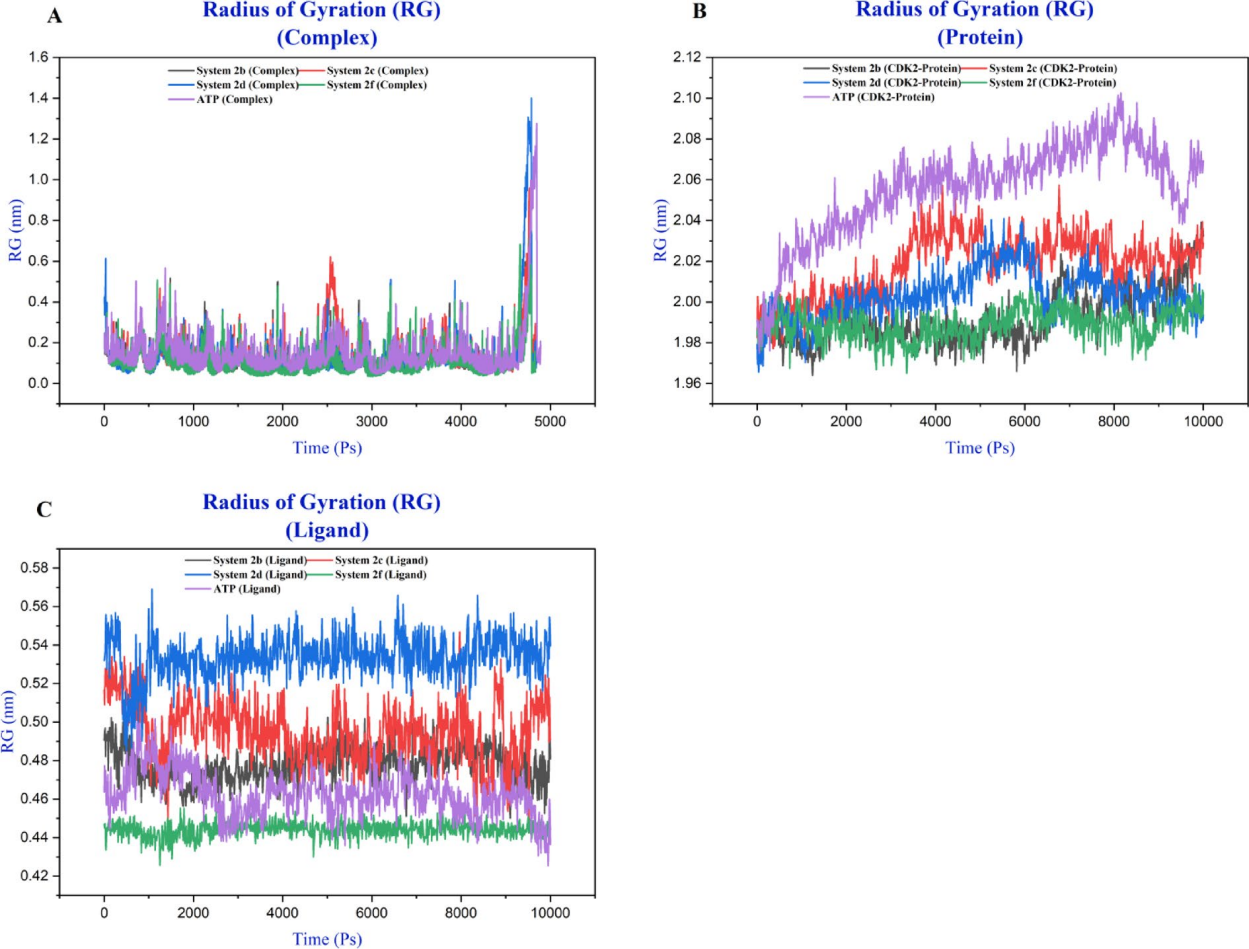
RG profiles of CDK2- Ligand complex (**A**), CDK2 protein (**B**) and ligands (**2b**,** 2c**,** 2d**, **2f** and **ATP**) (**C**) over the 10 ns molecular dynamics simulations.

The 10 ns molecular dynamics simulations provided comprehensive insights into the structural dynamics and binding characteristics of CDK2-ligand complexes (Fig. S11). Radius of gyration analysis (Fig. 8 and S12-14) demonstrated that CDK2 maintained remarkable structural integrity across all systems, with protein Rg values consistently fluctuating between 1.96 and 2.10 nm. The ATP-bound system exhibited progressive expansion from 2.00 nm to 2.06–2.10 nm after 2 ns, suggesting ATP binding induces a more expanded protein conformation. Ligand Rg values ranged from 0.40 to 0.57 nm, with compounds **2d** and **2e** displaying the largest values (0.52–0.55 nm). Notably, the complex Rg analysis revealed dissociation events around 5 ns where several systems, particularly compounds **3**, **2b**, **2d** and **ATP**, showed spikes reaching 0.95–1.50 nm, indicating partial or complete ligand exit from the binding pocket.

**Fig. 9:**
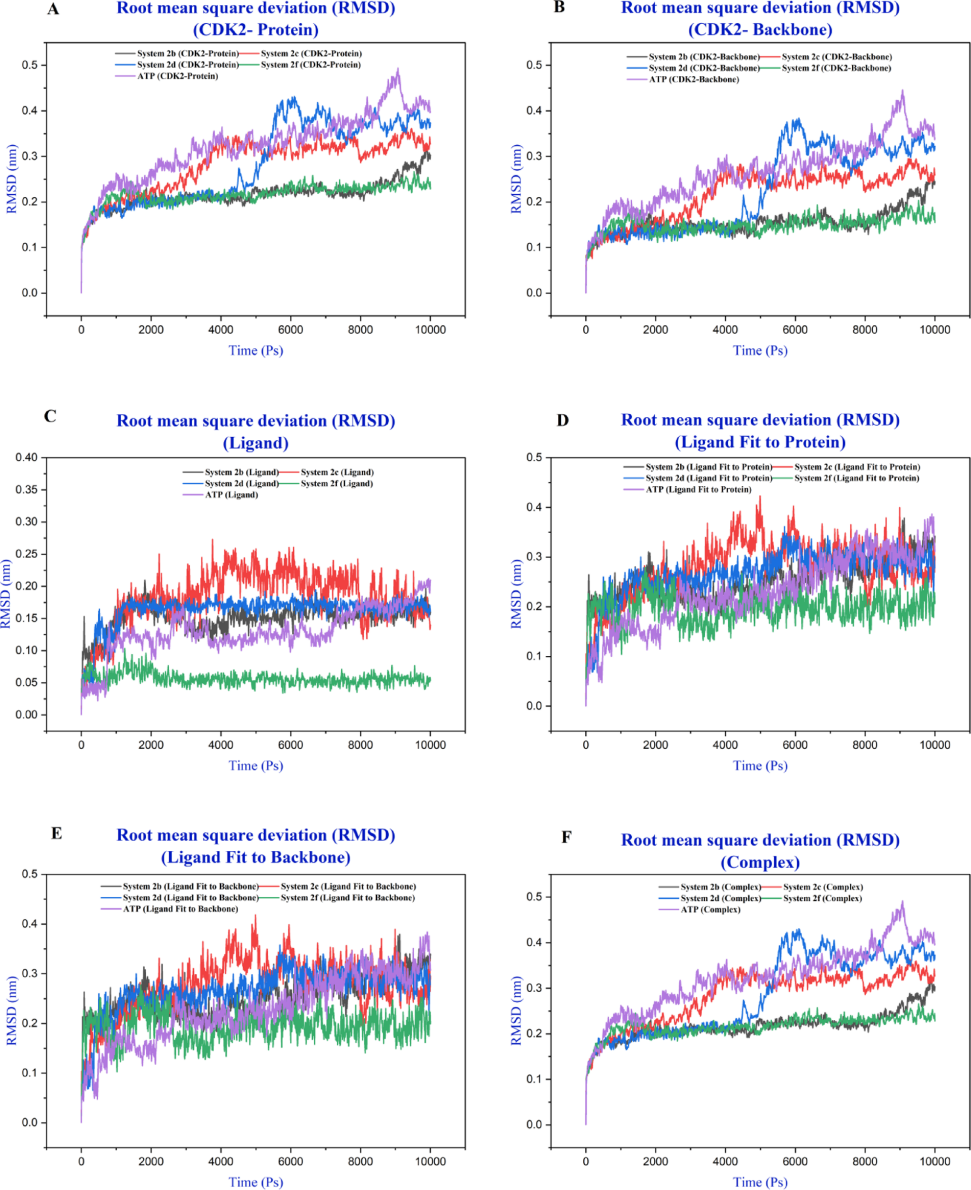
RMSD profiles of the CDK2 systems complexed with **2b**, **2c**, **2d**, **2f**, and **ATP** over 10 ns of molecular dynamics simulations: CDK2 protein (A), backbone (B), ligands (C), ligand fitted to the protein (D), ligand fitted to the backbone (E), and CDK2–ligand complex (F).

Root mean square deviation (RMSD) trajectories provide critical insights into the structural stability and conformational dynamics of CDK2 in complex with various ligands throughout the 10 ns MD simulations (Fig. 9 and S15- S20). Analysis of the CDK2-protein and CDK2-backbone reveals relatively stable RMSD values ranging from approximately 0.2 to 0.5 nm across most systems, indicating that the overall protein architecture remains well-preserved during the simulation period. The overlaid trajectories demonstrate convergence behavior, with most complexes reaching equilibrium within the first 2–3 ns, suggesting adequate sampling of the conformational space. Ligand RMSD profiles calculated after complex fitting demonstrated varying conformational stability. Compounds **2f** and **2h** exhibited the highest stability with RMSD values near 0.05 nm throughout the simulation, while compound **2e** showed the largest fluctuations, frequently exceeding 0.25 nm after 4 ns. When fitted to the CDK2-backbone and CDK2-protien, compounds **2f**,** 2h** and **ATP** remained highly stable (RMSD < 0.2 nm), whereas ligands **1** and **3** displayed pronounced deviations up to 1.0 nm, indicative of dissociation events. Overall system RMSD confirmed equilibration within 0.25–0.4 nm, with **2f** and **2i**-containing complexes exhibiting the lowest deviations. The RMSD analysis of the complete CDK2-ligand complexes integrates these observations, showing that overall complex stability is generally maintained across all systems, with mean RMSD values clustering between 0.3 and 0.5 nm. However, the varying degrees of fluctuation observed in individual trajectories (**Fig S7**) suggest that different ligands induce distinct dynamic behaviors in the CDK2 active site. Compounds exhibiting lower RMSD values with minimal fluctuations, such as **2f**, **2h**, and **ATP**, appear to form more rigid, stable complexes, while ligands with higher RMSD variability may engage in more dynamic binding interactions that could influence their pharmacological profiles. These RMSD patterns correlate with structural features of the ligands and provide a foundation for understanding their binding kinetics and potential efficacy as CDK2 inhibitors.

**Fig. 10:**
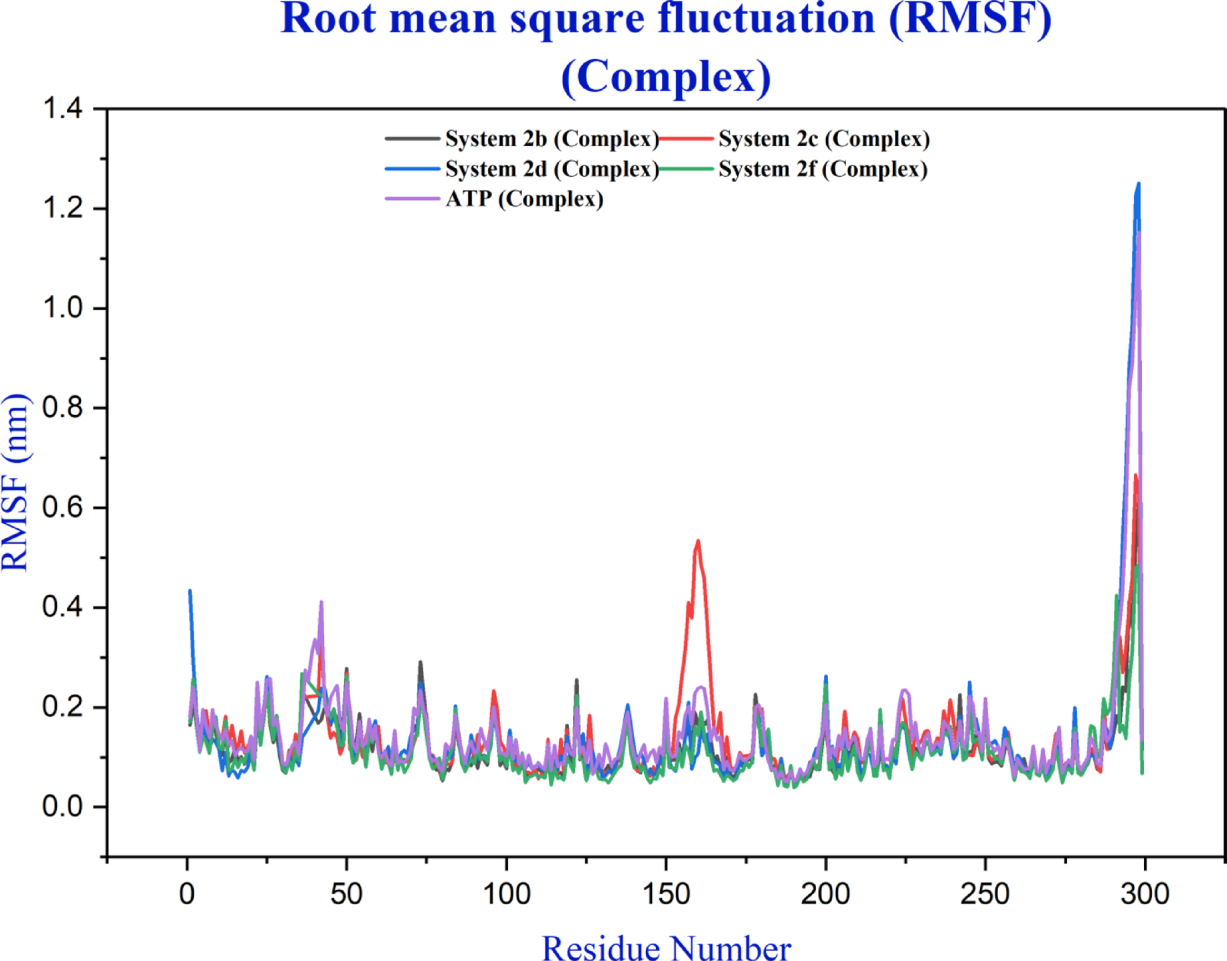
RMSF analysis of CDK2 in complex with ligands **2b**, **2c**, **2d**, **2f** and **ATP** highlights structural flexibility over the 10 ns molecular dynamics simulations.

Root mean square fluctuation (RMSF) analysis revealed consistent conformational dynamics across all systems, with backbone fluctuations predominantly ranging between 0.1 and 0.4 nm (Fig. 10 and S21). Three regions displayed elevated flexibility: The N-terminus (residues 0–50), mid-domain (residues 150–180), and C-terminus (residues 280–300), corresponding to characteristic kinase loop structures. Mean RMSF values clustered tightly between 0.11 and 0.15 nm with minimal standard deviations, indicating that ligand binding does not significantly perturb the intrinsic dynamic properties of CDK2.

**Fig. 11:**
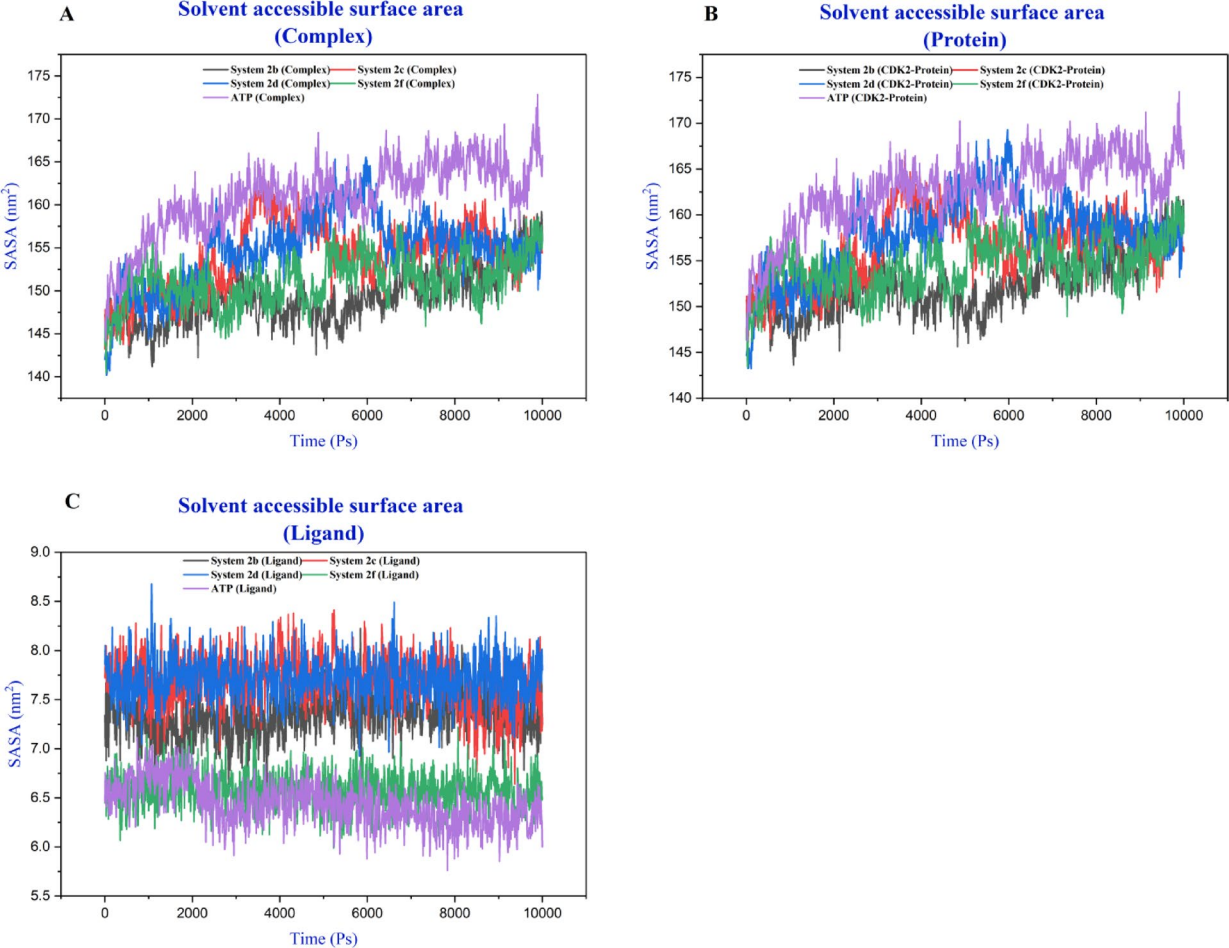
SASA profiles of CDK2- Ligand complex (**A**), CDK2 protein (**B**) and ligands (**2b**, **2c**, **2d**, **2f** and **ATP**) (**C**) over the 10 ns molecular dynamics simulations.

Solvent accessible surface area (SASA) analysis showed that complex and protein SASA values ranged between 145 and 165 nm² with considerable temporal fluctuation (Fig. 11 and S22- 24). The **ATP**-bound system consistently maintained higher SASA values (165–170 nm² after 2 ns), suggesting a more exposed conformation. Ligand SASA values (5.5–8.5 nm²) revealed that compounds **2c**, **2e** and **2d** exhibited consistently higher solvent exposure, while compound **1** showed deeper binding pocket burial.

**Fig. 12:**
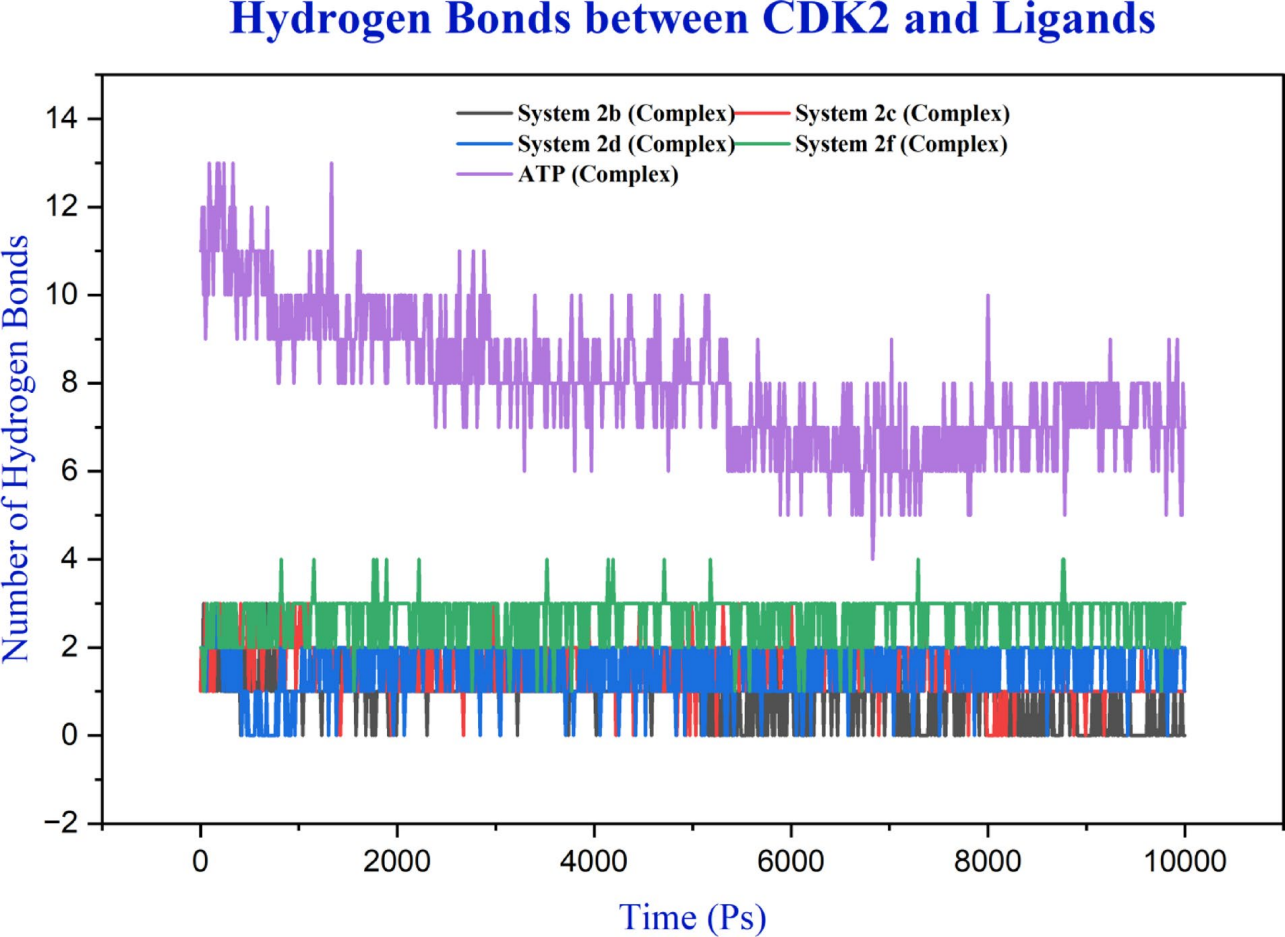
Time evolution of protein–ligand hydrogen bonds for systems **2b**, **2c**, **2d**, **2f** and **ATP** obtained from molecular dynamics simulations.

Hydrogen bond analysis revealed substantial variability in binding interactions (Fig. 12 and S25). The **ATP** reference system demonstrated the most extensive network, maintaining 5–13 bonds (mean: 7.95) with progressive decline from initial values of 10–13 bonds to a stable 6–9 bonds. Among synthetic ligands, compound **2f** exhibited the most robust interactions (mean: 2.70 bonds), maintaining stable 2–3 bonds throughout the trajectory. Compounds **2c** and **2d** showed moderate hydrogen bonding (means: 1.54 and 1.51 bonds), while compounds **2a** and **2b** averaged 0.83 bonds. Systems **1**, **2e**, **2h**, **2i**, and **3** demonstrated poor hydrogen bonding profiles (means: 0.27–0.93 bonds) with sporadic, transient contacts. Most systems achieved equilibration after 2–3 ns. The stark contrast between ATP’s extensive hydrogen bonding and the limited interactions of synthetic ligands highlights the challenge of achieving comparable binding affinity, suggesting that compounds **2c**, **2d**, and particularly **2f** represent the most promising scaffolds for further optimization.

### MM-PBSA binding free energy analysis of CDK2–ligand complexes

**Fig. 13:**
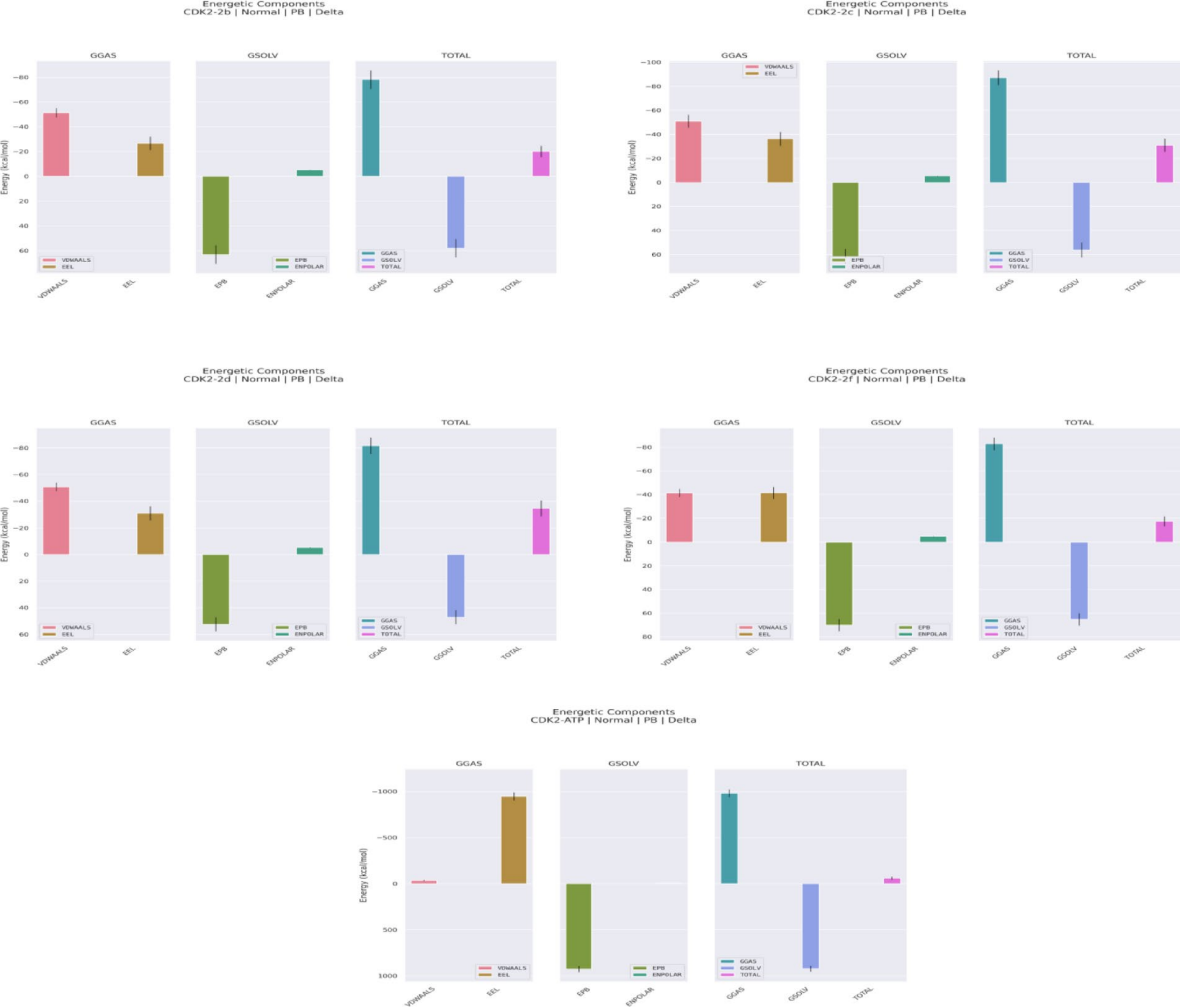
MM-PBSA energy decomposition profiles for CDK2–ligand complexes. Gas-phase (ΔG_GAS), solvation (ΔG_SOLV), and total binding free energies (ΔG_TOTAL) are shown for **2b**, **2c**, **2d**, **2f** and ATP, highlighting key energetic differences underlying their binding affinities.

The binding affinities of CDK2 toward the synthesized compounds (**1**, **2a–2i**, and **3**) and ATP were evaluated using MM-PBSA analyses derived from molecular dynamics simulations (Table 7; Fig. 13 and S26). The synthetic ligands exhibited ΔG_bind values ranging from − 8.61 to − 34.50 kcal/mol, reflecting the strong influence of structural modifications on binding thermodynamics. Compound **2d** showed the highest affinity (ΔG_bind = − 34.50 ± 0.42 kcal/mol), representing a three-fold improvement over compound **1** (–11.43 ± 0.34 kcal/mol) and surpassing structurally related analogues **2a**, **2b**, and **2c**. Van der Waals forces (ΔE_VDWAALS) were the dominant contributors to binding across all ligands, with compounds **2a–2d** displaying the strongest interactions (–50.74 to − 51.77 kcal/mol). Electrostatic energies (ΔE_EEL) varied substantially, with **2f** (–41.49 kcal/mol), **2c** (–36.21 kcal/mol), and **2d** (–30.91 kcal/mol) showing the largest contributions, whereas compounds **2h** and **3** exhibited weak electrostatic stabilization. The resulting gas-phase energies favored binding for all compounds, particularly **2c** (ΔG_GAS = − 87.15 kcal/mol), followed by **2f**, **2d**, and **2a**. However, these enthalpic gains were mitigated by polar solvation penalties (ΔE_PB = 43.91–70.14 kcal/mol), with **2d** benefiting from the lowest penalty among high-affinity ligands, enabling an optimal balance between gas-phase interactions and solvation effects. In contrast, compound **2f**, despite strong electrostatic and gas-phase interactions, exhibited reduced affinity (ΔG_bind = − 17.51 kcal/mol) due to the highest desolvation cost (ΔE_PB = 70.14 kcal/mol). Compounds **2a**, **2b**, and **2i** showed moderate binding energies (–20.02 to − 23.46 kcal/mol), while compounds **2e**, **2g**, **2h**, and **3** were the weakest binders (–8.61 to − 19.88 kcal/mol), primarily due to insufficient van der Waals and/or electrostatic stabilization. ATP displayed a distinct energetic signature dominated by its highly charged triphosphate group, yielding exceptionally strong electrostatics (ΔE_EEL = − 947.61 kcal/mol) and a gas-phase energy of − 980.68 kcal/mol. However, the extremely high polar solvation penalty (ΔE_PB = 925.44 kcal/mol) resulted in a final ΔG_bind of − 59.68 ± 1.17 kcal/mol, exceeding all synthetic ligands as expected for the endogenous substrate. Overall, MM-PBSA and MD analyses highlight **2c and 2d** as the most promising scaffolds, with **2d** achieving optimal affinity through balanced interactions and favorable desolvation, and **2c** through maximized gas-phase stabilization. Compounds **2a**–**2f** and **2i** constitute the most compelling candidates for further lead optimization, whereas compounds **1**, **2g**, **2h**, and **3** exhibit comparatively weak binding profiles.

**Table 7:** MM-PBSA binding free energy analysis of CDK2-ligand (**1**, **2a-2i**, and **3**) and **ATP** Complexes, all energy values are in kcal/mol. Values represent average binding energies from molecular dynamics simulations.

Compound	ΔVDWAALS	ΔE_EEL	ΔG_GAS	ΔE_PB	ΔENPOLAR	ΔG_SOLV	ΔG_bind	SEM
1	−37.79	−16.98	−54.77	47.58	−4.23	43.35	**−11.43**	± 0.34
2a	−51.77	−29.93	−81.70	66.48	−5.09	61.39	**−20.30**	± 0.31
2b	−51.36	−26.68	−78.04	63.17	−5.15	58.02	**−20.02**	± 0.32
2c	−50.95	−36.21	−87.15	61.56	−5.34	56.22	**−30.94**	± 0.39
2d★	**−50.74**	**−30.91**	**−81.65**	**52.40**	**−5.26**	**47.15**	**−34.50**	**± 0.42**
2e	−43.58	−16.77	−60.35	45.05	−4.58	40.47	**−19.88**	± 0.25
2f	−41.31	−41.49	−82.80	70.14	−4.86	65.29	**−17.51**	± 0.28
2 g	−49.20	−28.96	−78.17	69.66	−5.02	64.64	**−13.52**	± 0.38
2 h	−43.20	−11.14	−54.34	50.21	−4.47	45.73	**−8.61**	± 0.37
2i	−48.35	−17.28	−65.63	47.11	−4.94	42.17	**−23.46**	± 0.36
3	−41.55	−10.14	−51.69	43.91	−4.25	39.66	**−12.03**	± 0.30
ATP	**−33.07**	**−947.61**	**−980.68**	**925.44**	**−4.43**	**921.00**	**−59.68**	**± 1.17**

ΔVDWAALS: Van der Waals contribution to binding. ΔE_EEL: Electrostatic energy contribution. ΔG_GAS: Total gas-phase free energy. ΔE_PB: Polar solvation energy (Poisson-Boltzmann). ΔE_NPOLAR: Non-polar solvation energy. ΔG_SOLV: Total solvation free energy. Δ_G_bind_: Total binding free energy. SEM: Standard error of the mean.

★ indicates the compound with the most favorable binding energy. More negative ΔG_bind_ values indicate stronger binding affinity.

## Conclusion

This comprehensive computational study successfully identified novel 2-thiohydantoin derivatives as promising CDK2 inhibitors through an integrated approach combining DFT, molecular docking, molecular dynamics simulations, and MM-PBSA calculations. The systematic investigation of compounds **1**, **2a-i**, and **3** revealed critical structure-activity relationships governing CDK2 binding. DFT calculations demonstrated that compounds **2b-e** with electron-withdrawing substituents exhibited narrowed HOMO-LUMO gaps (3.02–3.26 eV in DMSO) and enhanced electrophilicity indices (> 3.20 eV), indicating superior reactivity. Fukui function and QTAIM analyses identified key electrophilic centers (C2, O12, C14) and nucleophilic sites essential for molecular recognition. Molecular electrostatic potential mapping revealed charge distributions facilitating protein interactions, while non-covalent interaction analysis demonstrated intramolecular stabilization preorganizing molecules into bioactive conformations. Molecular docking identified compounds **2c**, **2d**, and **2b** as top inhibitors with binding affinities of −9.312, −9.303, and − 9.269 kcal/mol, respectively, surpassing ATP (−8.460 kcal/mol). These compounds formed critical hydrogen bonds with Lys33 and Thr14 and extensive hydrophobic networks with key active site residues. The 10 ns MD simulations validated complex stability, with CDK2 maintaining structural integrity (Rg: 1.96–2.10 nm). Compound **2f** exhibited exceptional stability (RMSD ~ 0.05 nm) and robust hydrogen bonding (mean: 2.70 bonds), while **2c** and **2d** maintained consistent interactions. MM-PBSA calculations identified compound **2d** as the most favorable inhibitor (ΔG_bind = −34.50 ± 0.42 kcal/mol), three-fold better than parent compound **1** (−11.43 ± 0.34 kcal/mol), through optimal van der Waals interactions (−50.74 kcal/mol), electrostatic stabilization (−30.91 kcal/mol), and minimal desolvation penalty (52.40 kcal/mol). Compound **2c** achieved comparable potency (−30.94 ± 0.39 kcal/mol) via maximized gas-phase interactions. Structure-activity analysis revealed that halogen substituents enhanced binding through favorable inductive effects and desolvation profiles, while carboxylic acid and ester functionalities improved conformational stability despite increased desolvation costs. Compounds **2b**, **2c**, **2d**, and **2f** emerge as lead candidates warranting experimental validation. Compound **2d** represents the premier candidate with the highest binding affinity and acceptable stability, **2c** offers comparable thermodynamics with the strongest gas-phase interactions, **2b** demonstrates strong binding affinity, and **2f** demonstrates exceptional conformational stability for prolonged target engagement. These findings establish a robust framework for developing next-generation CDK2 inhibitors, addressing selectivity challenges and advancing targeted anticancer therapies. Although these computational findings provide a strong foundation, experimental validation is required. Future work should include synthesis of the lead compounds followed by in vitro kinase inhibition and cell-based antiproliferative assays. Selectivity profiling against other CDK isoforms, ADMET optimization, and resistance modeling through mutation analysis will be essential to guide further optimization. Collectively, this study establishes a robust computational framework for the rational design of next-generation CDK2 inhibitors and supports the continued development of targeted anticancer therapies.

## Supplementary Information

Below is the link to the electronic supplementary material.

Supplementary Material 1

## Data Availability

The data that support the findings of this study are available from the corresponding author upon reasonable request.
