# Supplementary material for: Integrated DFT, molecular docking, and molecular dynamics investigation of some novel 2-thiohydantoin analogues as potent CDK2 inhibitors for anticancer therapy
Source: Sci Rep. 2026 Mar 26;16:10985. doi: 10.1038/s41598-026-42330-4 (PMC13044310; doi:10.1038/s41598-026-42330-4)
Supplement: Supplementary file 1 — Supplementary Material 1 [file 41598_2026_42330_MOESM1_ESM.docx]

**Integrated DFT, Molecular Docking, and Molecular Dynamics Investigation of some Novel 2-Thiohydantoin Analogues as Potent CDK2 Inhibitors for Anticancer Therapy**

**
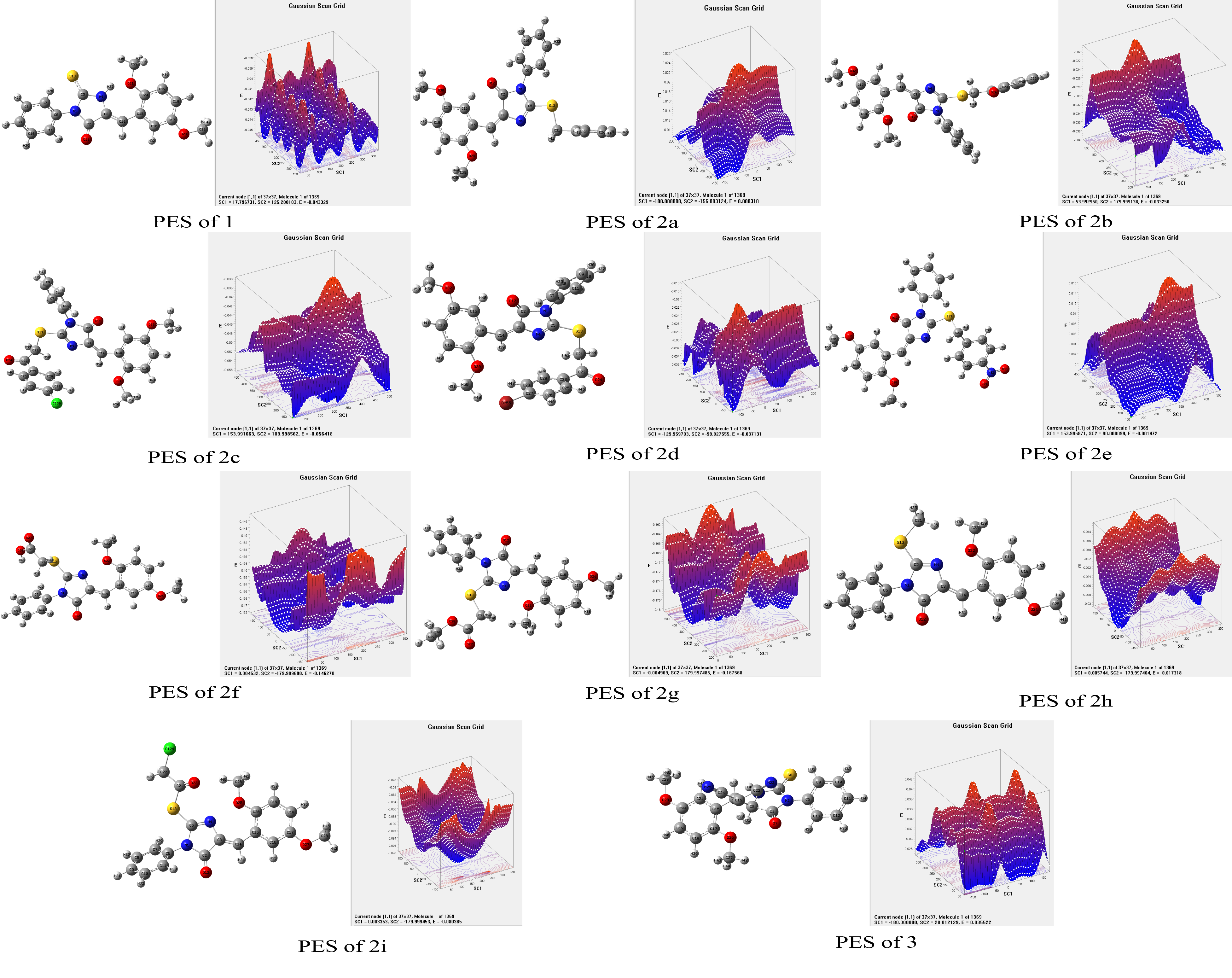
**

**Fig. S1.** Potential energy surface (PES) scan of compounds **1**, **2a**–**2i**, and **3**.


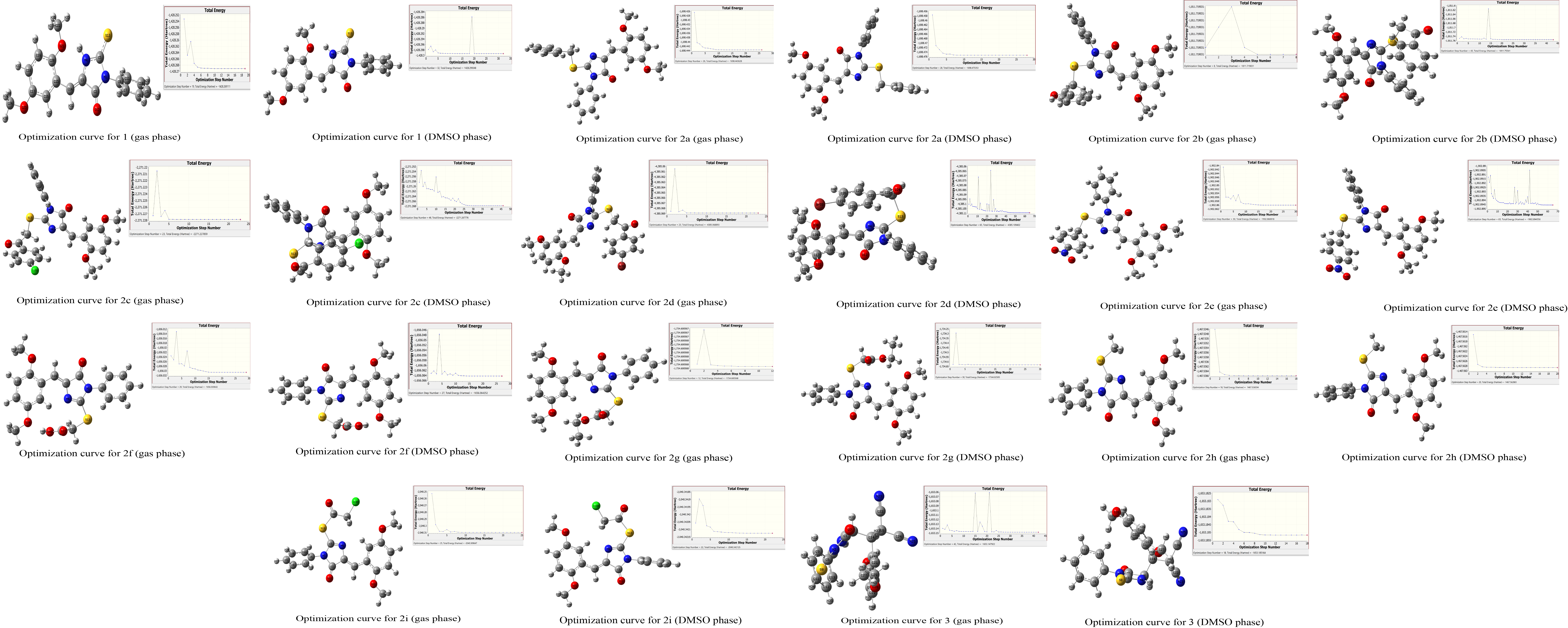


**Fig. S2.** Optimized curve of compounds **1**, **2a**–**2i**, and **3** in the gas and DMSO phase.


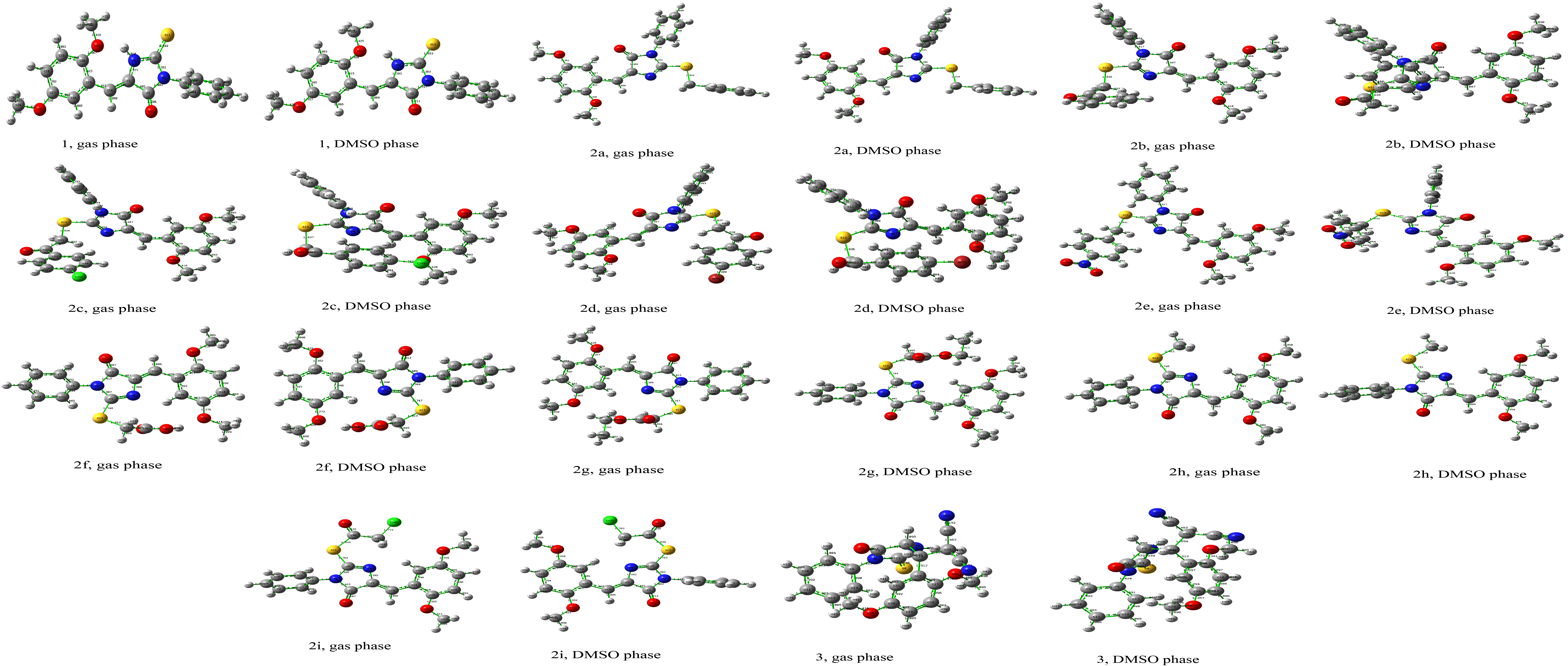


**Fig. S3.** Optimized structures of compounds **1**, **2a**–**2i**, and **3** calculated in gas and DMSO phase.


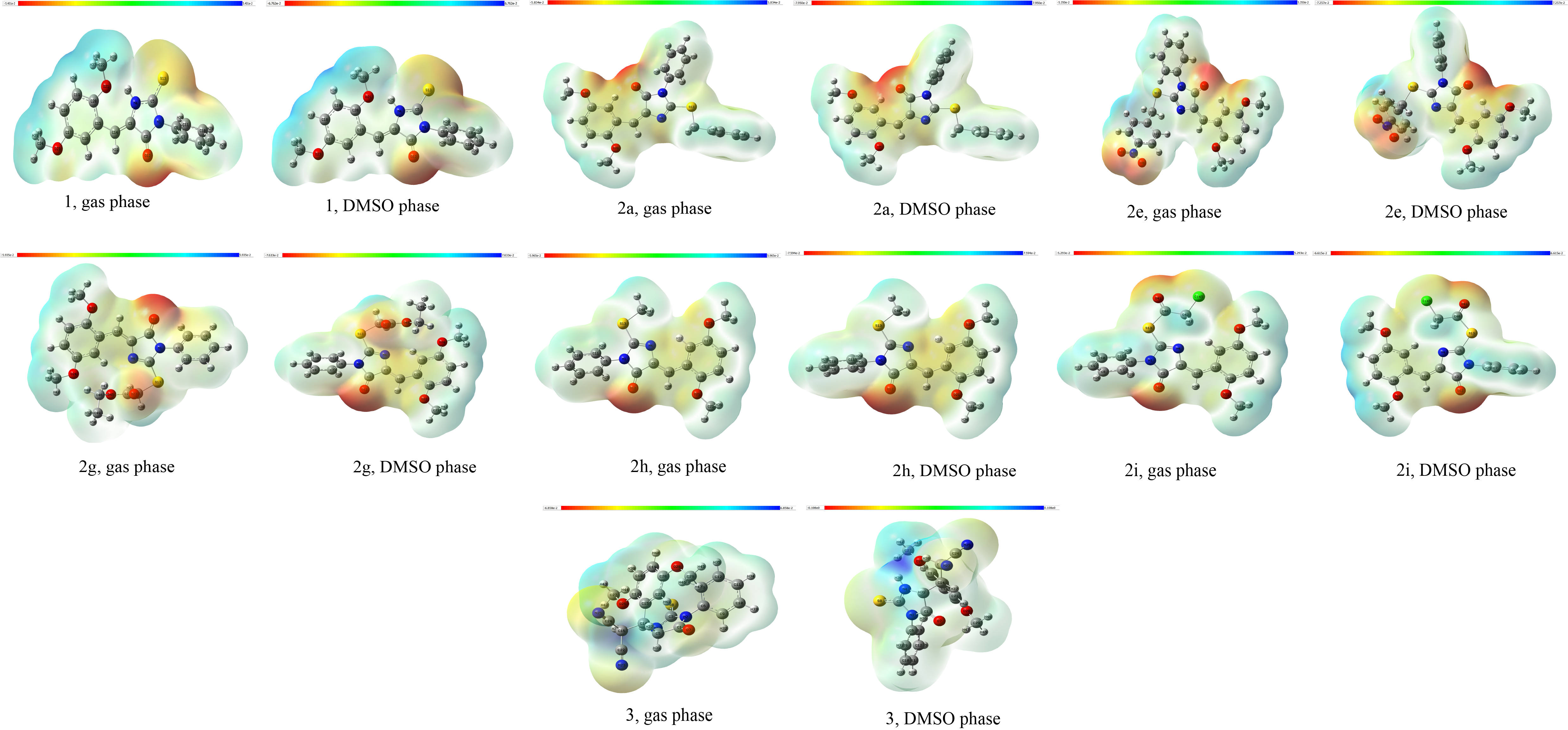


**Fig. S4.** Molecular electrostatic potential (MESP) surfaces of compounds **1, 2a, 2e**, **2g-2i** and **3** in gas and DMSO phase.


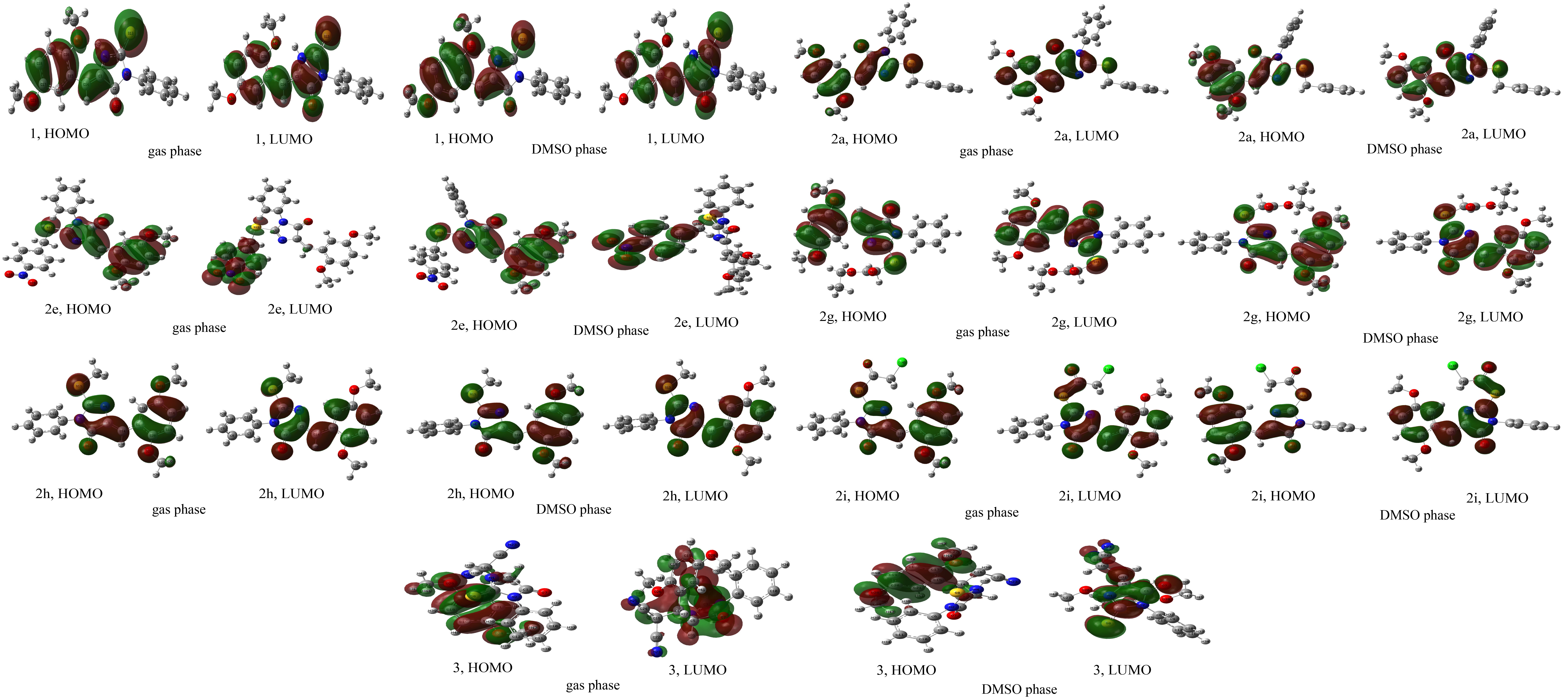


**Fig. S5.** Frontier molecular orbitals (HOMO and LUMO) of compounds **1, 2a, 2e**, **2g-2i** and **3** calculated in gas and DMSO phase.


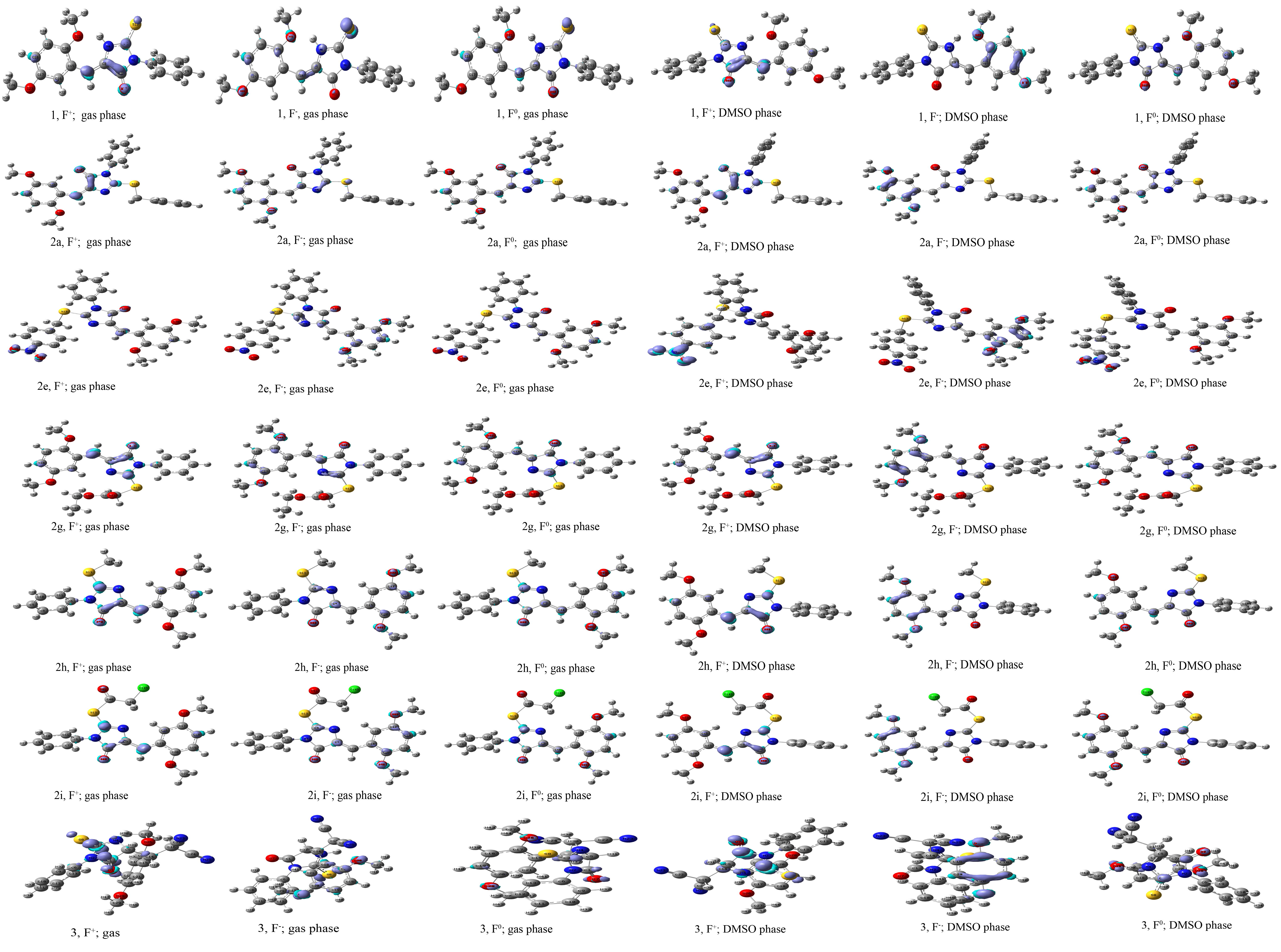


**Fig. S6.** Fukui functions and local reactivity indices for compounds **1, 2a, 2e**, **2g-2i** and **3** in gas and DMSO phase. Site-specific reactivity descriptors for electrophilic (f⁻), nucleophilic (f⁺), and radical (f⁰) attacks derived from Hirshfeld charges.


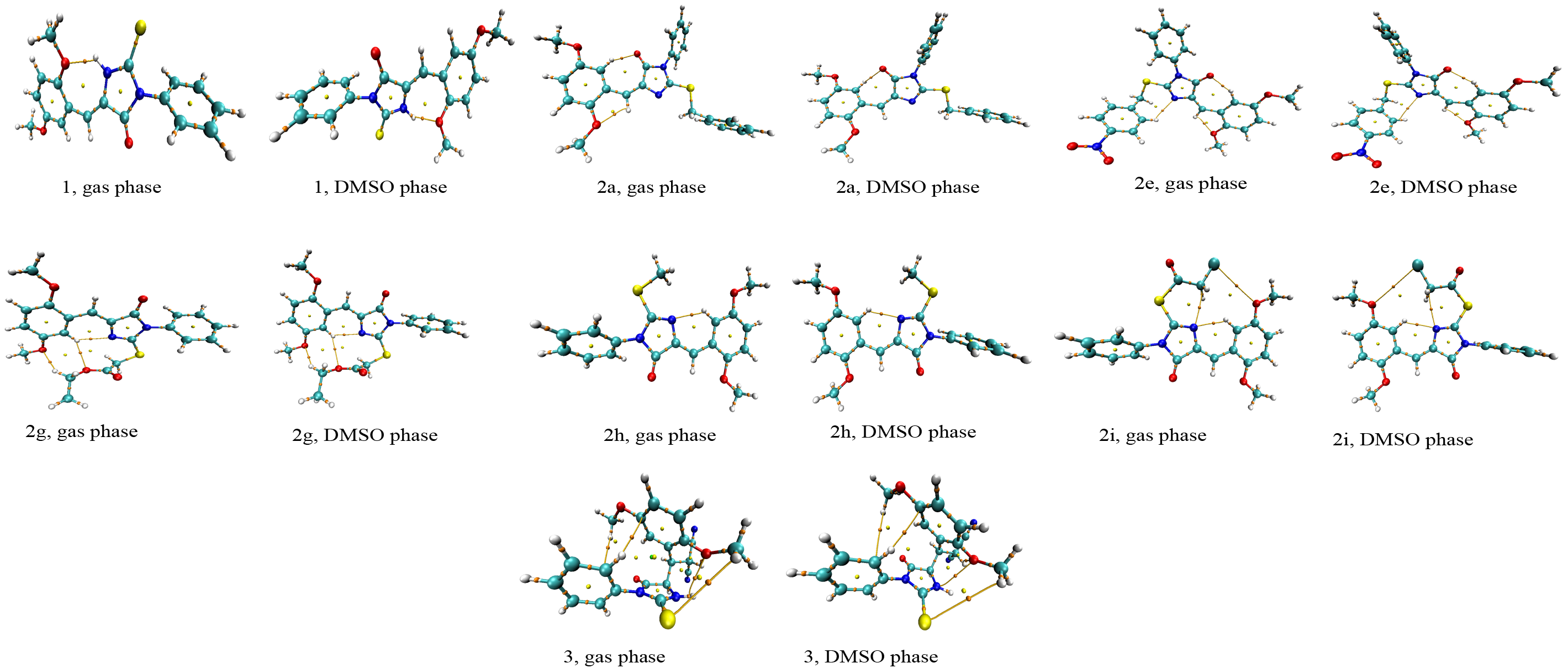


**Fig. S7.** Molecular graphs illustrating the nuclear critical points (NCPs), bond critical points (BCPs), and associated bond paths for compounds **1, 2a, 2e**, **2g-2i** and **3** (Gas and DMSO phase).


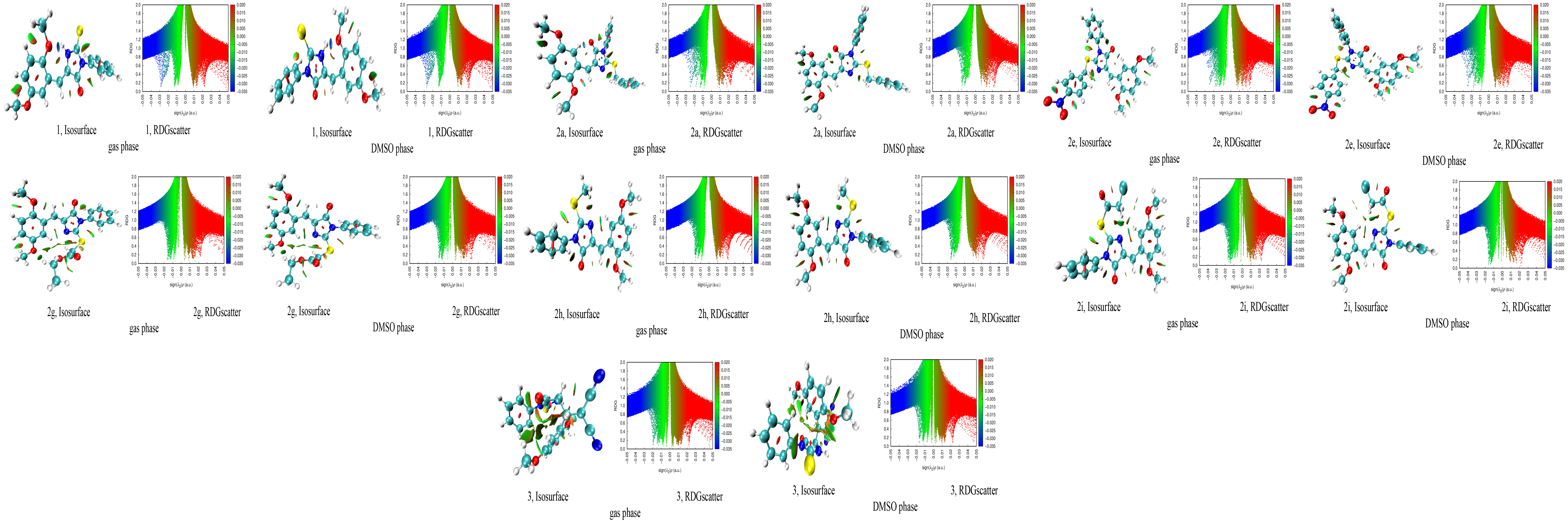


**Fig. S8.** NCI isosurfaces and RDG scatter plots for compounds **1, 2a, 2e**, **2g-2i** and **3** in the gas and DMSO phase.


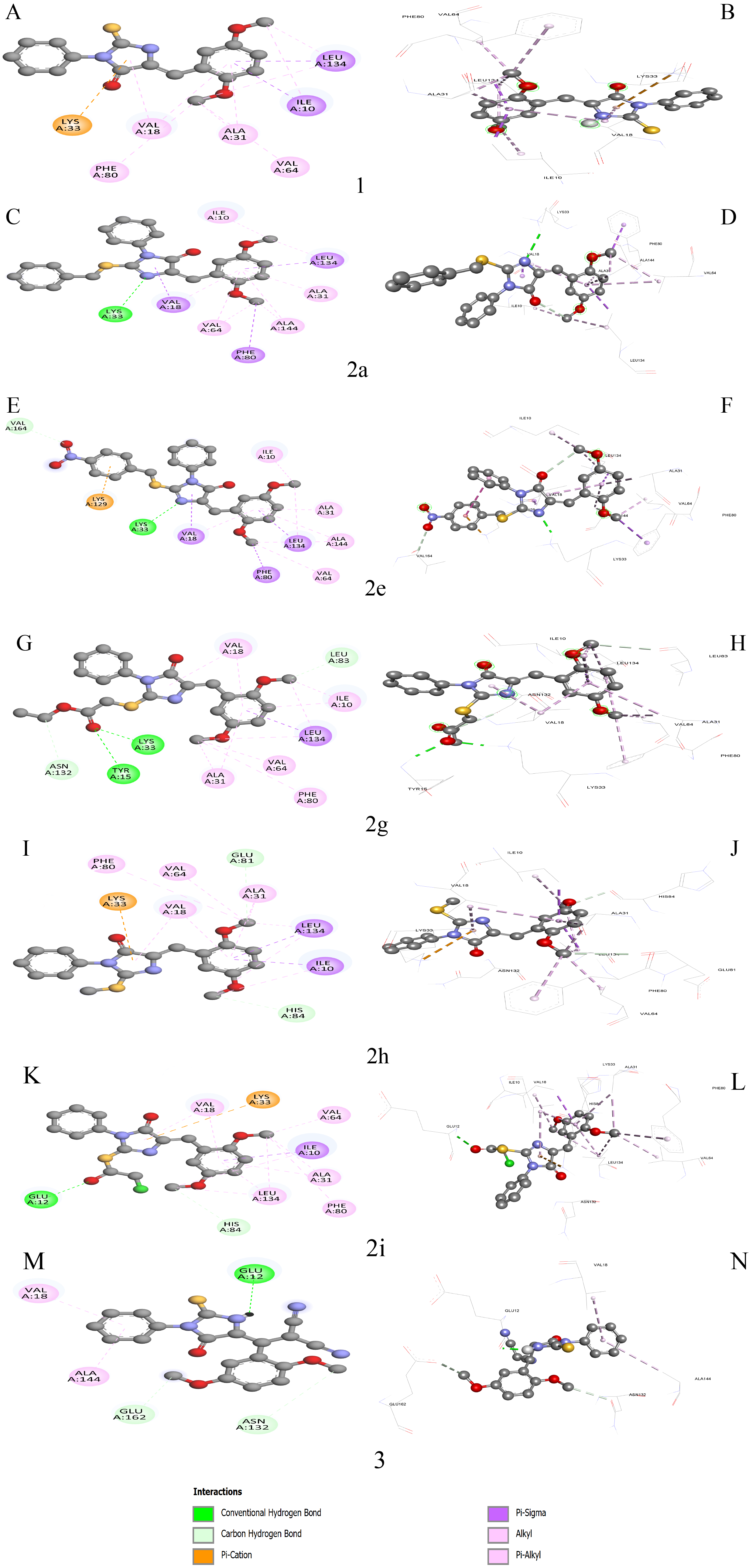


**Fig. S9.** 2D and 3D binding interaction diagrams of compounds **1, 2a, 2e**, **2g-2i** and **3** with CDK2 (PDB: 1HCK).


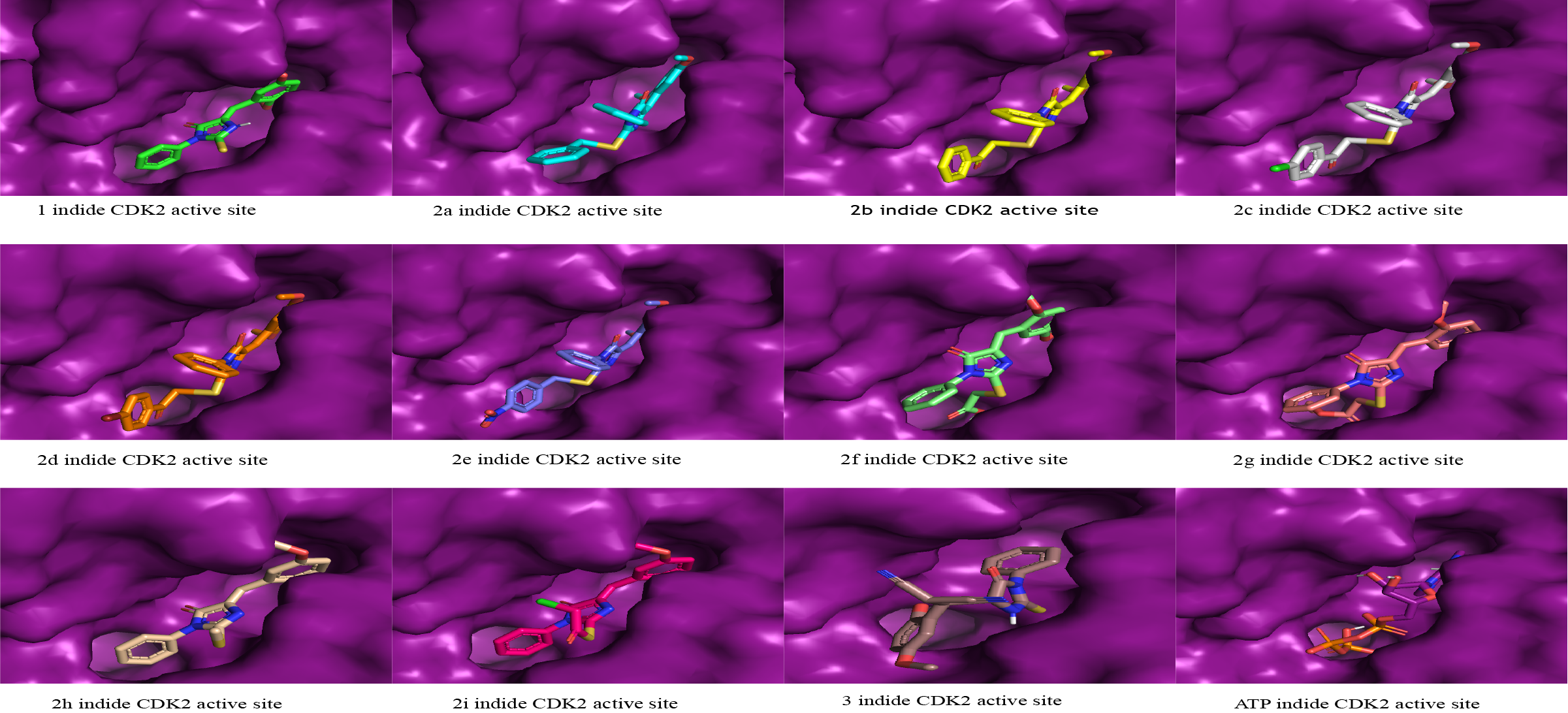


**Fig. S10.** Molecular docking poses of compounds **1**, **2a-h**, **3**, and **ATP** (colored sticks) within the CDK2 binding pocket (magenta surface), showing distinct binding orientations for each ligand.


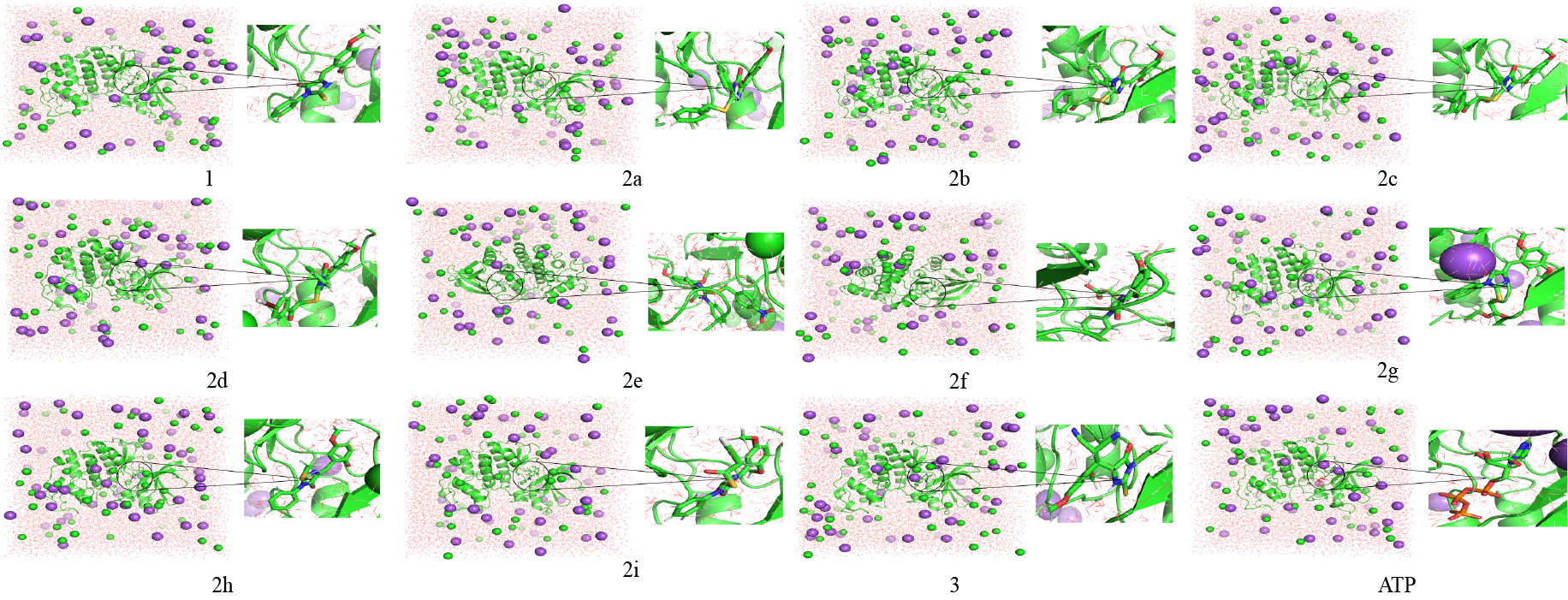


**Fig. S11.** MD snapshots of CDK2 with compounds **1, 2a–2i**, **3**, and **ATP**, showing ligand binding poses, surrounding water (pink) and ions (purple/green).

**
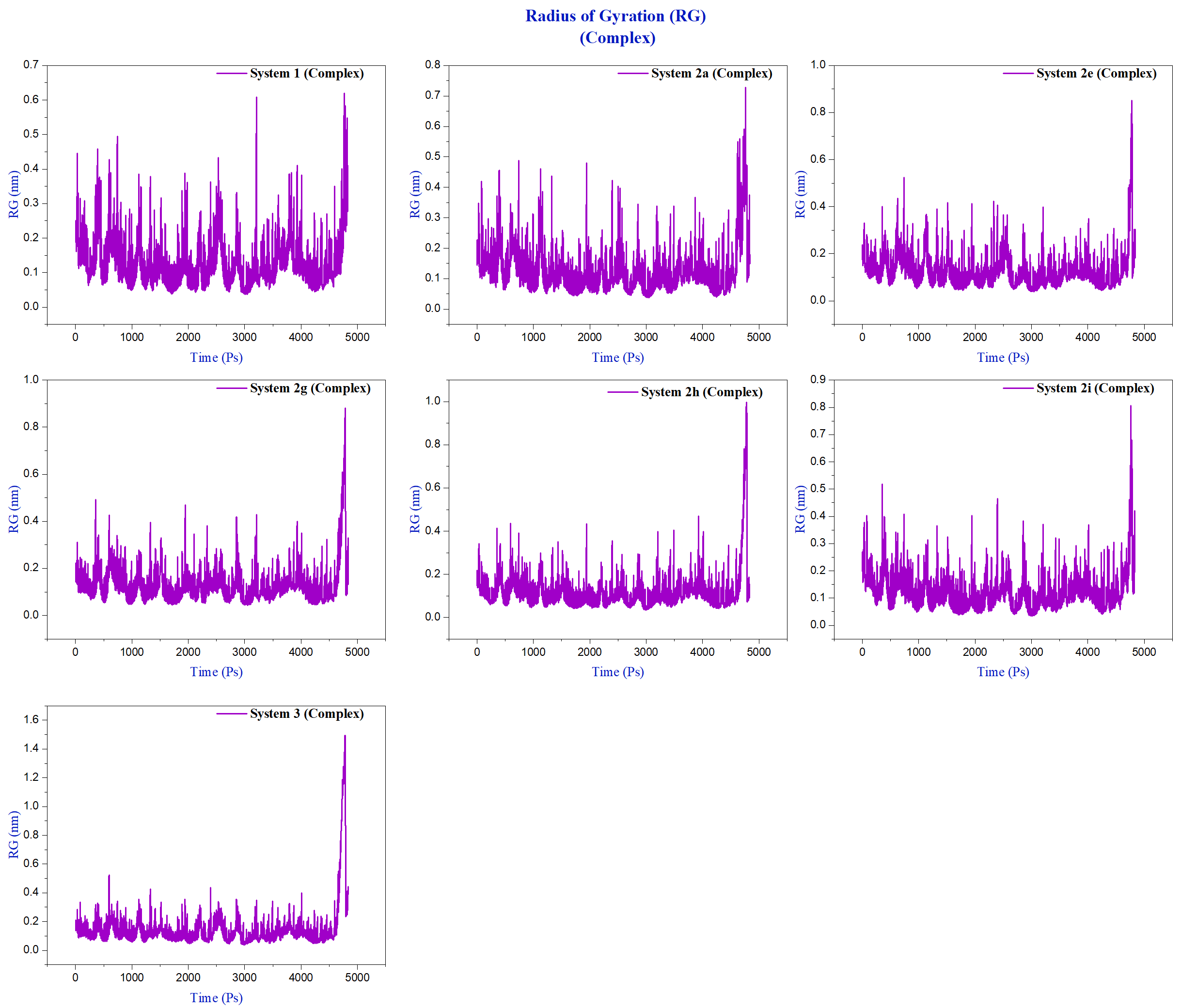
**

**Fig. S12.** RG profiles of CDK2 complexes with compounds **1**, **2a**, **2e**, **2g**-**2i** and **3** over 10 ns of MD simulations, showing the structural compactness and stability of each protein–ligand system.


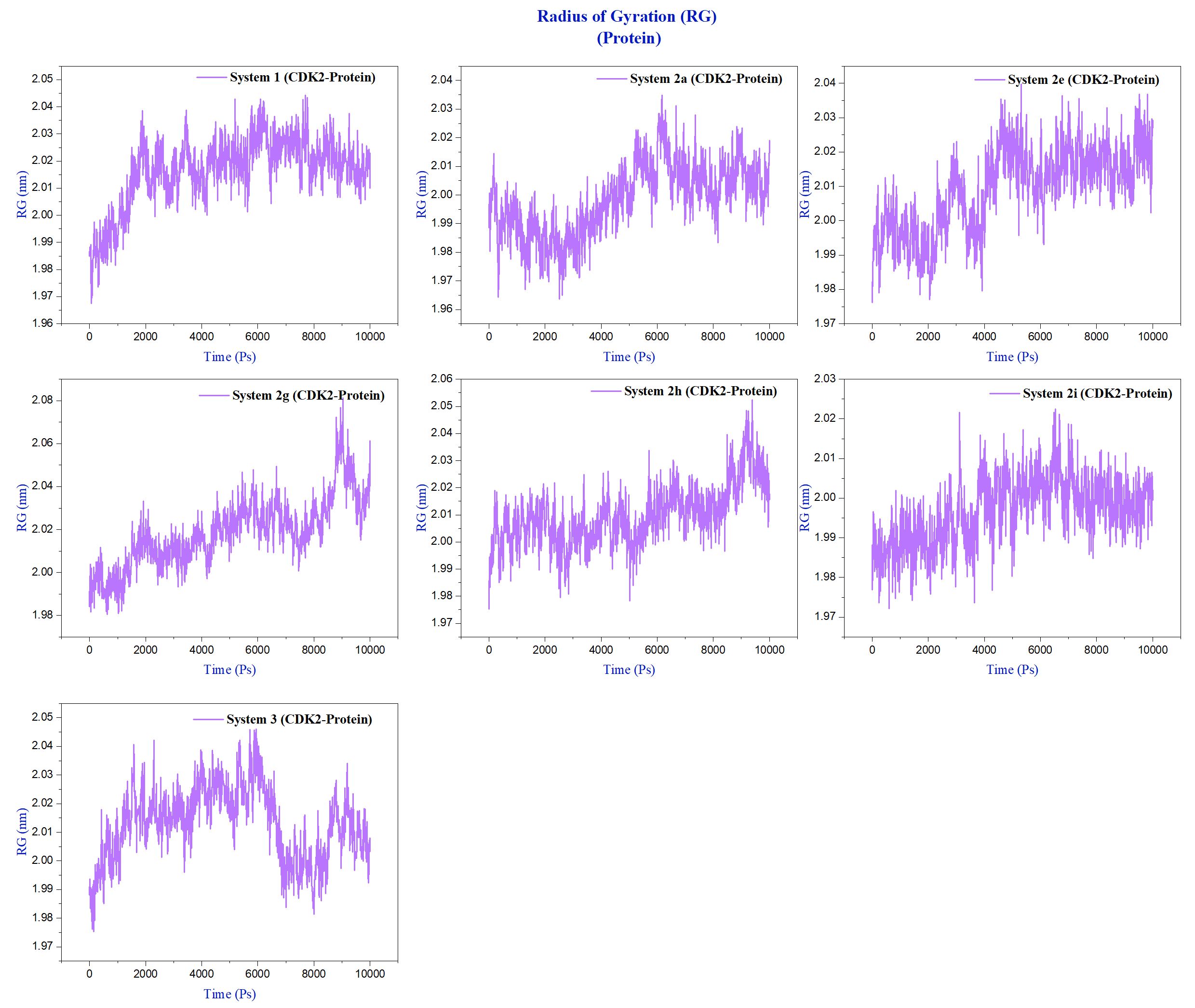


**Fig. S13.** RG plots of CDK2 protein over 10 ns MD simulations for systems **1, 2a, 2e**, **2g-2i** and **3**, showing protein compactness and structural stability for each system.


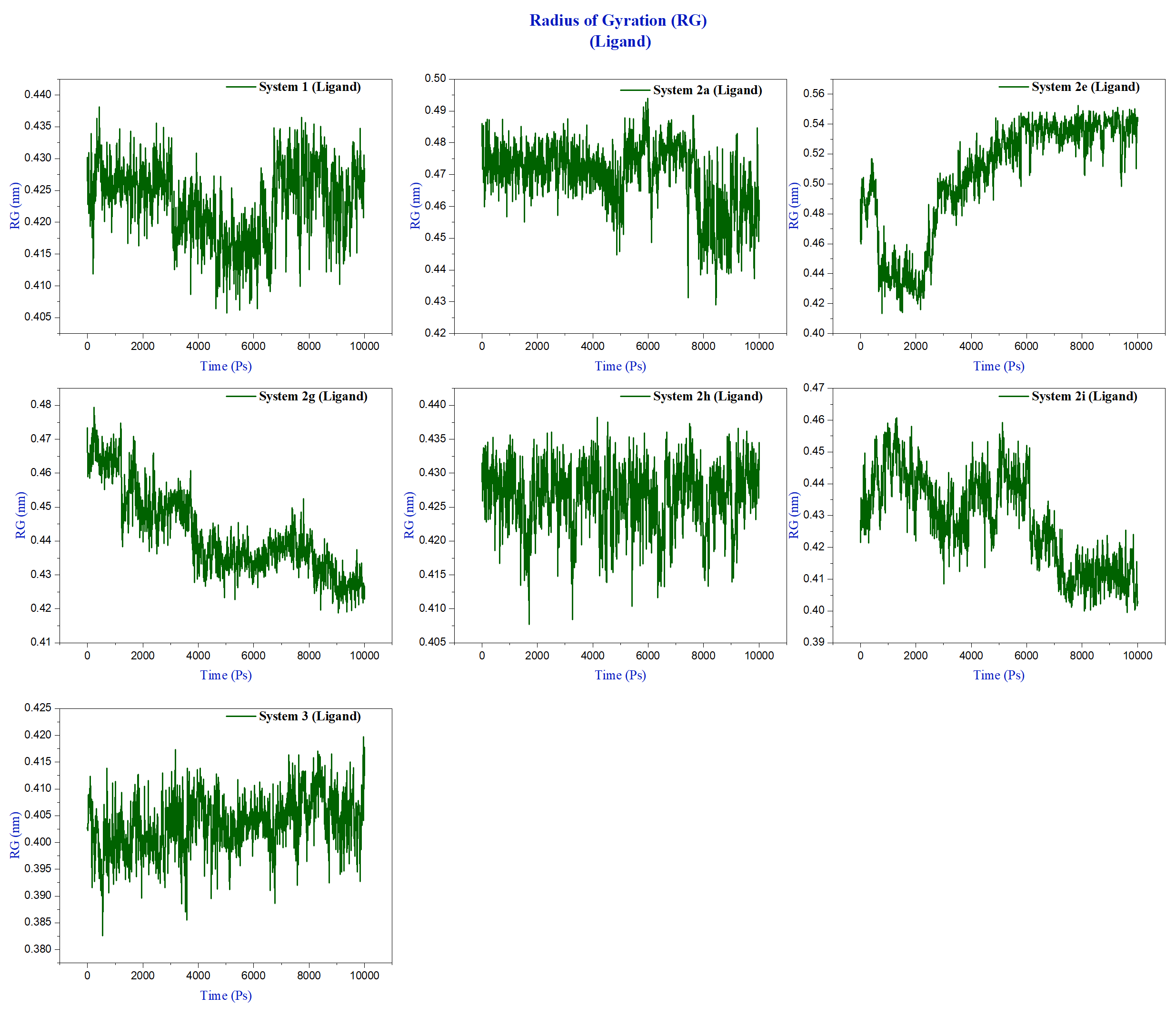


**Fig. S14.** RG profiles of the ligands in systems **1, 2a, 2e, 2g–2i, and 3**, showing stable ligand compactness throughout the 10 ns molecular dynamics simulations.


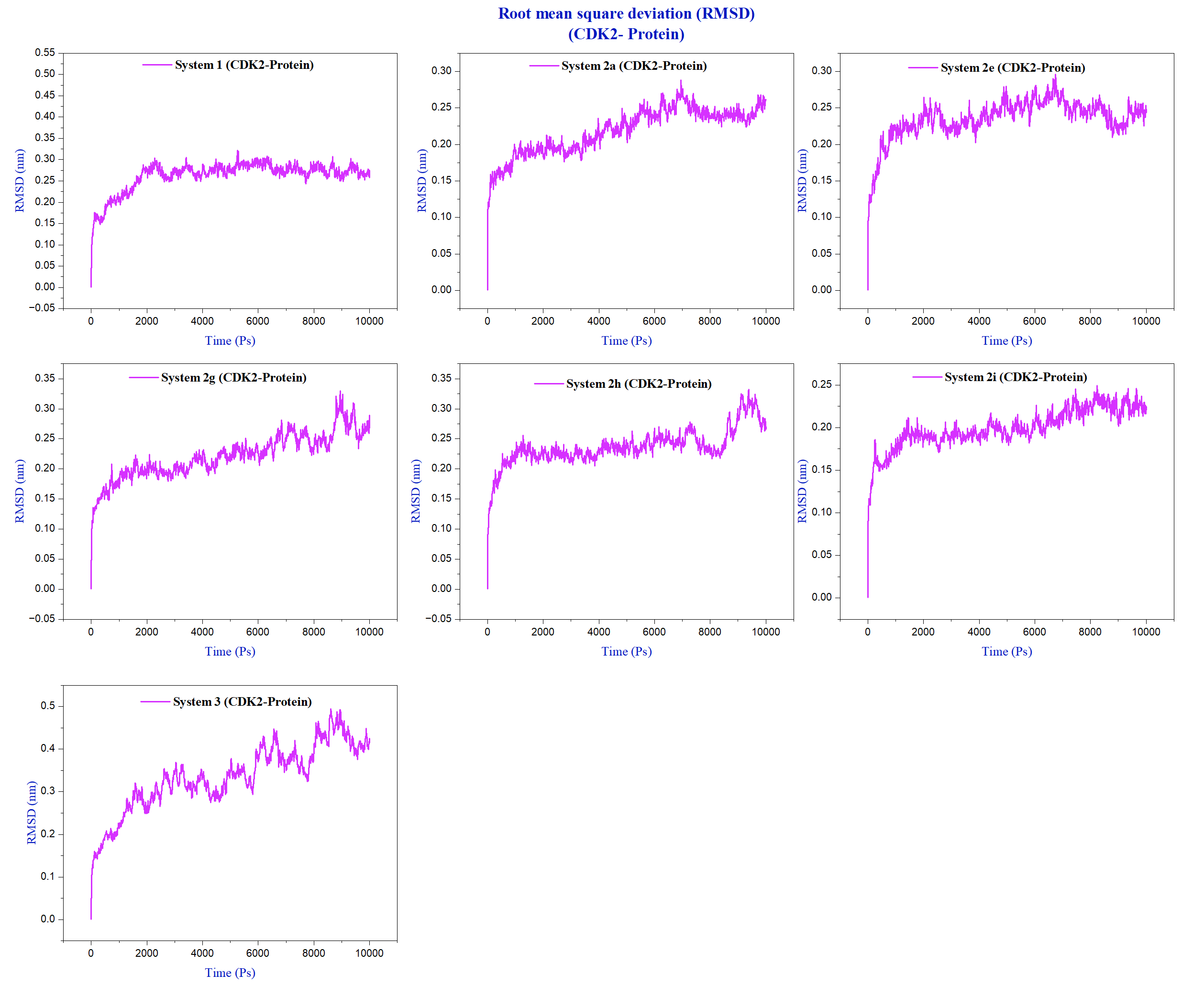


**Fig. S15.** RMSD trajectories of the CDK2 protein over 10 ns MD simulations for systems **1, 2a, 2e**, **2g-2i** and **3**.


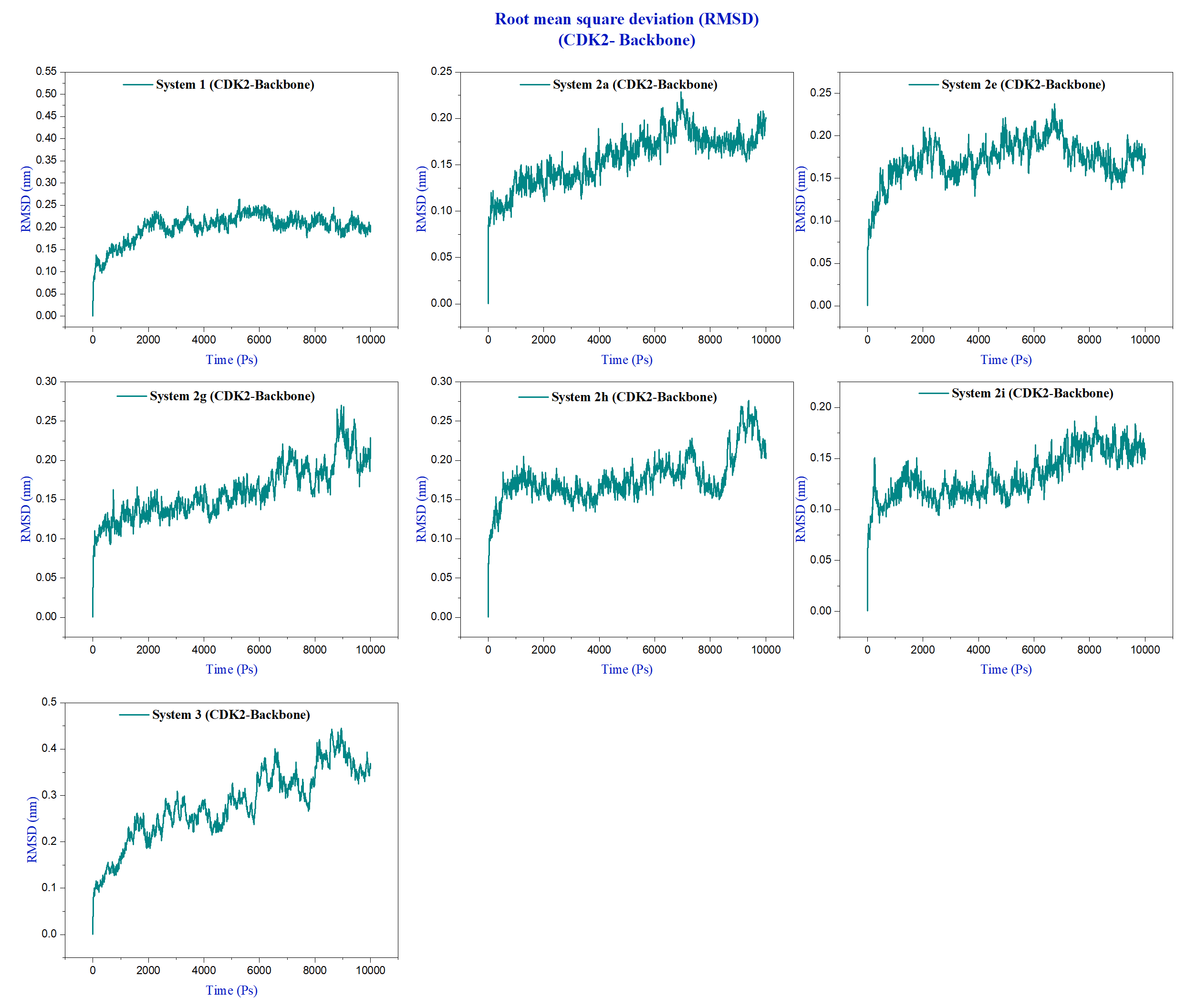


**Fig. S16.** RMSD trajectories of the CDK2 backbone over 10 ns MD simulations for systems **1, 2a, 2e**, **2g-2i** and **3**.


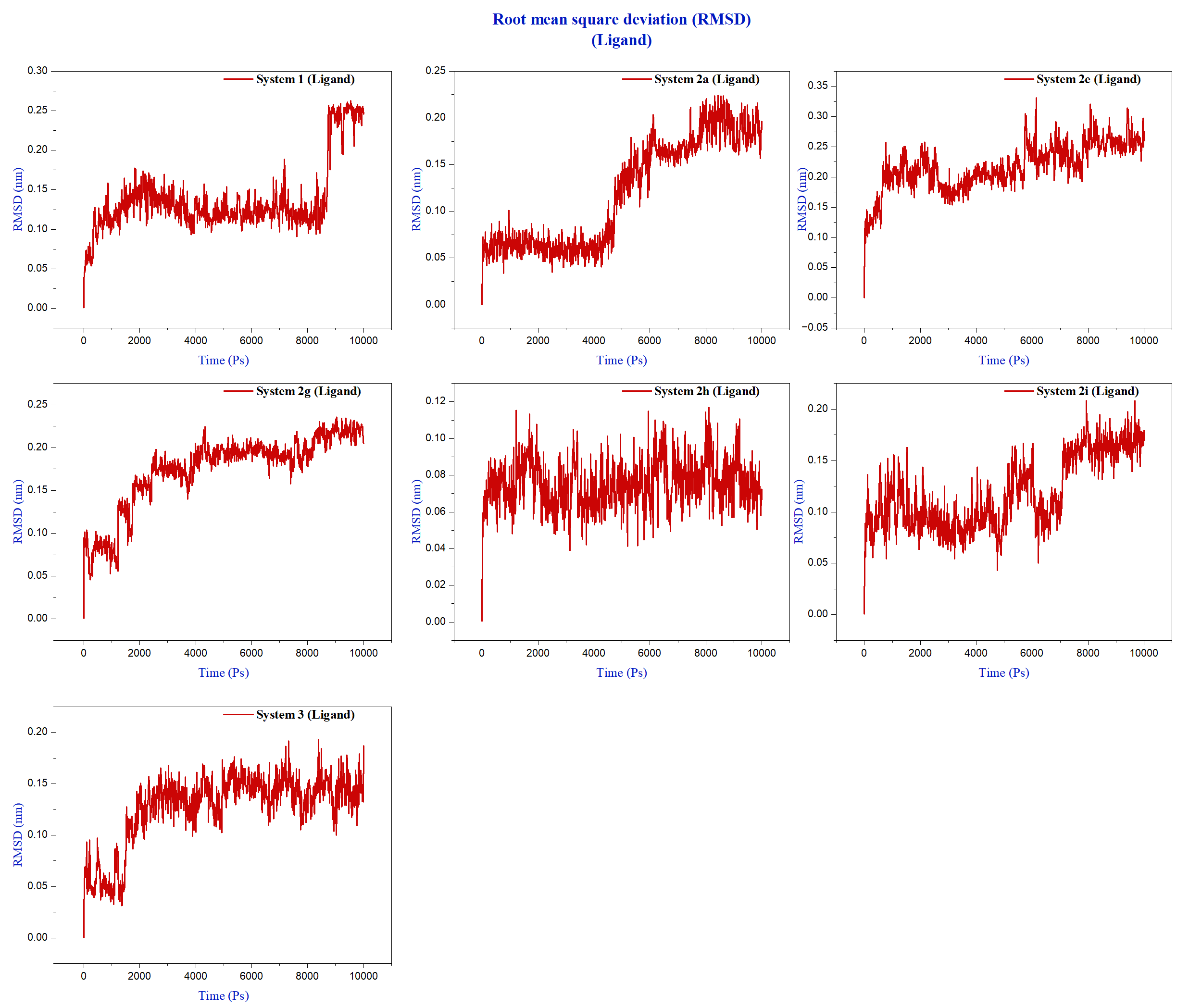


**Fig. S17.** RMSD of ligands (**1, 2a, 2e**, **2g-2i** and **3**) over 10 ns MD simulations.


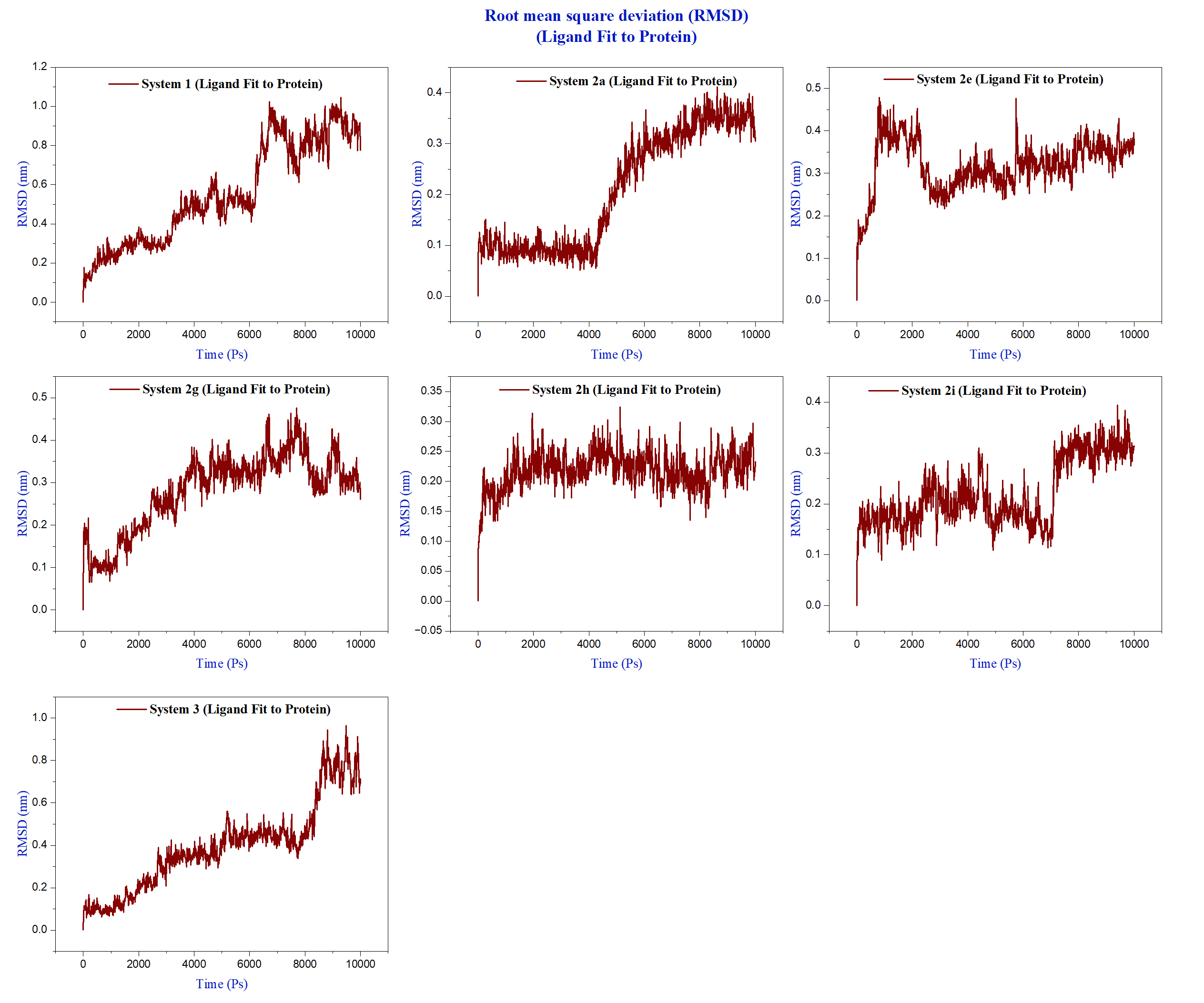


**Fig. S18.** RMSD trajectories of ligands fitted to the protein over 10 ns MD simulations for systems **1, 2a, 2e**, **2g-2i** and **3**.


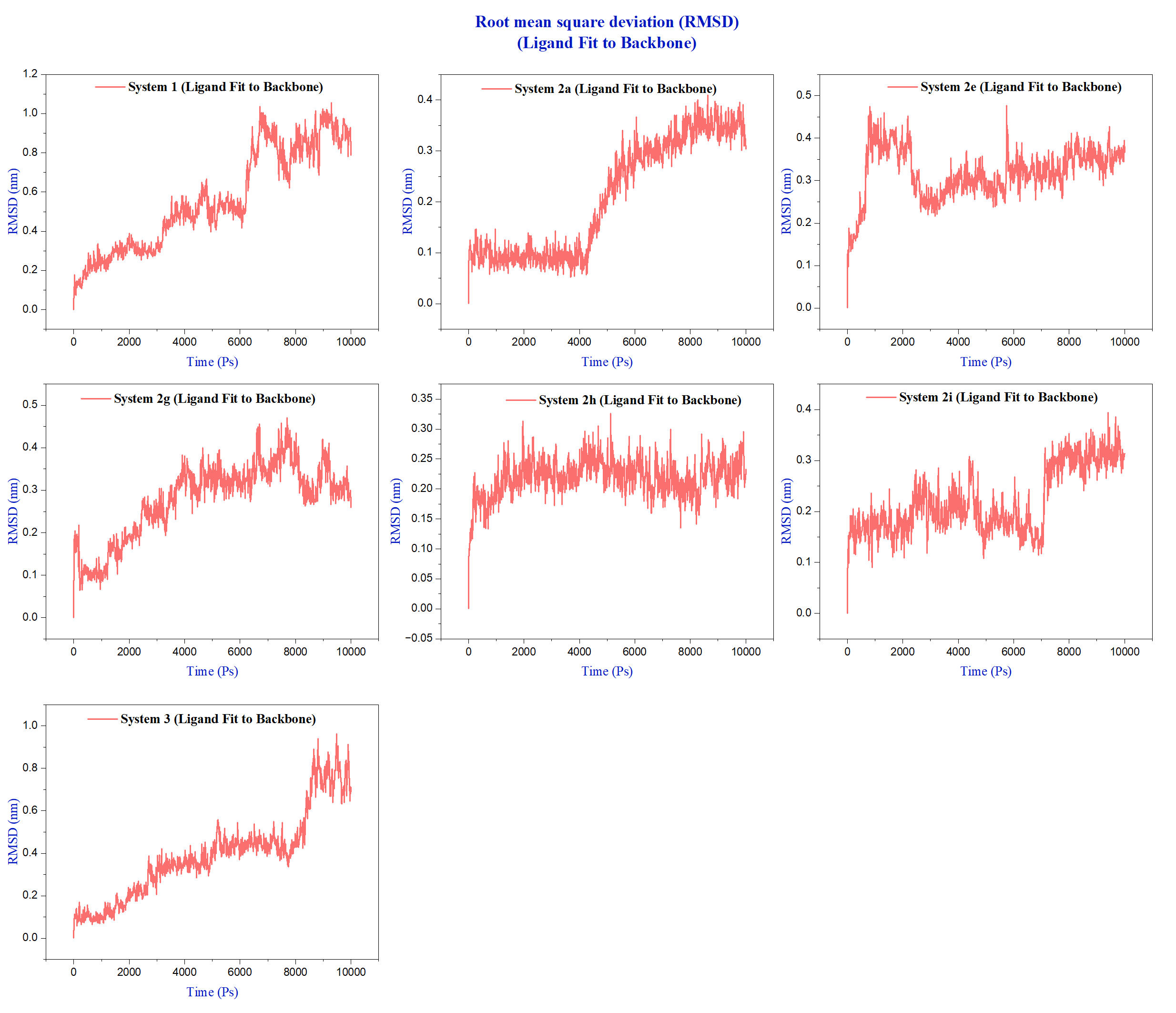


**Fig. S19.** RMSD trajectories of ligands fitted to the backbone over 10 ns MD simulations for systems **1, 2a, 2e**, **2g-2i** and **3**.


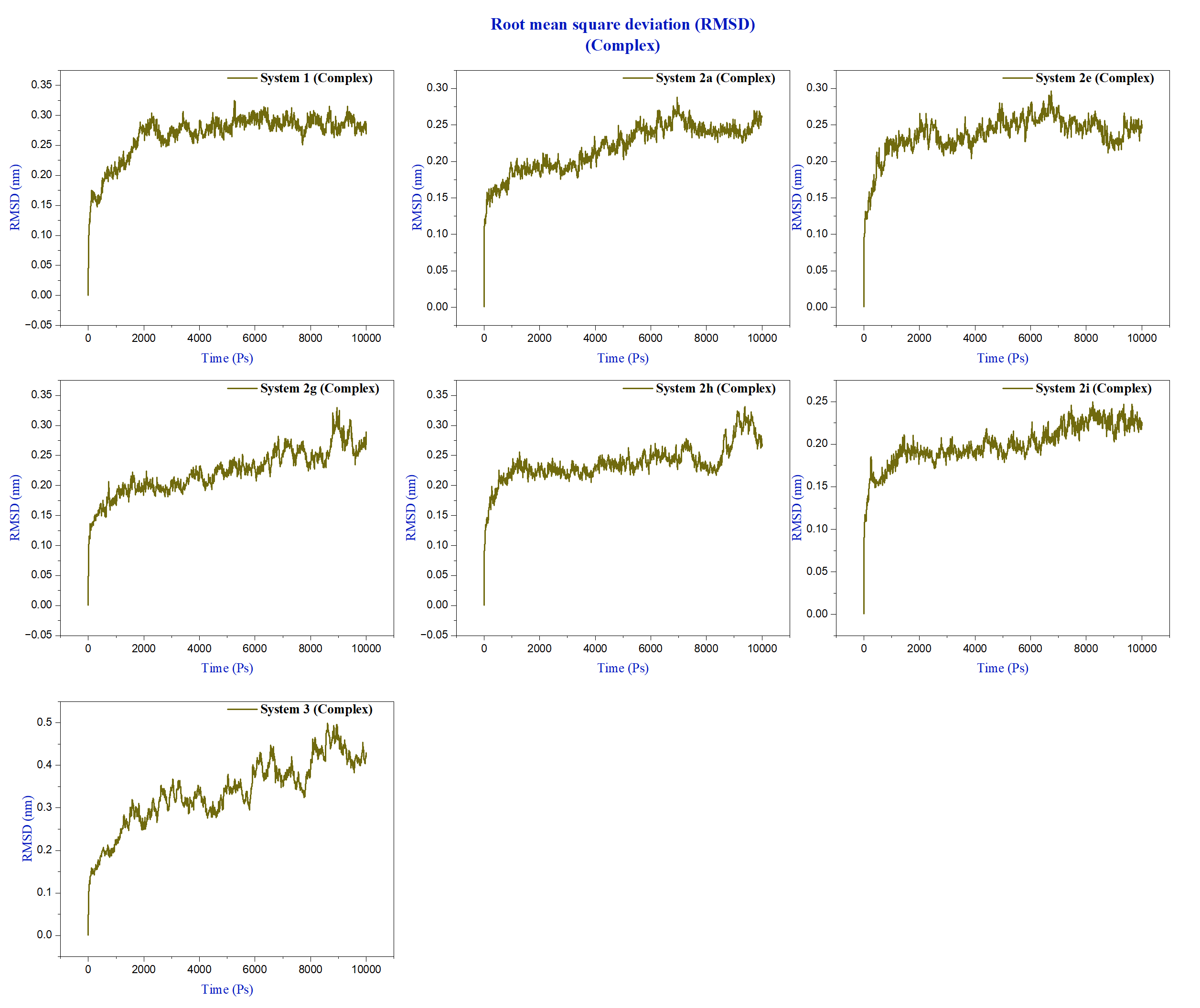


**Fig. S20.** RMSD trajectories of the CDK2–ligand complexes over 10 ns MD simulations for systems **1, 2a, 2e**, **2g-2i** and **3**.


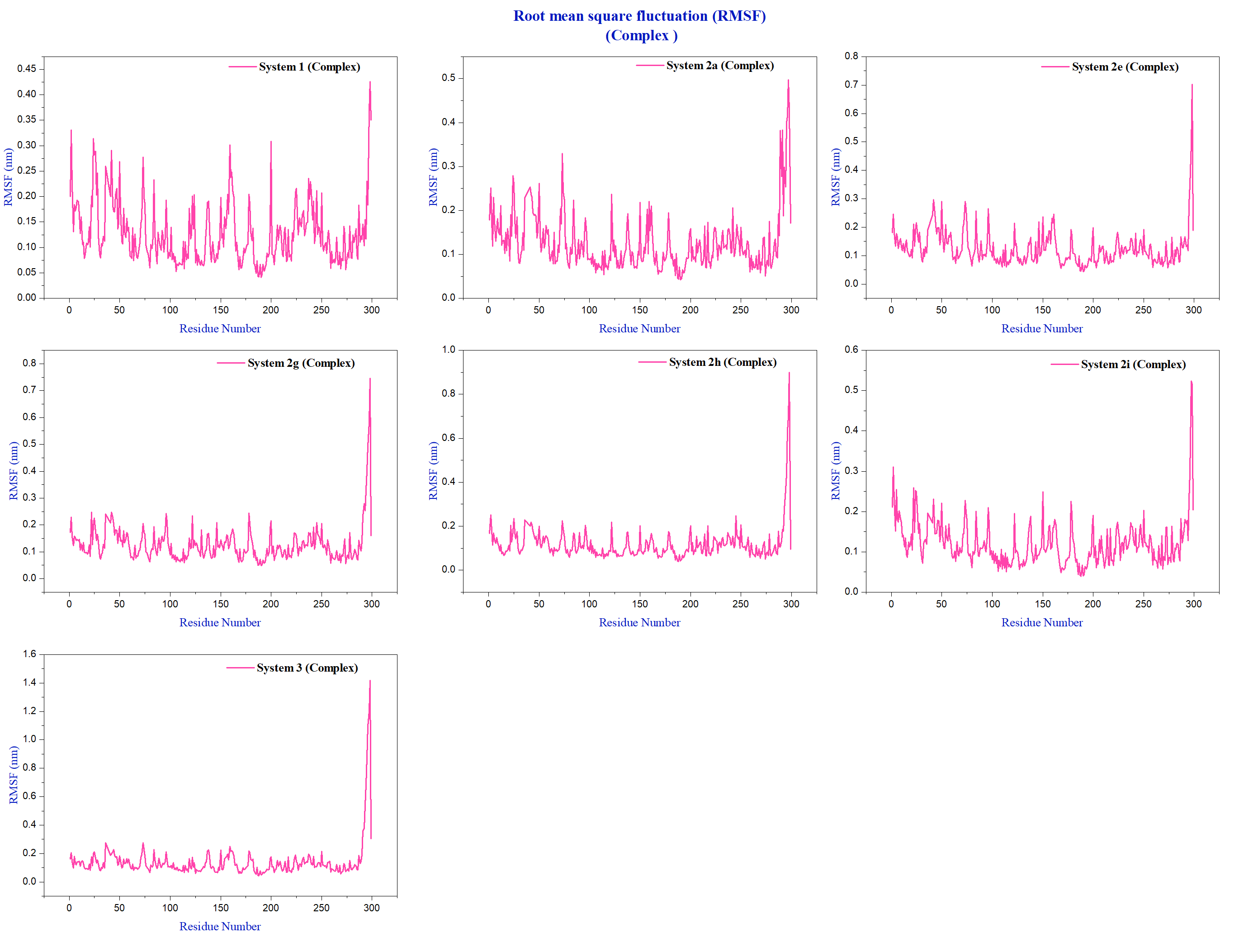


**Fig. S21.** RMSF profiles of CDK2 in complex with ligands **1, 2a, 2e**, **2g-2i** and **3**, highlighting residue-level structural flexibility.

**
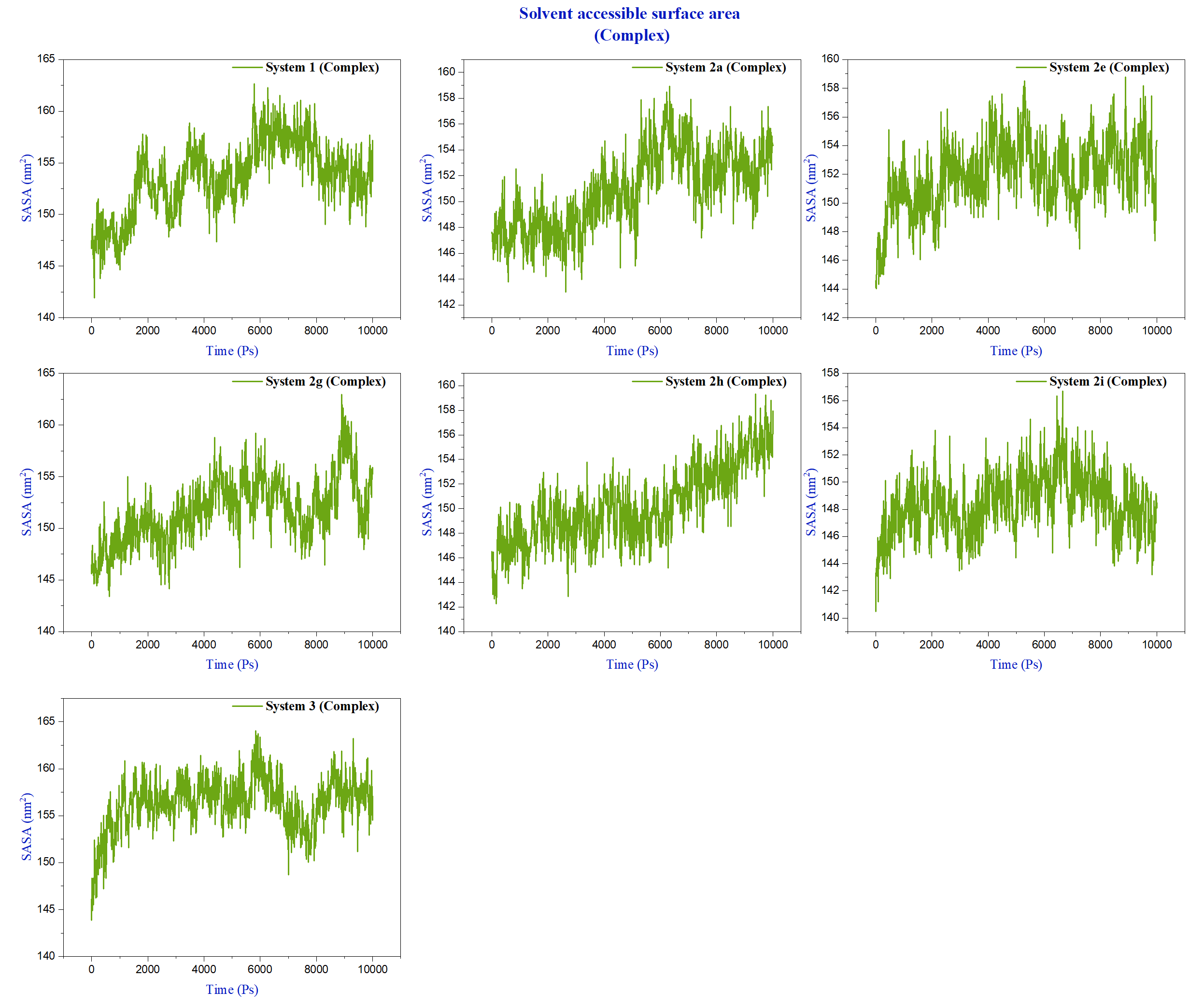
**

**Fig. S22.** SASA profiles of CDK2–ligand complexes during 10 ns molecular dynamics simulations for systems **1, 2a, 2e, 2g–2i, and 3**.


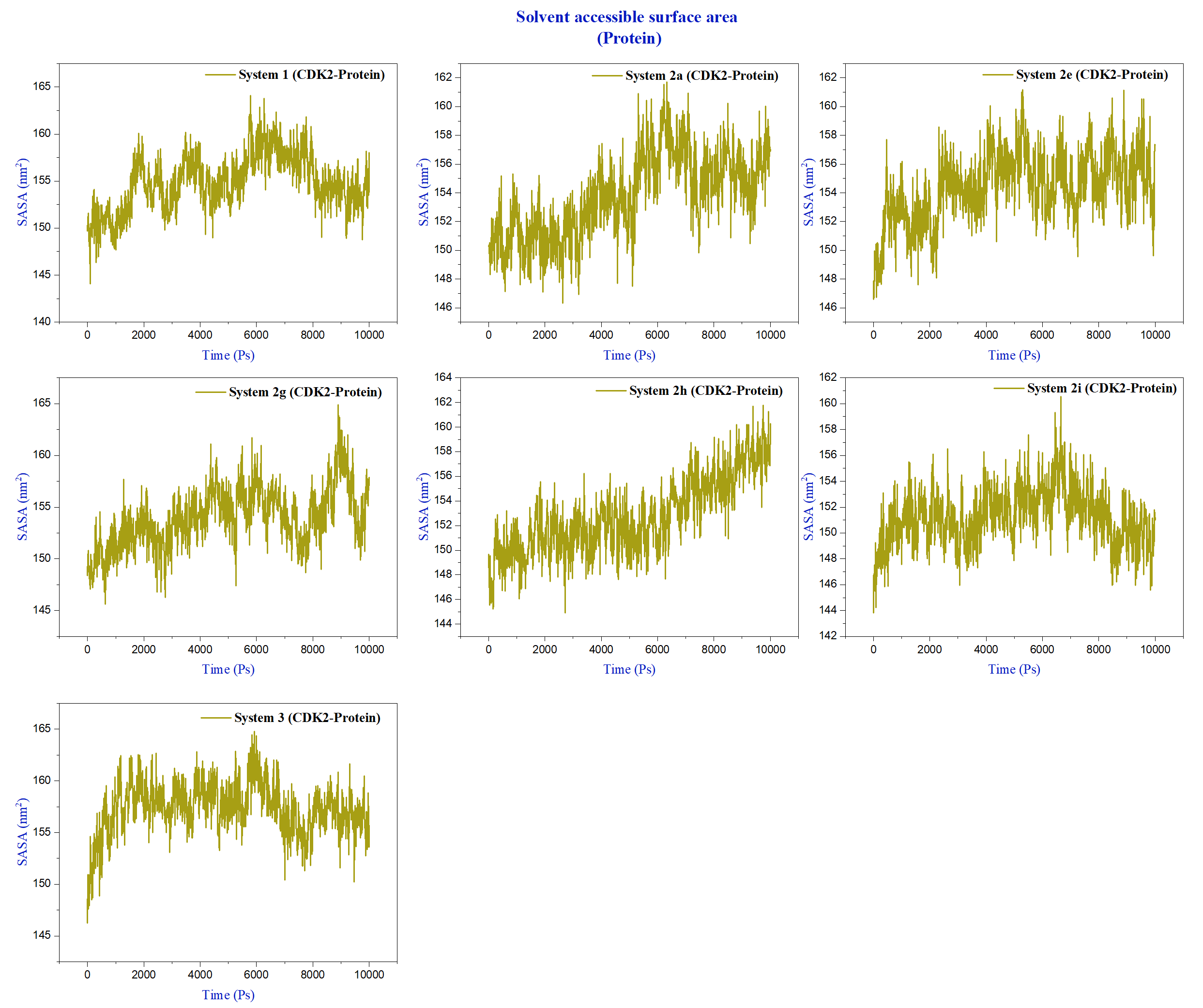


**Fig. S23.** SASA profiles of CDK2 protein over 10 ns MD simulations for systems **1, 2a, 2e**, **2g-2i** and **3**.


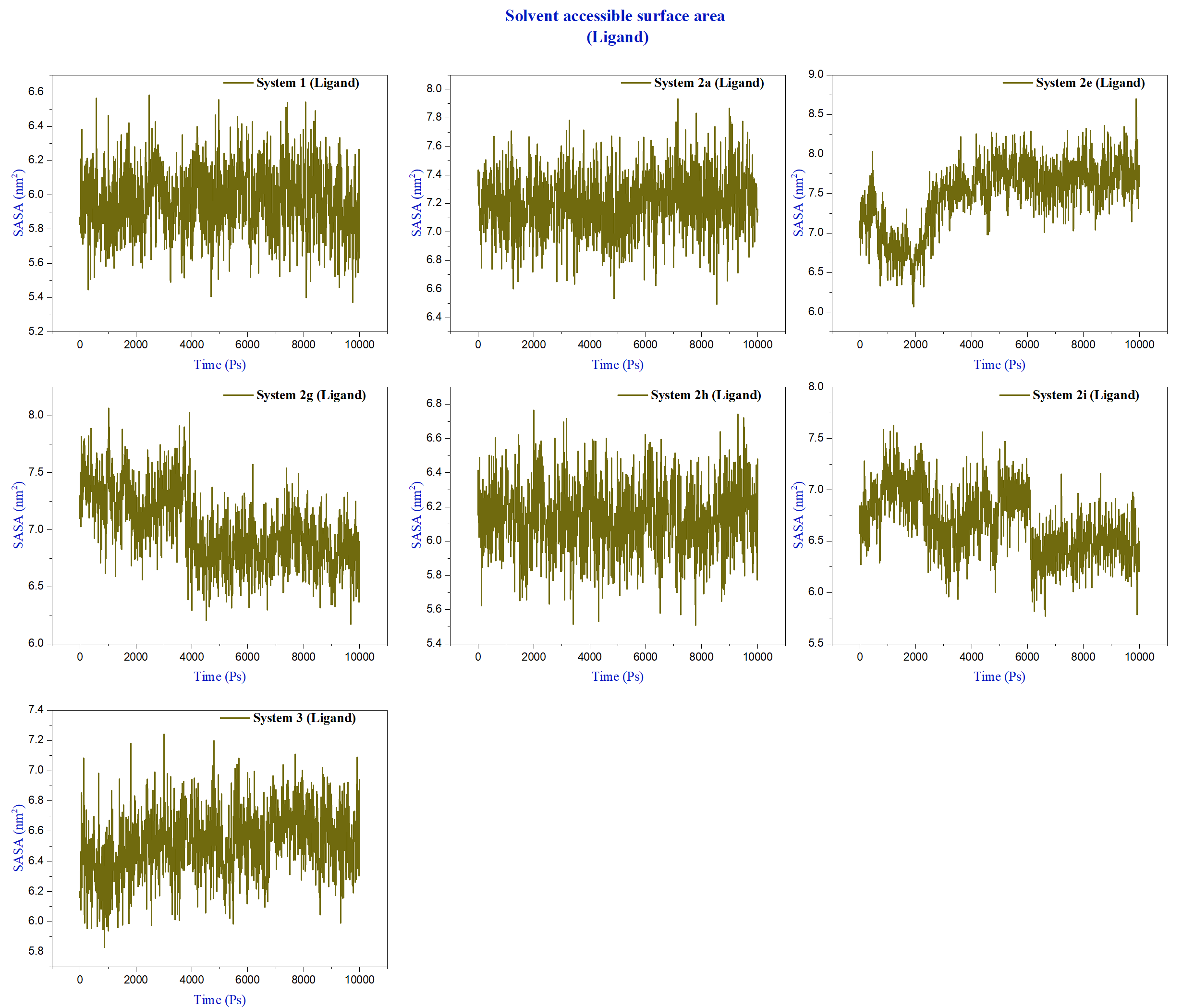


**Fig. S24.** SASA profiles of ligands during 10 ns molecular dynamics simulations in systems **1, 2a, 2e, 2g–2i, and 3**.


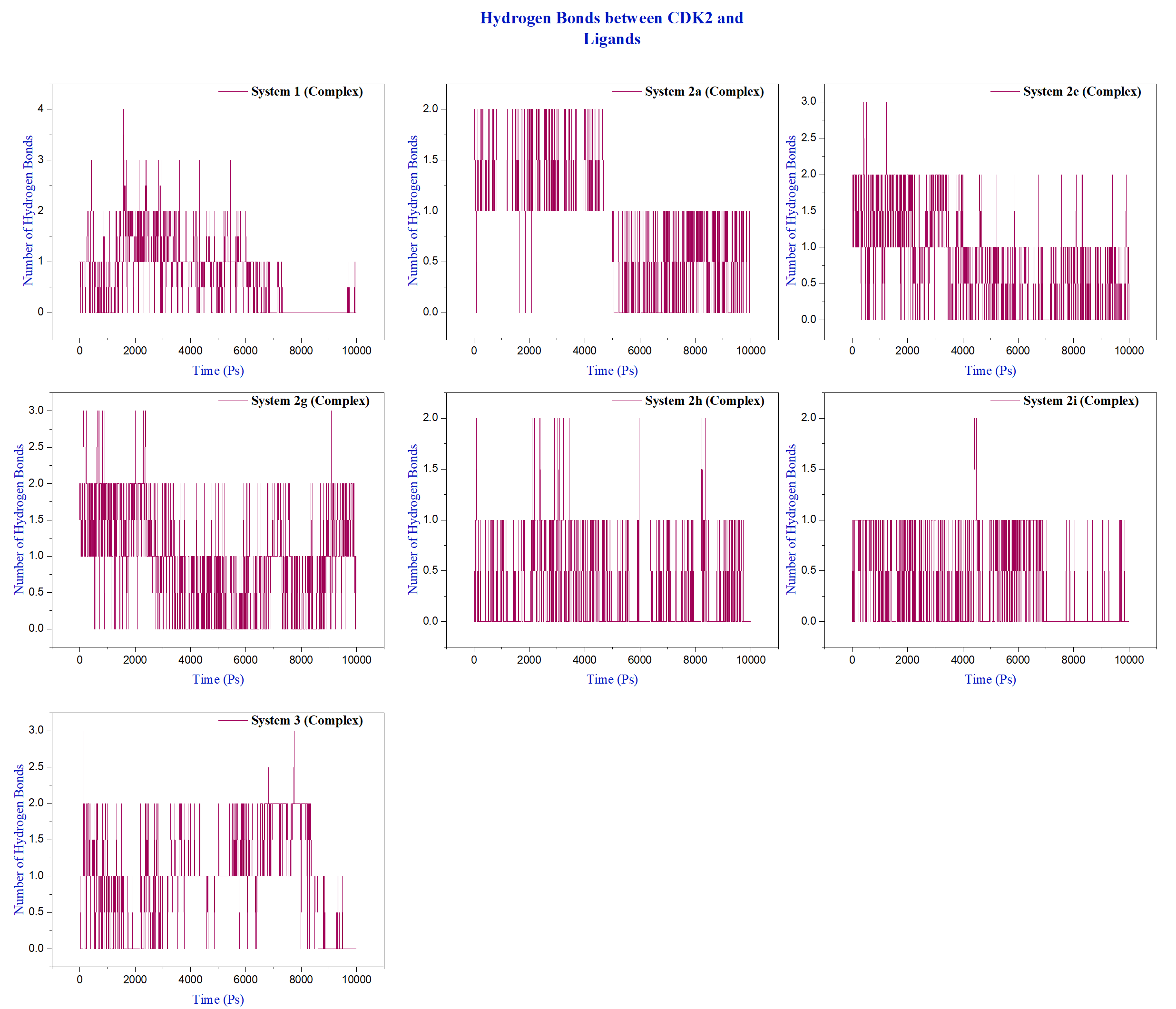


**Fig. S25.** Time evolution of protein–ligand hydrogen bonds for systems **1, 2a, 2e**, **2g-2i** and **3** obtained from molecular dynamics simulations.

**
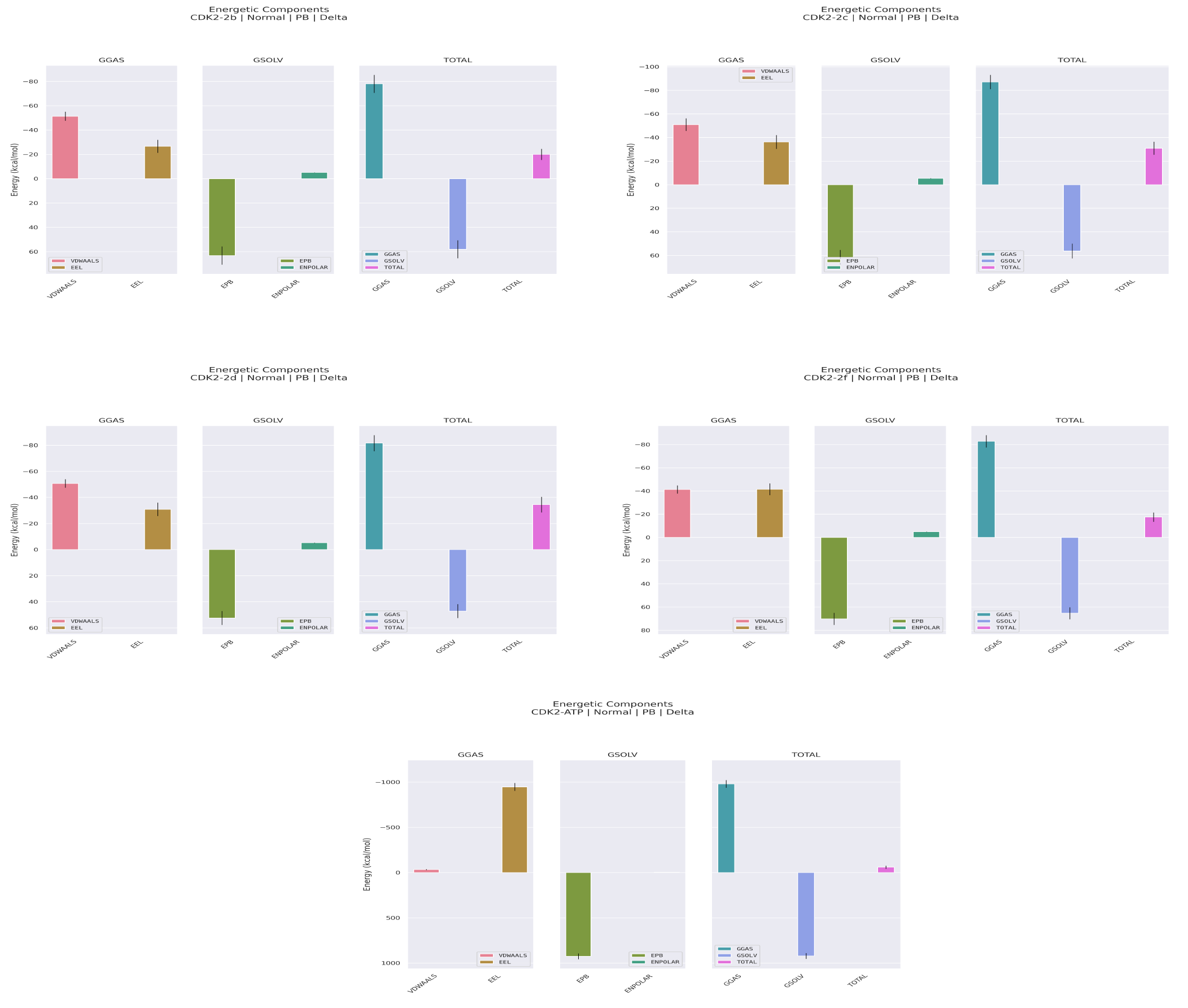
**

**Fig. S26.** MM-PBSA energy decomposition profiles for CDK2–ligand complexes. Gas-phase (ΔG_GAS), solvation (ΔG_SOLV), and total binding free energies (ΔG_TOTAL) are shown for **1, 2a, 2e**, **2g-2i** and **3**, highlighting key energetic differences underlying their binding affinities.

**Table S1:** Fukui functions (local reactivity indices) include electrophilic (F⁻), nucleophilic (F⁺), and radical attacks (F⁰) on **1 (**Gas phase**)**. f⁻ = _qk_(N) - _qk_(N−1); f⁺ = _qk_(N+1) - _qk_(N); and f⁰ = _qk_(N+1) - _qk_(N−1). qk represents the electron population at the k^th^ atom in a neutral (N), anionic (N+1), or cationic (N−1) species (derived from Hirshfeld charges). The condensed dual descriptor (CDD = f⁺ − f⁻) differentiates nucleophilic (CDD > 0) from electrophilic (CDD < 0) attack preferences at each atomic center.

| Atom | q(N) | q(N+1) | q(N-1) | f⁻ | f⁺ | f⁰ | CDD |
| --- | --- | --- | --- | --- | --- | --- | --- |
| 1(N) | -0.0163 | -0.0276 | -0.0047 | 0.0116 | 0.0113 | 0.0114 | -0.0003 |
| 2(C) | 0.1598 | 0.0968 | 0.1696 | 0.0098 | 0.063 | 0.0364 | 0.0531 |
| 3(C) | 0.0192 | -0.0273 | 0.0598 | 0.0406 | 0.0465 | 0.0435 | 0.006 |
| 4(N) | -0.0649 | -0.0718 | -0.025 | 0.0399 | 0.0068 | 0.0234 | -0.0331 |
| 5(C) | 0.0908 | 0.0471 | 0.1053 | 0.0145 | 0.0437 | 0.0291 | 0.0293 |
| 6(C) | 0.0362 | 0.0485 | 0.0279 | -0.0084 | -0.0123 | -0.0103 | -0.0039 |
| 7(C) | -0.0387 | -0.0406 | -0.0347 | 0.004 | 0.0018 | 0.0029 | -0.0022 |
| 8(C) | -0.0373 | -0.0528 | -0.0198 | 0.0175 | 0.0155 | 0.0165 | -0.0019 |
| 9(C) | -0.0385 | -0.0608 | -0.0172 | 0.0213 | 0.0223 | 0.0218 | 0.001 |
| 10(C) | -0.0374 | -0.0527 | -0.0234 | 0.0139 | 0.0154 | 0.0146 | 0.0014 |
| 11(C) | -0.0406 | -0.0424 | -0.0386 | 0.002 | 0.0019 | 0.0019 | -0.0002 |
| 12(O) | -0.2626 | -0.3516 | -0.2208 | 0.0418 | 0.0891 | 0.0654 | 0.0473 |
| 13(S) | -0.2548 | -0.431 | -0.0426 | 0.2121 | 0.1762 | 0.1942 | -0.0359 |
| 14(C) | -0.0391 | -0.116 | 0.0101 | 0.0492 | 0.0769 | 0.063 | 0.0276 |
| 15(C) | -0.014 | -0.0321 | 0.0121 | 0.0261 | 0.0181 | 0.0221 | -0.0081 |
| 16(C) | -0.0472 | -0.0872 | -0.0279 | 0.0193 | 0.0401 | 0.0297 | 0.0208 |
| 17(C) | 0.0615 | 0.0378 | 0.098 | 0.0365 | 0.0237 | 0.0301 | -0.0128 |
| 18(C) | -0.0639 | -0.1194 | -0.0019 | 0.062 | 0.0555 | 0.0588 | -0.0065 |
| 19(C) | -0.0662 | -0.0939 | -0.0403 | 0.0259 | 0.0277 | 0.0268 | 0.0019 |
| 20(C) | 0.0602 | 0.0397 | 0.1028 | 0.0426 | 0.0205 | 0.0316 | -0.0221 |
| 21(O) | -0.0969 | -0.0979 | -0.0711 | 0.0258 | 0.001 | 0.0134 | -0.0248 |
| 22(C) | 0.0021 | -0.0059 | 0.014 | 0.0118 | 0.008 | 0.0099 | -0.0038 |
| 23(O) | -0.1258 | -0.1386 | -0.0906 | 0.0352 | 0.0128 | 0.024 | -0.0224 |
| 24(C) | -0.003 | -0.0127 | 0.0093 | 0.0123 | 0.0097 | 0.011 | -0.0025 |
| 25(H) | 0.1154 | 0.1039 | 0.1283 | 0.0129 | 0.0114 | 0.0122 | -0.0015 |
| 26(H) | 0.0498 | 0.0474 | 0.0532 | 0.0034 | 0.0024 | 0.0029 | -0.001 |
| 27(H) | 0.048 | 0.0343 | 0.0612 | 0.0132 | 0.0136 | 0.0134 | 0.0004 |
| 28(H) | 0.0464 | 0.0299 | 0.0615 | 0.0151 | 0.0165 | 0.0158 | 0.0014 |
| 29(H) | 0.0481 | 0.0346 | 0.0602 | 0.0121 | 0.0135 | 0.0128 | 0.0014 |
| 30(H) | 0.046 | 0.0423 | 0.0479 | 0.0019 | 0.0037 | 0.0028 | 0.0018 |
| 31(H) | 0.0512 | 0.0222 | 0.0751 | 0.0239 | 0.029 | 0.0265 | 0.0051 |
| 32(H) | 0.0538 | 0.0339 | 0.072 | 0.0181 | 0.0199 | 0.019 | 0.0018 |
| 33(H) | 0.0441 | 0.0157 | 0.0714 | 0.0274 | 0.0284 | 0.0279 | 0.001 |
| 34(H) | 0.0441 | 0.0246 | 0.0639 | 0.0199 | 0.0195 | 0.0197 | -0.0003 |
| 35(H) | 0.055 | 0.0459 | 0.0667 | 0.0117 | 0.0091 | 0.0104 | -0.0026 |
| 36(H) | 0.0423 | 0.0312 | 0.0575 | 0.0152 | 0.0111 | 0.0131 | -0.0041 |
| 37(H) | 0.0442 | 0.0322 | 0.0603 | 0.0161 | 0.012 | 0.014 | -0.004 |
| 38(H) | 0.0522 | 0.0373 | 0.0697 | 0.0174 | 0.0149 | 0.0162 | -0.0025 |
| 39(H) | 0.0385 | 0.0287 | 0.0508 | 0.0124 | 0.0097 | 0.011 | -0.0027 |
| 40(H) | 0.0387 | 0.0292 | 0.0507 | 0.012 | 0.0095 | 0.0107 | -0.0026 |

**Table S2:** Fukui functions (local reactivity indices) include electrophilic (F⁻), nucleophilic (F⁺), and radical attacks (F⁰) on **1 (**DMSO phase**)**. f⁻ = _qk_(N) - _qk_(N−1); f⁺ = _qk_(N+1) - _qk_(N); and f⁰ = _qk_(N+1) - _qk_(N−1). qk represents the electron population at the k^th^ atom in a neutral (N), anionic (N+1), or cationic (N−1) species (derived from Hirshfeld charges). The condensed dual descriptor (CDD = f⁺ − f⁻) differentiates nucleophilic (CDD > 0) from electrophilic (CDD < 0) attack preferences at each atomic center.

| Atom | q(N) | q(N+1) | q(N-1) | f⁻ | f⁺ | f⁰ | CDD |
| --- | --- | --- | --- | --- | --- | --- | --- |
| 1(N) | -0.0129 | -0.0289 | -0.0062 | 0.0067 | 0.016 | 0.0114 | 0.0093 |
| 2(C) | 0.1588 | 0.0797 | 0.1745 | 0.0157 | 0.0791 | 0.0474 | 0.0634 |
| 3(C) | 0.0169 | -0.0431 | 0.0618 | 0.0448 | 0.06 | 0.0524 | 0.0151 |
| 4(N) | -0.0546 | -0.0726 | -0.0276 | 0.027 | 0.0179 | 0.0225 | -0.0091 |
| 5(C) | 0.0965 | 0.0386 | 0.1101 | 0.0136 | 0.058 | 0.0358 | 0.0444 |
| 6(C) | 0.025 | 0.0269 | 0.0244 | -0.0005 | -0.002 | -0.0012 | -0.0014 |
| 7(C) | -0.0465 | -0.0534 | -0.0434 | 0.0031 | 0.0069 | 0.005 | 0.0038 |
| 8(C) | -0.0412 | -0.0473 | -0.0383 | 0.0029 | 0.0061 | 0.0045 | 0.0032 |
| 9(C) | -0.0399 | -0.0482 | -0.0361 | 0.0038 | 0.0083 | 0.006 | 0.0045 |
| 10(C) | -0.0412 | -0.0473 | -0.0383 | 0.0029 | 0.0061 | 0.0045 | 0.0032 |
| 11(C) | -0.0464 | -0.0533 | -0.0433 | 0.0031 | 0.0069 | 0.005 | 0.0038 |
| 12(O) | -0.3012 | -0.3923 | -0.2733 | 0.0279 | 0.091 | 0.0595 | 0.0631 |
| 13(S) | -0.3416 | -0.5035 | -0.2409 | 0.1007 | 0.1619 | 0.1313 | 0.0612 |
| 14(C) | -0.0295 | -0.1391 | 0.0011 | 0.0306 | 0.1096 | 0.0701 | 0.079 |
| 15(C) | -0.0244 | -0.0505 | 0.0302 | 0.0547 | 0.0261 | 0.0404 | -0.0286 |
| 16(C) | -0.0563 | -0.101 | -0.0232 | 0.033 | 0.0448 | 0.0389 | 0.0117 |
| 17(C) | 0.0518 | 0.033 | 0.1177 | 0.0659 | 0.0189 | 0.0424 | -0.047 |
| 18(C) | -0.0592 | -0.1079 | 0.0203 | 0.0796 | 0.0487 | 0.0641 | -0.0309 |
| 19(C) | -0.0649 | -0.089 | -0.0155 | 0.0494 | 0.0241 | 0.0367 | -0.0253 |
| 20(C) | 0.058 | 0.0301 | 0.1347 | 0.0767 | 0.0279 | 0.0523 | -0.0488 |
| 21(O) | -0.1009 | -0.1085 | -0.0457 | 0.0552 | 0.0076 | 0.0314 | -0.0476 |
| 22(C) | 0.0065 | 0.0007 | 0.0234 | 0.0169 | 0.0059 | 0.0114 | -0.011 |
| 23(O) | -0.1386 | -0.1458 | -0.0799 | 0.0586 | 0.0073 | 0.0329 | -0.0514 |
| 24(C) | -0.0019 | -0.0062 | 0.0136 | 0.0155 | 0.0043 | 0.0099 | -0.0112 |
| 25(H) | 0.1285 | 0.1139 | 0.1411 | 0.0126 | 0.0146 | 0.0136 | 0.002 |
| 26(H) | 0.0613 | 0.0562 | 0.0634 | 0.0021 | 0.0051 | 0.0036 | 0.003 |
| 27(H) | 0.0603 | 0.0568 | 0.062 | 0.0016 | 0.0035 | 0.0026 | 0.0018 |
| 28(H) | 0.0594 | 0.0556 | 0.0611 | 0.0018 | 0.0038 | 0.0028 | 0.002 |
| 29(H) | 0.0603 | 0.0569 | 0.062 | 0.0016 | 0.0035 | 0.0026 | 0.0018 |
| 30(H) | 0.0616 | 0.0566 | 0.0636 | 0.0021 | 0.005 | 0.0035 | 0.0029 |
| 31(H) | 0.059 | 0.0174 | 0.0775 | 0.0185 | 0.0416 | 0.0301 | 0.0232 |
| 32(H) | 0.0597 | 0.0395 | 0.0814 | 0.0218 | 0.0202 | 0.021 | -0.0016 |
| 33(H) | 0.0603 | 0.0411 | 0.0911 | 0.0309 | 0.0191 | 0.025 | -0.0117 |
| 34(H) | 0.0606 | 0.0481 | 0.0853 | 0.0248 | 0.0124 | 0.0186 | -0.0124 |
| 35(H) | 0.0591 | 0.0535 | 0.0724 | 0.0133 | 0.0056 | 0.0094 | -0.0077 |
| 36(H) | 0.0536 | 0.0479 | 0.0716 | 0.0179 | 0.0057 | 0.0118 | -0.0122 |
| 37(H) | 0.0537 | 0.0478 | 0.0717 | 0.0179 | 0.006 | 0.012 | -0.012 |
| 38(H) | 0.0551 | 0.0514 | 0.0674 | 0.0122 | 0.0037 | 0.008 | -0.0085 |
| 39(H) | 0.0479 | 0.0435 | 0.0645 | 0.0166 | 0.0044 | 0.0105 | -0.0122 |
| 40(H) | 0.048 | 0.0436 | 0.0645 | 0.0165 | 0.0044 | 0.0105 | -0.0121 |

**Table S3:** Fukui functions (local reactivity indices) include electrophilic (F⁻), nucleophilic (F⁺), and radical attacks (F⁰) on **2a (**Gas phase**)**. f⁻ = _qk_(N) - _qk_(N−1); f⁺ = _qk_(N+1) - _qk_(N); and f⁰ = _qk_(N+1) - _qk_(N−1). qk represents the electron population at the k^th^ atom in a neutral (N), anionic (N+1), or cationic (N−1) species (derived from Hirshfeld charges). The condensed dual descriptor (CDD = f⁺ − f⁻) differentiates nucleophilic (CDD > 0) from electrophilic (CDD < 0) attack preferences at each atomic center.

| Atom | q(N) | q(N+1) | q(N-1) | f⁻ | f⁺ | f⁰ | CDD |
| --- | --- | --- | --- | --- | --- | --- | --- |
| 1(N) | -0.0283 | -0.0407 | -0.0146 | 0.0137 | 0.0124 | 0.0131 | -0.0013 |
| 2(C) | 0.1566 | 0.0974 | 0.1746 | 0.018 | 0.0592 | 0.0386 | 0.0413 |
| 3(C) | 0.0049 | -0.044 | 0.0522 | 0.0473 | 0.0488 | 0.0481 | 0.0015 |
| 4(N) | -0.1971 | -0.2113 | -0.1545 | 0.0426 | 0.0142 | 0.0284 | -0.0284 |
| 5(C) | 0.0748 | 0.0256 | 0.1053 | 0.0304 | 0.0492 | 0.0398 | 0.0187 |
| 6(C) | 0.0353 | 0.0455 | 0.0264 | -0.0089 | -0.0103 | -0.0096 | -0.0014 |
| 7(C) | -0.046 | -0.0498 | -0.0439 | 0.0022 | 0.0038 | 0.003 | 0.0016 |
| 8(C) | -0.0379 | -0.0528 | -0.0248 | 0.0131 | 0.0149 | 0.014 | 0.0017 |
| 9(C) | -0.0393 | -0.061 | -0.0197 | 0.0196 | 0.0217 | 0.0207 | 0.0021 |
| 10(C) | -0.0355 | -0.0495 | -0.0229 | 0.0126 | 0.014 | 0.0133 | 0.0014 |
| 11(C) | -0.0378 | -0.0393 | -0.0355 | 0.0024 | 0.0015 | 0.0019 | -0.0009 |
| 12(O) | -0.2445 | -0.3211 | -0.1962 | 0.0484 | 0.0766 | 0.0625 | 0.0282 |
| 13(S) | 0.0287 | -0.0371 | 0.13 | 0.1013 | 0.0657 | 0.0835 | -0.0356 |
| 14(C) | -0.0176 | -0.108 | 0.0111 | 0.0287 | 0.0904 | 0.0595 | 0.0617 |
| 15(C) | -0.0175 | -0.0333 | 0.0125 | 0.03 | 0.0158 | 0.0229 | -0.0142 |
| 16(C) | -0.0482 | -0.0873 | -0.0359 | 0.0123 | 0.039 | 0.0257 | 0.0267 |
| 17(C) | 0.0588 | 0.0375 | 0.0987 | 0.0399 | 0.0213 | 0.0306 | -0.0185 |
| 18(C) | -0.0632 | -0.1221 | -0.0016 | 0.0616 | 0.0589 | 0.0603 | -0.0026 |
| 19(C) | -0.0747 | -0.1056 | -0.0435 | 0.0313 | 0.0308 | 0.0311 | -0.0004 |
| 20(C) | 0.0657 | 0.0402 | 0.116 | 0.0503 | 0.0255 | 0.0379 | -0.0247 |
| 21(C) | -0.0433 | -0.0508 | -0.0358 | 0.0076 | 0.0075 | 0.0075 | -0.0001 |
| 22(C) | -0.0063 | 0.0003 | -0.0147 | -0.0085 | -0.0066 | -0.0075 | 0.0019 |
| 23(C) | -0.0423 | -0.0454 | -0.0407 | 0.0016 | 0.003 | 0.0023 | 0.0014 |
| 24(C) | -0.0408 | -0.0525 | -0.0301 | 0.0107 | 0.0118 | 0.0112 | 0.0011 |
| 25(C) | -0.0416 | -0.057 | -0.0263 | 0.0154 | 0.0154 | 0.0154 | 0 |
| 26(C) | -0.0412 | -0.0527 | -0.0305 | 0.0107 | 0.0116 | 0.0111 | 0.0009 |
| 27(C) | -0.0428 | -0.0463 | -0.0412 | 0.0017 | 0.0034 | 0.0025 | 0.0017 |
| 28(O) | -0.1119 | -0.1185 | -0.0691 | 0.0428 | 0.0066 | 0.0247 | -0.0362 |
| 29(C) | -0.0021 | -0.0123 | 0.0128 | 0.0149 | 0.0102 | 0.0126 | -0.0047 |
| 30(O) | -0.1252 | -0.1346 | -0.0879 | 0.0373 | 0.0094 | 0.0234 | -0.0279 |
| 31(C) | -0.0054 | -0.015 | 0.0074 | 0.0128 | 0.0096 | 0.0112 | -0.0032 |
| 32(H) | 0.0444 | 0.0407 | 0.0468 | 0.0024 | 0.0037 | 0.0031 | 0.0013 |
| 33(H) | 0.0481 | 0.0352 | 0.0593 | 0.0112 | 0.0129 | 0.0121 | 0.0017 |
| 34(H) | 0.0466 | 0.031 | 0.0603 | 0.0137 | 0.0156 | 0.0147 | 0.002 |
| 35(H) | 0.0488 | 0.0365 | 0.0596 | 0.0109 | 0.0123 | 0.0116 | 0.0014 |
| 36(H) | 0.0515 | 0.0501 | 0.0527 | 0.0012 | 0.0014 | 0.0013 | 0.0002 |
| 37(H) | 0.0417 | 0.0062 | 0.0574 | 0.0157 | 0.0355 | 0.0256 | 0.0199 |
| 38(H) | 0.0351 | 0.0232 | 0.0458 | 0.0107 | 0.0118 | 0.0113 | 0.0012 |
| 39(H) | 0.0412 | 0.0113 | 0.0691 | 0.0279 | 0.03 | 0.0289 | 0.0021 |
| 40(H) | 0.0397 | 0.0179 | 0.0619 | 0.0222 | 0.0218 | 0.022 | -0.0004 |
| 41(H) | 0.0459 | 0.0402 | 0.0528 | 0.0069 | 0.0056 | 0.0063 | -0.0013 |
| 42(H) | 0.0454 | 0.0397 | 0.0524 | 0.007 | 0.0057 | 0.0064 | -0.0013 |
| 43(H) | 0.0457 | 0.0434 | 0.0469 | 0.0012 | 0.0023 | 0.0018 | 0.0011 |
| 44(H) | 0.0463 | 0.0368 | 0.0552 | 0.0089 | 0.0095 | 0.0092 | 0.0006 |
| 45(H) | 0.0457 | 0.0344 | 0.0565 | 0.0109 | 0.0113 | 0.0111 | 0.0004 |
| 46(H) | 0.0461 | 0.0366 | 0.0549 | 0.0089 | 0.0094 | 0.0092 | 0.0005 |
| 47(H) | 0.0451 | 0.0427 | 0.0463 | 0.0012 | 0.0024 | 0.0018 | 0.0013 |
| 48(H) | 0.0507 | 0.0371 | 0.0681 | 0.0173 | 0.0136 | 0.0155 | -0.0037 |
| 49(H) | 0.0386 | 0.0268 | 0.0552 | 0.0166 | 0.0118 | 0.0142 | -0.0048 |
| 50(H) | 0.0387 | 0.0269 | 0.0552 | 0.0166 | 0.0118 | 0.0142 | -0.0048 |
| 51(H) | 0.05 | 0.0354 | 0.0675 | 0.0176 | 0.0146 | 0.0161 | -0.003 |
| 52(H) | 0.0358 | 0.0254 | 0.0494 | 0.0136 | 0.0104 | 0.012 | -0.0032 |
| 53(H) | 0.0356 | 0.0251 | 0.0492 | 0.0137 | 0.0105 | 0.0121 | -0.0032 |

**Table S4:** Fukui functions (local reactivity indices) include electrophilic (F⁻), nucleophilic (F⁺), and radical attacks (F⁰) on **2a (**DMSO phase**)**. f⁻ = _qk_(N) - _qk_(N−1); f⁺ = _qk_(N+1) - _qk_(N); and f⁰ = _qk_(N+1) - _qk_(N−1). qk represents the electron population at the k^th^ atom in a neutral (N), anionic (N+1), or cationic (N−1) species (derived from Hirshfeld charges). The condensed dual descriptor (CDD = f⁺ − f⁻) differentiates nucleophilic (CDD > 0) from electrophilic (CDD < 0) attack preferences at each atomic center.

| Atom | q(N) | q(N+1) | q(N-1) | f⁻ | f⁺ | f⁰ | CDD |
| --- | --- | --- | --- | --- | --- | --- | --- |
| 1(N) | -0.0245 | -0.0418 | 0.0464 | 0.0709 | 0.0173 | 0.0441 | -0.0536 |
| 2(C) | 0.1539 | 0.0767 | 0.0012 | -0.1527 | 0.0773 | -0.0377 | 0.23 |
| 3(C) | -0.0023 | -0.074 | 0.1734 | 0.1757 | 0.0717 | 0.1237 | -0.104 |
| 4(N) | -0.2251 | -0.2581 | 0.023 | 0.2481 | 0.033 | 0.1406 | -0.2151 |
| 5(C) | 0.0787 | 0.0262 | 0.0617 | -0.017 | 0.0524 | 0.0177 | 0.0694 |
| 6(C) | 0.0246 | 0.026 | -0.1932 | -0.2178 | -0.0014 | -0.1096 | 0.2164 |
| 7(C) | -0.0458 | -0.052 | -0.0159 | 0.0299 | 0.0062 | 0.018 | -0.0237 |
| 8(C) | -0.0404 | -0.0463 | -0.2572 | -0.2168 | 0.006 | -0.1054 | 0.2228 |
| 9(C) | -0.0389 | -0.0472 | -0.0357 | 0.0033 | 0.0083 | 0.0058 | 0.005 |
| 10(C) | -0.0402 | -0.0462 | 0.1341 | 0.1743 | 0.006 | 0.0901 | -0.1684 |
| 11(C) | -0.0448 | -0.051 | 0.1033 | 0.1481 | 0.0062 | 0.0772 | -0.1418 |
| 12(O) | -0.2908 | -0.3746 | 0.0238 | 0.3146 | 0.0838 | 0.1992 | -0.2309 |
| 13(S) | 0.0169 | -0.0426 | 0.1139 | 0.097 | 0.0595 | 0.0782 | -0.0375 |
| 14(C) | -0.0253 | -0.1476 | 0.0536 | 0.0788 | 0.1223 | 0.1006 | 0.0434 |
| 15(C) | -0.0301 | -0.0583 | -0.0198 | 0.0103 | 0.0282 | 0.0193 | 0.0179 |
| 16(C) | -0.0651 | -0.1121 | -0.056 | 0.0091 | 0.047 | 0.028 | 0.0379 |
| 17(C) | 0.0475 | 0.0258 | 0.0694 | 0.0219 | 0.0217 | 0.0218 | -0.0002 |
| 18(C) | -0.0623 | -0.118 | -0.0416 | 0.0206 | 0.0558 | 0.0382 | 0.0352 |
| 19(C) | -0.0722 | -0.1026 | -0.0428 | 0.0294 | 0.0303 | 0.0299 | 0.0009 |
| 20(C) | 0.0588 | 0.0272 | 0.0142 | -0.0446 | 0.0315 | -0.0065 | 0.0761 |
| 21(C) | -0.0398 | -0.0475 | -0.0839 | -0.0441 | 0.0077 | -0.0182 | 0.0518 |
| 22(C) | -0.0119 | -0.012 | 0.0827 | 0.0946 | 0 | 0.0473 | -0.0946 |
| 23(C) | -0.0485 | -0.0515 | 0.0191 | 0.0675 | 0.003 | 0.0353 | -0.0645 |
| 24(C) | -0.0487 | -0.0514 | -0.0344 | 0.0143 | 0.0027 | 0.0085 | -0.0116 |
| 25(C) | -0.049 | -0.0527 | -0.0371 | 0.0119 | 0.0037 | 0.0078 | -0.0082 |
| 26(C) | -0.0487 | -0.0514 | 0.0642 | 0.1129 | 0.0027 | 0.0578 | -0.1102 |
| 27(C) | -0.0486 | -0.0517 | -0.0372 | 0.0113 | 0.0031 | 0.0072 | -0.0082 |
| 28(O) | -0.1204 | -0.1319 | 0.063 | 0.1834 | 0.0115 | 0.0975 | -0.1719 |
| 29(C) | 0.0011 | -0.0059 | 0.0883 | 0.0872 | 0.007 | 0.0471 | -0.0802 |
| 30(O) | -0.1409 | -0.1489 | 0.0117 | 0.1527 | 0.008 | 0.0803 | -0.1447 |
| 31(C) | -0.0036 | -0.0087 | 0.0684 | 0.072 | 0.0051 | 0.0386 | -0.0669 |
| 32(H) | 0.0607 | 0.0559 | 0.0678 | 0.0072 | 0.0047 | 0.0059 | -0.0025 |
| 33(H) | 0.0607 | 0.0572 | 0.07 | 0.0093 | 0.0035 | 0.0064 | -0.0058 |
| 34(H) | 0.0597 | 0.0559 | -0.0123 | -0.0719 | 0.0038 | -0.0341 | 0.0757 |
| 35(H) | 0.0606 | 0.0572 | 0.0592 | -0.0014 | 0.0035 | 0.001 | 0.0049 |
| 36(H) | 0.062 | 0.0573 | 0.0593 | -0.0027 | 0.0048 | 0.001 | 0.0075 |
| 37(H) | 0.0444 | -0.0024 | -0.0346 | -0.079 | 0.0468 | -0.0161 | 0.1258 |
| 38(H) | 0.0363 | 0.0174 | 0.0624 | 0.0262 | 0.0189 | 0.0225 | -0.0073 |
| 39(H) | 0.0579 | 0.0356 | 0.0626 | 0.0047 | 0.0222 | 0.0135 | 0.0175 |
| 40(H) | 0.0569 | 0.0417 | 0.063 | 0.0061 | 0.0152 | 0.0106 | 0.0091 |
| 41(H) | 0.0532 | 0.0451 | 0.0629 | 0.0097 | 0.0082 | 0.0089 | -0.0015 |
| 42(H) | 0.0531 | 0.045 | 0.0662 | 0.0131 | 0.0082 | 0.0106 | -0.005 |
| 43(H) | 0.0561 | 0.0539 | -0.0466 | -0.1027 | 0.0022 | -0.0502 | 0.1048 |
| 44(H) | 0.0559 | 0.0544 | -0.0467 | -0.1025 | 0.0015 | -0.0505 | 0.104 |
| 45(H) | 0.0553 | 0.0536 | 0.0617 | 0.0063 | 0.0017 | 0.004 | -0.0046 |
| 46(H) | 0.0559 | 0.0544 | -0.0469 | -0.1028 | 0.0015 | -0.0506 | 0.1042 |
| 47(H) | 0.0561 | 0.0538 | 0.0574 | 0.0013 | 0.0023 | 0.0018 | 0.0009 |
| 48(H) | 0.0564 | 0.0508 | -0.0468 | -0.1032 | 0.0056 | -0.0488 | 0.1088 |
| 49(H) | 0.0497 | 0.043 | 0.0574 | 0.0077 | 0.0067 | 0.0072 | -0.001 |
| 50(H) | 0.0491 | 0.0427 | -0.0464 | -0.0955 | 0.0064 | -0.0446 | 0.1019 |
| 51(H) | 0.0541 | 0.0497 | 0.0569 | 0.0028 | 0.0044 | 0.0036 | 0.0015 |
| 52(H) | 0.0465 | 0.0412 | 0.0569 | 0.0104 | 0.0053 | 0.0079 | -0.005 |
| 53(H) | 0.0466 | 0.0414 | 0.0565 | 0.0099 | 0.0052 | 0.0076 | -0.0047 |

**Table S5:** Fukui functions (local reactivity indices) include electrophilic (F⁻), nucleophilic (F⁺), and radical attacks (F⁰) on **2b (**Gas phase**)**. f f⁻ = _qk_(N) - _qk_(N−1); f⁺ = _qk_(N+1) - _qk_(N); and f⁰ = _qk_(N+1) - _qk_(N−1). qk represents the electron population at the k^th^ atom in a neutral (N), anionic (N+1), or cationic (N−1) species (derived from Hirshfeld charges). The condensed dual descriptor (CDD = f⁺ − f⁻) differentiates nucleophilic (CDD > 0) from electrophilic (CDD < 0) attack preferences at each atomic center.

| Atom | q(N) | q(N+1) | q(N-1) | f⁻ | f⁺ | f⁰ | CDD |
| --- | --- | --- | --- | --- | --- | --- | --- |
| 1(N) | -0.0256 | -0.0365 | -0.0122 | 0.0134 | 0.0109 | 0.0121 | -0.0025 |
| 2(C) | 0.1583 | 0.1112 | 0.1762 | 0.0179 | 0.0471 | 0.0325 | 0.0292 |
| 3(C) | 0.0071 | -0.0296 | 0.0536 | 0.0465 | 0.0367 | 0.0416 | -0.0098 |
| 4(N) | -0.1791 | -0.1908 | -0.1412 | 0.0379 | 0.0117 | 0.0248 | -0.0262 |
| 5(C) | 0.0812 | 0.0399 | 0.1097 | 0.0285 | 0.0413 | 0.0349 | 0.0128 |
| 6(C) | 0.0348 | 0.045 | 0.0261 | -0.0087 | -0.0102 | -0.0095 | -0.0015 |
| 7(C) | -0.0449 | -0.0476 | -0.0428 | 0.0021 | 0.0027 | 0.0024 | 0.0006 |
| 8(C) | -0.0362 | -0.0498 | -0.0232 | 0.0129 | 0.0136 | 0.0133 | 0.0007 |
| 9(C) | -0.0367 | -0.0561 | -0.0177 | 0.019 | 0.0194 | 0.0192 | 0.0005 |
| 10(C) | -0.0335 | -0.0461 | -0.0215 | 0.012 | 0.0126 | 0.0123 | 0.0006 |
| 11(C) | -0.036 | -0.0363 | -0.0343 | 0.0017 | 0.0002 | 0.001 | -0.0015 |
| 12(O) | -0.2415 | -0.3069 | -0.1945 | 0.047 | 0.0654 | 0.0562 | 0.0184 |
| 13(S) | 0.0505 | -0.0096 | 0.1459 | 0.0954 | 0.0601 | 0.0778 | -0.0353 |
| 14(C) | -0.0161 | -0.0946 | 0.009 | 0.025 | 0.0785 | 0.0518 | 0.0535 |
| 15(C) | -0.0182 | -0.0296 | 0.0129 | 0.0311 | 0.0114 | 0.0212 | -0.0197 |
| 16(C) | -0.0475 | -0.0805 | -0.0355 | 0.012 | 0.033 | 0.0225 | 0.021 |
| 17(C) | 0.0596 | 0.0412 | 0.1008 | 0.0412 | 0.0184 | 0.0298 | -0.0228 |
| 18(C) | -0.061 | -0.1124 | 0 | 0.061 | 0.0513 | 0.0562 | -0.0096 |
| 19(C) | -0.0735 | -0.1 | -0.0418 | 0.0317 | 0.0265 | 0.0291 | -0.0053 |
| 20(C) | 0.0663 | 0.0442 | 0.1175 | 0.0512 | 0.0221 | 0.0366 | -0.0291 |
| 21(C) | -0.0532 | -0.0642 | -0.0431 | 0.01 | 0.011 | 0.0105 | 0.0009 |
| 22(C) | 0.1443 | 0.1152 | 0.1472 | 0.0029 | 0.0291 | 0.016 | 0.0262 |
| 23(C) | -0.0236 | -0.0281 | -0.0253 | -0.0016 | 0.0044 | 0.0014 | 0.0061 |
| 24(O) | -0.2434 | -0.293 | -0.2173 | 0.0261 | 0.0495 | 0.0378 | 0.0234 |
| 25(C) | -0.0366 | -0.0381 | -0.0454 | -0.0089 | 0.0015 | -0.0037 | 0.0104 |
| 26(C) | -0.0401 | -0.0454 | -0.0414 | -0.0013 | 0.0053 | 0.002 | 0.0066 |
| 27(C) | -0.0299 | -0.0544 | -0.0188 | 0.0111 | 0.0245 | 0.0178 | 0.0134 |
| 28(C) | -0.0392 | -0.058 | -0.0258 | 0.0134 | 0.0188 | 0.0161 | 0.0055 |
| 29(C) | -0.0266 | -0.0441 | -0.0168 | 0.0098 | 0.0175 | 0.0137 | 0.0077 |
| 30(O) | -0.1119 | -0.1172 | -0.0671 | 0.0448 | 0.0053 | 0.025 | -0.0395 |
| 31(C) | -0.0014 | -0.0101 | 0.0138 | 0.0152 | 0.0087 | 0.0119 | -0.0064 |
| 32(O) | -0.1246 | -0.1329 | -0.0848 | 0.0398 | 0.0083 | 0.0241 | -0.0315 |
| 33(C) | -0.0048 | -0.0133 | 0.0084 | 0.0132 | 0.0085 | 0.0108 | -0.0048 |
| 34(H) | 0.0463 | 0.0431 | 0.0487 | 0.0024 | 0.0032 | 0.0028 | 0.0008 |
| 35(H) | 0.0495 | 0.0374 | 0.0606 | 0.0111 | 0.0121 | 0.0116 | 0.0009 |
| 36(H) | 0.0481 | 0.0337 | 0.0614 | 0.0133 | 0.0144 | 0.0138 | 0.0011 |
| 37(H) | 0.05 | 0.0388 | 0.0604 | 0.0104 | 0.0112 | 0.0108 | 0.0007 |
| 38(H) | 0.0527 | 0.0522 | 0.0535 | 0.0008 | 0.0005 | 0.0007 | -0.0004 |
| 39(H) | 0.0427 | 0.0136 | 0.0574 | 0.0147 | 0.029 | 0.0219 | 0.0143 |
| 40(H) | 0.0356 | 0.0256 | 0.0465 | 0.0108 | 0.0101 | 0.0104 | -0.0007 |
| 41(H) | 0.0423 | 0.0163 | 0.0701 | 0.0277 | 0.0261 | 0.0269 | -0.0017 |
| 42(H) | 0.0407 | 0.0217 | 0.063 | 0.0223 | 0.019 | 0.0206 | -0.0033 |
| 43(H) | 0.0531 | 0.0377 | 0.0668 | 0.0137 | 0.0154 | 0.0145 | 0.0018 |
| 44(H) | 0.0474 | 0.0407 | 0.0515 | 0.0041 | 0.0067 | 0.0054 | 0.0025 |
| 45(H) | 0.0308 | 0.0336 | 0.0234 | -0.0073 | -0.0028 | -0.0051 | 0.0045 |
| 46(H) | 0.0477 | 0.0448 | 0.0452 | -0.0025 | 0.0029 | 0.0002 | 0.0054 |
| 47(H) | 0.0475 | 0.0322 | 0.0584 | 0.011 | 0.0153 | 0.0131 | 0.0043 |
| 48(H) | 0.048 | 0.0353 | 0.0563 | 0.0084 | 0.0126 | 0.0105 | 0.0043 |
| 49(H) | 0.0516 | 0.0402 | 0.0691 | 0.0176 | 0.0113 | 0.0144 | -0.0062 |
| 50(H) | 0.0396 | 0.0289 | 0.0564 | 0.0168 | 0.0107 | 0.0138 | -0.0061 |
| 51(H) | 0.0392 | 0.0291 | 0.0559 | 0.0167 | 0.0101 | 0.0134 | -0.0066 |
| 52(H) | 0.0507 | 0.0378 | 0.0685 | 0.0178 | 0.0129 | 0.0153 | -0.0049 |
| 53(H) | 0.0361 | 0.0271 | 0.0501 | 0.014 | 0.009 | 0.0115 | -0.0051 |
| 54(H) | 0.0365 | 0.0272 | 0.0506 | 0.0141 | 0.0093 | 0.0117 | -0.0048 |
| 55(H) | 0.048 | 0.0326 | 0.0565 | 0.0085 | 0.0154 | 0.012 | 0.0069 |

**Table S6:** Fukui functions (local reactivity indices) include electrophilic (F⁻), nucleophilic (F⁺), and radical attacks (F⁰) on **2b (**DMSO phase**)**. f⁻ = _qk_(N) - _qk_(N−1); f⁺ = _qk_(N+1) - _qk_(N); and f⁰ = _qk_(N+1) - _qk_(N−1). qk represents the electron population at the k^th^ atom in a neutral (N), anionic (N+1), or cationic (N−1) species (derived from Hirshfeld charges). The condensed dual descriptor (CDD = f⁺ − f⁻) differentiates nucleophilic (CDD > 0) from electrophilic (CDD < 0) attack preferences at each atomic center.

| Atom | q(N) | q(N+1) | q(N-1) | f⁻ | f⁺ | f⁰ | CDD |
| --- | --- | --- | --- | --- | --- | --- | --- |
| 1(N) | -0.0203 | -0.0353 | -0.0108 | 0.0095 | 0.015 | 0.0123 | 0.0055 |
| 2(C) | 0.1532 | 0.0895 | 0.1734 | 0.0202 | 0.0637 | 0.0419 | 0.0435 |
| 3(C) | 0.004 | -0.0534 | 0.0498 | 0.0458 | 0.0574 | 0.0516 | 0.0116 |
| 4(N) | -0.2038 | -0.2378 | -0.1807 | 0.0231 | 0.034 | 0.0286 | 0.0109 |
| 5(C) | 0.0777 | 0.0238 | 0.102 | 0.0244 | 0.0539 | 0.0391 | 0.0295 |
| 6(C) | 0.0241 | 0.0252 | 0.0237 | -0.0004 | -0.0011 | -0.0008 | -0.0007 |
| 7(C) | -0.049 | -0.0554 | -0.0455 | 0.0035 | 0.0064 | 0.0049 | 0.0029 |
| 8(C) | -0.0422 | -0.0488 | -0.0386 | 0.0036 | 0.0066 | 0.0051 | 0.003 |
| 9(C) | -0.0424 | -0.0512 | -0.0373 | 0.0051 | 0.0088 | 0.0069 | 0.0037 |
| 10(C) | -0.0414 | -0.0479 | -0.0378 | 0.0035 | 0.0065 | 0.005 | 0.003 |
| 11(C) | -0.0495 | -0.055 | -0.0464 | 0.0031 | 0.0055 | 0.0043 | 0.0024 |
| 12(O) | -0.2743 | -0.3495 | -0.237 | 0.0374 | 0.0751 | 0.0562 | 0.0378 |
| 13(S) | 0.0173 | -0.035 | 0.0536 | 0.0362 | 0.0523 | 0.0443 | 0.0161 |
| 14(C) | -0.0103 | -0.1295 | 0.011 | 0.0212 | 0.1192 | 0.0702 | 0.098 |
| 15(C) | -0.0278 | -0.0553 | 0.025 | 0.0528 | 0.0275 | 0.0401 | -0.0253 |
| 16(C) | -0.0663 | -0.1167 | -0.0328 | 0.0335 | 0.0504 | 0.0419 | 0.0169 |
| 17(C) | 0.0495 | 0.0272 | 0.1192 | 0.0697 | 0.0223 | 0.046 | -0.0474 |
| 18(C) | -0.0536 | -0.1132 | 0.0198 | 0.0734 | 0.0596 | 0.0665 | -0.0138 |
| 19(C) | -0.0695 | -0.1 | -0.0143 | 0.0551 | 0.0305 | 0.0428 | -0.0246 |
| 20(C) | 0.0658 | 0.031 | 0.1416 | 0.0758 | 0.0348 | 0.0553 | -0.041 |
| 21(C) | -0.0447 | -0.0574 | -0.0389 | 0.0058 | 0.0128 | 0.0093 | 0.007 |
| 22(C) | 0.145 | 0.1377 | 0.1477 | 0.0027 | 0.0074 | 0.005 | 0.0047 |
| 23(C) | -0.0274 | -0.0278 | -0.0272 | 0.0002 | 0.0004 | 0.0003 | 0.0002 |
| 24(O) | -0.273 | -0.2869 | -0.2657 | 0.0073 | 0.0139 | 0.0106 | 0.0066 |
| 25(C) | -0.0345 | -0.034 | -0.0351 | -0.0007 | -0.0004 | -0.0005 | 0.0002 |
| 26(C) | -0.0394 | -0.0431 | -0.0379 | 0.0015 | 0.0037 | 0.0026 | 0.0021 |
| 27(C) | -0.025 | -0.0295 | -0.0226 | 0.0023 | 0.0045 | 0.0034 | 0.0022 |
| 28(C) | -0.034 | -0.0384 | -0.0314 | 0.0026 | 0.0045 | 0.0035 | 0.0019 |
| 29(C) | -0.0328 | -0.0372 | -0.0302 | 0.0026 | 0.0044 | 0.0035 | 0.0018 |
| 30(O) | -0.1154 | -0.1277 | -0.0487 | 0.0667 | 0.0123 | 0.0395 | -0.0543 |
| 31(C) | 0.0029 | -0.0041 | 0.0213 | 0.0184 | 0.007 | 0.0127 | -0.0113 |
| 32(O) | -0.1402 | -0.1483 | -0.0778 | 0.0625 | 0.0081 | 0.0353 | -0.0544 |
| 33(C) | -0.003 | -0.0082 | 0.0136 | 0.0165 | 0.0052 | 0.0109 | -0.0114 |
| 34(H) | 0.0606 | 0.0554 | 0.063 | 0.0024 | 0.0052 | 0.0038 | 0.0028 |
| 35(H) | 0.0598 | 0.0561 | 0.0619 | 0.0021 | 0.0037 | 0.0029 | 0.0016 |
| 36(H) | 0.0584 | 0.0543 | 0.0607 | 0.0023 | 0.0041 | 0.0032 | 0.0017 |
| 37(H) | 0.0594 | 0.0556 | 0.0615 | 0.0021 | 0.0037 | 0.0029 | 0.0017 |
| 38(H) | 0.0436 | 0.0398 | 0.0456 | 0.0019 | 0.0038 | 0.0029 | 0.0019 |
| 39(H) | 0.0446 | 0.001 | 0.0589 | 0.0142 | 0.0436 | 0.0289 | 0.0294 |
| 40(H) | 0.0248 | 0.0092 | 0.0402 | 0.0154 | 0.0156 | 0.0155 | 0.0002 |
| 41(H) | 0.0606 | 0.0372 | 0.0906 | 0.03 | 0.0234 | 0.0267 | -0.0066 |
| 42(H) | 0.0585 | 0.0432 | 0.0849 | 0.0264 | 0.0153 | 0.0209 | -0.0112 |
| 43(H) | 0.0659 | 0.0556 | 0.0712 | 0.0052 | 0.0104 | 0.0078 | 0.0051 |
| 44(H) | 0.0615 | 0.0524 | 0.0664 | 0.0049 | 0.0091 | 0.007 | 0.0042 |
| 45(H) | 0.0492 | 0.0462 | 0.0504 | 0.0013 | 0.003 | 0.0021 | 0.0018 |
| 46(H) | 0.055 | 0.0505 | 0.0568 | 0.0018 | 0.0045 | 0.0032 | 0.0028 |
| 47(H) | 0.0603 | 0.0577 | 0.0617 | 0.0015 | 0.0026 | 0.002 | 0.0011 |
| 48(H) | 0.0503 | 0.0474 | 0.052 | 0.0017 | 0.0029 | 0.0023 | 0.0012 |
| 49(H) | 0.0575 | 0.0518 | 0.0715 | 0.014 | 0.0057 | 0.0098 | -0.0083 |
| 50(H) | 0.0509 | 0.0442 | 0.0701 | 0.0193 | 0.0067 | 0.013 | -0.0126 |
| 51(H) | 0.0505 | 0.0439 | 0.0698 | 0.0193 | 0.0066 | 0.0129 | -0.0126 |
| 52(H) | 0.0545 | 0.05 | 0.0673 | 0.0129 | 0.0045 | 0.0087 | -0.0084 |
| 53(H) | 0.0472 | 0.0418 | 0.0648 | 0.0176 | 0.0054 | 0.0115 | -0.0122 |
| 54(H) | 0.0472 | 0.0418 | 0.0648 | 0.0176 | 0.0054 | 0.0115 | -0.0122 |
| 55(H) | 0.0612 | 0.0588 | 0.0623 | 0.0012 | 0.0024 | 0.0018 | 0.0012 |

**Table S7:** Fukui functions (local reactivity indices) include electrophilic (F⁻), nucleophilic (F⁺), and radical attacks (F⁰) on **2c (**Gas phase**)**. f⁻ = _qk_(N) - _qk_(N−1); f⁺ = _qk_(N+1) - _qk_(N); and f⁰ = _qk_(N+1) - _qk_(N−1). qk represents the electron population at the k^th^ atom in a neutral (N), anionic (N+1), or cationic (N−1) species (derived from Hirshfeld charges). The condensed dual descriptor (CDD = f⁺ − f⁻) differentiates nucleophilic (CDD > 0) from electrophilic (CDD < 0) attack preferences at each atomic center.

| Atom | q(N) | q(N+1) | q(N-1) | f⁻ | f⁺ | f⁰ | CDD |
| --- | --- | --- | --- | --- | --- | --- | --- |
| 1(N) | -0.0255 | -0.0359 | -0.0122 | 0.0134 | 0.0104 | 0.0119 | -0.003 |
| 2(C) | 0.1583 | 0.1145 | 0.1763 | 0.018 | 0.0438 | 0.0309 | 0.0257 |
| 3(C) | 0.0067 | -0.027 | 0.0533 | 0.0467 | 0.0336 | 0.0402 | -0.013 |
| 4(N) | -0.1789 | -0.1902 | -0.1422 | 0.0367 | 0.0113 | 0.024 | -0.0254 |
| 5(C) | 0.0807 | 0.0419 | 0.1092 | 0.0285 | 0.0389 | 0.0337 | 0.0104 |
| 6(C) | 0.0344 | 0.0444 | 0.0259 | -0.0086 | -0.01 | -0.0093 | -0.0014 |
| 7(C) | -0.045 | -0.0475 | -0.0428 | 0.0022 | 0.0025 | 0.0024 | 0.0003 |
| 8(C) | -0.0359 | -0.0492 | -0.023 | 0.0129 | 0.0133 | 0.0131 | 0.0004 |
| 9(C) | -0.0362 | -0.055 | -0.0173 | 0.0188 | 0.0188 | 0.0188 | 0 |
| 10(C) | -0.0329 | -0.0452 | -0.0211 | 0.0118 | 0.0122 | 0.012 | 0.0004 |
| 11(C) | -0.0358 | -0.0358 | -0.0342 | 0.0016 | 0 | 0.0008 | -0.0016 |
| 12(O) | -0.2406 | -0.3025 | -0.1938 | 0.0468 | 0.062 | 0.0544 | 0.0152 |
| 13(S) | 0.0509 | -0.0071 | 0.1436 | 0.0927 | 0.058 | 0.0753 | -0.0347 |
| 14(C) | -0.0155 | -0.0903 | 0.008 | 0.0234 | 0.0748 | 0.0491 | 0.0514 |
| 15(C) | -0.0184 | -0.0285 | 0.0133 | 0.0317 | 0.0101 | 0.0209 | -0.0216 |
| 16(C) | -0.047 | -0.0782 | -0.035 | 0.012 | 0.0312 | 0.0216 | 0.0192 |
| 17(C) | 0.0601 | 0.0426 | 0.1022 | 0.0421 | 0.0176 | 0.0298 | -0.0246 |
| 18(C) | -0.0601 | -0.1091 | 0.0009 | 0.061 | 0.049 | 0.055 | -0.012 |
| 19(C) | -0.073 | -0.0981 | -0.0409 | 0.0321 | 0.0251 | 0.0286 | -0.007 |
| 20(C) | 0.0666 | 0.0456 | 0.1185 | 0.0519 | 0.021 | 0.0365 | -0.0309 |
| 21(C) | -0.0529 | -0.0641 | -0.0431 | 0.0098 | 0.0112 | 0.0105 | 0.0013 |
| 22(C) | 0.1439 | 0.1096 | 0.1464 | 0.0025 | 0.0343 | 0.0184 | 0.0318 |
| 23(C) | -0.0243 | -0.0301 | -0.0264 | -0.0021 | 0.0059 | 0.0019 | 0.008 |
| 24(O) | -0.2424 | -0.297 | -0.2174 | 0.025 | 0.0546 | 0.0398 | 0.0296 |
| 25(C) | -0.0305 | -0.0345 | -0.0397 | -0.0092 | 0.0041 | -0.0026 | 0.0132 |
| 26(C) | -0.0451 | -0.0505 | -0.0481 | -0.0031 | 0.0054 | 0.0012 | 0.0085 |
| 27(C) | 0.0326 | 0.0117 | 0.0394 | 0.0068 | 0.0209 | 0.0138 | 0.014 |
| 28(C) | -0.0439 | -0.0621 | -0.0321 | 0.0118 | 0.0183 | 0.015 | 0.0064 |
| 29(C) | -0.0199 | -0.0389 | -0.0107 | 0.0091 | 0.019 | 0.0141 | 0.0099 |
| 30(O) | -0.1122 | -0.1171 | -0.0662 | 0.0459 | 0.0049 | 0.0254 | -0.0411 |
| 31(C) | -0.0013 | -0.0096 | 0.014 | 0.0154 | 0.0083 | 0.0118 | -0.0071 |
| 32(O) | -0.1243 | -0.1323 | -0.083 | 0.0413 | 0.008 | 0.0246 | -0.0333 |
| 33(C) | -0.0046 | -0.0127 | 0.0089 | 0.0135 | 0.0081 | 0.0108 | -0.0054 |
| 34(H) | 0.0464 | 0.0432 | 0.0489 | 0.0025 | 0.0032 | 0.0028 | 0.0007 |
| 35(H) | 0.0497 | 0.0379 | 0.0608 | 0.0111 | 0.0118 | 0.0114 | 0.0007 |
| 36(H) | 0.0484 | 0.0344 | 0.0616 | 0.0132 | 0.014 | 0.0136 | 0.0008 |
| 37(H) | 0.0505 | 0.0397 | 0.0607 | 0.0102 | 0.0108 | 0.0105 | 0.0005 |
| 38(H) | 0.0531 | 0.0528 | 0.0538 | 0.0007 | 0.0002 | 0.0005 | -0.0005 |
| 39(H) | 0.0426 | 0.0153 | 0.057 | 0.0144 | 0.0273 | 0.0209 | 0.0128 |
| 40(H) | 0.0359 | 0.0264 | 0.0469 | 0.0109 | 0.0095 | 0.0102 | -0.0014 |
| 41(H) | 0.0428 | 0.0179 | 0.0705 | 0.0278 | 0.0249 | 0.0263 | -0.0029 |
| 42(H) | 0.0411 | 0.0229 | 0.0635 | 0.0224 | 0.0181 | 0.0203 | -0.0043 |
| 43(H) | 0.0543 | 0.0384 | 0.0676 | 0.0133 | 0.0158 | 0.0146 | 0.0025 |
| 44(H) | 0.0477 | 0.0404 | 0.0518 | 0.0041 | 0.0073 | 0.0057 | 0.0033 |
| 45(H) | 0.034 | 0.0355 | 0.0267 | -0.0073 | -0.0015 | -0.0044 | 0.0057 |
| 46(H) | 0.0533 | 0.0502 | 0.0495 | -0.0038 | 0.0031 | -0.0003 | 0.0069 |
| 47(H) | 0.0532 | 0.0383 | 0.0631 | 0.0099 | 0.0149 | 0.0124 | 0.005 |
| 48(H) | 0.0527 | 0.0394 | 0.0607 | 0.008 | 0.0133 | 0.0107 | 0.0053 |
| 49(H) | 0.0516 | 0.0409 | 0.0694 | 0.0178 | 0.0107 | 0.0142 | -0.0071 |
| 50(H) | 0.0398 | 0.0294 | 0.0568 | 0.017 | 0.0104 | 0.0137 | -0.0065 |
| 51(H) | 0.0396 | 0.0301 | 0.0564 | 0.0168 | 0.0096 | 0.0132 | -0.0072 |
| 52(H) | 0.051 | 0.0386 | 0.069 | 0.018 | 0.0124 | 0.0152 | -0.0057 |
| 53(H) | 0.0364 | 0.028 | 0.0507 | 0.0143 | 0.0085 | 0.0114 | -0.0058 |
| 54(H) | 0.0365 | 0.0275 | 0.051 | 0.0144 | 0.009 | 0.0117 | -0.0054 |
| 55(Cl) | -0.0477 | -0.0878 | -0.0262 | 0.0215 | 0.04 | 0.0308 | 0.0186 |

**Table S8:** Fukui functions (local reactivity indices) include electrophilic (F⁻), nucleophilic (F⁺), and radical attacks (F⁰) on **2c (**DMSO phase**)**. f⁻ = _qk_(N) - _qk_(N−1); f⁺ = _qk_(N+1) - _qk_(N); and f⁰ = _qk_(N+1) - _qk_(N−1). qk represents the electron population at the k^th^ atom in a neutral (N), anionic (N+1), or cationic (N−1) species (derived from Hirshfeld charges). The condensed dual descriptor (CDD = f⁺ − f⁻) differentiates nucleophilic (CDD > 0) from electrophilic (CDD < 0) attack preferences at each atomic center.

| Atom | q(N) | q(N+1) | q(N-1) | f⁻ | f⁺ | f⁰ | CDD |
| --- | --- | --- | --- | --- | --- | --- | --- |
| 1(N) | -0.0204 | -0.0357 | -0.0103 | 0.0101 | 0.0152 | 0.0127 | 0.0051 |
| 2(C) | 0.1521 | 0.0881 | 0.1731 | 0.021 | 0.064 | 0.0425 | 0.043 |
| 3(C) | -0.001 | -0.0577 | 0.0459 | 0.0469 | 0.0567 | 0.0518 | 0.0098 |
| 4(N) | -0.2051 | -0.24 | -0.181 | 0.0241 | 0.0349 | 0.0295 | 0.0108 |
| 5(C) | 0.0738 | 0.0164 | 0.1005 | 0.0267 | 0.0574 | 0.0421 | 0.0307 |
| 6(C) | 0.0256 | 0.0263 | 0.0255 | -0.0001 | -0.0007 | -0.0004 | -0.0006 |
| 7(C) | -0.0506 | -0.0573 | -0.0467 | 0.0039 | 0.0067 | 0.0053 | 0.0028 |
| 8(C) | -0.0422 | -0.0493 | -0.0382 | 0.004 | 0.0071 | 0.0056 | 0.003 |
| 9(C) | -0.0438 | -0.0532 | -0.038 | 0.0058 | 0.0094 | 0.0076 | 0.0036 |
| 10(C) | -0.0416 | -0.0486 | -0.0376 | 0.004 | 0.007 | 0.0055 | 0.003 |
| 11(C) | -0.0465 | -0.0525 | -0.0429 | 0.0036 | 0.0061 | 0.0048 | 0.0025 |
| 12(O) | -0.2752 | -0.351 | -0.2362 | 0.039 | 0.0758 | 0.0574 | 0.0368 |
| 13(S) | 0.0137 | -0.0407 | 0.0513 | 0.0376 | 0.0545 | 0.046 | 0.0168 |
| 14(C) | -0.0088 | -0.1206 | 0.0096 | 0.0184 | 0.1118 | 0.0651 | 0.0934 |
| 15(C) | -0.0271 | -0.0491 | 0.0193 | 0.0464 | 0.022 | 0.0342 | -0.0243 |
| 16(C) | -0.0651 | -0.1133 | -0.034 | 0.0311 | 0.0483 | 0.0397 | 0.0172 |
| 17(C) | 0.0488 | 0.0269 | 0.1158 | 0.067 | 0.0219 | 0.0444 | -0.0451 |
| 18(C) | -0.0546 | -0.1131 | 0.0161 | 0.0707 | 0.0585 | 0.0646 | -0.0122 |
| 19(C) | -0.0694 | -0.0988 | -0.0178 | 0.0515 | 0.0295 | 0.0405 | -0.0221 |
| 20(C) | 0.0665 | 0.0366 | 0.134 | 0.0675 | 0.0299 | 0.0487 | -0.0376 |
| 21(C) | -0.0443 | -0.0576 | -0.0382 | 0.0062 | 0.0133 | 0.0097 | 0.0071 |
| 22(C) | 0.1495 | 0.1459 | 0.1518 | 0.0023 | 0.0036 | 0.0029 | 0.0013 |
| 23(C) | -0.0216 | -0.0225 | -0.0204 | 0.0012 | 0.0009 | 0.001 | -0.0002 |
| 24(O) | -0.2729 | -0.2835 | -0.265 | 0.0078 | 0.0107 | 0.0092 | 0.0028 |
| 25(C) | -0.0194 | -0.0243 | -0.0166 | 0.0028 | 0.0049 | 0.0039 | 0.0021 |
| 26(C) | -0.0412 | -0.0428 | -0.041 | 0.0001 | 0.0016 | 0.0009 | 0.0015 |
| 27(C) | 0.0274 | 0.0251 | 0.0283 | 0.0009 | 0.0024 | 0.0016 | 0.0015 |
| 28(C) | -0.0432 | -0.0493 | -0.0387 | 0.0045 | 0.0062 | 0.0053 | 0.0017 |
| 29(C) | -0.0235 | -0.0301 | -0.0194 | 0.0041 | 0.0066 | 0.0053 | 0.0024 |
| 30(O) | -0.1103 | -0.1207 | -0.0471 | 0.0632 | 0.0105 | 0.0368 | -0.0527 |
| 31(C) | 0.003 | -0.004 | 0.0208 | 0.0178 | 0.007 | 0.0124 | -0.0108 |
| 32(O) | -0.1407 | -0.1492 | -0.0794 | 0.0613 | 0.0086 | 0.0349 | -0.0527 |
| 33(C) | -0.0035 | -0.0089 | 0.0129 | 0.0164 | 0.0054 | 0.0109 | -0.011 |
| 34(H) | 0.0594 | 0.0538 | 0.062 | 0.0026 | 0.0056 | 0.0041 | 0.003 |
| 35(H) | 0.06 | 0.056 | 0.0623 | 0.0023 | 0.004 | 0.0032 | 0.0017 |
| 36(H) | 0.0581 | 0.0538 | 0.0608 | 0.0026 | 0.0044 | 0.0035 | 0.0017 |
| 37(H) | 0.0596 | 0.0557 | 0.062 | 0.0023 | 0.004 | 0.0031 | 0.0017 |
| 38(H) | 0.0575 | 0.053 | 0.0599 | 0.0024 | 0.0045 | 0.0034 | 0.0021 |
| 39(H) | 0.0446 | 0.0032 | 0.0585 | 0.0139 | 0.0414 | 0.0276 | 0.0274 |
| 40(H) | 0.0267 | 0.0102 | 0.0431 | 0.0164 | 0.0166 | 0.0165 | 0.0001 |
| 41(H) | 0.0595 | 0.0354 | 0.0898 | 0.0304 | 0.0241 | 0.0272 | -0.0063 |
| 42(H) | 0.0575 | 0.0413 | 0.0846 | 0.0271 | 0.0162 | 0.0216 | -0.0109 |
| 43(H) | 0.0704 | 0.0587 | 0.0765 | 0.0061 | 0.0116 | 0.0089 | 0.0055 |
| 44(H) | 0.0586 | 0.0494 | 0.0641 | 0.0054 | 0.0092 | 0.0073 | 0.0038 |
| 45(H) | 0.0671 | 0.062 | 0.0702 | 0.0031 | 0.0051 | 0.0041 | 0.002 |
| 46(H) | 0.059 | 0.056 | 0.0605 | 0.0015 | 0.003 | 0.0023 | 0.0015 |
| 47(H) | 0.0641 | 0.0599 | 0.0672 | 0.0031 | 0.0042 | 0.0037 | 0.0011 |
| 48(H) | 0.0574 | 0.053 | 0.0602 | 0.0028 | 0.0043 | 0.0036 | 0.0015 |
| 49(H) | 0.0574 | 0.0514 | 0.0715 | 0.0142 | 0.006 | 0.0101 | -0.0082 |
| 50(H) | 0.0504 | 0.0433 | 0.0701 | 0.0197 | 0.0071 | 0.0134 | -0.0126 |
| 51(H) | 0.0484 | 0.0415 | 0.067 | 0.0186 | 0.0068 | 0.0127 | -0.0117 |
| 52(H) | 0.0542 | 0.0495 | 0.0671 | 0.0129 | 0.0047 | 0.0088 | -0.0082 |
| 53(H) | 0.0467 | 0.041 | 0.0642 | 0.0175 | 0.0057 | 0.0116 | -0.0118 |
| 54(H) | 0.0467 | 0.041 | 0.0642 | 0.0175 | 0.0057 | 0.0116 | -0.0118 |
| 55(Cl) | -0.0508 | -0.0593 | -0.0411 | 0.0097 | 0.0085 | 0.0091 | -0.0012 |

**Table S9:** Fukui functions (local reactivity indices) include electrophilic (F⁻), nucleophilic (F⁺), and radical attacks (F⁰) on **2d (**Gas phase**)**. f⁻ = _qk_(N) - _qk_(N−1); f⁺ = _qk_(N+1) - _qk_(N); and f⁰ = _qk_(N+1) - _qk_(N−1). qk represents the electron population at the k^th^ atom in a neutral (N), anionic (N+1), or cationic (N−1) species (derived from Hirshfeld charges). The condensed dual descriptor (CDD = f⁺ − f⁻) differentiates nucleophilic (CDD > 0) from electrophilic (CDD < 0) attack preferences at each atomic center.

| Atom | q(N) | q(N+1) | q(N-1) | f⁻ | f⁺ | f⁰ | CDD |
| --- | --- | --- | --- | --- | --- | --- | --- |
| 1(N) | -0.0255 | -0.0357 | -0.0122 | 0.0133 | 0.0102 | 0.0117 | -0.0031 |
| 2(C) | 0.1584 | 0.1158 | 0.1764 | 0.018 | 0.0426 | 0.0303 | 0.0246 |
| 3(C) | 0.0066 | -0.026 | 0.0533 | 0.0468 | 0.0326 | 0.0397 | -0.0141 |
| 4(N) | -0.1787 | -0.1896 | -0.1421 | 0.0366 | 0.0109 | 0.0237 | -0.0257 |
| 5(C) | 0.0808 | 0.0431 | 0.1093 | 0.0285 | 0.0377 | 0.0331 | 0.0092 |
| 6(C) | 0.0344 | 0.0443 | 0.0258 | -0.0086 | -0.0099 | -0.0092 | -0.0013 |
| 7(C) | -0.045 | -0.0474 | -0.0428 | 0.0022 | 0.0024 | 0.0023 | 0.0002 |
| 8(C) | -0.0359 | -0.049 | -0.0231 | 0.0128 | 0.0131 | 0.013 | 0.0002 |
| 9(C) | -0.0361 | -0.0546 | -0.0173 | 0.0187 | 0.0186 | 0.0186 | -0.0002 |
| 10(C) | -0.0329 | -0.0449 | -0.0211 | 0.0118 | 0.012 | 0.0119 | 0.0003 |
| 11(C) | -0.0358 | -0.0358 | -0.0342 | 0.0016 | 0 | 0.0008 | -0.0017 |
| 12(O) | -0.2405 | -0.3011 | -0.1939 | 0.0466 | 0.0606 | 0.0536 | 0.014 |
| 13(S) | 0.0513 | -0.0055 | 0.1439 | 0.0926 | 0.0569 | 0.0747 | -0.0357 |
| 14(C) | -0.0154 | -0.0887 | 0.0079 | 0.0233 | 0.0732 | 0.0483 | 0.0499 |
| 15(C) | -0.0183 | -0.0281 | 0.0134 | 0.0318 | 0.0098 | 0.0208 | -0.022 |
| 16(C) | -0.0469 | -0.0774 | -0.0349 | 0.012 | 0.0305 | 0.0212 | 0.0185 |
| 17(C) | 0.0601 | 0.0429 | 0.1022 | 0.0421 | 0.0172 | 0.0297 | -0.0249 |
| 18(C) | -0.06 | -0.1081 | 0.0009 | 0.0609 | 0.0481 | 0.0545 | -0.0128 |
| 19(C) | -0.0729 | -0.0976 | -0.0408 | 0.0321 | 0.0247 | 0.0284 | -0.0075 |
| 20(C) | 0.0667 | 0.0462 | 0.1187 | 0.0519 | 0.0206 | 0.0363 | -0.0314 |
| 21(C) | -0.053 | -0.064 | -0.0432 | 0.0097 | 0.011 | 0.0104 | 0.0013 |
| 22(C) | 0.1443 | 0.1083 | 0.1466 | 0.0023 | 0.036 | 0.0191 | 0.0337 |
| 23(C) | -0.023 | -0.0296 | -0.0253 | -0.0023 | 0.0066 | 0.0021 | 0.0089 |
| 24(O) | -0.2416 | -0.2977 | -0.2171 | 0.0245 | 0.0561 | 0.0403 | 0.0317 |
| 25(C) | -0.0303 | -0.0353 | -0.0397 | -0.0094 | 0.005 | -0.0022 | 0.0144 |
| 26(C) | -0.0434 | -0.0494 | -0.0467 | -0.0033 | 0.006 | 0.0013 | 0.0093 |
| 27(C) | 0.0128 | -0.0078 | 0.0187 | 0.0059 | 0.0206 | 0.0133 | 0.0147 |
| 28(C) | -0.0422 | -0.0604 | -0.0308 | 0.0114 | 0.0182 | 0.0148 | 0.0068 |
| 29(C) | -0.0198 | -0.0394 | -0.0109 | 0.0089 | 0.0196 | 0.0143 | 0.0107 |
| 30(O) | -0.1121 | -0.1168 | -0.0661 | 0.046 | 0.0047 | 0.0254 | -0.0413 |
| 31(C) | -0.0012 | -0.0093 | 0.0142 | 0.0154 | 0.0081 | 0.0117 | -0.0073 |
| 32(O) | -0.1243 | -0.1322 | -0.083 | 0.0413 | 0.0079 | 0.0246 | -0.0334 |
| 33(C) | -0.0046 | -0.0125 | 0.0089 | 0.0135 | 0.008 | 0.0107 | -0.0055 |
| 34(H) | 0.0463 | 0.0432 | 0.0488 | 0.0024 | 0.0031 | 0.0028 | 0.0007 |
| 35(H) | 0.0497 | 0.0381 | 0.0608 | 0.011 | 0.0116 | 0.0113 | 0.0006 |
| 36(H) | 0.0485 | 0.0346 | 0.0616 | 0.0131 | 0.0138 | 0.0135 | 0.0007 |
| 37(H) | 0.0505 | 0.0399 | 0.0607 | 0.0102 | 0.0106 | 0.0104 | 0.0004 |
| 38(H) | 0.0531 | 0.0529 | 0.0538 | 0.0007 | 0.0002 | 0.0004 | -0.0005 |
| 39(H) | 0.0426 | 0.016 | 0.0571 | 0.0144 | 0.0266 | 0.0205 | 0.0122 |
| 40(H) | 0.036 | 0.0266 | 0.0469 | 0.0109 | 0.0093 | 0.0101 | -0.0016 |
| 41(H) | 0.0428 | 0.0183 | 0.0705 | 0.0277 | 0.0244 | 0.0261 | -0.0033 |
| 42(H) | 0.0411 | 0.0233 | 0.0635 | 0.0224 | 0.0178 | 0.0201 | -0.0046 |
| 43(H) | 0.0542 | 0.0384 | 0.0675 | 0.0133 | 0.0158 | 0.0145 | 0.0026 |
| 44(H) | 0.0477 | 0.0403 | 0.0516 | 0.004 | 0.0074 | 0.0057 | 0.0034 |
| 45(H) | 0.0339 | 0.0351 | 0.0264 | -0.0075 | -0.0012 | -0.0043 | 0.0062 |
| 46(H) | 0.0527 | 0.0493 | 0.0486 | -0.004 | 0.0033 | -0.0004 | 0.0074 |
| 47(H) | 0.0526 | 0.0379 | 0.0621 | 0.0096 | 0.0147 | 0.0121 | 0.0051 |
| 48(H) | 0.0528 | 0.0393 | 0.0606 | 0.0078 | 0.0135 | 0.0107 | 0.0057 |
| 49(H) | 0.0516 | 0.0412 | 0.0694 | 0.0178 | 0.0104 | 0.0141 | -0.0074 |
| 50(H) | 0.0398 | 0.0295 | 0.0568 | 0.017 | 0.0103 | 0.0136 | -0.0067 |
| 51(H) | 0.0397 | 0.0303 | 0.0564 | 0.0168 | 0.0093 | 0.0131 | -0.0074 |
| 52(H) | 0.051 | 0.0388 | 0.069 | 0.018 | 0.0122 | 0.0151 | -0.0059 |
| 53(H) | 0.0364 | 0.0282 | 0.0507 | 0.0143 | 0.0083 | 0.0113 | -0.006 |
| 54(H) | 0.0365 | 0.0277 | 0.0509 | 0.0144 | 0.0089 | 0.0116 | -0.0056 |
| 55(Br) | -0.034 | -0.0838 | -0.0077 | 0.0264 | 0.0498 | 0.0381 | 0.0234 |

**Table S10:** Fukui functions (local reactivity indices) include electrophilic (F⁻), nucleophilic (F⁺), and radical attacks (F⁰) on **2d (**DMSO phase**)**. f⁻ = _qk_(N) - _qk_(N−1); f⁺ = _qk_(N+1) - _qk_(N); and f⁰ = _qk_(N+1) - _qk_(N−1). qk represents the electron population at the k^th^ atom in a neutral (N), anionic (N+1), or cationic (N−1) species (derived from Hirshfeld charges). The condensed dual descriptor (CDD = f⁺ − f⁻) differentiates nucleophilic (CDD > 0) from electrophilic (CDD < 0) attack preferences at each atomic center.

| Atom | q(N) | q(N+1) | q(N-1) | f⁻ | f⁺ | f⁰ | CDD |
| --- | --- | --- | --- | --- | --- | --- | --- |
| 1(N) | -0.0205 | -0.0356 | -0.0102 | 0.0103 | 0.0151 | 0.0127 | 0.0048 |
| 2(C) | 0.1506 | 0.0881 | 0.1719 | 0.0212 | 0.0625 | 0.0419 | 0.0413 |
| 3(C) | -0.0018 | -0.0574 | 0.0453 | 0.0471 | 0.0555 | 0.0513 | 0.0084 |
| 4(N) | -0.2057 | -0.2416 | -0.1822 | 0.0235 | 0.036 | 0.0297 | 0.0125 |
| 5(C) | 0.0719 | 0.0143 | 0.0993 | 0.0274 | 0.0576 | 0.0425 | 0.0302 |
| 6(C) | 0.0254 | 0.0262 | 0.0252 | -0.0002 | -0.0008 | -0.0005 | -0.0006 |
| 7(C) | -0.0502 | -0.057 | -0.0463 | 0.0039 | 0.0067 | 0.0053 | 0.0028 |
| 8(C) | -0.0423 | -0.0493 | -0.0383 | 0.004 | 0.007 | 0.0055 | 0.003 |
| 9(C) | -0.0437 | -0.053 | -0.0379 | 0.0057 | 0.0093 | 0.0075 | 0.0036 |
| 10(C) | -0.0418 | -0.0487 | -0.0378 | 0.004 | 0.007 | 0.0055 | 0.003 |
| 11(C) | -0.0473 | -0.0535 | -0.0437 | 0.0036 | 0.0061 | 0.0049 | 0.0025 |
| 12(O) | -0.276 | -0.3517 | -0.2356 | 0.0403 | 0.0758 | 0.058 | 0.0354 |
| 13(S) | 0.0073 | -0.0463 | 0.043 | 0.0357 | 0.0537 | 0.0447 | 0.018 |
| 14(C) | -0.0085 | -0.1208 | 0.009 | 0.0175 | 0.1123 | 0.0649 | 0.0949 |
| 15(C) | -0.0275 | -0.0488 | 0.0182 | 0.0458 | 0.0213 | 0.0335 | -0.0245 |
| 16(C) | -0.0636 | -0.1105 | -0.0344 | 0.0292 | 0.0468 | 0.038 | 0.0176 |
| 17(C) | 0.0507 | 0.0305 | 0.1147 | 0.0639 | 0.0202 | 0.0421 | -0.0438 |
| 18(C) | -0.0516 | -0.1097 | 0.0151 | 0.0666 | 0.0581 | 0.0624 | -0.0085 |
| 19(C) | -0.0682 | -0.0974 | -0.0166 | 0.0516 | 0.0292 | 0.0404 | -0.0224 |
| 20(C) | 0.0677 | 0.0365 | 0.1355 | 0.0678 | 0.0312 | 0.0495 | -0.0365 |
| 21(C) | -0.0452 | -0.0584 | -0.039 | 0.0062 | 0.0132 | 0.0097 | 0.007 |
| 22(C) | 0.1494 | 0.1466 | 0.1514 | 0.002 | 0.0028 | 0.0024 | 0.0007 |
| 23(C) | -0.0213 | -0.0229 | -0.0196 | 0.0017 | 0.0016 | 0.0017 | -0.0001 |
| 24(O) | -0.2706 | -0.2805 | -0.2631 | 0.0075 | 0.01 | 0.0087 | 0.0025 |
| 25(C) | -0.0188 | -0.0245 | -0.0152 | 0.0035 | 0.0057 | 0.0046 | 0.0022 |
| 26(C) | -0.0397 | -0.0431 | -0.0379 | 0.0018 | 0.0034 | 0.0026 | 0.0015 |
| 27(C) | 0.0101 | 0.0097 | 0.0092 | -0.0009 | 0.0003 | -0.0003 | 0.0012 |
| 28(C) | -0.0419 | -0.0476 | -0.0377 | 0.0042 | 0.0057 | 0.005 | 0.0015 |
| 29(C) | -0.0226 | -0.0281 | -0.0187 | 0.004 | 0.0055 | 0.0047 | 0.0015 |
| 30(O) | -0.1137 | -0.1259 | -0.0476 | 0.0661 | 0.0122 | 0.0391 | -0.0538 |
| 31(C) | 0.0024 | -0.005 | 0.021 | 0.0186 | 0.0074 | 0.013 | -0.0111 |
| 32(O) | -0.1405 | -0.1492 | -0.0778 | 0.0627 | 0.0087 | 0.0357 | -0.054 |
| 33(C) | -0.0038 | -0.0095 | 0.013 | 0.0169 | 0.0057 | 0.0113 | -0.0112 |
| 34(H) | 0.0599 | 0.0542 | 0.0625 | 0.0026 | 0.0056 | 0.0041 | 0.003 |
| 35(H) | 0.0598 | 0.0559 | 0.0622 | 0.0023 | 0.004 | 0.0031 | 0.0016 |
| 36(H) | 0.0581 | 0.0538 | 0.0607 | 0.0026 | 0.0043 | 0.0035 | 0.0017 |
| 37(H) | 0.0596 | 0.0556 | 0.0619 | 0.0023 | 0.004 | 0.0031 | 0.0016 |
| 38(H) | 0.0578 | 0.0533 | 0.0602 | 0.0024 | 0.0045 | 0.0035 | 0.0021 |
| 39(H) | 0.0432 | 0.0013 | 0.0573 | 0.014 | 0.0419 | 0.028 | 0.0279 |
| 40(H) | 0.0241 | 0.008 | 0.0403 | 0.0162 | 0.0162 | 0.0162 | 0 |
| 41(H) | 0.0592 | 0.0341 | 0.09 | 0.0308 | 0.0251 | 0.0279 | -0.0058 |
| 42(H) | 0.057 | 0.0401 | 0.0851 | 0.0281 | 0.0169 | 0.0225 | -0.0112 |
| 43(H) | 0.071 | 0.0587 | 0.0774 | 0.0064 | 0.0123 | 0.0094 | 0.0058 |
| 44(H) | 0.0582 | 0.0493 | 0.0634 | 0.0052 | 0.0088 | 0.007 | 0.0036 |
| 45(H) | 0.0669 | 0.0616 | 0.0702 | 0.0034 | 0.0053 | 0.0043 | 0.0019 |
| 46(H) | 0.0617 | 0.0577 | 0.0643 | 0.0026 | 0.004 | 0.0033 | 0.0014 |
| 47(H) | 0.0619 | 0.0576 | 0.0651 | 0.0032 | 0.0043 | 0.0038 | 0.0011 |
| 48(H) | 0.0579 | 0.0536 | 0.061 | 0.003 | 0.0043 | 0.0037 | 0.0013 |
| 49(H) | 0.0569 | 0.0505 | 0.0716 | 0.0147 | 0.0064 | 0.0105 | -0.0084 |
| 50(H) | 0.0501 | 0.0425 | 0.0704 | 0.0203 | 0.0076 | 0.0139 | -0.0127 |
| 51(H) | 0.0489 | 0.0419 | 0.0681 | 0.0192 | 0.007 | 0.0131 | -0.0122 |
| 52(H) | 0.0538 | 0.0488 | 0.0673 | 0.0135 | 0.005 | 0.0093 | -0.0085 |
| 53(H) | 0.0461 | 0.0402 | 0.0641 | 0.0179 | 0.0059 | 0.0119 | -0.012 |
| 54(H) | 0.0464 | 0.0404 | 0.0647 | 0.0183 | 0.0061 | 0.0122 | -0.0122 |
| 55(Br) | -0.0259 | -0.0336 | -0.0185 | 0.0074 | 0.0077 | 0.0075 | 0.0003 |

**Table S11:** Fukui functions (local reactivity indices) include electrophilic (F⁻), nucleophilic (F⁺), and radical attacks (F⁰) on **2e (**Gas phase**)**. f⁻ = _qk_(N) - _qk_(N−1); f⁺ = _qk_(N+1) - _qk_(N); and f⁰ = _qk_(N+1) - _qk_(N−1). qk represents the electron population at the k^th^ atom in a neutral (N), anionic (N+1), or cationic (N−1) species (derived from Hirshfeld charges). The condensed dual descriptor (CDD = f⁺ − f⁻) differentiates nucleophilic (CDD > 0) from electrophilic (CDD < 0) attack preferences at each atomic center.

| Atom | q(N) | q(N+1) | q(N-1) | f⁻ | f⁺ | f⁰ | CDD |
| --- | --- | --- | --- | --- | --- | --- | --- |
| 1(N) | -0.0272 | -0.0356 | -0.0134 | 0.0138 | 0.0084 | 0.0111 | -0.0054 |
| 2(C) | 0.1578 | 0.1238 | 0.176 | 0.0182 | 0.034 | 0.0261 | 0.0158 |
| 3(C) | 0.0057 | -0.0189 | 0.0534 | 0.0478 | 0.0246 | 0.0362 | -0.0231 |
| 4(N) | -0.1827 | -0.1874 | -0.1454 | 0.0373 | 0.0048 | 0.021 | -0.0326 |
| 5(C) | 0.0787 | 0.0527 | 0.1093 | 0.0306 | 0.026 | 0.0283 | -0.0046 |
| 6(C) | 0.0339 | 0.0422 | 0.0249 | -0.009 | -0.0083 | -0.0087 | 0.0007 |
| 7(C) | -0.0447 | -0.0437 | -0.0431 | 0.0016 | -0.001 | 0.0003 | -0.0027 |
| 8(C) | -0.037 | -0.0466 | -0.0243 | 0.0127 | 0.0095 | 0.0111 | -0.0032 |
| 9(C) | -0.037 | -0.0536 | -0.0183 | 0.0187 | 0.0166 | 0.0176 | -0.0021 |
| 10(C) | -0.0346 | -0.0468 | -0.0223 | 0.0123 | 0.0123 | 0.0123 | 0 |
| 11(C) | -0.0372 | -0.04 | -0.0352 | 0.002 | 0.0028 | 0.0024 | 0.0007 |
| 12(O) | -0.2421 | -0.2915 | -0.1943 | 0.0478 | 0.0494 | 0.0486 | 0.0015 |
| 13(S) | 0.0358 | -0.0152 | 0.128 | 0.0922 | 0.0511 | 0.0716 | -0.0411 |
| 14(C) | -0.0156 | -0.0716 | 0.0081 | 0.0237 | 0.056 | 0.0398 | 0.0322 |
| 15(C) | -0.0179 | -0.0251 | 0.0143 | 0.0322 | 0.0072 | 0.0197 | -0.025 |
| 16(C) | -0.0468 | -0.0711 | -0.0346 | 0.0122 | 0.0243 | 0.0182 | 0.0121 |
| 17(C) | 0.06 | 0.0456 | 0.1028 | 0.0428 | 0.0144 | 0.0286 | -0.0284 |
| 18(C) | -0.0604 | -0.0998 | 0.0015 | 0.062 | 0.0394 | 0.0507 | -0.0226 |
| 19(C) | -0.0731 | -0.0933 | -0.0405 | 0.0326 | 0.0201 | 0.0264 | -0.0124 |
| 20(C) | 0.0666 | 0.051 | 0.1193 | 0.0528 | 0.0156 | 0.0342 | -0.0372 |
| 21(C) | -0.0409 | -0.0499 | -0.0324 | 0.0085 | 0.009 | 0.0087 | 0.0005 |
| 22(C) | 0.009 | -0.0178 | 0.0021 | -0.0069 | 0.0268 | 0.01 | 0.0337 |
| 23(C) | -0.0322 | -0.0463 | -0.0378 | -0.0057 | 0.0141 | 0.0042 | 0.0198 |
| 24(C) | -0.029 | -0.0505 | -0.0267 | 0.0023 | 0.0215 | 0.0119 | 0.0191 |
| 25(C) | 0.0171 | 0.0001 | 0.0268 | 0.0097 | 0.017 | 0.0134 | 0.0073 |
| 26(C) | -0.0324 | -0.0608 | -0.0211 | 0.0113 | 0.0283 | 0.0198 | 0.017 |
| 27(C) | -0.0374 | -0.0581 | -0.0312 | 0.0062 | 0.0208 | 0.0135 | 0.0146 |
| 28(O) | -0.1128 | -0.1152 | -0.0663 | 0.0465 | 0.0024 | 0.0245 | -0.0441 |
| 29(C) | -0.0015 | -0.0077 | 0.0141 | 0.0156 | 0.0062 | 0.0109 | -0.0094 |
| 30(O) | -0.1244 | -0.1311 | -0.0825 | 0.0419 | 0.0067 | 0.0243 | -0.0352 |
| 31(C) | -0.0047 | -0.0114 | 0.009 | 0.0137 | 0.0067 | 0.0102 | -0.007 |
| 32(N) | 0.2508 | 0.2015 | 0.2537 | 0.0028 | 0.0493 | 0.0261 | 0.0465 |
| 33(O) | -0.206 | -0.289 | -0.1884 | 0.0176 | 0.083 | 0.0503 | 0.0655 |
| 34(O) | -0.2043 | -0.283 | -0.195 | 0.0094 | 0.0786 | 0.044 | 0.0693 |
| 35(H) | 0.0462 | 0.0481 | 0.0482 | 0.0019 | -0.0018 | 0.0001 | -0.0038 |
| 36(H) | 0.0492 | 0.0409 | 0.06 | 0.0108 | 0.0083 | 0.0095 | -0.0025 |
| 37(H) | 0.048 | 0.0357 | 0.0612 | 0.0132 | 0.0123 | 0.0128 | -0.0008 |
| 38(H) | 0.0498 | 0.0392 | 0.0604 | 0.0106 | 0.0106 | 0.0106 | 0.0001 |
| 39(H) | 0.0524 | 0.0498 | 0.0535 | 0.0011 | 0.0026 | 0.0018 | 0.0015 |
| 40(H) | 0.0416 | 0.0223 | 0.0561 | 0.0146 | 0.0192 | 0.0169 | 0.0047 |
| 41(H) | 0.0359 | 0.0283 | 0.047 | 0.0111 | 0.0076 | 0.0093 | -0.0036 |
| 42(H) | 0.0426 | 0.0223 | 0.0707 | 0.0282 | 0.0203 | 0.0242 | -0.0079 |
| 43(H) | 0.041 | 0.0263 | 0.0637 | 0.0227 | 0.0146 | 0.0187 | -0.0081 |
| 44(H) | 0.0527 | 0.0379 | 0.0655 | 0.0128 | 0.0148 | 0.0138 | 0.002 |
| 45(H) | 0.0498 | 0.0413 | 0.055 | 0.0053 | 0.0085 | 0.0069 | 0.0032 |
| 46(H) | 0.045 | 0.0376 | 0.0391 | -0.0059 | 0.0075 | 0.0008 | 0.0134 |
| 47(H) | 0.0554 | 0.0416 | 0.0573 | 0.0018 | 0.0138 | 0.0078 | 0.012 |
| 48(H) | 0.0545 | 0.0359 | 0.0636 | 0.009 | 0.0186 | 0.0138 | 0.0096 |
| 49(H) | 0.0495 | 0.0327 | 0.0554 | 0.0059 | 0.0168 | 0.0113 | 0.0109 |
| 50(H) | 0.0511 | 0.0437 | 0.0691 | 0.018 | 0.0073 | 0.0127 | -0.0107 |
| 51(H) | 0.0395 | 0.0304 | 0.0567 | 0.0172 | 0.0091 | 0.0132 | -0.0081 |
| 52(H) | 0.0399 | 0.0326 | 0.0569 | 0.017 | 0.0073 | 0.0121 | -0.0097 |
| 53(H) | 0.0508 | 0.0404 | 0.0691 | 0.0183 | 0.0103 | 0.0143 | -0.008 |
| 54(H) | 0.0366 | 0.0298 | 0.0511 | 0.0144 | 0.0068 | 0.0106 | -0.0076 |
| 55(H) | 0.0361 | 0.0285 | 0.0508 | 0.0147 | 0.0076 | 0.0111 | -0.0071 |

**Table S12:** Fukui functions (local reactivity indices) include electrophilic (F⁻), nucleophilic (F⁺), and radical attacks (F⁰) on **2e (**DMSO phase**)**. f⁻ = _qk_(N) - _qk_(N−1); f⁺ = _qk_(N+1) - _qk_(N); and f⁰ = _qk_(N+1) - _qk_(N−1). qk represents the electron population at the k^th^ atom in a neutral (N), anionic (N+1), or cationic (N−1) species (derived from Hirshfeld charges). The condensed dual descriptor (CDD = f⁺ − f⁻) differentiates nucleophilic (CDD > 0) from electrophilic (CDD < 0) attack preferences at each atomic center.

| Atom | q(N) | q(N+1) | q(N-1) | f⁻ | f⁺ | f⁰ | CDD |
| --- | --- | --- | --- | --- | --- | --- | --- |
| 1(N) | -0.0227 | -0.0249 | -0.0132 | 0.0095 | 0.0021 | 0.0058 | -0.0074 |
| 2(C) | 0.1549 | 0.1499 | 0.1766 | 0.0217 | 0.005 | 0.0134 | -0.0166 |
| 3(C) | -0.0002 | -0.0043 | 0.0516 | 0.0518 | 0.0041 | 0.028 | -0.0477 |
| 4(N) | -0.2023 | -0.2063 | -0.1721 | 0.0302 | 0.004 | 0.0171 | -0.0262 |
| 5(C) | 0.0781 | 0.0735 | 0.1046 | 0.0265 | 0.0046 | 0.0156 | -0.0218 |
| 6(C) | 0.0248 | 0.0252 | 0.0241 | -0.0007 | -0.0003 | -0.0005 | 0.0004 |
| 7(C) | -0.045 | -0.0458 | -0.0417 | 0.0033 | 0.0008 | 0.0021 | -0.0025 |
| 8(C) | -0.0399 | -0.0411 | -0.0365 | 0.0034 | 0.0013 | 0.0023 | -0.0021 |
| 9(C) | -0.0395 | -0.0412 | -0.0347 | 0.0048 | 0.0018 | 0.0033 | -0.003 |
| 10(C) | -0.0402 | -0.0418 | -0.0368 | 0.0034 | 0.0016 | 0.0025 | -0.0018 |
| 11(C) | -0.0464 | -0.0477 | -0.0432 | 0.0032 | 0.0013 | 0.0023 | -0.0019 |
| 12(O) | -0.2775 | -0.284 | -0.2404 | 0.037 | 0.0065 | 0.0218 | -0.0305 |
| 13(S) | 0.0294 | 0.0054 | 0.0818 | 0.0524 | 0.024 | 0.0382 | -0.0284 |
| 14(C) | -0.0243 | -0.035 | 0.0035 | 0.0278 | 0.0107 | 0.0192 | -0.017 |
| 15(C) | -0.0305 | -0.0324 | 0.0223 | 0.0528 | 0.0019 | 0.0273 | -0.0508 |
| 16(C) | -0.0703 | -0.0743 | -0.0405 | 0.0298 | 0.0039 | 0.0169 | -0.0259 |
| 17(C) | 0.048 | 0.0461 | 0.1129 | 0.0649 | 0.0019 | 0.0334 | -0.063 |
| 18(C) | -0.058 | -0.0631 | 0.0169 | 0.0749 | 0.0051 | 0.04 | -0.0698 |
| 19(C) | -0.0713 | -0.0738 | -0.02 | 0.0513 | 0.0025 | 0.0269 | -0.0488 |
| 20(C) | 0.0623 | 0.0595 | 0.1364 | 0.0741 | 0.0029 | 0.0385 | -0.0712 |
| 21(C) | -0.0316 | -0.0435 | -0.0264 | 0.0052 | 0.0119 | 0.0085 | 0.0068 |
| 22(C) | 0.0148 | -0.0447 | 0.0144 | -0.0004 | 0.0595 | 0.0295 | 0.0598 |
| 23(C) | -0.0314 | -0.0685 | -0.0295 | 0.0019 | 0.0371 | 0.0195 | 0.0351 |
| 24(C) | -0.0306 | -0.0938 | -0.0287 | 0.0019 | 0.0632 | 0.0326 | 0.0612 |
| 25(C) | 0.0133 | -0.0274 | 0.0154 | 0.0021 | 0.0407 | 0.0214 | 0.0386 |
| 26(C) | -0.0313 | -0.0905 | -0.0289 | 0.0025 | 0.0591 | 0.0308 | 0.0567 |
| 27(C) | -0.0326 | -0.0707 | -0.0303 | 0.0023 | 0.0381 | 0.0202 | 0.0358 |
| 28(O) | -0.1172 | -0.118 | -0.0542 | 0.063 | 0.0009 | 0.0319 | -0.0621 |
| 29(C) | 0.0021 | 0.0015 | 0.0197 | 0.0176 | 0.0006 | 0.0091 | -0.017 |
| 30(O) | -0.1409 | -0.1417 | -0.0856 | 0.0553 | 0.0007 | 0.028 | -0.0546 |
| 31(C) | -0.0035 | -0.004 | 0.0114 | 0.0149 | 0.0005 | 0.0077 | -0.0144 |
| 32(N) | 0.2495 | 0.1321 | 0.2502 | 0.0007 | 0.1174 | 0.059 | 0.1167 |
| 33(O) | -0.2277 | -0.3958 | -0.2258 | 0.0019 | 0.1681 | 0.085 | 0.1662 |
| 34(O) | -0.2277 | -0.3955 | -0.2263 | 0.0014 | 0.1678 | 0.0846 | 0.1664 |
| 35(H) | 0.0618 | 0.0614 | 0.0642 | 0.0023 | 0.0005 | 0.0014 | -0.0018 |
| 36(H) | 0.0608 | 0.0601 | 0.0628 | 0.002 | 0.0007 | 0.0013 | -0.0013 |
| 37(H) | 0.0596 | 0.0587 | 0.0618 | 0.0022 | 0.0009 | 0.0016 | -0.0013 |
| 38(H) | 0.0608 | 0.0599 | 0.0628 | 0.002 | 0.0009 | 0.0015 | -0.0011 |
| 39(H) | 0.0606 | 0.0592 | 0.0632 | 0.0025 | 0.0015 | 0.002 | -0.001 |
| 40(H) | 0.0428 | 0.0388 | 0.0581 | 0.0153 | 0.004 | 0.0097 | -0.0113 |
| 41(H) | 0.0235 | 0.0222 | 0.0381 | 0.0146 | 0.0013 | 0.008 | -0.0133 |
| 42(H) | 0.0592 | 0.0571 | 0.0889 | 0.0297 | 0.0021 | 0.0159 | -0.0277 |
| 43(H) | 0.0575 | 0.0561 | 0.0826 | 0.0252 | 0.0014 | 0.0133 | -0.0238 |
| 44(H) | 0.0727 | 0.0586 | 0.078 | 0.0053 | 0.0141 | 0.0097 | 0.0088 |
| 45(H) | 0.0588 | 0.0491 | 0.0648 | 0.0059 | 0.0098 | 0.0079 | 0.0038 |
| 46(H) | 0.0576 | 0.0375 | 0.0599 | 0.0022 | 0.0202 | 0.0112 | 0.0179 |
| 47(H) | 0.0612 | 0.0271 | 0.0622 | 0.001 | 0.034 | 0.0175 | 0.033 |
| 48(H) | 0.0614 | 0.0288 | 0.0628 | 0.0013 | 0.0326 | 0.017 | 0.0313 |
| 49(H) | 0.0635 | 0.0423 | 0.0657 | 0.0022 | 0.0212 | 0.0117 | 0.019 |
| 50(H) | 0.0569 | 0.0565 | 0.0702 | 0.0133 | 0.0004 | 0.0068 | -0.0129 |
| 51(H) | 0.05 | 0.0492 | 0.0684 | 0.0184 | 0.0008 | 0.0096 | -0.0176 |
| 52(H) | 0.05 | 0.0495 | 0.0684 | 0.0184 | 0.0005 | 0.0094 | -0.0179 |
| 53(H) | 0.0542 | 0.0537 | 0.066 | 0.0118 | 0.0005 | 0.0061 | -0.0114 |
| 54(H) | 0.0467 | 0.0463 | 0.0626 | 0.0159 | 0.0004 | 0.0082 | -0.0155 |
| 55(H) | 0.0468 | 0.0462 | 0.0627 | 0.0159 | 0.0006 | 0.0082 | -0.0153 |

**Table S13:** Fukui functions (local reactivity indices) include electrophilic (F⁻), nucleophilic (F⁺), and radical attacks (F⁰) on **2f (**Gas phase**)**. f⁻ = _qk_(N) - _qk_(N−1); f⁺ = _qk_(N+1) - _qk_(N); and f⁰ = _qk_(N+1) - _qk_(N−1). qk represents the electron population at the k^th^ atom in a neutral (N), anionic (N+1), or cationic (N−1) species (derived from Hirshfeld charges). The condensed dual descriptor (CDD = f⁺ − f⁻) differentiates nucleophilic (CDD > 0) from electrophilic (CDD < 0) attack preferences at each atomic center.

| Atom | q(N) | q(N+1) | q(N-1) | f⁻ | f⁺ | f⁰ | CDD |
| --- | --- | --- | --- | --- | --- | --- | --- |
| 1(N) | -0.0268 | -0.0395 | -0.0083 | 0.0185 | 0.0127 | 0.0156 | -0.0057 |
| 2(C) | 0.1565 | 0.0946 | 0.1769 | 0.0204 | 0.0619 | 0.0412 | 0.0415 |
| 3(C) | 0.0098 | -0.0384 | 0.056 | 0.0462 | 0.0482 | 0.0472 | 0.002 |
| 4(N) | -0.1726 | -0.1826 | -0.1339 | 0.0388 | 0.01 | 0.0244 | -0.0288 |
| 5(C) | 0.0882 | 0.0345 | 0.1167 | 0.0284 | 0.0538 | 0.0411 | 0.0254 |
| 6(C) | 0.0348 | 0.0456 | 0.0275 | -0.0073 | -0.0107 | -0.009 | -0.0034 |
| 7(C) | -0.0391 | -0.0422 | -0.0337 | 0.0055 | 0.0031 | 0.0043 | -0.0024 |
| 8(C) | -0.0351 | -0.0508 | -0.0197 | 0.0154 | 0.0158 | 0.0156 | 0.0004 |
| 9(C) | -0.0395 | -0.063 | -0.0157 | 0.0238 | 0.0235 | 0.0236 | -0.0002 |
| 10(C) | -0.0372 | -0.0526 | -0.0222 | 0.015 | 0.0155 | 0.0152 | 0.0005 |
| 11(C) | -0.0465 | -0.0496 | -0.043 | 0.0035 | 0.0031 | 0.0033 | -0.0004 |
| 12(O) | -0.27 | -0.3597 | -0.2022 | 0.0678 | 0.0896 | 0.0787 | 0.0219 |
| 13(S) | 0.0488 | -0.0336 | 0.1465 | 0.0978 | 0.0824 | 0.0901 | -0.0154 |
| 14(C) | -0.0167 | -0.107 | 0.0219 | 0.0387 | 0.0902 | 0.0645 | 0.0516 |
| 15(C) | -0.0123 | -0.0295 | 0.0179 | 0.0301 | 0.0172 | 0.0237 | -0.0129 |
| 16(C) | -0.0461 | -0.0827 | -0.0337 | 0.0125 | 0.0365 | 0.0245 | 0.024 |
| 17(C) | 0.0563 | 0.0358 | 0.0932 | 0.0368 | 0.0205 | 0.0287 | -0.0164 |
| 18(C) | -0.0622 | -0.1196 | -0.0031 | 0.0591 | 0.0574 | 0.0582 | -0.0017 |
| 19(C) | -0.0713 | -0.1027 | -0.0406 | 0.0307 | 0.0314 | 0.031 | 0.0007 |
| 20(C) | 0.0678 | 0.0438 | 0.1143 | 0.0465 | 0.0239 | 0.0352 | -0.0226 |
| 21(C) | -0.052 | -0.062 | -0.043 | 0.009 | 0.0101 | 0.0095 | 0.0011 |
| 22(O) | -0.1132 | -0.1191 | -0.0705 | 0.0427 | 0.0059 | 0.0243 | -0.0368 |
| 23(C) | -0.0014 | -0.0116 | 0.0138 | 0.0152 | 0.0102 | 0.0127 | -0.005 |
| 24(O) | -0.0952 | -0.1022 | -0.0702 | 0.025 | 0.007 | 0.016 | -0.018 |
| 25(C) | 0.0003 | -0.0088 | 0.0112 | 0.0109 | 0.0091 | 0.01 | -0.0018 |
| 26(C) | 0.1986 | 0.1955 | 0.2017 | 0.0031 | 0.0031 | 0.0031 | -0.0001 |
| 27(O) | -0.2778 | -0.2881 | -0.2653 | 0.0125 | 0.0103 | 0.0114 | -0.0022 |
| 28(H) | 0.0513 | 0.0484 | 0.0547 | 0.0033 | 0.003 | 0.0032 | -0.0004 |
| 29(H) | 0.0492 | 0.0356 | 0.0621 | 0.0129 | 0.0136 | 0.0132 | 0.0007 |
| 30(H) | 0.0471 | 0.0303 | 0.0626 | 0.0155 | 0.0167 | 0.0161 | 0.0012 |
| 31(H) | 0.0491 | 0.0357 | 0.0615 | 0.0124 | 0.0134 | 0.0129 | 0.001 |
| 32(H) | 0.0473 | 0.0444 | 0.0498 | 0.0025 | 0.0029 | 0.0027 | 0.0004 |
| 33(H) | 0.0513 | 0.0163 | 0.0714 | 0.0202 | 0.035 | 0.0276 | 0.0148 |
| 34(H) | 0.036 | 0.0245 | 0.0446 | 0.0087 | 0.0114 | 0.01 | 0.0028 |
| 35(H) | 0.0432 | 0.0135 | 0.0707 | 0.0275 | 0.0297 | 0.0286 | 0.0022 |
| 36(H) | 0.0413 | 0.0193 | 0.0636 | 0.0222 | 0.0221 | 0.0221 | -0.0002 |
| 37(H) | 0.0522 | 0.0432 | 0.0613 | 0.0091 | 0.009 | 0.0091 | 0 |
| 38(H) | 0.0617 | 0.0436 | 0.0783 | 0.0166 | 0.018 | 0.0173 | 0.0014 |
| 39(H) | 0.0526 | 0.0391 | 0.07 | 0.0175 | 0.0134 | 0.0155 | -0.004 |
| 40(H) | 0.0392 | 0.0273 | 0.0559 | 0.0167 | 0.0119 | 0.0143 | -0.0048 |
| 41(H) | 0.0391 | 0.0272 | 0.0559 | 0.0168 | 0.0118 | 0.0143 | -0.005 |
| 42(H) | 0.0535 | 0.0399 | 0.0693 | 0.0157 | 0.0137 | 0.0147 | -0.002 |
| 43(H) | 0.0427 | 0.0325 | 0.0547 | 0.012 | 0.0101 | 0.0111 | -0.0019 |
| 44(H) | 0.0404 | 0.0307 | 0.0521 | 0.0117 | 0.0097 | 0.0107 | -0.002 |
| 45(O) | -0.175 | -0.1832 | -0.1667 | 0.0083 | 0.0082 | 0.0082 | -0.0001 |
| 46(H) | 0.1328 | 0.1285 | 0.1367 | 0.0039 | 0.0043 | 0.0041 | 0.0004 |

**Table S14:** Fukui functions (local reactivity indices) include electrophilic (F⁻), nucleophilic (F⁺), and radical attacks (F⁰) on **2f (**DMSO phase**)**. f⁻ = _qk_(N) - _qk_(N−1); f⁺ = _qk_(N+1) - _qk_(N); and f⁰ = _qk_(N+1) - _qk_(N−1). qk represents the electron population at the k^th^ atom in a neutral (N), anionic (N+1), or cationic (N−1) species (derived from Hirshfeld charges). The condensed dual descriptor (CDD = f⁺ − f⁻) differentiates nucleophilic (CDD > 0) from electrophilic (CDD < 0) attack preferences at each atomic center.

| Atom | q(N) | q(N+1) | q(N-1) | f⁻ | f⁺ | f⁰ | CDD |
| --- | --- | --- | --- | --- | --- | --- | --- |
| 1(N) | -0.0227 | -0.0405 | -0.0122 | 0.0105 | 0.0178 | 0.0142 | 0.0073 |
| 2(C) | 0.1524 | 0.0733 | 0.175 | 0.0227 | 0.0791 | 0.0509 | 0.0564 |
| 3(C) | 0.0024 | -0.0665 | 0.0544 | 0.052 | 0.0689 | 0.0604 | 0.0169 |
| 4(N) | -0.1858 | -0.211 | -0.1585 | 0.0273 | 0.0252 | 0.0262 | -0.0021 |
| 5(C) | 0.0884 | 0.0309 | 0.1138 | 0.0254 | 0.0574 | 0.0414 | 0.032 |
| 6(C) | 0.0248 | 0.0259 | 0.0242 | -0.0006 | -0.0011 | -0.0008 | -0.0005 |
| 7(C) | -0.0452 | -0.052 | -0.0415 | 0.0037 | 0.0068 | 0.0053 | 0.0031 |
| 8(C) | -0.0398 | -0.0462 | -0.0362 | 0.0036 | 0.0064 | 0.005 | 0.0028 |
| 9(C) | -0.0394 | -0.0484 | -0.0343 | 0.005 | 0.0091 | 0.0071 | 0.004 |
| 10(C) | -0.0399 | -0.0462 | -0.0362 | 0.0036 | 0.0063 | 0.005 | 0.0027 |
| 11(C) | -0.0463 | -0.0528 | -0.0429 | 0.0034 | 0.0065 | 0.0049 | 0.0031 |
| 12(O) | -0.3107 | -0.4017 | -0.2678 | 0.0429 | 0.091 | 0.0669 | 0.0481 |
| 13(S) | 0.041 | -0.0288 | 0.0993 | 0.0582 | 0.0698 | 0.064 | 0.0116 |
| 14(C) | -0.0239 | -0.1471 | 0.0099 | 0.0338 | 0.1232 | 0.0785 | 0.0894 |
| 15(C) | -0.0275 | -0.0564 | 0.0289 | 0.0564 | 0.0289 | 0.0426 | -0.0275 |
| 16(C) | -0.0649 | -0.1087 | -0.0387 | 0.0262 | 0.0439 | 0.035 | 0.0177 |
| 17(C) | 0.0487 | 0.029 | 0.1117 | 0.063 | 0.0197 | 0.0414 | -0.0432 |
| 18(C) | -0.0571 | -0.111 | 0.0173 | 0.0744 | 0.0539 | 0.0641 | -0.0205 |
| 19(C) | -0.0694 | -0.0988 | -0.0184 | 0.051 | 0.0294 | 0.0402 | -0.0216 |
| 20(C) | 0.0615 | 0.0306 | 0.1349 | 0.0733 | 0.0309 | 0.0521 | -0.0425 |
| 21(C) | -0.0463 | -0.0551 | -0.0402 | 0.0061 | 0.0087 | 0.0074 | 0.0026 |
| 22(O) | -0.1219 | -0.133 | -0.056 | 0.0659 | 0.0111 | 0.0385 | -0.0548 |
| 23(C) | 0.0013 | -0.0053 | 0.0196 | 0.0183 | 0.0067 | 0.0125 | -0.0116 |
| 24(O) | -0.1006 | -0.1061 | -0.0608 | 0.0399 | 0.0055 | 0.0227 | -0.0344 |
| 25(C) | 0.0048 | 0 | 0.0171 | 0.0123 | 0.0048 | 0.0086 | -0.0075 |
| 26(C) | 0.2004 | 0.1977 | 0.2029 | 0.0025 | 0.0027 | 0.0026 | 0.0001 |
| 27(O) | -0.3056 | -0.313 | -0.2983 | 0.0073 | 0.0073 | 0.0073 | 0 |
| 28(H) | 0.062 | 0.0567 | 0.0645 | 0.0026 | 0.0052 | 0.0039 | 0.0027 |
| 29(H) | 0.0609 | 0.0571 | 0.063 | 0.0021 | 0.0037 | 0.0029 | 0.0016 |
| 30(H) | 0.0597 | 0.0556 | 0.062 | 0.0023 | 0.0041 | 0.0032 | 0.0018 |
| 31(H) | 0.0611 | 0.0574 | 0.0632 | 0.0021 | 0.0037 | 0.0029 | 0.0016 |
| 32(H) | 0.0605 | 0.0556 | 0.0632 | 0.0027 | 0.005 | 0.0038 | 0.0023 |
| 33(H) | 0.0507 | 0.003 | 0.0701 | 0.0194 | 0.0477 | 0.0335 | 0.0283 |
| 34(H) | 0.0291 | 0.0152 | 0.0423 | 0.0132 | 0.0139 | 0.0136 | 0.0007 |
| 35(H) | 0.0617 | 0.0401 | 0.0912 | 0.0295 | 0.0217 | 0.0256 | -0.0078 |
| 36(H) | 0.058 | 0.0432 | 0.0832 | 0.0252 | 0.0148 | 0.02 | -0.0105 |
| 37(H) | 0.0602 | 0.0503 | 0.0678 | 0.0077 | 0.0099 | 0.0088 | 0.0023 |
| 38(H) | 0.082 | 0.0717 | 0.0895 | 0.0075 | 0.0102 | 0.0089 | 0.0027 |
| 39(H) | 0.0568 | 0.0514 | 0.0705 | 0.0137 | 0.0054 | 0.0096 | -0.0083 |
| 40(H) | 0.0499 | 0.0436 | 0.0689 | 0.019 | 0.0064 | 0.0127 | -0.0127 |
| 41(H) | 0.0496 | 0.0434 | 0.0685 | 0.019 | 0.0061 | 0.0125 | -0.0128 |
| 42(H) | 0.0601 | 0.0559 | 0.0704 | 0.0103 | 0.0042 | 0.0072 | -0.0061 |
| 43(H) | 0.0531 | 0.048 | 0.0665 | 0.0134 | 0.0051 | 0.0093 | -0.0084 |
| 44(H) | 0.052 | 0.0471 | 0.0655 | 0.0135 | 0.005 | 0.0092 | -0.0086 |
| 45(O) | -0.1763 | -0.1814 | -0.171 | 0.0053 | 0.0052 | 0.0052 | -0.0001 |
| 46(H) | 0.1309 | 0.1283 | 0.1341 | 0.0031 | 0.0026 | 0.0029 | -0.0005 |

**Table S15:** Fukui functions (local reactivity indices) include electrophilic (F⁻), nucleophilic (F⁺), and radical attacks (F⁰) on **2g (**Gas phase**)**. f⁻ = _qk_(N) - _qk_(N−1); f⁺ = _qk_(N+1) - _qk_(N); and f⁰ = _qk_(N+1) - _qk_(N−1). qk represents the electron population at the k^th^ atom in a neutral (N), anionic (N+1), or cationic (N−1) species (derived from Hirshfeld charges). The condensed dual descriptor (CDD = f⁺ − f⁻) differentiates nucleophilic (CDD > 0) from electrophilic (CDD < 0) attack preferences at each atomic center.

| Atom | q(N) | q(N+1) | q(N-1) | f⁻ | f⁺ | f⁰ | CDD |
| --- | --- | --- | --- | --- | --- | --- | --- |
| 1(N) | -0.0268 | -0.0392 | -0.0104 | 0.0163 | 0.0125 | 0.0144 | -0.0039 |
| 2(C) | 0.1546 | 0.0971 | 0.1749 | 0.0204 | 0.0575 | 0.0389 | 0.0371 |
| 3(C) | 0.0065 | -0.0394 | 0.055 | 0.0485 | 0.0459 | 0.0472 | -0.0027 |
| 4(N) | -0.175 | -0.1863 | -0.1411 | 0.034 | 0.0113 | 0.0226 | -0.0227 |
| 5(C) | 0.0885 | 0.0329 | 0.1168 | 0.0283 | 0.0556 | 0.0419 | 0.0273 |
| 6(C) | 0.0347 | 0.0459 | 0.0261 | -0.0087 | -0.0111 | -0.0099 | -0.0024 |
| 7(C) | -0.0386 | -0.0411 | -0.0347 | 0.0039 | 0.0025 | 0.0032 | -0.0014 |
| 8(C) | -0.0358 | -0.0509 | -0.0219 | 0.0139 | 0.015 | 0.0145 | 0.0011 |
| 9(C) | -0.0391 | -0.0613 | -0.0182 | 0.0209 | 0.0222 | 0.0216 | 0.0013 |
| 10(C) | -0.0373 | -0.0521 | -0.0236 | 0.0137 | 0.0148 | 0.0143 | 0.0011 |
| 11(C) | -0.0445 | -0.0469 | -0.0424 | 0.0021 | 0.0024 | 0.0023 | 0.0003 |
| 12(O) | -0.2722 | -0.3577 | -0.2072 | 0.0651 | 0.0855 | 0.0753 | 0.0204 |
| 13(S) | 0.0512 | -0.0312 | 0.1437 | 0.0925 | 0.0825 | 0.0875 | -0.01 |
| 14(C) | -0.0218 | -0.1072 | 0.0096 | 0.0315 | 0.0854 | 0.0584 | 0.0539 |
| 15(C) | -0.0154 | -0.0345 | 0.0159 | 0.0312 | 0.0192 | 0.0252 | -0.0121 |
| 16(C) | -0.048 | -0.0896 | -0.035 | 0.013 | 0.0416 | 0.0273 | 0.0286 |
| 17(C) | 0.0571 | 0.0362 | 0.098 | 0.0409 | 0.021 | 0.0309 | -0.0199 |
| 18(C) | -0.0648 | -0.1243 | -0.0028 | 0.062 | 0.0596 | 0.0608 | -0.0024 |
| 19(C) | -0.0729 | -0.1055 | -0.0406 | 0.0323 | 0.0326 | 0.0324 | 0.0003 |
| 20(C) | 0.0671 | 0.0425 | 0.1182 | 0.0511 | 0.0246 | 0.0379 | -0.0265 |
| 21(C) | -0.0532 | -0.0625 | -0.0456 | 0.0076 | 0.0092 | 0.0084 | 0.0017 |
| 22(O) | -0.1103 | -0.1161 | -0.0647 | 0.0457 | 0.0058 | 0.0257 | -0.0399 |
| 23(C) | -0.0012 | -0.0113 | 0.0144 | 0.0156 | 0.01 | 0.0128 | -0.0056 |
| 24(O) | -0.1152 | -0.1238 | -0.0806 | 0.0346 | 0.0086 | 0.0216 | -0.026 |
| 25(C) | -0.0047 | -0.014 | 0.0076 | 0.0123 | 0.0093 | 0.0108 | -0.0031 |
| 26(C) | 0.1974 | 0.1972 | 0.1977 | 0.0002 | 0.0003 | 0.0002 | 0 |
| 27(O) | -0.2664 | -0.2766 | -0.2545 | 0.0119 | 0.0102 | 0.011 | -0.0017 |
| 28(H) | 0.0517 | 0.0493 | 0.0541 | 0.0024 | 0.0025 | 0.0024 | 0.0001 |
| 29(H) | 0.0489 | 0.0358 | 0.0607 | 0.0118 | 0.0131 | 0.0124 | 0.0012 |
| 30(H) | 0.0468 | 0.0308 | 0.0611 | 0.0143 | 0.016 | 0.0151 | 0.0018 |
| 31(H) | 0.0489 | 0.036 | 0.0603 | 0.0115 | 0.0129 | 0.0122 | 0.0014 |
| 32(H) | 0.0487 | 0.0461 | 0.0505 | 0.0018 | 0.0026 | 0.0022 | 0.0007 |
| 33(H) | 0.0453 | 0.0134 | 0.0616 | 0.0163 | 0.0319 | 0.0241 | 0.0156 |
| 34(H) | 0.0356 | 0.0211 | 0.0461 | 0.0105 | 0.0145 | 0.0125 | 0.0039 |
| 35(H) | 0.0412 | 0.011 | 0.0694 | 0.0283 | 0.0301 | 0.0292 | 0.0019 |
| 36(H) | 0.0406 | 0.0184 | 0.0634 | 0.0228 | 0.0222 | 0.0225 | -0.0006 |
| 37(H) | 0.0525 | 0.0457 | 0.0586 | 0.0061 | 0.0068 | 0.0064 | 0.0007 |
| 38(H) | 0.0614 | 0.0442 | 0.0765 | 0.0151 | 0.0172 | 0.0161 | 0.0021 |
| 39(H) | 0.0526 | 0.0393 | 0.0706 | 0.018 | 0.0133 | 0.0157 | -0.0047 |
| 40(H) | 0.0387 | 0.0269 | 0.056 | 0.0173 | 0.0118 | 0.0146 | -0.0055 |
| 41(H) | 0.0388 | 0.0269 | 0.056 | 0.0173 | 0.0118 | 0.0145 | -0.0055 |
| 42(H) | 0.0496 | 0.0355 | 0.0668 | 0.0172 | 0.0141 | 0.0157 | -0.0031 |
| 43(H) | 0.0357 | 0.0255 | 0.0492 | 0.0135 | 0.0102 | 0.0119 | -0.0033 |
| 44(H) | 0.0381 | 0.0279 | 0.0514 | 0.0134 | 0.0102 | 0.0118 | -0.0032 |
| 45(O) | -0.1025 | -0.1028 | -0.1032 | -0.0006 | 0.0003 | -0.0002 | 0.0009 |
| 46(C) | 0.027 | 0.0265 | 0.027 | 0.0001 | 0.0004 | 0.0002 | 0.0004 |
| 47(H) | 0.0398 | 0.0414 | 0.0387 | -0.0011 | -0.0016 | -0.0013 | -0.0005 |
| 48(H) | 0.0328 | 0.0337 | 0.0299 | -0.0029 | -0.0008 | -0.0019 | 0.0021 |
| 49(C) | -0.0959 | -0.101 | -0.0908 | 0.0051 | 0.0051 | 0.0051 | 0.0001 |
| 50(H) | 0.0335 | 0.0263 | 0.0402 | 0.0067 | 0.0072 | 0.007 | 0.0005 |
| 51(H) | 0.0359 | 0.0301 | 0.0424 | 0.0065 | 0.0058 | 0.0062 | -0.0007 |
| 52(H) | 0.0412 | 0.0329 | 0.0491 | 0.0078 | 0.0083 | 0.0081 | 0.0005 |

**Table S16:** Fukui functions (local reactivity indices) include electrophilic (F⁻), nucleophilic (F⁺), and radical attacks (F⁰) on **2g (**DMSO phase**)**. f⁻ = _qk_(N) - _qk_(N−1); f⁺ = _qk_(N+1) - _qk_(N); and f⁰ = _qk_(N+1) - _qk_(N−1). qk represents the electron population at the k^th^ atom in a neutral (N), anionic (N+1), or cationic (N−1) species (derived from Hirshfeld charges). The condensed dual descriptor (CDD = f⁺ − f⁻) differentiates nucleophilic (CDD > 0) from electrophilic (CDD < 0) attack preferences at each atomic center.

| Atom | q(N) | q(N+1) | q(N-1) | f⁻ | f⁺ | f⁰ | CDD |
| --- | --- | --- | --- | --- | --- | --- | --- |
| 1(N) | -0.0232 | -0.0397 | -0.0137 | 0.0094 | 0.0165 | 0.013 | 0.0071 |
| 2(C) | 0.149 | 0.0755 | 0.1712 | 0.0223 | 0.0734 | 0.0479 | 0.0512 |
| 3(C) | -0.0022 | -0.0676 | 0.0484 | 0.0506 | 0.0653 | 0.058 | 0.0147 |
| 4(N) | -0.1871 | -0.213 | -0.1619 | 0.0253 | 0.0258 | 0.0256 | 0.0006 |
| 5(C) | 0.0878 | 0.0308 | 0.1119 | 0.0241 | 0.057 | 0.0405 | 0.0328 |
| 6(C) | 0.0249 | 0.0264 | 0.0241 | -0.0008 | -0.0015 | -0.0011 | -0.0007 |
| 7(C) | -0.0444 | -0.0508 | -0.041 | 0.0034 | 0.0063 | 0.0049 | 0.0029 |
| 8(C) | -0.0399 | -0.0458 | -0.0367 | 0.0033 | 0.0059 | 0.0046 | 0.0026 |
| 9(C) | -0.0387 | -0.047 | -0.0341 | 0.0046 | 0.0083 | 0.0064 | 0.0037 |
| 10(C) | -0.0399 | -0.0458 | -0.0366 | 0.0033 | 0.0059 | 0.0046 | 0.0026 |
| 11(C) | -0.0453 | -0.0513 | -0.0422 | 0.0031 | 0.006 | 0.0046 | 0.0028 |
| 12(O) | -0.3143 | -0.3998 | -0.273 | 0.0414 | 0.0855 | 0.0634 | 0.0441 |
| 13(S) | 0.0416 | -0.0277 | 0.0938 | 0.0523 | 0.0692 | 0.0608 | 0.017 |
| 14(C) | -0.0319 | -0.1486 | -0.0036 | 0.0282 | 0.1168 | 0.0725 | 0.0885 |
| 15(C) | -0.0308 | -0.0623 | 0.0238 | 0.0545 | 0.0316 | 0.0431 | -0.0229 |
| 16(C) | -0.0645 | -0.1156 | -0.0353 | 0.0293 | 0.0511 | 0.0402 | 0.0218 |
| 17(C) | 0.0513 | 0.0301 | 0.116 | 0.0646 | 0.0212 | 0.0429 | -0.0434 |
| 18(C) | -0.0601 | -0.1192 | 0.016 | 0.0761 | 0.0591 | 0.0676 | -0.0171 |
| 19(C) | -0.0714 | -0.1033 | -0.0193 | 0.0521 | 0.032 | 0.042 | -0.0201 |
| 20(C) | 0.0605 | 0.0273 | 0.1359 | 0.0754 | 0.0332 | 0.0543 | -0.0421 |
| 21(C) | -0.0475 | -0.0554 | -0.0425 | 0.0049 | 0.008 | 0.0065 | 0.0031 |
| 22(O) | -0.1192 | -0.1304 | -0.0546 | 0.0646 | 0.0112 | 0.0379 | -0.0534 |
| 23(C) | 0.0013 | -0.0055 | 0.0193 | 0.018 | 0.0068 | 0.0124 | -0.0112 |
| 24(O) | -0.1245 | -0.1312 | -0.0723 | 0.0522 | 0.0068 | 0.0295 | -0.0454 |
| 25(C) | -0.0013 | -0.0062 | 0.0131 | 0.0144 | 0.005 | 0.0097 | -0.0094 |
| 26(C) | 0.1988 | 0.1969 | 0.2002 | 0.0014 | 0.0019 | 0.0016 | 0.0005 |
| 27(O) | -0.2928 | -0.2987 | -0.2878 | 0.005 | 0.0059 | 0.0055 | 0.0009 |
| 28(H) | 0.0624 | 0.0577 | 0.0648 | 0.0023 | 0.0047 | 0.0035 | 0.0024 |
| 29(H) | 0.0608 | 0.0574 | 0.0627 | 0.0019 | 0.0034 | 0.0027 | 0.0015 |
| 30(H) | 0.0598 | 0.056 | 0.0619 | 0.0021 | 0.0038 | 0.003 | 0.0017 |
| 31(H) | 0.061 | 0.0575 | 0.063 | 0.0019 | 0.0035 | 0.0027 | 0.0015 |
| 32(H) | 0.0608 | 0.0563 | 0.0633 | 0.0024 | 0.0046 | 0.0035 | 0.0022 |
| 33(H) | 0.041 | -0.0019 | 0.0565 | 0.0155 | 0.043 | 0.0292 | 0.0275 |
| 34(H) | 0.0307 | 0.0128 | 0.0459 | 0.0152 | 0.018 | 0.0166 | 0.0028 |
| 35(H) | 0.0585 | 0.0351 | 0.0887 | 0.0302 | 0.0234 | 0.0268 | -0.0068 |
| 36(H) | 0.057 | 0.0415 | 0.0826 | 0.0256 | 0.0156 | 0.0206 | -0.01 |
| 37(H) | 0.0602 | 0.0512 | 0.0664 | 0.0062 | 0.009 | 0.0076 | 0.0028 |
| 38(H) | 0.0815 | 0.0719 | 0.0878 | 0.0063 | 0.0096 | 0.008 | 0.0033 |
| 39(H) | 0.0566 | 0.0511 | 0.0703 | 0.0137 | 0.0056 | 0.0096 | -0.0081 |
| 40(H) | 0.0494 | 0.0429 | 0.0682 | 0.0188 | 0.0065 | 0.0126 | -0.0124 |
| 41(H) | 0.0496 | 0.043 | 0.0683 | 0.0188 | 0.0065 | 0.0126 | -0.0123 |
| 42(H) | 0.055 | 0.0506 | 0.0669 | 0.0118 | 0.0044 | 0.0081 | -0.0074 |
| 43(H) | 0.0473 | 0.0419 | 0.0633 | 0.0159 | 0.0054 | 0.0107 | -0.0105 |
| 44(H) | 0.0474 | 0.0421 | 0.0634 | 0.016 | 0.0053 | 0.0107 | -0.0107 |
| 45(O) | -0.1044 | -0.1064 | -0.1038 | 0.0007 | 0.002 | 0.0013 | 0.0013 |
| 46(C) | 0.0282 | 0.0261 | 0.0303 | 0.0021 | 0.0021 | 0.0021 | 0 |
| 47(H) | 0.0393 | 0.0368 | 0.0416 | 0.0024 | 0.0025 | 0.0024 | 0.0001 |
| 48(H) | 0.037 | 0.0356 | 0.038 | 0.0009 | 0.0014 | 0.0012 | 0.0005 |
| 49(C) | -0.0957 | -0.0971 | -0.0941 | 0.0016 | 0.0014 | 0.0015 | -0.0002 |
| 50(H) | 0.0385 | 0.0372 | 0.0399 | 0.0014 | 0.0013 | 0.0014 | -0.0001 |
| 51(H) | 0.0335 | 0.0322 | 0.0352 | 0.0016 | 0.0013 | 0.0015 | -0.0004 |
| 52(H) | 0.0491 | 0.0476 | 0.0505 | 0.0014 | 0.0014 | 0.0014 | 0 |

**Table S17:** Fukui functions (local reactivity indices) include electrophilic (F⁻), nucleophilic (F⁺), and radical attacks (F⁰) on **2h (**Gas phase**)**. f⁻ = _qk_(N) - _qk_(N−1); f⁺ = _qk_(N+1) - _qk_(N); and f⁰ = _qk_(N+1) - _qk_(N−1). qk represents the electron population at the k^th^ atom in a neutral (N), anionic (N+1), or cationic (N−1) species (derived from Hirshfeld charges). The condensed dual descriptor (CDD = f⁺ − f⁻) differentiates nucleophilic (CDD > 0) from electrophilic (CDD < 0) attack preferences at each atomic center.

| Atom | q(N) | q(N+1) | q(N-1) | f⁻ | f⁺ | f⁰ | CDD |
| --- | --- | --- | --- | --- | --- | --- | --- |
| 1(N) | -0.0291 | -0.0417 | -0.0136 | 0.0156 | 0.0126 | 0.0141 | -0.003 |
| 2(C) | 0.1524 | 0.0939 | 0.1726 | 0.0202 | 0.0584 | 0.0393 | 0.0382 |
| 3(C) | 0.0038 | -0.0423 | 0.0522 | 0.0484 | 0.0461 | 0.0472 | -0.0022 |
| 4(N) | -0.18 | -0.1914 | -0.1478 | 0.0322 | 0.0113 | 0.0218 | -0.0209 |
| 5(C) | 0.084 | 0.0284 | 0.1124 | 0.0284 | 0.0556 | 0.042 | 0.0272 |
| 6(C) | 0.0351 | 0.0462 | 0.0263 | -0.0088 | -0.0111 | -0.01 | -0.0023 |
| 7(C) | -0.038 | -0.0404 | -0.0345 | 0.0035 | 0.0024 | 0.003 | -0.001 |
| 8(C) | -0.0352 | -0.0505 | -0.0214 | 0.0139 | 0.0153 | 0.0146 | 0.0014 |
| 9(C) | -0.0395 | -0.0623 | -0.0185 | 0.0209 | 0.0228 | 0.0219 | 0.0019 |
| 10(C) | -0.0383 | -0.0536 | -0.0245 | 0.0137 | 0.0153 | 0.0145 | 0.0015 |
| 11(C) | -0.0468 | -0.0498 | -0.0445 | 0.0023 | 0.0029 | 0.0026 | 0.0006 |
| 12(O) | -0.2745 | -0.3608 | -0.2104 | 0.0641 | 0.0863 | 0.0752 | 0.0222 |
| 13(S) | 0.035 | -0.0517 | 0.1325 | 0.0974 | 0.0867 | 0.0921 | -0.0107 |
| 14(C) | -0.0217 | -0.1085 | 0.0075 | 0.0292 | 0.0868 | 0.058 | 0.0576 |
| 15(C) | -0.0162 | -0.0351 | 0.0158 | 0.032 | 0.0189 | 0.0254 | -0.0131 |
| 16(C) | -0.0494 | -0.092 | -0.0365 | 0.013 | 0.0426 | 0.0278 | 0.0296 |
| 17(C) | 0.0562 | 0.0345 | 0.0991 | 0.0428 | 0.0218 | 0.0323 | -0.0211 |
| 18(C) | -0.0646 | -0.1244 | -0.0015 | 0.063 | 0.0599 | 0.0615 | -0.0032 |
| 19(C) | -0.0734 | -0.1061 | -0.0398 | 0.0335 | 0.0327 | 0.0331 | -0.0008 |
| 20(C) | 0.0663 | 0.0414 | 0.1191 | 0.0528 | 0.0249 | 0.0388 | -0.0279 |
| 21(O) | -0.1114 | -0.1172 | -0.0641 | 0.0473 | 0.0058 | 0.0266 | -0.0415 |
| 22(C) | -0.0017 | -0.0118 | 0.0143 | 0.016 | 0.0101 | 0.0131 | -0.0058 |
| 23(O) | -0.1307 | -0.1403 | -0.0919 | 0.0388 | 0.0096 | 0.0242 | -0.0292 |
| 24(C) | -0.006 | -0.0157 | 0.0073 | 0.0133 | 0.0097 | 0.0115 | -0.0037 |
| 25(H) | 0.0518 | 0.0494 | 0.054 | 0.0021 | 0.0024 | 0.0023 | 0.0003 |
| 26(H) | 0.0491 | 0.0358 | 0.0608 | 0.0118 | 0.0133 | 0.0125 | 0.0015 |
| 27(H) | 0.0468 | 0.0304 | 0.0611 | 0.0143 | 0.0164 | 0.0153 | 0.0021 |
| 28(H) | 0.048 | 0.0347 | 0.0596 | 0.0116 | 0.0134 | 0.0125 | 0.0017 |
| 29(H) | 0.0441 | 0.0407 | 0.0465 | 0.0023 | 0.0034 | 0.0029 | 0.0011 |
| 30(H) | 0.0453 | 0.0126 | 0.0613 | 0.016 | 0.0327 | 0.0244 | 0.0167 |
| 31(H) | 0.0376 | 0.0218 | 0.0494 | 0.0117 | 0.0158 | 0.0138 | 0.0041 |
| 32(H) | 0.041 | 0.0107 | 0.0699 | 0.0289 | 0.0303 | 0.0296 | 0.0015 |
| 33(H) | 0.0402 | 0.0177 | 0.0636 | 0.0234 | 0.0224 | 0.0229 | -0.001 |
| 34(H) | 0.0521 | 0.0386 | 0.0706 | 0.0185 | 0.0135 | 0.016 | -0.005 |
| 35(H) | 0.0384 | 0.0264 | 0.0561 | 0.0177 | 0.012 | 0.0148 | -0.0057 |
| 36(H) | 0.0385 | 0.0266 | 0.0562 | 0.0177 | 0.012 | 0.0148 | -0.0057 |
| 37(H) | 0.0487 | 0.0341 | 0.0669 | 0.0182 | 0.0147 | 0.0164 | -0.0035 |
| 38(H) | 0.0363 | 0.0258 | 0.0506 | 0.0143 | 0.0105 | 0.0124 | -0.0038 |
| 39(H) | 0.0363 | 0.0258 | 0.0506 | 0.0143 | 0.0105 | 0.0124 | -0.0038 |
| 40(C) | -0.0788 | -0.0915 | -0.068 | 0.0109 | 0.0126 | 0.0118 | 0.0018 |
| 41(H) | 0.0492 | 0.0408 | 0.0569 | 0.0077 | 0.0084 | 0.008 | 0.0007 |
| 42(H) | 0.0494 | 0.0409 | 0.057 | 0.0077 | 0.0084 | 0.0081 | 0.0007 |
| 43(H) | 0.0503 | 0.031 | 0.0674 | 0.0171 | 0.0193 | 0.0182 | 0.0022 |

**Table S18:** Fukui functions (local reactivity indices) include electrophilic (F⁻), nucleophilic (F⁺), and radical attacks (F⁰) on **2h (**DMSO phase**)**. f⁻ = _qk_(N) - _qk_(N−1); f⁺ = _qk_(N+1) - _qk_(N); and f⁰ = _qk_(N+1) - _qk_(N−1). qk represents the electron population at the k^th^ atom in a neutral (N), anionic (N+1), or cationic (N−1) species (derived from Hirshfeld charges). The condensed dual descriptor (CDD = f⁺ − f⁻) differentiates nucleophilic (CDD > 0) from electrophilic (CDD < 0) attack preferences at each atomic center.

| Atom | q(N) | q(N+1) | q(N-1) | f⁻ | f⁺ | f⁰ | CDD |
| --- | --- | --- | --- | --- | --- | --- | --- |
| 1(N) | -0.0244 | -0.0404 | -0.0153 | 0.0091 | 0.0161 | 0.0126 | 0.007 |
| 2(C) | 0.1478 | 0.0752 | 0.1696 | 0.0218 | 0.0726 | 0.0472 | 0.0508 |
| 3(C) | -0.0041 | -0.0698 | 0.0469 | 0.051 | 0.0657 | 0.0583 | 0.0148 |
| 4(N) | -0.1989 | -0.2281 | -0.1687 | 0.0302 | 0.0292 | 0.0297 | -0.0011 |
| 5(C) | 0.0858 | 0.0282 | 0.1107 | 0.0249 | 0.0576 | 0.0413 | 0.0327 |
| 6(C) | 0.0247 | 0.0263 | 0.024 | -0.0008 | -0.0015 | -0.0012 | -0.0007 |
| 7(C) | -0.0451 | -0.0512 | -0.0417 | 0.0033 | 0.0061 | 0.0047 | 0.0028 |
| 8(C) | -0.0403 | -0.0461 | -0.0371 | 0.0033 | 0.0058 | 0.0045 | 0.0025 |
| 9(C) | -0.0392 | -0.0473 | -0.0347 | 0.0045 | 0.0081 | 0.0063 | 0.0036 |
| 10(C) | -0.0405 | -0.0462 | -0.0372 | 0.0033 | 0.0057 | 0.0045 | 0.0025 |
| 11(C) | -0.046 | -0.0519 | -0.0429 | 0.0031 | 0.0059 | 0.0045 | 0.0028 |
| 12(O) | -0.3159 | -0.4002 | -0.2754 | 0.0406 | 0.0842 | 0.0624 | 0.0437 |
| 13(S) | 0.0298 | -0.042 | 0.0875 | 0.0577 | 0.0718 | 0.0648 | 0.0141 |
| 14(C) | -0.0346 | -0.1501 | -0.0051 | 0.0295 | 0.1155 | 0.0725 | 0.086 |
| 15(C) | -0.0316 | -0.0635 | 0.0217 | 0.0532 | 0.0319 | 0.0426 | -0.0213 |
| 16(C) | -0.0694 | -0.1234 | -0.0392 | 0.0303 | 0.054 | 0.0421 | 0.0237 |
| 17(C) | 0.0476 | 0.0256 | 0.112 | 0.0644 | 0.022 | 0.0432 | -0.0424 |
| 18(C) | -0.0614 | -0.1201 | 0.0146 | 0.076 | 0.0587 | 0.0673 | -0.0174 |
| 19(C) | -0.0716 | -0.1032 | -0.021 | 0.0506 | 0.0316 | 0.0411 | -0.019 |
| 20(C) | 0.0598 | 0.027 | 0.1342 | 0.0743 | 0.0328 | 0.0536 | -0.0415 |
| 21(O) | -0.1197 | -0.1307 | -0.0567 | 0.063 | 0.011 | 0.037 | -0.052 |
| 22(C) | 0.0012 | -0.0055 | 0.0188 | 0.0176 | 0.0067 | 0.0121 | -0.0109 |
| 23(O) | -0.1412 | -0.1491 | -0.0871 | 0.0541 | 0.0079 | 0.031 | -0.0462 |
| 24(C) | -0.0035 | -0.0085 | 0.011 | 0.0145 | 0.005 | 0.0098 | -0.0095 |
| 25(H) | 0.062 | 0.0574 | 0.0643 | 0.0023 | 0.0047 | 0.0035 | 0.0024 |
| 26(H) | 0.0607 | 0.0573 | 0.0626 | 0.0019 | 0.0034 | 0.0026 | 0.0015 |
| 27(H) | 0.0596 | 0.0559 | 0.0617 | 0.0021 | 0.0037 | 0.0029 | 0.0016 |
| 28(H) | 0.0607 | 0.0573 | 0.0626 | 0.0019 | 0.0034 | 0.0026 | 0.0015 |
| 29(H) | 0.0606 | 0.056 | 0.063 | 0.0024 | 0.0046 | 0.0035 | 0.0021 |
| 30(H) | 0.0401 | -0.0023 | 0.0556 | 0.0155 | 0.0424 | 0.029 | 0.0269 |
| 31(H) | 0.0304 | 0.0082 | 0.0487 | 0.0183 | 0.0222 | 0.0202 | 0.0039 |
| 32(H) | 0.0581 | 0.0351 | 0.0881 | 0.0299 | 0.0231 | 0.0265 | -0.0069 |
| 33(H) | 0.057 | 0.0416 | 0.082 | 0.025 | 0.0154 | 0.0202 | -0.0097 |
| 34(H) | 0.0565 | 0.0511 | 0.0698 | 0.0133 | 0.0054 | 0.0093 | -0.0078 |
| 35(H) | 0.0493 | 0.043 | 0.0677 | 0.0184 | 0.0064 | 0.0124 | -0.012 |
| 36(H) | 0.0493 | 0.043 | 0.0677 | 0.0184 | 0.0064 | 0.0124 | -0.012 |
| 37(H) | 0.0541 | 0.0498 | 0.0656 | 0.0116 | 0.0043 | 0.0079 | -0.0073 |
| 38(H) | 0.0467 | 0.0414 | 0.0623 | 0.0156 | 0.0053 | 0.0104 | -0.0103 |
| 39(H) | 0.0467 | 0.0414 | 0.0623 | 0.0156 | 0.0053 | 0.0104 | -0.0103 |
| 40(C) | -0.0743 | -0.0855 | -0.0666 | 0.0076 | 0.0113 | 0.0095 | 0.0036 |
| 41(H) | 0.0542 | 0.0446 | 0.0614 | 0.0072 | 0.0096 | 0.0084 | 0.0024 |
| 42(H) | 0.0543 | 0.0446 | 0.0614 | 0.0072 | 0.0097 | 0.0084 | 0.0025 |
| 43(H) | 0.0652 | 0.056 | 0.0713 | 0.0061 | 0.0091 | 0.0076 | 0.003 |

**Table S19:** Fukui functions (local reactivity indices) include electrophilic (F⁻), nucleophilic (F⁺), and radical attacks (F⁰) on **2i (**Gas phase**)**. f⁻ = _qk_(N) - _qk_(N−1); f⁺ = _qk_(N+1) - _qk_(N); and f⁰ = _qk_(N+1) - _qk_(N−1). qk represents the electron population at the k^th^ atom in a neutral (N), anionic (N+1), or cationic (N−1) species (derived from Hirshfeld charges). The condensed dual descriptor (CDD = f⁺ − f⁻) differentiates nucleophilic (CDD > 0) from electrophilic (CDD < 0) attack preferences at each atomic center.

| Atom | q(N) | q(N+1) | q(N-1) | f⁻ | f⁺ | f⁰ | CDD |
| --- | --- | --- | --- | --- | --- | --- | --- |
| 1(N) | -0.0282 | -0.0415 | -0.0122 | 0.016 | 0.0133 | 0.0147 | -0.0027 |
| 2(C) | 0.1524 | 0.0999 | 0.1736 | 0.0212 | 0.0525 | 0.0368 | 0.0313 |
| 3(C) | 0.0033 | -0.0372 | 0.0519 | 0.0486 | 0.0405 | 0.0446 | -0.0081 |
| 4(N) | -0.1714 | -0.1859 | -0.1491 | 0.0223 | 0.0145 | 0.0184 | -0.0078 |
| 5(C) | 0.0846 | 0.0249 | 0.1138 | 0.0292 | 0.0597 | 0.0444 | 0.0305 |
| 6(C) | 0.0325 | 0.0438 | 0.0242 | -0.0083 | -0.0113 | -0.0098 | -0.003 |
| 7(C) | -0.0364 | -0.0385 | -0.0337 | 0.0027 | 0.0021 | 0.0024 | -0.0007 |
| 8(C) | -0.0328 | -0.0474 | -0.02 | 0.0127 | 0.0147 | 0.0137 | 0.0019 |
| 9(C) | -0.0348 | -0.0564 | -0.0153 | 0.0195 | 0.0216 | 0.0205 | 0.0021 |
| 10(C) | -0.035 | -0.0495 | -0.0221 | 0.013 | 0.0145 | 0.0137 | 0.0015 |
| 11(C) | -0.0443 | -0.0466 | -0.0418 | 0.0024 | 0.0023 | 0.0024 | -0.0002 |
| 12(O) | -0.2706 | -0.3533 | -0.2058 | 0.0648 | 0.0827 | 0.0737 | 0.0179 |
| 13(S) | 0.0468 | -0.0144 | 0.1107 | 0.0639 | 0.0611 | 0.0625 | -0.0028 |
| 14(C) | -0.0135 | -0.1018 | 0.0048 | 0.0183 | 0.0883 | 0.0533 | 0.07 |
| 15(C) | -0.017 | -0.0319 | 0.0187 | 0.0357 | 0.0149 | 0.0253 | -0.0208 |
| 16(C) | -0.0499 | -0.0876 | -0.0363 | 0.0136 | 0.0377 | 0.0257 | 0.024 |
| 17(C) | 0.0587 | 0.039 | 0.1063 | 0.0476 | 0.0197 | 0.0336 | -0.0279 |
| 18(C) | -0.0593 | -0.1158 | 0.0032 | 0.0625 | 0.0565 | 0.0595 | -0.006 |
| 19(C) | -0.0703 | -0.1008 | -0.0346 | 0.0358 | 0.0305 | 0.0331 | -0.0053 |
| 20(C) | 0.0687 | 0.0439 | 0.1251 | 0.0563 | 0.0248 | 0.0406 | -0.0315 |
| 21(O) | -0.1106 | -0.1168 | -0.0566 | 0.054 | 0.0062 | 0.0301 | -0.0479 |
| 22(C) | -0.0005 | -0.0102 | 0.0166 | 0.0171 | 0.0098 | 0.0134 | -0.0073 |
| 23(O) | -0.126 | -0.1339 | -0.0792 | 0.0468 | 0.0079 | 0.0274 | -0.039 |
| 24(C) | -0.0042 | -0.0129 | 0.0104 | 0.0146 | 0.0088 | 0.0117 | -0.0058 |
| 25(H) | 0.0531 | 0.051 | 0.0547 | 0.0016 | 0.0021 | 0.0019 | 0.0005 |
| 26(H) | 0.0509 | 0.0381 | 0.0619 | 0.011 | 0.0128 | 0.0119 | 0.0019 |
| 27(H) | 0.0493 | 0.0337 | 0.0626 | 0.0133 | 0.0156 | 0.0145 | 0.0023 |
| 28(H) | 0.0506 | 0.0379 | 0.0615 | 0.0109 | 0.0127 | 0.0118 | 0.0018 |
| 29(H) | 0.047 | 0.0446 | 0.0491 | 0.002 | 0.0024 | 0.0022 | 0.0004 |
| 30(H) | 0.0488 | 0.0162 | 0.062 | 0.0131 | 0.0326 | 0.0229 | 0.0195 |
| 31(H) | 0.0352 | 0.0229 | 0.0471 | 0.0119 | 0.0123 | 0.0121 | 0.0004 |
| 32(H) | 0.0437 | 0.0153 | 0.0727 | 0.029 | 0.0284 | 0.0287 | -0.0006 |
| 33(H) | 0.0421 | 0.0208 | 0.0662 | 0.0242 | 0.0213 | 0.0227 | -0.0029 |
| 34(H) | 0.0534 | 0.0404 | 0.0729 | 0.0196 | 0.0129 | 0.0162 | -0.0066 |
| 35(H) | 0.0398 | 0.0283 | 0.0585 | 0.0187 | 0.0115 | 0.0151 | -0.0072 |
| 36(H) | 0.0398 | 0.0284 | 0.0585 | 0.0187 | 0.0115 | 0.0151 | -0.0072 |
| 37(H) | 0.0513 | 0.0385 | 0.0699 | 0.0186 | 0.0128 | 0.0157 | -0.0058 |
| 38(H) | 0.0371 | 0.0271 | 0.0531 | 0.016 | 0.0101 | 0.013 | -0.0059 |
| 39(H) | 0.0373 | 0.0274 | 0.0531 | 0.0158 | 0.0099 | 0.0129 | -0.0059 |
| 40(C) | 0.1542 | 0.1282 | 0.1634 | 0.0092 | 0.026 | 0.0176 | 0.0168 |
| 41(O) | -0.2047 | -0.2564 | -0.1689 | 0.0358 | 0.0517 | 0.0438 | 0.0159 |
| 42(C) | -0.0141 | -0.0151 | -0.0156 | -0.0016 | 0.001 | -0.0003 | 0.0026 |
| 43(H) | 0.0586 | 0.0571 | 0.0563 | -0.0023 | 0.0014 | -0.0004 | 0.0037 |
| 44(H) | 0.0583 | 0.0573 | 0.0558 | -0.0025 | 0.001 | -0.0007 | 0.0035 |
| 45(Cl) | -0.0729 | -0.1095 | -0.0465 | 0.0264 | 0.0366 | 0.0315 | 0.0102 |

**Table S20:** Fukui functions (local reactivity indices) include electrophilic (F⁻), nucleophilic (F⁺), and radical attacks (F⁰) on **2i (**DMSO phase**)**. f⁻ = _qk_(N) - _qk_(N−1); f⁺ = _qk_(N+1) - _qk_(N); and f⁰ = _qk_(N+1) - _qk_(N−1). qk represents the electron population at the k^th^ atom in a neutral (N), anionic (N+1), or cationic (N−1) species (derived from Hirshfeld charges). The condensed dual descriptor (CDD = f⁺ − f⁻) differentiates nucleophilic (CDD > 0) from electrophilic (CDD < 0) attack preferences at each atomic center.

| Atom | q(N) | q(N+1) | q(N-1) | f⁻ | f⁺ | f⁰ | CDD |
| --- | --- | --- | --- | --- | --- | --- | --- |
| 1(N) | -0.0222 | -0.0383 | -0.0138 | 0.0085 | 0.0161 | 0.0123 | 0.0077 |
| 2(C) | 0.1493 | 0.0815 | 0.1705 | 0.0212 | 0.0678 | 0.0445 | 0.0466 |
| 3(C) | -0.0006 | -0.0616 | 0.0459 | 0.0465 | 0.061 | 0.0537 | 0.0144 |
| 4(N) | -0.1723 | -0.2031 | -0.1543 | 0.018 | 0.0308 | 0.0244 | 0.0128 |
| 5(C) | 0.0926 | 0.0302 | 0.116 | 0.0234 | 0.0624 | 0.0429 | 0.0389 |
| 6(C) | 0.0231 | 0.0249 | 0.0223 | -0.0008 | -0.0018 | -0.0013 | -0.0011 |
| 7(C) | -0.0431 | -0.0491 | -0.0403 | 0.0028 | 0.006 | 0.0044 | 0.0032 |
| 8(C) | -0.0384 | -0.0441 | -0.0356 | 0.0028 | 0.0057 | 0.0042 | 0.0029 |
| 9(C) | -0.0363 | -0.0443 | -0.0324 | 0.0039 | 0.008 | 0.006 | 0.0041 |
| 10(C) | -0.0384 | -0.0441 | -0.0357 | 0.0028 | 0.0057 | 0.0042 | 0.0029 |
| 11(C) | -0.0436 | -0.0495 | -0.0408 | 0.0028 | 0.0059 | 0.0043 | 0.0031 |
| 12(O) | -0.3124 | -0.3937 | -0.2735 | 0.0389 | 0.0812 | 0.0601 | 0.0424 |
| 13(S) | 0.0455 | -0.0072 | 0.0763 | 0.0307 | 0.0527 | 0.0417 | 0.0219 |
| 14(C) | -0.0213 | -0.139 | -0.0017 | 0.0197 | 0.1177 | 0.0687 | 0.098 |
| 15(C) | -0.03 | -0.0589 | 0.0258 | 0.0558 | 0.0289 | 0.0424 | -0.0269 |
| 16(C) | -0.0638 | -0.1143 | -0.0276 | 0.0362 | 0.0506 | 0.0434 | 0.0144 |
| 17(C) | 0.0501 | 0.029 | 0.1223 | 0.0722 | 0.0211 | 0.0467 | -0.051 |
| 18(C) | -0.0563 | -0.1134 | 0.0188 | 0.0751 | 0.0571 | 0.0661 | -0.018 |
| 19(C) | -0.0691 | -0.0993 | -0.0121 | 0.057 | 0.0302 | 0.0436 | -0.0268 |
| 20(C) | 0.0635 | 0.0305 | 0.142 | 0.0785 | 0.033 | 0.0557 | -0.0454 |
| 21(O) | -0.1178 | -0.1293 | -0.048 | 0.0699 | 0.0114 | 0.0406 | -0.0584 |
| 22(C) | 0.0023 | -0.0044 | 0.0215 | 0.0192 | 0.0067 | 0.0129 | -0.0125 |
| 23(O) | -0.1381 | -0.1455 | -0.0734 | 0.0647 | 0.0074 | 0.0361 | -0.0573 |
| 24(C) | -0.0032 | -0.008 | 0.0139 | 0.0171 | 0.0048 | 0.0109 | -0.0123 |
| 25(H) | 0.0636 | 0.0592 | 0.0656 | 0.0019 | 0.0044 | 0.0032 | 0.0025 |
| 26(H) | 0.0617 | 0.0583 | 0.0633 | 0.0016 | 0.0033 | 0.0025 | 0.0017 |
| 27(H) | 0.0607 | 0.0571 | 0.0625 | 0.0018 | 0.0037 | 0.0027 | 0.0019 |
| 28(H) | 0.0617 | 0.0584 | 0.0634 | 0.0016 | 0.0033 | 0.0025 | 0.0017 |
| 29(H) | 0.0633 | 0.0589 | 0.0652 | 0.0019 | 0.0044 | 0.0031 | 0.0025 |
| 30(H) | 0.0446 | 0.0021 | 0.0574 | 0.0129 | 0.0425 | 0.0277 | 0.0296 |
| 31(H) | 0.0386 | 0.0199 | 0.0566 | 0.018 | 0.0187 | 0.0184 | 0.0007 |
| 32(H) | 0.0598 | 0.0375 | 0.0906 | 0.0308 | 0.0223 | 0.0265 | -0.0085 |
| 33(H) | 0.0583 | 0.0435 | 0.0857 | 0.0273 | 0.0149 | 0.0211 | -0.0125 |
| 34(H) | 0.0572 | 0.0518 | 0.0716 | 0.0144 | 0.0054 | 0.0099 | -0.009 |
| 35(H) | 0.0502 | 0.0439 | 0.0703 | 0.0201 | 0.0063 | 0.0132 | -0.0138 |
| 36(H) | 0.0502 | 0.0439 | 0.0703 | 0.0201 | 0.0063 | 0.0132 | -0.0137 |
| 37(H) | 0.0535 | 0.0493 | 0.0667 | 0.0133 | 0.0042 | 0.0087 | -0.0091 |
| 38(H) | 0.047 | 0.0419 | 0.0654 | 0.0184 | 0.0051 | 0.0118 | -0.0133 |
| 39(H) | 0.047 | 0.0419 | 0.0654 | 0.0184 | 0.0051 | 0.0117 | -0.0133 |
| 40(C) | 0.1594 | 0.1393 | 0.1651 | 0.0057 | 0.0201 | 0.0129 | 0.0144 |
| 41(O) | -0.2341 | -0.2616 | -0.2234 | 0.0107 | 0.0276 | 0.0191 | 0.0168 |
| 42(C) | -0.0054 | -0.01 | -0.0037 | 0.0017 | 0.0045 | 0.0031 | 0.0028 |
| 43(H) | 0.0724 | 0.0655 | 0.0753 | 0.0029 | 0.0068 | 0.0049 | 0.0039 |
| 44(H) | 0.0728 | 0.0661 | 0.0756 | 0.0028 | 0.0066 | 0.0047 | 0.0038 |
| 45(Cl) | -0.1013 | -0.1152 | -0.0944 | 0.0069 | 0.0139 | 0.0104 | 0.007 |

**Table S21:** Fukui functions (local reactivity indices) include electrophilic (F⁻), nucleophilic (F⁺), and radical attacks (F⁰) on **3 (**Gas phase**)**. f⁻ = _qk_(N) - _qk_(N−1); f⁺ = _qk_(N+1) - _qk_(N); and f⁰ = _qk_(N+1) - _qk_(N−1). qk represents the electron population at the k^th^ atom in a neutral (N), anionic (N+1), or cationic (N−1) species (derived from Hirshfeld charges). The condensed dual descriptor (CDD = f⁺ − f⁻) differentiates nucleophilic (CDD > 0) from electrophilic (CDD < 0) attack preferences at each atomic center.

| Atom | q(N) | q(N+1) | q(N-1) | f⁻ | f⁺ | f⁰ | CDD |
| --- | --- | --- | --- | --- | --- | --- | --- |
| 1(C) | 0.1732 | 0.1047 | 0.1764 | 0.0033 | 0.0684 | 0.0358 | 0.0651 |
| 2(N) | -0.0121 | -0.0244 | -0.0094 | 0.0027 | 0.0123 | 0.0075 | 0.0096 |
| 3(C) | 0.097 | 0.0249 | 0.1062 | 0.0093 | 0.0721 | 0.0407 | 0.0628 |
| 4(N) | -0.086 | -0.1185 | -0.0702 | 0.0158 | 0.0326 | 0.0242 | 0.0167 |
| 5(C) | 0.0201 | 0.0028 | 0.0288 | 0.0087 | 0.0173 | 0.013 | 0.0086 |
| 6(C) | 0.0349 | 0.0476 | 0.0327 | -0.0022 | -0.0127 | -0.0074 | -0.0105 |
| 7(O) | -0.2533 | -0.345 | -0.2292 | 0.0241 | 0.0916 | 0.0579 | 0.0675 |
| 8(S) | -0.2371 | -0.4225 | -0.0875 | 0.1497 | 0.1854 | 0.1675 | 0.0357 |
| 9(C) | -0.0381 | -0.0438 | -0.0276 | 0.0105 | 0.0057 | 0.0081 | -0.0048 |
| 10(C) | -0.0363 | -0.0551 | -0.0105 | 0.0258 | 0.0188 | 0.0223 | -0.007 |
| 11(C) | -0.036 | -0.0599 | -0.014 | 0.022 | 0.0239 | 0.0229 | 0.0018 |
| 12(C) | -0.0335 | -0.0469 | -0.0258 | 0.0077 | 0.0133 | 0.0105 | 0.0056 |
| 13(C) | -0.0393 | -0.0361 | -0.0443 | -0.005 | -0.0032 | -0.0041 | 0.0018 |
| 14(C) | -0.0032 | -0.015 | -0.0003 | 0.0029 | 0.0118 | 0.0074 | 0.0089 |
| 15(C) | -0.0279 | -0.0335 | 0.0059 | 0.0338 | 0.0056 | 0.0197 | -0.0282 |
| 16(C) | 0.0296 | 0.0224 | 0.0319 | 0.0023 | 0.0071 | 0.0047 | 0.0048 |
| 17(C) | 0.0554 | 0.0533 | 0.1056 | 0.0502 | 0.0021 | 0.0261 | -0.0481 |
| 18(C) | -0.054 | -0.0797 | -0.0218 | 0.0322 | 0.0258 | 0.029 | -0.0064 |
| 19(C) | -0.0441 | -0.0767 | 0.0017 | 0.0458 | 0.0326 | 0.0392 | -0.0132 |
| 20(C) | 0.0685 | 0.0548 | 0.1194 | 0.0509 | 0.0138 | 0.0323 | -0.0371 |
| 21(C) | -0.0605 | -0.0786 | -0.0263 | 0.0342 | 0.0182 | 0.0262 | -0.016 |
| 22(C) | 0.078 | 0.0739 | 0.0776 | -0.0003 | 0.004 | 0.0018 | 0.0044 |
| 23(N) | -0.1885 | -0.2294 | -0.1528 | 0.0358 | 0.0408 | 0.0383 | 0.005 |
| 24(C) | 0.0764 | 0.0644 | 0.0724 | -0.004 | 0.012 | 0.004 | 0.016 |
| 25(N) | -0.1897 | -0.2385 | -0.163 | 0.0267 | 0.0488 | 0.0377 | 0.0221 |
| 26(O) | -0.108 | -0.1078 | -0.0595 | 0.0485 | -0.0002 | 0.0241 | -0.0487 |
| 27(C) | -0.0022 | -0.0108 | 0.0143 | 0.0165 | 0.0086 | 0.0125 | -0.0079 |
| 28(O) | -0.1248 | -0.1378 | -0.0556 | 0.0692 | 0.013 | 0.0411 | -0.0562 |
| 29(C) | -0.0034 | -0.0101 | 0.0153 | 0.0188 | 0.0067 | 0.0127 | -0.012 |
| 30(H) | 0.1252 | 0.1029 | 0.1342 | 0.0089 | 0.0223 | 0.0156 | 0.0134 |
| 31(H) | 0.0554 | 0.0302 | 0.0732 | 0.0178 | 0.0252 | 0.0215 | 0.0075 |
| 32(H) | 0.0514 | 0.0452 | 0.062 | 0.0106 | 0.0062 | 0.0084 | -0.0044 |
| 33(H) | 0.0495 | 0.033 | 0.067 | 0.0175 | 0.0164 | 0.017 | -0.0011 |
| 34(H) | 0.0481 | 0.0299 | 0.0646 | 0.0165 | 0.0182 | 0.0174 | 0.0016 |
| 35(H) | 0.0501 | 0.0379 | 0.0586 | 0.0085 | 0.0122 | 0.0104 | 0.0037 |
| 36(H) | 0.0363 | 0.0378 | 0.0296 | -0.0067 | -0.0015 | -0.0041 | 0.0052 |
| 37(H) | 0.0529 | 0.0414 | 0.0637 | 0.0108 | 0.0115 | 0.0111 | 0.0007 |
| 38(H) | 0.0667 | 0.0616 | 0.067 | 0.0002 | 0.0051 | 0.0027 | 0.0048 |
| 39(H) | 0.049 | 0.0309 | 0.0711 | 0.022 | 0.0181 | 0.0201 | -0.0039 |
| 40(H) | 0.0549 | 0.0334 | 0.0825 | 0.0276 | 0.0215 | 0.0246 | -0.006 |
| 41(H) | 0.0441 | 0.0331 | 0.0635 | 0.0194 | 0.011 | 0.0152 | -0.0084 |
| 42(H) | 0.0506 | 0.038 | 0.0706 | 0.0199 | 0.0127 | 0.0163 | -0.0073 |
| 43(H) | 0.0444 | 0.0307 | 0.0656 | 0.0212 | 0.0136 | 0.0174 | -0.0075 |
| 44(H) | 0.0382 | 0.0299 | 0.0508 | 0.0125 | 0.0083 | 0.0104 | -0.0042 |
| 45(H) | 0.0511 | 0.0372 | 0.0728 | 0.0218 | 0.0139 | 0.0178 | -0.0079 |
| 46(H) | 0.0405 | 0.0312 | 0.0623 | 0.0219 | 0.0093 | 0.0156 | -0.0126 |
| 47(H) | 0.0379 | 0.0392 | 0.0514 | 0.0135 | -0.0012 | 0.0061 | -0.0147 |

**Table S22:** Fukui functions (local reactivity indices) include electrophilic (F⁻), nucleophilic (F⁺), and radical attacks (F⁰) on **3 (**DMSO phase**)**. f⁻ = _qk_(N) - _qk_(N−1); f⁺ = _qk_(N+1) - _qk_(N); and f⁰ = _qk_(N+1) - _qk_(N−1). qk represents the electron population at the k^th^ atom in a neutral (N), anionic (N+1), or cationic (N−1) species (derived from Hirshfeld charges). The condensed dual descriptor (CDD = f⁺ − f⁻) differentiates nucleophilic (CDD > 0) from electrophilic (CDD < 0) attack preferences at each atomic center.

| Atom | q(N) | q(N+1) | q(N-1) | f⁻ | f⁺ | f⁰ | CDD |
| --- | --- | --- | --- | --- | --- | --- | --- |
| 1(C) | 0.1792 | 0.0875 | 0.1795 | 0.0003 | 0.0917 | 0.046 | 0.0914 |
| 2(N) | -0.0109 | -0.0321 | -0.0094 | 0.0014 | 0.0212 | 0.0113 | 0.0198 |
| 3(C) | 0.1012 | 0.0012 | 0.1019 | 0.0007 | 0.1 | 0.0504 | 0.0993 |
| 4(N) | -0.0572 | -0.1052 | -0.0581 | -0.0009 | 0.048 | 0.0235 | 0.0488 |
| 5(C) | 0.0347 | 0.0103 | 0.0394 | 0.0048 | 0.0243 | 0.0146 | 0.0196 |
| 6(C) | 0.0253 | 0.0282 | 0.0257 | 0.0004 | -0.0029 | -0.0013 | -0.0034 |
| 7(O) | -0.2784 | -0.3848 | -0.2705 | 0.0079 | 0.1064 | 0.0572 | 0.0985 |
| 8(S) | -0.3381 | -0.5697 | -0.3092 | 0.0288 | 0.2316 | 0.1302 | 0.2028 |
| 9(C) | -0.0463 | -0.057 | -0.0433 | 0.003 | 0.0107 | 0.0069 | 0.0078 |
| 10(C) | -0.0418 | -0.0515 | -0.0382 | 0.0036 | 0.0097 | 0.0067 | 0.0061 |
| 11(C) | -0.0403 | -0.053 | -0.037 | 0.0034 | 0.0127 | 0.008 | 0.0093 |
| 12(C) | -0.0401 | -0.0502 | -0.0374 | 0.0027 | 0.0101 | 0.0064 | 0.0074 |
| 13(C) | -0.0464 | -0.055 | -0.0445 | 0.0018 | 0.0086 | 0.0052 | 0.0067 |
| 14(C) | 0.0043 | -0.0104 | 0.0091 | 0.0048 | 0.0146 | 0.0097 | 0.0098 |
| 15(C) | -0.033 | -0.0319 | 0.0211 | 0.0541 | -0.0011 | 0.0265 | -0.0552 |
| 16(C) | 0.0457 | 0.0373 | 0.0492 | 0.0035 | 0.0085 | 0.006 | 0.0049 |
| 17(C) | 0.05 | 0.0473 | 0.1315 | 0.0814 | 0.0027 | 0.0421 | -0.0787 |
| 18(C) | -0.062 | -0.0781 | 0.0007 | 0.0627 | 0.0162 | 0.0394 | -0.0465 |
| 19(C) | -0.0552 | -0.0714 | 0.022 | 0.0772 | 0.0162 | 0.0467 | -0.0609 |
| 20(C) | 0.058 | 0.0501 | 0.1434 | 0.0854 | 0.0079 | 0.0466 | -0.0774 |
| 21(C) | -0.0633 | -0.072 | -0.002 | 0.0613 | 0.0087 | 0.035 | -0.0526 |
| 22(C) | 0.1096 | 0.0999 | 0.1146 | 0.0049 | 0.0097 | 0.0073 | 0.0048 |
| 23(N) | -0.2447 | -0.2643 | -0.2319 | 0.0128 | 0.0197 | 0.0162 | 0.0069 |
| 24(C) | 0.0996 | 0.0859 | 0.1007 | 0.0011 | 0.0137 | 0.0074 | 0.0125 |
| 25(N) | -0.2487 | -0.2751 | -0.234 | 0.0146 | 0.0265 | 0.0206 | 0.0118 |
| 26(O) | -0.109 | -0.1087 | -0.0365 | 0.0726 | -0.0004 | 0.0361 | -0.0729 |
| 27(C) | 0.0022 | -0.0023 | 0.0233 | 0.0211 | 0.0046 | 0.0128 | -0.0165 |
| 28(O) | -0.1369 | -0.1413 | -0.0439 | 0.093 | 0.0045 | 0.0487 | -0.0885 |
| 29(C) | -0.0033 | -0.0057 | 0.0207 | 0.024 | 0.0024 | 0.0132 | -0.0216 |
| 30(H) | 0.1585 | 0.1271 | 0.162 | 0.0035 | 0.0314 | 0.0175 | 0.0278 |
| 31(H) | 0.0763 | 0.0368 | 0.086 | 0.0097 | 0.0395 | 0.0246 | 0.0298 |
| 32(H) | 0.0617 | 0.0538 | 0.0638 | 0.0021 | 0.0079 | 0.005 | 0.0057 |
| 33(H) | 0.0599 | 0.0544 | 0.0618 | 0.0019 | 0.0055 | 0.0037 | 0.0036 |
| 34(H) | 0.059 | 0.0531 | 0.0609 | 0.0018 | 0.0059 | 0.0039 | 0.004 |
| 35(H) | 0.0599 | 0.0542 | 0.0618 | 0.0019 | 0.0057 | 0.0038 | 0.0039 |
| 36(H) | 0.0427 | 0.0361 | 0.0459 | 0.0032 | 0.0066 | 0.0049 | 0.0034 |
| 37(H) | 0.065 | 0.0505 | 0.0772 | 0.0123 | 0.0145 | 0.0134 | 0.0022 |
| 38(H) | 0.0955 | 0.0889 | 0.0989 | 0.0034 | 0.0066 | 0.005 | 0.0032 |
| 39(H) | 0.0599 | 0.0509 | 0.0915 | 0.0316 | 0.009 | 0.0203 | -0.0225 |
| 40(H) | 0.0585 | 0.0506 | 0.0951 | 0.0366 | 0.0079 | 0.0222 | -0.0288 |
| 41(H) | 0.0527 | 0.0453 | 0.0816 | 0.0289 | 0.0074 | 0.0181 | -0.0215 |
| 42(H) | 0.0585 | 0.0551 | 0.0752 | 0.0168 | 0.0034 | 0.0101 | -0.0134 |
| 43(H) | 0.0507 | 0.0466 | 0.0743 | 0.0235 | 0.0041 | 0.0138 | -0.0194 |
| 44(H) | 0.0443 | 0.0342 | 0.0664 | 0.0222 | 0.01 | 0.0161 | -0.0121 |
| 45(H) | 0.0546 | 0.0523 | 0.0732 | 0.0186 | 0.0023 | 0.0105 | -0.0163 |
| 46(H) | 0.0479 | 0.0454 | 0.074 | 0.026 | 0.0026 | 0.0143 | -0.0235 |
| 47(H) | 0.041 | 0.0381 | 0.0645 | 0.0234 | 0.003 | 0.0132 | -0.0204 |

**Table S23.** QTAIM topological parameters of **1(**Gas phase**)** at nuclear critical points (NCPs), including Electron Density ρ (a.u.), Laplacian of electron density ∇²ρ (a.u), and Average local ionization energy (ALIE) (a.u.) values for the corresponding nuclei, describing the electron distribution and local ionization characteristics of the molecule.

| CP | Corresponding nucleus | Electron Density ρ (a.u.) | Laplacian ∇²ρ (a.u.) | ALIE (a.u.) |
| --- | --- | --- | --- | --- |
| 1 | 38(H) | 0.44 | -25.74 | 0.49 |
| 2 | 32(H) | 0.43 | -25.25 | 0.50 |
| 3 | 23(O) | 295.27 | -2462873.84 | 18.37 |
| 4 | 24(C) | 119.50 | -544114.95 | 9.94 |
| 5 | 12(O) | 295.62 | -2465860.31 | 18.30 |
| 6 | 31(H) | 0.43 | -25.10 | 0.49 |
| 7 | 39(H) | 0.44 | -25.67 | 0.49 |
| 8 | 16(C) | 119.62 | -544670.96 | 9.88 |
| 9 | 27(H) | 0.43 | -25.48 | 0.48 |
| 10 | 40(H) | 0.44 | -25.67 | 0.49 |
| 11 | 17(C) | 119.60 | -544557.05 | 9.94 |
| 12 | 26(H) | 0.43 | -25.39 | 0.49 |
| 13 | 14(C) | 119.59 | -544547.35 | 9.89 |
| 14 | 8(C) | 119.62 | -544657.36 | 9.87 |
| 15 | 2(C) | 119.68 | -544966.12 | 9.98 |
| 16 | 7(C) | 119.62 | -544693.25 | 9.87 |
| 17 | 15(C) | 119.57 | -544445.79 | 9.91 |
| 18 | 28(H) | 0.43 | -25.49 | 0.48 |
| 19 | 9(C) | 119.62 | -544676.20 | 9.87 |
| 20 | 3(C) | 119.60 | -544583.12 | 9.93 |
| 21 | 18(C) | 119.58 | -544500.98 | 9.89 |
| 22 | 6(C) | 119.56 | -544414.34 | 9.92 |
| 23 | 33(H) | 0.43 | -25.44 | 0.51 |
| 24 | 1(N) | 193.97 | -1211276.58 | 13.88 |
| 25 | 10(C) | 119.62 | -544656.74 | 9.87 |
| 26 | 20(C) | 119.58 | -544503.91 | 9.95 |
| 27 | 11(C) | 119.63 | -544706.25 | 9.87 |
| 28 | 19(C) | 119.58 | -544494.07 | 9.89 |
| 29 | 29(H) | 0.43 | -25.48 | 0.48 |
| 30 | 4(N) | 193.93 | -1211062.85 | 13.87 |
| 31 | 30(H) | 0.43 | -25.38 | 0.49 |
| 32 | 5(C) | 119.66 | -544842.59 | 9.99 |
| 33 | 21(O) | 295.20 | -2462339.89 | 18.39 |
| 34 | 25(H) | 0.41 | -23.38 | 0.63 |
| 35 | 34(H) | 0.43 | -25.48 | 0.51 |
| 36 | 37(H) | 0.44 | -25.65 | 0.50 |
| 37 | 22(C) | 119.50 | -544132.55 | 9.95 |
| 38 | 13(S) | 2588.28 | -143783398.50 | 82.58 |
| 39 | 35(H) | 0.44 | -25.65 | 0.50 |
| 40 | 36(H) | 0.44 | -25.69 | 0.50 |

**Table S24.** QTAIM topological parameters of **1(**DMSO phase**)** at nuclear critical points (NCPs), including Electron Density ρ (a.u.), Laplacian of electron density ∇²ρ (a.u), and Average local ionization energy (ALIE) (a.u.) values for the corresponding nuclei, describing the electron distribution and local ionization characteristics of the molecule.

| CP | Corresponding nucleus | Electron Density ρ (a.u.) | Laplacian ∇²ρ (a.u.) | ALIE (a.u.) |
| --- | --- | --- | --- | --- |
| 1 | 29(H) | 0.43 | -25.27 | 0.48 |
| 2 | 10(C) | 119.62 | -544691.13 | 9.87 |
| 3 | 28(H) | 0.43 | -25.29 | 0.48 |
| 4 | 30(H) | 0.43 | -25.12 | 0.49 |
| 5 | 12(O) | 295.57 | -2465416.25 | 18.31 |
| 6 | 32(H) | 0.43 | -25.09 | 0.49 |
| 7 | 38(H) | 0.44 | -25.61 | 0.47 |
| 8 | 9(C) | 119.63 | -544716.86 | 9.87 |
| 9 | 11(C) | 119.64 | -544742.61 | 9.87 |
| 10 | 31(H) | 0.43 | -25.00 | 0.49 |
| 11 | 23(O) | 295.26 | -2462792.90 | 18.36 |
| 12 | 2(C) | 119.68 | -544972.49 | 9.99 |
| 13 | 24(C) | 119.50 | -544139.93 | 9.91 |
| 14 | 8(C) | 119.62 | -544691.10 | 9.87 |
| 15 | 6(C) | 119.56 | -544421.06 | 9.92 |
| 16 | 16(C) | 119.62 | -544676.31 | 9.87 |
| 17 | 40(H) | 0.43 | -25.55 | 0.47 |
| 18 | 14(C) | 119.61 | -544639.40 | 9.89 |
| 19 | 27(H) | 0.43 | -25.27 | 0.48 |
| 20 | 17(C) | 119.59 | -544552.68 | 9.93 |
| 21 | 7(C) | 119.64 | -544743.68 | 9.87 |
| 22 | 1(N) | 193.96 | -1211265.36 | 13.89 |
| 23 | 3(C) | 119.59 | -544533.07 | 9.93 |
| 24 | 39(H) | 0.43 | -25.55 | 0.47 |
| 25 | 15(C) | 119.57 | -544424.66 | 9.90 |
| 26 | 26(H) | 0.43 | -25.12 | 0.49 |
| 27 | 5(C) | 119.66 | -544855.18 | 10.00 |
| 28 | 4(N) | 193.92 | -1211039.13 | 13.88 |
| 29 | 18(C) | 119.60 | -544565.30 | 9.87 |
| 30 | 33(H) | 0.43 | -25.18 | 0.49 |
| 31 | 20(C) | 119.59 | -544530.66 | 9.94 |
| 32 | 25(H) | 0.41 | -23.07 | 0.63 |
| 33 | 13(S) | 2588.18 | -143777705.00 | 82.59 |
| 34 | 19(C) | 119.59 | -544526.42 | 9.87 |
| 35 | 21(O) | 295.21 | -2462389.44 | 18.38 |
| 36 | 34(H) | 0.43 | -25.19 | 0.49 |
| 37 | 22(C) | 119.51 | -544163.99 | 9.92 |
| 38 | 36(H) | 0.43 | -25.52 | 0.47 |
| 39 | 37(H) | 0.43 | -25.52 | 0.47 |
| 40 | 35(H) | 0.44 | -25.53 | 0.48 |

**Table S25.** QTAIM topological parameters of **2a (**Gas phase**)** at nuclear critical points (NCPs), including Electron Density ρ (a.u.), Laplacian of electron density ∇²ρ (a.u), and Average local ionization energy (ALIE) (a.u.) values for the corresponding nuclei, describing the electron distribution and local ionization characteristics of the molecule.

| CP | Corresponding nucleus | Electron Density ρ (a.u.) | Laplacian ∇²ρ (a.u.) | ALIE (a.u.) |
| --- | --- | --- | --- | --- |
| 1 | 48(H) | 0.44 | -25.76 | 0.49 |
| 2 | 49(H) | 0.44 | -25.69 | 0.48 |
| 3 | 29(C) | 119.49 | -544112.37 | 9.93 |
| 4 | 50(H) | 0.44 | -25.69 | 0.48 |
| 5 | 28(O) | 295.23 | -2462593.14 | 18.37 |
| 6 | 40(H) | 0.43 | -25.51 | 0.49 |
| 7 | 19(C) | 119.57 | -544457.16 | 9.87 |
| 8 | 20(C) | 119.60 | -544573.73 | 9.93 |
| 9 | 37(H) | 0.43 | -25.21 | 0.47 |
| 10 | 44(H) | 0.43 | -25.49 | 0.49 |
| 11 | 41(H) | 0.43 | -25.49 | 0.48 |
| 12 | 39(H) | 0.43 | -25.48 | 0.49 |
| 13 | 43(H) | 0.43 | -25.47 | 0.49 |
| 14 | 24(C) | 119.62 | -544676.92 | 9.87 |
| 15 | 18(C) | 119.59 | -544533.36 | 9.87 |
| 16 | 23(C) | 119.63 | -544695.89 | 9.87 |
| 17 | 45(H) | 0.43 | -25.50 | 0.49 |
| 18 | 25(C) | 119.62 | -544673.06 | 9.87 |
| 19 | 42(H) | 0.43 | -25.47 | 0.48 |
| 20 | 15(C) | 119.57 | -544418.26 | 9.88 |
| 21 | 14(C) | 119.65 | -544823.97 | 9.88 |
| 22 | 22(C) | 119.60 | -544565.52 | 9.89 |
| 23 | 21(C) | 119.54 | -544300.24 | 9.91 |
| 24 | 26(C) | 119.62 | -544676.24 | 9.87 |
| 25 | 27(C) | 119.63 | -544696.03 | 9.87 |
| 26 | 46(H) | 0.43 | -25.49 | 0.49 |
| 27 | 47(H) | 0.43 | -25.48 | 0.49 |
| 28 | 4(N) | 194.46 | -1214435.20 | 13.77 |
| 29 | 17(C) | 119.59 | -544548.10 | 9.92 |
| 30 | 3(C) | 119.61 | -544623.91 | 9.90 |
| 31 | 16(C) | 119.63 | -544726.60 | 9.85 |
| 32 | 53(H) | 0.44 | -25.69 | 0.48 |
| 33 | 5(C) | 119.64 | -544783.45 | 9.96 |
| 34 | 52(H) | 0.44 | -25.68 | 0.48 |
| 35 | 13(S) | 2588.38 | -143789364.50 | 82.64 |
| 36 | 38(H) | 0.42 | -24.50 | 0.47 |
| 37 | 31(C) | 119.49 | -544105.55 | 9.92 |
| 38 | 30(O) | 295.28 | -2462937.62 | 18.36 |
| 39 | 2(C) | 119.69 | -544992.46 | 9.97 |
| 40 | 1(N) | 193.95 | -1211165.22 | 13.87 |
| 41 | 51(H) | 0.44 | -25.76 | 0.48 |
| 42 | 12(O) | 295.59 | -2465662.70 | 18.29 |
| 43 | 36(H) | 0.43 | -25.35 | 0.49 |
| 44 | 6(C) | 119.56 | -544418.83 | 9.92 |
| 45 | 32(H) | 0.43 | -25.40 | 0.49 |
| 46 | 11(C) | 119.63 | -544703.39 | 9.87 |
| 47 | 7(C) | 119.62 | -544683.01 | 9.87 |
| 48 | 10(C) | 119.62 | -544671.22 | 9.87 |
| 49 | 8(C) | 119.62 | -544659.81 | 9.87 |
| 50 | 35(H) | 0.43 | -25.48 | 0.49 |
| 51 | 33(H) | 0.43 | -25.48 | 0.49 |
| 52 | 9(C) | 119.62 | -544669.28 | 9.87 |
| 53 | 34(H) | 0.43 | -25.49 | 0.49 |

**Table S26.** QTAIM topological parameters of **2a (**DMSO phase**)** at nuclear critical points (NCPs), including Electron Density ρ (a.u.), Laplacian of electron density ∇²ρ (a.u), and Average local ionization energy (ALIE) (a.u.) values for the corresponding nuclei, describing the electron distribution and local ionization characteristics of the molecule.

| CP | Corresponding nucleus | | Electron Density ρ (a.u.) | Laplacian ∇²ρ (a.u.) | ALIE (a.u.) |
| --- | --- | --- | --- | --- | --- |
| 1 | | 48(H) | 0.44 | -25.60 | 0.47 |
| 2 | | 50(H) | 0.43 | -25.54 | 0.47 |
| 3 | | 49(H) | 0.43 | -25.55 | 0.47 |
| 4 | | 29(C) | 119.50 | -544146.08 | 9.91 |
| 5 | | 28(O) | 295.24 | -2462658.50 | 18.37 |
| 6 | | 40(H) | 0.43 | -25.22 | 0.48 |
| 7 | | 19(C) | 119.58 | -544499.66 | 9.87 |
| 8 | | 20(C) | 119.60 | -544575.53 | 9.93 |
| 9 | | 37(H) | 0.43 | -25.14 | 0.48 |
| 10 | | 44(H) | 0.43 | -25.31 | 0.48 |
| 11 | | 43(H) | 0.43 | -25.28 | 0.48 |
| 12 | | 41(H) | 0.43 | -25.35 | 0.48 |
| 13 | | 24(C) | 119.63 | -544693.18 | 9.87 |
| 14 | | 23(C) | 119.63 | -544714.44 | 9.87 |
| 15 | | 39(H) | 0.43 | -25.21 | 0.49 |
| 16 | | 18(C) | 119.60 | -544568.42 | 9.87 |
| 17 | | 14(C) | 119.65 | -544815.12 | 9.89 |
| 18 | | 15(C) | 119.57 | -544410.74 | 9.89 |
| 19 | | 42(H) | 0.43 | -25.35 | 0.48 |
| 20 | | 45(H) | 0.43 | -25.32 | 0.48 |
| 21 | | 25(C) | 119.63 | -544694.62 | 9.87 |
| 22 | | 22(C) | 119.60 | -544571.19 | 9.89 |
| 23 | | 21(C) | 119.54 | -544317.77 | 9.91 |
| 24 | | 4(N) | 194.42 | -1214219.79 | 13.78 |
| 25 | | 26(C) | 119.63 | -544693.17 | 9.87 |
| 26 | | 27(C) | 119.63 | -544713.58 | 9.87 |
| 27 | | 3(C) | 119.61 | -544637.40 | 9.91 |
| 28 | | 46(H) | 0.43 | -25.31 | 0.48 |
| 29 | | 47(H) | 0.43 | -25.28 | 0.48 |
| 30 | | 17(C) | 119.59 | -544540.37 | 9.93 |
| 31 | | 16(C) | 119.62 | -544671.29 | 9.86 |
| 32 | | 5(C) | 119.65 | -544835.24 | 9.97 |
| 33 | | 52(H) | 0.43 | -25.57 | 0.46 |
| 34 | | 13(S) | 2588.36 | -143787832.00 | 82.65 |
| 35 | | 53(H) | 0.43 | -25.57 | 0.46 |
| 36 | | 38(H) | 0.43 | -25.12 | 0.48 |
| 37 | | 2(C) | 119.69 | -544993.89 | 9.98 |
| 38 | | 30(O) | 295.26 | -2462797.17 | 18.36 |
| 39 | | 31(C) | 119.50 | -544133.98 | 9.91 |
| 40 | | 1(N) | 193.95 | -1211170.52 | 13.88 |
| 41 | | 12(O) | 295.56 | -2465369.10 | 18.30 |
| 42 | | 36(H) | 0.43 | -25.13 | 0.49 |
| 43 | | 51(H) | 0.44 | -25.63 | 0.47 |
| 44 | | 6(C) | 119.56 | -544414.35 | 9.92 |
| 45 | | 11(C) | 119.64 | -544746.10 | 9.87 |
| 46 | | 32(H) | 0.43 | -25.13 | 0.49 |
| 47 | | 7(C) | 119.64 | -544744.12 | 9.87 |
| 48 | | 10(C) | 119.62 | -544693.46 | 9.87 |
| 49 | | 35(H) | 0.43 | -25.27 | 0.48 |
| 50 | | 8(C) | 119.62 | -544690.21 | 9.87 |
| 51 | | 9(C) | 119.63 | -544717.68 | 9.87 |
| 52 | | 33(H) | 0.43 | -25.27 | 0.48 |
| 53 | | 34(H) | 0.43 | -25.29 | 0.48 |

**Table S27.** QTAIM topological parameters of **2b (**Gas phase**)** at nuclear critical points (NCPs), including Electron Density ρ (a.u.), Laplacian of electron density ∇²ρ (a.u), and Average local ionization energy (ALIE) (a.u.) values for the corresponding nuclei, describing the electron distribution and local ionization characteristics of the molecule.

| CP | Corresponding nucleus | Electron Density ρ (a.u.) | Laplacian ∇²ρ (a.u.) | ALIE (a.u.) |
| --- | --- | --- | --- | --- |
| 1 | 47(H) | 0.43 | -25.48 | 0.49 |
| 2 | 55(H) | 0.43 | -25.50 | 0.49 |
| 3 | 28(C) | 119.62 | -544666.98 | 9.87 |
| 4 | 27(C) | 119.64 | -544750.30 | 9.88 |
| 5 | 48(H) | 0.43 | -25.25 | 0.48 |
| 6 | 29(C) | 119.64 | -544768.99 | 9.87 |
| 7 | 49(H) | 0.44 | -25.75 | 0.49 |
| 8 | 51(H) | 0.44 | -25.69 | 0.49 |
| 9 | 26(C) | 119.62 | -544662.11 | 9.87 |
| 10 | 46(H) | 0.43 | -25.46 | 0.49 |
| 11 | 23(C) | 119.61 | -544626.63 | 9.88 |
| 12 | 31(C) | 119.49 | -544114.76 | 9.93 |
| 13 | 25(C) | 119.64 | -544756.11 | 9.87 |
| 14 | 24(O) | 295.67 | -2466281.18 | 18.30 |
| 15 | 22(C) | 119.75 | -545262.04 | 9.95 |
| 16 | 50(H) | 0.44 | -25.68 | 0.49 |
| 17 | 45(H) | 0.43 | -25.17 | 0.49 |
| 18 | 30(O) | 295.23 | -2462576.37 | 18.38 |
| 19 | 44(H) | 0.42 | -24.72 | 0.50 |
| 20 | 42(H) | 0.43 | -25.50 | 0.50 |
| 21 | 21(C) | 119.56 | -544413.47 | 9.91 |
| 22 | 43(H) | 0.43 | -25.40 | 0.49 |
| 23 | 39(H) | 0.43 | -25.21 | 0.48 |
| 24 | 20(C) | 119.60 | -544574.59 | 9.94 |
| 25 | 19(C) | 119.57 | -544459.32 | 9.88 |
| 26 | 14(C) | 119.65 | -544826.12 | 9.88 |
| 27 | 4(N) | 194.43 | -1214248.19 | 13.78 |
| 28 | 15(C) | 119.57 | -544412.51 | 9.89 |
| 29 | 18(C) | 119.59 | -544541.66 | 9.88 |
| 30 | 13(S) | 2588.35 | -143787680.20 | 82.66 |
| 31 | 3(C) | 119.61 | -544607.55 | 9.91 |
| 32 | 41(H) | 0.43 | -25.47 | 0.50 |
| 33 | 5(C) | 119.64 | -544763.07 | 9.98 |
| 34 | 16(C) | 119.63 | -544726.63 | 9.86 |
| 35 | 17(C) | 119.59 | -544547.20 | 9.93 |
| 36 | 2(C) | 119.69 | -544997.25 | 9.98 |
| 37 | 53(H) | 0.44 | -25.69 | 0.48 |
| 38 | 1(N) | 193.95 | -1211179.75 | 13.88 |
| 39 | 40(H) | 0.42 | -24.51 | 0.47 |
| 40 | 38(H) | 0.43 | -25.34 | 0.49 |
| 41 | 32(O) | 295.28 | -2462935.01 | 18.36 |
| 42 | 12(O) | 295.60 | -2465712.34 | 18.30 |
| 43 | 33(C) | 119.49 | -544107.25 | 9.93 |
| 44 | 6(C) | 119.56 | -544415.03 | 9.93 |
| 45 | 54(H) | 0.44 | -25.68 | 0.48 |
| 46 | 11(C) | 119.63 | -544707.10 | 9.88 |
| 47 | 52(H) | 0.44 | -25.75 | 0.48 |
| 48 | 34(H) | 0.43 | -25.39 | 0.50 |
| 49 | 7(C) | 119.62 | -544685.28 | 9.88 |
| 50 | 10(C) | 119.62 | -544674.33 | 9.88 |
| 51 | 37(H) | 0.43 | -25.47 | 0.49 |
| 52 | 8(C) | 119.62 | -544661.82 | 9.88 |
| 53 | 9(C) | 119.62 | -544676.68 | 9.88 |
| 54 | 35(H) | 0.43 | -25.47 | 0.49 |
| 55 | 36(H) | 0.43 | -25.48 | 0.49 |

**Table S28.** QTAIM topological parameters of **2b (**DMSO phase**)** at nuclear critical points (NCPs), including Electron Density ρ (a.u.), Laplacian of electron density ∇²ρ (a.u), and Average local ionization energy (ALIE) (a.u.) values for the corresponding nuclei, describing the electron distribution and local ionization characteristics of the molecule.

| CP | Corresponding nucleus | Electron Density ρ (a.u.) | Laplacian ∇²ρ (a.u.) | ALIE (a.u.) |
| --- | --- | --- | --- | --- |
| 1 | 43(H) | 0.43 | -25.13 | 0.49 |
| 2 | 24(O) | 295.63 | -2465948.43 | 18.31 |
| 3 | 49(H) | 0.44 | -25.59 | 0.47 |
| 4 | 44(H) | 0.42 | -24.98 | 0.49 |
| 5 | 21(C) | 119.57 | -544457.47 | 9.91 |
| 6 | 22(C) | 119.75 | -545299.84 | 9.95 |
| 7 | 51(H) | 0.43 | -25.54 | 0.47 |
| 8 | 45(H) | 0.43 | -25.38 | 0.48 |
| 9 | 31(C) | 119.50 | -544151.36 | 9.91 |
| 10 | 23(C) | 119.61 | -544619.24 | 9.88 |
| 11 | 25(C) | 119.64 | -544756.85 | 9.87 |
| 12 | 50(H) | 0.43 | -25.54 | 0.47 |
| 13 | 30(O) | 295.23 | -2462571.87 | 18.37 |
| 14 | 48(H) | 0.43 | -25.23 | 0.48 |
| 15 | 29(C) | 119.64 | -544760.55 | 9.87 |
| 16 | 39(H) | 0.43 | -25.12 | 0.49 |
| 17 | 26(C) | 119.62 | -544684.69 | 9.87 |
| 18 | 13(S) | 2588.30 | -143784869.60 | 82.65 |
| 19 | 46(H) | 0.43 | -25.28 | 0.48 |
| 20 | 4(N) | 194.46 | -1214458.82 | 13.78 |
| 21 | 28(C) | 119.62 | -544682.84 | 9.87 |
| 22 | 27(C) | 119.64 | -544765.31 | 9.87 |
| 23 | 20(C) | 119.60 | -544597.54 | 9.94 |
| 24 | 5(C) | 119.64 | -544755.58 | 9.97 |
| 25 | 42(H) | 0.43 | -25.21 | 0.49 |
| 26 | 14(C) | 119.66 | -544885.12 | 9.89 |
| 27 | 47(H) | 0.43 | -25.29 | 0.48 |
| 28 | 55(H) | 0.43 | -25.28 | 0.48 |
| 29 | 3(C) | 119.60 | -544598.90 | 9.91 |
| 30 | 19(C) | 119.58 | -544504.85 | 9.87 |
| 31 | 15(C) | 119.56 | -544397.24 | 9.89 |
| 32 | 1(N) | 193.97 | -1211292.97 | 13.88 |
| 33 | 2(C) | 119.68 | -544960.82 | 9.98 |
| 34 | 18(C) | 119.61 | -544610.24 | 9.88 |
| 35 | 38(H) | 0.43 | -25.20 | 0.49 |
| 36 | 16(C) | 119.63 | -544694.50 | 9.86 |
| 37 | 6(C) | 119.56 | -544423.84 | 9.92 |
| 38 | 41(H) | 0.43 | -25.19 | 0.49 |
| 39 | 11(C) | 119.63 | -544737.58 | 9.87 |
| 40 | 40(H) | 0.42 | -24.63 | 0.48 |
| 41 | 17(C) | 119.59 | -544544.39 | 9.93 |
| 42 | 34(H) | 0.43 | -25.15 | 0.49 |
| 43 | 12(O) | 295.55 | -2465301.42 | 18.30 |
| 44 | 7(C) | 119.63 | -544716.91 | 9.87 |
| 45 | 10(C) | 119.62 | -544687.92 | 9.87 |
| 46 | 37(H) | 0.43 | -25.27 | 0.48 |
| 47 | 8(C) | 119.62 | -544685.46 | 9.87 |
| 48 | 53(H) | 0.43 | -25.56 | 0.46 |
| 49 | 9(C) | 119.63 | -544703.32 | 9.87 |
| 50 | 32(O) | 295.26 | -2462801.48 | 18.36 |
| 51 | 35(H) | 0.43 | -25.28 | 0.48 |
| 52 | 36(H) | 0.43 | -25.29 | 0.48 |
| 53 | 33(C) | 119.50 | -544135.19 | 9.91 |
| 54 | 54(H) | 0.43 | -25.56 | 0.46 |
| 55 | 52(H) | 0.44 | -25.62 | 0.47 |

**Table S29.** QTAIM topological parameters of **2c (**Gas phase**)** at nuclear critical points (NCPs), including Electron Density ρ (a.u.), Laplacian of electron density ∇²ρ (a.u), and Average local ionization energy (ALIE) (a.u.) values for the corresponding nuclei, describing the electron distribution and local ionization characteristics of the molecule.

| CP | Corresponding nucleus | Electron Density ρ (a.u.) | Laplacian ∇²ρ (a.u.) | ALIE (a.u.) |
| --- | --- | --- | --- | --- |
| 1 | 55(Cl) | 3122.44 | -195966588.70 | 94.19 |
| 2 | 47(H) | 0.43 | -25.33 | 0.50 |
| 3 | 28(C) | 119.62 | -544660.43 | 9.89 |
| 4 | 27(C) | 119.66 | -544869.87 | 9.94 |
| 5 | 48(H) | 0.43 | -25.18 | 0.49 |
| 6 | 29(C) | 119.64 | -544782.27 | 9.88 |
| 7 | 26(C) | 119.62 | -544653.91 | 9.89 |
| 8 | 46(H) | 0.43 | -25.30 | 0.50 |
| 9 | 51(H) | 0.44 | -25.68 | 0.49 |
| 10 | 49(H) | 0.44 | -25.75 | 0.49 |
| 11 | 23(C) | 119.61 | -544600.75 | 9.89 |
| 12 | 25(C) | 119.64 | -544772.74 | 9.88 |
| 13 | 31(C) | 119.49 | -544115.58 | 9.94 |
| 14 | 50(H) | 0.44 | -25.68 | 0.49 |
| 15 | 45(H) | 0.43 | -25.06 | 0.50 |
| 16 | 24(O) | 295.67 | -2466292.32 | 18.30 |
| 17 | 22(C) | 119.74 | -545250.68 | 9.96 |
| 18 | 30(O) | 295.23 | -2462569.73 | 18.38 |
| 19 | 42(H) | 0.43 | -25.50 | 0.50 |
| 20 | 19(C) | 119.57 | -544460.07 | 9.88 |
| 21 | 20(C) | 119.60 | -544575.21 | 9.94 |
| 22 | 39(H) | 0.43 | -25.21 | 0.48 |
| 23 | 44(H) | 0.42 | -24.73 | 0.50 |
| 24 | 21(C) | 119.56 | -544408.05 | 9.92 |
| 25 | 41(H) | 0.43 | -25.47 | 0.50 |
| 26 | 18(C) | 119.59 | -544545.22 | 9.88 |
| 27 | 43(H) | 0.43 | -25.39 | 0.50 |
| 28 | 14(C) | 119.65 | -544830.28 | 9.89 |
| 29 | 15(C) | 119.56 | -544410.15 | 9.89 |
| 30 | 4(N) | 194.43 | -1214239.34 | 13.78 |
| 31 | 53(H) | 0.44 | -25.68 | 0.48 |
| 32 | 3(C) | 119.61 | -544603.63 | 9.91 |
| 33 | 17(C) | 119.59 | -544547.07 | 9.93 |
| 34 | 16(C) | 119.63 | -544727.68 | 9.86 |
| 35 | 13(S) | 2588.36 | -143788060.30 | 82.66 |
| 36 | 5(C) | 119.64 | -544759.51 | 9.98 |
| 37 | 40(H) | 0.42 | -24.52 | 0.47 |
| 38 | 33(C) | 119.49 | -544107.83 | 9.93 |
| 39 | 32(O) | 295.28 | -2462934.54 | 18.36 |
| 40 | 38(H) | 0.43 | -25.34 | 0.49 |
| 41 | 2(C) | 119.69 | -544996.33 | 9.98 |
| 42 | 1(N) | 193.95 | -1211184.83 | 13.88 |
| 43 | 54(H) | 0.44 | -25.68 | 0.48 |
| 44 | 12(O) | 295.60 | -2465727.35 | 18.30 |
| 45 | 52(H) | 0.44 | -25.75 | 0.48 |
| 46 | 11(C) | 119.63 | -544707.54 | 9.88 |
| 47 | 6(C) | 119.56 | -544414.69 | 9.93 |
| 48 | 37(H) | 0.43 | -25.46 | 0.49 |
| 49 | 10(C) | 119.62 | -544675.84 | 9.88 |
| 50 | 7(C) | 119.62 | -544684.58 | 9.88 |
| 51 | 34(H) | 0.43 | -25.39 | 0.50 |
| 52 | 9(C) | 119.62 | -544678.13 | 9.88 |
| 53 | 8(C) | 119.62 | -544662.37 | 9.88 |
| 54 | 36(H) | 0.43 | -25.48 | 0.49 |
| 55 | 35(H) | 0.43 | -25.46 | 0.49 |

**Table S30.** QTAIM topological parameters of **2c (**DMSO phase**)** at nuclear critical points (NCPs), including Electron Density ρ (a.u.), Laplacian of electron density ∇²ρ (a.u), and Average local ionization energy (ALIE) (a.u.) values for the corresponding nuclei, describing the electron distribution and local ionization characteristics of the molecule.

| CP | Corresponding nucleus | Electron Density ρ (a.u.) | Laplacian ∇²ρ (a.u.) | ALIE (a.u.) |
| --- | --- | --- | --- | --- |
| 1.00 | 43(H ) | 0.42 | -25.01 | 0.49 |
| 2.00 | 45(H ) | 0.43 | -25.21 | 0.49 |
| 3.00 | 24(O ) | 295.63 | -2465977.37 | 18.31 |
| 4.00 | 44(H ) | 0.43 | -25.22 | 0.49 |
| 5.00 | 22(C ) | 119.75 | -545272.69 | 9.96 |
| 6.00 | 21(C ) | 119.57 | -544446.40 | 9.91 |
| 7.00 | 25(C ) | 119.64 | -544751.78 | 9.88 |
| 8.00 | 46(H ) | 0.43 | -25.14 | 0.49 |
| 9.00 | 49(H ) | 0.44 | -25.60 | 0.47 |
| 10.00 | 23(C ) | 119.61 | -544616.25 | 9.89 |
| 11.00 | 26(C ) | 119.62 | -544674.45 | 9.88 |
| 12.00 | 51(H ) | 0.43 | -25.54 | 0.47 |
| 13.00 | 31(C ) | 119.50 | -544147.86 | 9.91 |
| 14.00 | 29(C ) | 119.64 | -544769.08 | 9.88 |
| 15.00 | 27(C ) | 119.66 | -544847.46 | 9.94 |
| 16.00 | 48(H ) | 0.43 | -25.20 | 0.49 |
| 17.00 | 13(S ) | 2588.33 | -143786307.00 | 82.65 |
| 18.00 | 28(C ) | 119.62 | -544669.06 | 9.88 |
| 19.00 | 50(H ) | 0.43 | -25.56 | 0.47 |
| 20.00 | 55(Cl) | 3122.41 | -195964778.40 | 94.18 |
| 21.00 | 30(O ) | 295.23 | -2462550.96 | 18.37 |
| 22.00 | 39(H ) | 0.43 | -25.19 | 0.48 |
| 23.00 | 47(H ) | 0.43 | -25.11 | 0.49 |
| 24.00 | 4(N ) | 194.47 | -1214535.16 | 13.78 |
| 25.00 | 5(C ) | 119.63 | -544728.09 | 9.97 |
| 26.00 | 20(C ) | 119.60 | -544598.47 | 9.93 |
| 27.00 | 14(C ) | 119.67 | -544919.30 | 9.89 |
| 28.00 | 42(H ) | 0.43 | -25.22 | 0.48 |
| 29.00 | 3(C ) | 119.60 | -544582.93 | 9.91 |
| 30.00 | 19(C ) | 119.58 | -544501.11 | 9.87 |
| 31.00 | 1(N ) | 193.97 | -1211309.62 | 13.88 |
| 32.00 | 15(C ) | 119.56 | -544396.58 | 9.89 |
| 33.00 | 38(H ) | 0.43 | -25.24 | 0.49 |
| 34.00 | 2(C ) | 119.68 | -544958.86 | 9.98 |
| 35.00 | 6(C ) | 119.56 | -544426.52 | 9.92 |
| 36.00 | 11(C ) | 119.63 | -544709.81 | 9.87 |
| 37.00 | 34(H ) | 0.43 | -25.17 | 0.49 |
| 38.00 | 7(C ) | 119.63 | -544704.84 | 9.87 |
| 39.00 | 10(C ) | 119.62 | -544692.06 | 9.87 |
| 40.00 | 37(H ) | 0.43 | -25.28 | 0.48 |
| 41.00 | 18(C ) | 119.60 | -544602.96 | 9.87 |
| 42.00 | 16(C ) | 119.62 | -544692.82 | 9.86 |
| 43.00 | 12(O ) | 295.55 | -2465304.77 | 18.30 |
| 44.00 | 8(C ) | 119.62 | -544687.60 | 9.87 |
| 45.00 | 41(H ) | 0.43 | -25.21 | 0.49 |
| 46.00 | 9(C ) | 119.63 | -544696.68 | 9.87 |
| 47.00 | 40(H ) | 0.42 | -24.73 | 0.48 |
| 48.00 | 17(C ) | 119.59 | -544543.71 | 9.93 |
| 49.00 | 35(H ) | 0.43 | -25.28 | 0.48 |
| 50.00 | 36(H ) | 0.43 | -25.29 | 0.48 |
| 51.00 | 53(H ) | 0.43 | -25.56 | 0.46 |
| 52.00 | 32(O ) | 295.26 | -2462800.57 | 18.36 |
| 53.00 | 33(C ) | 119.50 | -544133.43 | 9.91 |
| 54.00 | 54(H ) | 0.43 | -25.56 | 0.46 |
| 55.00 | 52(H ) | 0.44 | -25.62 | 0.47 |

**Table S31.** QTAIM topological parameters of **2d (**Gas phase**)** at nuclear critical points (NCPs), including Electron Density ρ (a.u.), Laplacian of electron density ∇²ρ (a.u), and Average local ionization energy (ALIE) (a.u.) values for the corresponding nuclei, describing the electron distribution and local ionization characteristics of the molecule.

| CP | Corresponding nucleus | Electron Density ρ (a.u.) | Laplacian ∇²ρ (a.u.) | ALIE (a.u.) |
| --- | --- | --- | --- | --- |
| 1 | 51(H) | 0.44 | -25.68 | 0.49 |
| 2 | 49(H) | 0.44 | -25.75 | 0.49 |
| 3 | 31(C) | 119.49 | -544115.60 | 9.94 |
| 4 | 50(H) | 0.44 | -25.68 | 0.49 |
| 5 | 42(H) | 0.43 | -25.50 | 0.50 |
| 6 | 30(O) | 295.23 | -2462569.70 | 18.38 |
| 7 | 55(Br) | 28735.71 | -7608128748.00 | 438.14 |
| 8 | 19(C) | 119.57 | -544460.10 | 9.88 |
| 9 | 20(C) | 119.60 | -544575.25 | 9.94 |
| 10 | 46(H) | 0.43 | -25.33 | 0.50 |
| 11 | 27(C) | 119.68 | -544948.91 | 9.94 |
| 12 | 41(H) | 0.43 | -25.47 | 0.50 |
| 13 | 47(H) | 0.43 | -25.35 | 0.50 |
| 14 | 26(C) | 119.62 | -544668.28 | 9.89 |
| 15 | 18(C) | 119.59 | -544545.30 | 9.88 |
| 16 | 28(C) | 119.62 | -544673.81 | 9.89 |
| 17 | 39(H) | 0.43 | -25.21 | 0.48 |
| 18 | 25(C) | 119.64 | -544775.70 | 9.88 |
| 19 | 29(C) | 119.65 | -544785.31 | 9.88 |
| 20 | 45(H) | 0.43 | -25.04 | 0.50 |
| 21 | 15(C) | 119.56 | -544410.02 | 9.89 |
| 22 | 23(C) | 119.61 | -544610.21 | 9.89 |
| 23 | 48(H) | 0.43 | -25.18 | 0.49 |
| 24 | 53(H) | 0.44 | -25.68 | 0.48 |
| 25 | 14(C) | 119.65 | -544830.56 | 9.89 |
| 26 | 17(C) | 119.59 | -544546.99 | 9.93 |
| 27 | 44(H) | 0.42 | -24.72 | 0.50 |
| 28 | 22(C) | 119.74 | -545250.14 | 9.96 |
| 29 | 24(O) | 295.67 | -2466300.01 | 18.30 |
| 30 | 16(C) | 119.63 | -544727.59 | 9.86 |
| 31 | 54(H) | 0.44 | -25.68 | 0.48 |
| 32 | 33(C) | 119.49 | -544107.81 | 9.93 |
| 33 | 21(C) | 119.56 | -544405.83 | 9.92 |
| 34 | 4(N) | 194.43 | -1214235.12 | 13.78 |
| 35 | 3(C) | 119.61 | -544602.98 | 9.91 |
| 36 | 32(O) | 295.28 | -2462934.41 | 18.36 |
| 37 | 43(H) | 0.43 | -25.39 | 0.50 |
| 38 | 40(H) | 0.42 | -24.52 | 0.47 |
| 39 | 52(H) | 0.44 | -25.75 | 0.48 |
| 40 | 5(C) | 119.64 | -544759.24 | 9.98 |
| 41 | 2(C) | 119.69 | -544996.63 | 9.98 |
| 42 | 13(S) | 2588.36 | -143788082.50 | 82.66 |
| 43 | 12(O) | 295.60 | -2465728.30 | 18.30 |
| 44 | 1(N) | 193.95 | -1211183.77 | 13.88 |
| 45 | 38(H) | 0.43 | -25.34 | 0.49 |
| 46 | 6(C) | 119.56 | -544414.39 | 9.93 |
| 47 | 11(C) | 119.63 | -544707.59 | 9.88 |
| 48 | 34(H) | 0.43 | -25.39 | 0.50 |
| 49 | 7(C) | 119.62 | -544684.74 | 9.88 |
| 50 | 10(C) | 119.62 | -544675.77 | 9.88 |
| 51 | 37(H) | 0.43 | -25.46 | 0.49 |
| 52 | 8(C) | 119.62 | -544662.33 | 9.88 |
| 53 | 9(C) | 119.62 | -544678.27 | 9.88 |
| 54 | 35(H) | 0.43 | -25.46 | 0.49 |
| 55 | 36(H) | 0.43 | -25.48 | 0.49 |

**Table S32.** QTAIM topological parameters of **2d (**DMSO phase**)** at nuclear critical points (NCPs), including Electron Density ρ (a.u.), Laplacian of electron density ∇²ρ (a.u), and Average local ionization energy (ALIE) (a.u.) values for the corresponding nuclei, describing the electron distribution and local ionization characteristics of the molecule.

| CP | Corresponding nucleus | Electron Density ρ (a.u.) | Laplacian ∇²ρ (a.u.) | ALIE (a.u.) |
| --- | --- | --- | --- | --- |
| 1 | 49(H) | 0.44 | -25.60 | 0.47 |
| 2 | 45(H) | 0.43 | -25.22 | 0.49 |
| 3 | 44(H) | 0.43 | -25.29 | 0.49 |
| 4 | 51(H) | 0.43 | -25.55 | 0.47 |
| 5 | 31(C) | 119.50 | -544148.02 | 9.91 |
| 6 | 43(H) | 0.42 | -24.99 | 0.49 |
| 7 | 46(H) | 0.43 | -25.17 | 0.49 |
| 8 | 50(H) | 0.43 | -25.55 | 0.47 |
| 9 | 21(C) | 119.57 | -544429.26 | 9.91 |
| 10 | 25(C) | 119.64 | -544769.88 | 9.88 |
| 11 | 26(C) | 119.62 | -544681.00 | 9.88 |
| 12 | 30(O) | 295.23 | -2462542.47 | 18.37 |
| 13 | 39(H) | 0.43 | -25.17 | 0.48 |
| 14 | 22(C) | 119.75 | -545259.62 | 9.96 |
| 15 | 23(C) | 119.61 | -544621.41 | 9.89 |
| 16 | 13(S) | 2588.32 | -143785738.80 | 82.65 |
| 17 | 24(O) | 295.63 | -2465949.83 | 18.31 |
| 18 | 4(N) | 194.48 | -1214593.81 | 13.78 |
| 19 | 27(C) | 119.67 | -544926.43 | 9.93 |
| 20 | 42(H) | 0.43 | -25.24 | 0.48 |
| 21 | 20(C) | 119.61 | -544605.66 | 9.93 |
| 22 | 55(Br) | 28735.71 | -7608128047.00 | 438.13 |
| 23 | 14(C) | 119.67 | -544926.05 | 9.89 |
| 24 | 5(C) | 119.63 | -544724.58 | 9.97 |
| 25 | 19(C) | 119.58 | -544501.18 | 9.87 |
| 26 | 29(C) | 119.64 | -544779.92 | 9.88 |
| 27 | 3(C) | 119.60 | -544575.11 | 9.91 |
| 28 | 15(C) | 119.56 | -544392.05 | 9.89 |
| 29 | 28(C) | 119.62 | -544677.98 | 9.88 |
| 30 | 48(H) | 0.43 | -25.18 | 0.49 |
| 31 | 1(N) | 193.97 | -1211331.23 | 13.88 |
| 32 | 47(H) | 0.43 | -25.16 | 0.49 |
| 33 | 18(C) | 119.61 | -544612.73 | 9.87 |
| 34 | 2(C) | 119.68 | -544953.89 | 9.98 |
| 35 | 16(C) | 119.63 | -544701.18 | 9.86 |
| 36 | 41(H) | 0.43 | -25.22 | 0.49 |
| 37 | 34(H) | 0.43 | -25.17 | 0.49 |
| 38 | 6(C) | 119.56 | -544425.81 | 9.92 |
| 39 | 17(C) | 119.59 | -544545.89 | 9.92 |
| 40 | 40(H) | 0.42 | -24.65 | 0.48 |
| 41 | 7(C) | 119.63 | -544709.56 | 9.87 |
| 42 | 12(O) | 295.55 | -2465270.88 | 18.30 |
| 43 | 38(H) | 0.43 | -25.22 | 0.49 |
| 44 | 11(C) | 119.63 | -544711.32 | 9.87 |
| 45 | 8(C) | 119.62 | -544687.70 | 9.87 |
| 46 | 35(H) | 0.43 | -25.28 | 0.48 |
| 47 | 32(O) | 295.26 | -2462782.02 | 18.36 |
| 48 | 53(H) | 0.43 | -25.57 | 0.46 |
| 49 | 10(C) | 119.62 | -544692.01 | 9.87 |
| 50 | 54(H) | 0.43 | -25.56 | 0.46 |
| 51 | 9(C) | 119.63 | -544697.51 | 9.87 |
| 52 | 33(C) | 119.50 | -544131.02 | 9.91 |
| 53 | 37(H) | 0.43 | -25.28 | 0.48 |
| 54 | 36(H) | 0.43 | -25.29 | 0.48 |
| 55 | 52(H) | 0.44 | -25.63 | 0.47 |

**Table S33.** QTAIM topological parameters of **2e (**Gas phase**)** at nuclear critical points (NCPs), including Electron Density ρ (a.u.), Laplacian of electron density ∇²ρ (a.u), and Average local ionization energy (ALIE) (a.u.) values for the corresponding nuclei, describing the electron distribution and local ionization characteristics of the molecule.

| CP | Corresponding nucleus | Electron Density ρ (a.u.) | Laplacian ∇²ρ (a.u.) | ALIE (a.u.) |
| --- | --- | --- | --- | --- |
| 1 | 34(O) | 295.89 | -2468150.20 | 18.33 |
| 2 | 52(H) | 0.44 | -25.68 | 0.49 |
| 3 | 33(O) | 295.89 | -2468137.49 | 18.33 |
| 4 | 32(N) | 194.30 | -1213449.39 | 14.04 |
| 5 | 50(H) | 0.44 | -25.75 | 0.49 |
| 6 | 47(H) | 0.43 | -25.08 | 0.51 |
| 7 | 29(C) | 119.49 | -544115.27 | 9.94 |
| 8 | 51(H) | 0.44 | -25.68 | 0.49 |
| 9 | 25(C) | 119.63 | -544710.29 | 9.93 |
| 10 | 24(C) | 119.64 | -544741.21 | 9.89 |
| 11 | 43(H) | 0.43 | -25.50 | 0.50 |
| 12 | 28(O) | 295.23 | -2462568.84 | 18.38 |
| 13 | 48(H) | 0.43 | -25.09 | 0.51 |
| 14 | 19(C) | 119.57 | -544459.91 | 9.88 |
| 15 | 26(C) | 119.63 | -544725.90 | 9.90 |
| 16 | 23(C) | 119.63 | -544729.94 | 9.89 |
| 17 | 20(C) | 119.60 | -544574.57 | 9.94 |
| 18 | 46(H) | 0.43 | -25.21 | 0.50 |
| 19 | 42(H) | 0.43 | -25.47 | 0.50 |
| 20 | 18(C) | 119.59 | -544543.30 | 9.88 |
| 21 | 40(H) | 0.43 | -25.24 | 0.48 |
| 22 | 27(C) | 119.62 | -544672.15 | 9.89 |
| 23 | 22(C) | 119.63 | -544709.97 | 9.91 |
| 24 | 15(C) | 119.57 | -544411.98 | 9.89 |
| 25 | 54(H) | 0.44 | -25.68 | 0.48 |
| 26 | 14(C) | 119.65 | -544833.12 | 9.89 |
| 27 | 49(H) | 0.43 | -25.43 | 0.51 |
| 28 | 17(C) | 119.59 | -544547.49 | 9.93 |
| 29 | 16(C) | 119.63 | -544728.24 | 9.86 |
| 30 | 45(H) | 0.43 | -25.23 | 0.50 |
| 31 | 21(C) | 119.53 | -544253.45 | 9.93 |
| 32 | 31(C) | 119.49 | -544107.51 | 9.93 |
| 33 | 4(N) | 194.44 | -1214330.98 | 13.78 |
| 34 | 55(H) | 0.44 | -25.69 | 0.48 |
| 35 | 3(C) | 119.61 | -544608.35 | 9.91 |
| 36 | 30(O) | 295.28 | -2462935.12 | 18.36 |
| 37 | 44(H) | 0.43 | -25.44 | 0.51 |
| 38 | 41(H) | 0.42 | -24.52 | 0.47 |
| 39 | 53(H) | 0.44 | -25.75 | 0.48 |
| 40 | 5(C) | 119.64 | -544766.28 | 9.98 |
| 41 | 2(C) | 119.69 | -544994.32 | 9.98 |
| 42 | 13(S) | 2588.34 | -143787171.20 | 82.66 |
| 43 | 35(H) | 0.43 | -25.38 | 0.50 |
| 44 | 12(O) | 295.60 | -2465715.49 | 18.30 |
| 45 | 1(N) | 193.95 | -1211165.88 | 13.88 |
| 46 | 7(C) | 119.62 | -544695.75 | 9.88 |
| 47 | 6(C) | 119.56 | -544412.79 | 9.93 |
| 48 | 36(H) | 0.43 | -25.47 | 0.49 |
| 49 | 8(C) | 119.62 | -544662.03 | 9.88 |
| 50 | 11(C) | 119.63 | -544710.16 | 9.88 |
| 51 | 39(H) | 0.43 | -25.35 | 0.49 |
| 52 | 9(C) | 119.62 | -544678.52 | 9.88 |
| 53 | 10(C) | 119.62 | -544671.50 | 9.88 |
| 54 | 37(H) | 0.43 | -25.48 | 0.49 |
| 55 | 38(H) | 0.43 | -25.46 | 0.49 |

**Table S34.** QTAIM topological parameters of **2e (**DMSO phase**)** at nuclear critical points (NCPs), including Electron Density ρ (a.u.), Laplacian of electron density ∇²ρ (a.u), and Average local ionization energy (ALIE) (a.u.) values for the corresponding nuclei, describing the electron distribution and local ionization characteristics of the molecule.

| CP | Corresponding nucleus | Electron Density ρ (a.u.) | Laplacian ∇²ρ (a.u.) | ALIE (a.u.) |
| --- | --- | --- | --- | --- |
| 1 | 34(O) | 295.86 | -2467887.44 | 18.33 |
| 2 | 33(O) | 295.86 | -2467889.59 | 18.33 |
| 3 | 32(N) | 194.31 | -1213512.00 | 14.04 |
| 4 | 47(H) | 0.43 | -24.98 | 0.50 |
| 5 | 25(C) | 119.62 | -544671.40 | 9.93 |
| 6 | 52(H) | 0.43 | -25.54 | 0.47 |
| 7 | 24(C) | 119.64 | -544744.03 | 9.89 |
| 8 | 50(H) | 0.44 | -25.60 | 0.47 |
| 9 | 48(H) | 0.43 | -24.98 | 0.50 |
| 10 | 26(C) | 119.63 | -544739.14 | 9.89 |
| 11 | 29(C) | 119.50 | -544148.00 | 9.91 |
| 12 | 23(C) | 119.64 | -544745.67 | 9.88 |
| 13 | 51(H) | 0.43 | -25.55 | 0.47 |
| 14 | 46(H) | 0.43 | -25.24 | 0.49 |
| 15 | 43(H) | 0.43 | -25.23 | 0.49 |
| 16 | 28(O) | 295.23 | -2462562.50 | 18.37 |
| 17 | 27(C) | 119.63 | -544697.15 | 9.88 |
| 18 | 22(C) | 119.63 | -544735.90 | 9.90 |
| 19 | 19(C) | 119.58 | -544503.67 | 9.87 |
| 20 | 20(C) | 119.60 | -544585.65 | 9.93 |
| 21 | 49(H) | 0.43 | -25.19 | 0.49 |
| 22 | 40(H) | 0.43 | -25.15 | 0.48 |
| 23 | 42(H) | 0.43 | -25.20 | 0.49 |
| 24 | 18(C) | 119.60 | -544591.58 | 9.87 |
| 25 | 15(C) | 119.56 | -544403.22 | 9.89 |
| 26 | 21(C) | 119.53 | -544272.92 | 9.92 |
| 27 | 45(H) | 0.43 | -25.25 | 0.48 |
| 28 | 14(C) | 119.65 | -544830.33 | 9.89 |
| 29 | 44(H) | 0.43 | -25.03 | 0.49 |
| 30 | 54(H) | 0.43 | -25.56 | 0.46 |
| 31 | 4(N) | 194.44 | -1214311.78 | 13.78 |
| 32 | 17(C) | 119.59 | -544546.72 | 9.93 |
| 33 | 16(C) | 119.62 | -544682.87 | 9.86 |
| 34 | 3(C) | 119.61 | -544610.20 | 9.91 |
| 35 | 31(C) | 119.50 | -544133.93 | 9.91 |
| 36 | 5(C) | 119.65 | -544803.09 | 9.97 |
| 37 | 30(O) | 295.26 | -2462802.43 | 18.36 |
| 38 | 41(H) | 0.42 | -24.65 | 0.48 |
| 39 | 13(S) | 2588.32 | -143786005.60 | 82.65 |
| 40 | 55(H) | 0.43 | -25.56 | 0.46 |
| 41 | 2(C) | 119.68 | -544973.64 | 9.98 |
| 42 | 35(H) | 0.43 | -25.14 | 0.49 |
| 43 | 53(H) | 0.44 | -25.62 | 0.47 |
| 44 | 1(N) | 193.95 | -1211184.04 | 13.88 |
| 45 | 12(O) | 295.55 | -2465304.89 | 18.30 |
| 46 | 7(C) | 119.63 | -544738.20 | 9.87 |
| 47 | 6(C) | 119.56 | -544417.52 | 9.92 |
| 48 | 36(H) | 0.43 | -25.27 | 0.48 |
| 49 | 8(C) | 119.63 | -544696.29 | 9.87 |
| 50 | 11(C) | 119.63 | -544733.52 | 9.88 |
| 51 | 39(H) | 0.43 | -25.13 | 0.49 |
| 52 | 9(C) | 119.63 | -544714.88 | 9.87 |
| 53 | 10(C) | 119.62 | -544690.60 | 9.87 |
| 54 | 37(H) | 0.43 | -25.28 | 0.48 |
| 55 | 38(H) | 0.43 | -25.27 | 0.48 |

**Table S35.** QTAIM topological parameters of **2f (**Gas phase**)** at nuclear critical points (NCPs), including Electron Density ρ (a.u.), Laplacian of electron density ∇²ρ (a.u), and Average local ionization energy (ALIE) (a.u.) values for the corresponding nuclei, describing the electron distribution and local ionization characteristics of the molecule.

| CP | Corresponding nucleus | Electron Density ρ (a.u.) | Laplacian ∇²ρ (a.u.) | ALIE (a.u.) |
| --- | --- | --- | --- | --- |
| 1 | 38(H) | 0.43 | -25.02 | 0.51 |
| 2 | 21(C) | 119.54 | -544296.21 | 9.92 |
| 3 | 45(O) | 295.35 | -2463538.32 | 18.36 |
| 4 | 37(H) | 0.43 | -25.25 | 0.50 |
| 5 | 26(C) | 119.70 | -545030.89 | 9.99 |
| 6 | 42(H) | 0.44 | -25.70 | 0.50 |
| 7 | 27(O) | 295.62 | -2465839.04 | 18.29 |
| 8 | 46(H) | 0.39 | -22.00 | 0.67 |
| 9 | 13(S) | 2588.31 | -143785417.40 | 82.66 |
| 10 | 44(H) | 0.44 | -25.70 | 0.49 |
| 11 | 25(C) | 119.50 | -544118.06 | 9.94 |
| 12 | 24(O) | 295.21 | -2462379.78 | 18.39 |
| 13 | 31(H) | 0.43 | -25.47 | 0.49 |
| 14 | 43(H) | 0.44 | -25.68 | 0.49 |
| 15 | 32(H) | 0.43 | -25.36 | 0.49 |
| 16 | 5(C) | 119.65 | -544815.59 | 9.97 |
| 17 | 10(C) | 119.62 | -544664.65 | 9.88 |
| 18 | 11(C) | 119.62 | -544674.09 | 9.88 |
| 19 | 34(H) | 0.43 | -25.14 | 0.48 |
| 20 | 4(N) | 194.42 | -1214217.77 | 13.77 |
| 21 | 17(C) | 119.59 | -544526.15 | 9.93 |
| 22 | 30(H) | 0.43 | -25.48 | 0.49 |
| 23 | 9(C) | 119.62 | -544667.56 | 9.87 |
| 24 | 6(C) | 119.56 | -544420.93 | 9.93 |
| 25 | 16(C) | 119.63 | -544704.11 | 9.87 |
| 26 | 1(N) | 193.94 | -1211142.38 | 13.88 |
| 27 | 35(H) | 0.43 | -25.46 | 0.50 |
| 28 | 18(C) | 119.58 | -544509.93 | 9.88 |
| 29 | 8(C) | 119.62 | -544674.60 | 9.87 |
| 30 | 7(C) | 119.62 | -544692.64 | 9.87 |
| 31 | 3(C) | 119.62 | -544682.05 | 9.90 |
| 32 | 29(H) | 0.43 | -25.47 | 0.49 |
| 33 | 28(H) | 0.43 | -25.34 | 0.49 |
| 34 | 15(C) | 119.58 | -544457.95 | 9.89 |
| 35 | 2(C) | 119.70 | -545070.58 | 9.97 |
| 36 | 14(C) | 119.63 | -544738.55 | 9.88 |
| 37 | 19(C) | 119.57 | -544448.66 | 9.88 |
| 38 | 20(C) | 119.60 | -544577.15 | 9.94 |
| 39 | 36(H) | 0.43 | -25.48 | 0.50 |
| 40 | 12(O) | 295.61 | -2465812.44 | 18.29 |
| 41 | 33(H) | 0.43 | -25.07 | 0.48 |
| 42 | 22(O) | 295.25 | -2462727.19 | 18.37 |
| 43 | 40(H) | 0.44 | -25.69 | 0.49 |
| 44 | 23(C) | 119.50 | -544115.65 | 9.93 |
| 45 | 41(H) | 0.44 | -25.67 | 0.49 |
| 46 | 39(H) | 0.44 | -25.73 | 0.49 |

**Table S36.** QTAIM topological parameters of **2f (**DMSO phase**)** at nuclear critical points (NCPs), including Electron Density ρ (a.u.), Laplacian of electron density ∇²ρ (a.u), and Average local ionization energy (ALIE) (a.u.) values for the corresponding nuclei, describing the electron distribution and local ionization characteristics of the molecule.

| CP | Corresponding nucleus | Electron Density ρ (a.u.) | Laplacian ∇²ρ (a.u.) | ALIE (a.u.) |
| --- | --- | --- | --- | --- |
| 1 | 42(H) | 0.44 | -25.54 | 0.47 |
| 2 | 25(C) | 119.50 | -544157.56 | 9.92 |
| 3 | 43(H) | 0.43 | -25.54 | 0.47 |
| 4 | 38(H) | 0.42 | -24.63 | 0.50 |
| 5 | 27(O) | 295.57 | -2465457.09 | 18.30 |
| 6 | 44(H) | 0.43 | -25.54 | 0.47 |
| 7 | 46(H) | 0.39 | -21.70 | 0.67 |
| 8 | 26(C) | 119.70 | -545052.00 | 10.00 |
| 9 | 45(O) | 295.34 | -2463460.42 | 18.37 |
| 10 | 24(O) | 295.21 | -2462352.82 | 18.38 |
| 11 | 21(C) | 119.54 | -544283.12 | 9.92 |
| 12 | 37(H) | 0.43 | -25.11 | 0.49 |
| 13 | 13(S) | 2588.30 | -143784728.00 | 82.66 |
| 14 | 17(C) | 119.59 | -544529.32 | 9.93 |
| 15 | 34(H) | 0.43 | -25.23 | 0.48 |
| 16 | 35(H) | 0.43 | -25.15 | 0.49 |
| 17 | 32(H) | 0.43 | -25.12 | 0.49 |
| 18 | 16(C) | 119.62 | -544682.06 | 9.87 |
| 19 | 18(C) | 119.60 | -544568.21 | 9.88 |
| 20 | 31(H) | 0.43 | -25.26 | 0.48 |
| 21 | 5(C) | 119.66 | -544852.93 | 9.98 |
| 22 | 4(N) | 194.41 | -1214140.39 | 13.78 |
| 23 | 11(C) | 119.63 | -544733.66 | 9.88 |
| 24 | 10(C) | 119.62 | -544692.68 | 9.87 |
| 25 | 1(N) | 193.94 | -1211137.73 | 13.88 |
| 26 | 15(C) | 119.57 | -544433.11 | 9.89 |
| 27 | 19(C) | 119.58 | -544491.56 | 9.87 |
| 28 | 6(C) | 119.56 | -544415.06 | 9.93 |
| 29 | 3(C) | 119.62 | -544670.51 | 9.91 |
| 30 | 9(C) | 119.63 | -544714.89 | 9.87 |
| 31 | 30(H) | 0.43 | -25.28 | 0.48 |
| 32 | 36(H) | 0.43 | -25.20 | 0.49 |
| 33 | 14(C) | 119.64 | -544750.22 | 9.89 |
| 34 | 20(C) | 119.60 | -544584.45 | 9.93 |
| 35 | 2(C) | 119.70 | -545050.56 | 9.98 |
| 36 | 7(C) | 119.63 | -544736.47 | 9.87 |
| 37 | 8(C) | 119.63 | -544696.83 | 9.87 |
| 38 | 33(H) | 0.43 | -25.03 | 0.49 |
| 39 | 28(H) | 0.43 | -25.13 | 0.49 |
| 40 | 12(O) | 295.56 | -2465358.29 | 18.30 |
| 41 | 29(H) | 0.43 | -25.26 | 0.48 |
| 42 | 22(O) | 295.24 | -2462682.71 | 18.37 |
| 43 | 41(H) | 0.43 | -25.54 | 0.47 |
| 44 | 40(H) | 0.43 | -25.55 | 0.47 |
| 45 | 23(C) | 119.50 | -544147.50 | 9.91 |
| 46 | 39(H) | 0.44 | -25.60 | 0.47 |

**Table S37.** QTAIM topological parameters of **2g (**Gas phase**)** at nuclear critical points (NCPs), including Electron Density ρ (a.u.), Laplacian of electron density ∇²ρ (a.u), and Average local ionization energy (ALIE) (a.u.) values for the corresponding nuclei, describing the electron distribution and local ionization characteristics of the molecule.

| CP | Corresponding nucleus | Electron Density ρ (a.u.) | Laplacian ∇²ρ (a.u.) | ALIE (a.u.) |
| --- | --- | --- | --- | --- |
| 1 | 50(H) | 0.43 | -25.44 | 0.46 |
| 2 | 51(H) | 0.43 | -25.42 | 0.46 |
| 3 | 52(H) | 0.43 | -25.32 | 0.47 |
| 4 | 49(C) | 119.47 | -543993.53 | 9.87 |
| 5 | 46(C) | 119.52 | -544212.94 | 9.92 |
| 6 | 38(H) | 0.43 | -25.04 | 0.51 |
| 7 | 27(O) | 295.61 | -2465764.68 | 18.30 |
| 8 | 48(H) | 0.44 | -25.78 | 0.47 |
| 9 | 45(O) | 295.44 | -2464281.24 | 18.36 |
| 10 | 47(H) | 0.44 | -25.72 | 0.47 |
| 11 | 42(H) | 0.44 | -25.76 | 0.49 |
| 12 | 26(C) | 119.70 | -545038.26 | 9.99 |
| 13 | 21(C) | 119.54 | -544302.72 | 9.92 |
| 14 | 43(H) | 0.44 | -25.71 | 0.48 |
| 15 | 25(C) | 119.49 | -544105.43 | 9.93 |
| 16 | 37(H) | 0.43 | -25.23 | 0.49 |
| 17 | 24(O) | 295.27 | -2462876.63 | 18.37 |
| 18 | 13(S) | 2588.31 | -143785244.00 | 82.65 |
| 19 | 44(H) | 0.44 | -25.69 | 0.48 |
| 20 | 32(H) | 0.43 | -25.33 | 0.49 |
| 21 | 31(H) | 0.43 | -25.47 | 0.49 |
| 22 | 34(H) | 0.43 | -24.88 | 0.47 |
| 23 | 17(C) | 119.59 | -544538.52 | 9.93 |
| 24 | 5(C) | 119.65 | -544827.93 | 9.97 |
| 25 | 4(N) | 194.41 | -1214156.90 | 13.78 |
| 26 | 16(C) | 119.62 | -544670.43 | 9.86 |
| 27 | 35(H) | 0.43 | -25.48 | 0.50 |
| 28 | 11(C) | 119.62 | -544689.30 | 9.87 |
| 29 | 10(C) | 119.62 | -544663.70 | 9.87 |
| 30 | 18(C) | 119.59 | -544520.19 | 9.88 |
| 31 | 1(N) | 193.94 | -1211127.21 | 13.88 |
| 32 | 6(C) | 119.56 | -544417.08 | 9.92 |
| 33 | 3(C) | 119.62 | -544654.42 | 9.90 |
| 34 | 15(C) | 119.57 | -544441.90 | 9.89 |
| 35 | 9(C) | 119.62 | -544671.85 | 9.87 |
| 36 | 30(H) | 0.43 | -25.49 | 0.49 |
| 37 | 19(C) | 119.57 | -544462.30 | 9.88 |
| 38 | 14(C) | 119.63 | -544740.45 | 9.87 |
| 39 | 2(C) | 119.71 | -545082.01 | 9.96 |
| 40 | 20(C) | 119.60 | -544576.82 | 9.93 |
| 41 | 36(H) | 0.43 | -25.50 | 0.50 |
| 42 | 7(C) | 119.63 | -544702.30 | 9.87 |
| 43 | 8(C) | 119.62 | -544670.59 | 9.87 |
| 44 | 33(H) | 0.43 | -25.06 | 0.48 |
| 45 | 12(O) | 295.61 | -2465770.98 | 18.29 |
| 46 | 28(H) | 0.43 | -25.34 | 0.49 |
| 47 | 22(O) | 295.24 | -2462634.33 | 18.37 |
| 48 | 29(H) | 0.43 | -25.47 | 0.49 |
| 49 | 41(H) | 0.44 | -25.68 | 0.48 |
| 50 | 23(C) | 119.49 | -544114.21 | 9.93 |
| 51 | 40(H) | 0.44 | -25.68 | 0.48 |
| 52 | 39(H) | 0.44 | -25.74 | 0.49 |

**Table S38.** QTAIM topological parameters of **2g (**DMSO phase**)** at nuclear critical points (NCPs), including Electron Density ρ (a.u.), Laplacian of electron density ∇²ρ (a.u), and Average local ionization energy (ALIE) (a.u.) values for the corresponding nuclei, describing the electron distribution and local ionization characteristics of the molecule.

| CP | Corresponding nucleus | Electron Density ρ (a.u.) | Laplacian ∇²ρ (a.u.) | ALIE (a.u.) |
| --- | --- | --- | --- | --- |
| 1 | 50(H) | 0.43 | -25.34 | 0.45 |
| 2 | 51(H) | 0.43 | -25.42 | 0.45 |
| 3 | 52(H) | 0.43 | -25.19 | 0.46 |
| 4 | 49(C) | 119.47 | -543979.95 | 9.86 |
| 5 | 46(C) | 119.52 | -544235.96 | 9.92 |
| 6 | 38(H) | 0.42 | -24.66 | 0.50 |
| 7 | 48(H) | 0.44 | -25.73 | 0.46 |
| 8 | 27(O) | 295.57 | -2465413.67 | 18.30 |
| 9 | 45(O) | 295.43 | -2464224.52 | 18.37 |
| 10 | 47(H) | 0.44 | -25.70 | 0.46 |
| 11 | 26(C) | 119.70 | -545049.06 | 9.99 |
| 12 | 42(H) | 0.44 | -25.62 | 0.47 |
| 13 | 21(C) | 119.54 | -544296.60 | 9.92 |
| 14 | 43(H) | 0.43 | -25.56 | 0.47 |
| 15 | 37(H) | 0.43 | -25.11 | 0.49 |
| 16 | 25(C) | 119.50 | -544132.62 | 9.91 |
| 17 | 13(S) | 2588.29 | -143784110.00 | 82.66 |
| 18 | 24(O) | 295.26 | -2462801.69 | 18.36 |
| 19 | 44(H) | 0.43 | -25.57 | 0.46 |
| 20 | 34(H) | 0.43 | -24.96 | 0.48 |
| 21 | 32(H) | 0.43 | -25.12 | 0.49 |
| 22 | 17(C) | 119.59 | -544542.89 | 9.93 |
| 23 | 5(C) | 119.66 | -544866.52 | 9.98 |
| 24 | 31(H) | 0.43 | -25.26 | 0.48 |
| 25 | 4(N) | 194.41 | -1214107.34 | 13.78 |
| 26 | 35(H) | 0.43 | -25.20 | 0.49 |
| 27 | 16(C) | 119.61 | -544645.65 | 9.86 |
| 28 | 11(C) | 119.64 | -544743.90 | 9.88 |
| 29 | 18(C) | 119.60 | -544577.10 | 9.87 |
| 30 | 10(C) | 119.62 | -544692.09 | 9.87 |
| 31 | 1(N) | 193.94 | -1211121.44 | 13.88 |
| 32 | 6(C) | 119.56 | -544411.94 | 9.92 |
| 33 | 3(C) | 119.61 | -544642.73 | 9.91 |
| 34 | 15(C) | 119.57 | -544416.22 | 9.89 |
| 35 | 9(C) | 119.63 | -544718.64 | 9.87 |
| 36 | 30(H) | 0.43 | -25.28 | 0.48 |
| 37 | 19(C) | 119.58 | -544505.01 | 9.87 |
| 38 | 14(C) | 119.63 | -544745.44 | 9.88 |
| 39 | 2(C) | 119.70 | -545059.55 | 9.97 |
| 40 | 7(C) | 119.64 | -544745.13 | 9.87 |
| 41 | 20(C) | 119.60 | -544582.49 | 9.93 |
| 42 | 36(H) | 0.43 | -25.23 | 0.48 |
| 43 | 8(C) | 119.63 | -544694.50 | 9.87 |
| 44 | 28(H) | 0.43 | -25.12 | 0.49 |
| 45 | 33(H) | 0.43 | -25.08 | 0.48 |
| 46 | 29(H) | 0.43 | -25.26 | 0.48 |
| 47 | 12(O) | 295.55 | -2465300.39 | 18.30 |
| 48 | 22(O) | 295.23 | -2462590.61 | 18.37 |
| 49 | 41(H) | 0.43 | -25.55 | 0.47 |
| 50 | 23(C) | 119.50 | -544146.28 | 9.91 |
| 51 | 40(H) | 0.43 | -25.54 | 0.47 |
| 52 | 39(H) | 0.44 | -25.60 | 0.47 |

**Table S39.** QTAIM topological parameters of **2h (**Gas phase**)** at nuclear critical points (NCPs), including Electron Density ρ (a.u.), Laplacian of electron density ∇²ρ (a.u), and Average local ionization energy (ALIE) (a.u.) values for the corresponding nuclei, describing the electron distribution and local ionization characteristics of the molecule.

| CP | Corresponding nucleus | Electron Density ρ (a.u.) | Laplacian ∇²ρ (a.u.) | ALIE (a.u.) |
| --- | --- | --- | --- | --- |
| 1 | 34(H) | 0.44 | -25.75 | 0.49 |
| 2 | 36(H) | 0.44 | -25.68 | 0.48 |
| 3 | 22(C) | 119.49 | -544112.98 | 9.93 |
| 4 | 35(H) | 0.44 | -25.68 | 0.48 |
| 5 | 12(O) | 295.60 | -2465745.06 | 18.29 |
| 6 | 21(O) | 295.24 | -2462631.70 | 18.37 |
| 7 | 26(H) | 0.43 | -25.47 | 0.49 |
| 8 | 30(H) | 0.43 | -25.06 | 0.48 |
| 9 | 27(H) | 0.43 | -25.49 | 0.49 |
| 10 | 8(C) | 119.62 | -544673.27 | 9.87 |
| 11 | 25(H) | 0.43 | -25.34 | 0.49 |
| 12 | 9(C) | 119.62 | -544668.92 | 9.87 |
| 13 | 2(C) | 119.70 | -545078.50 | 9.96 |
| 14 | 7(C) | 119.63 | -544702.98 | 9.87 |
| 15 | 20(C) | 119.60 | -544572.34 | 9.93 |
| 16 | 33(H) | 0.43 | -25.50 | 0.49 |
| 17 | 14(C) | 119.63 | -544745.00 | 9.87 |
| 18 | 10(C) | 119.62 | -544661.23 | 9.88 |
| 19 | 6(C) | 119.56 | -544417.60 | 9.92 |
| 20 | 28(H) | 0.43 | -25.48 | 0.49 |
| 21 | 11(C) | 119.62 | -544680.72 | 9.88 |
| 22 | 3(C) | 119.62 | -544652.59 | 9.90 |
| 23 | 19(C) | 119.57 | -544459.77 | 9.87 |
| 24 | 1(N) | 193.94 | -1211127.99 | 13.88 |
| 25 | 15(C) | 119.57 | -544442.33 | 9.88 |
| 26 | 29(H) | 0.43 | -25.41 | 0.49 |
| 27 | 4(N) | 194.42 | -1214221.00 | 13.77 |
| 28 | 5(C) | 119.65 | -544827.48 | 9.97 |
| 29 | 18(C) | 119.59 | -544518.68 | 9.88 |
| 30 | 16(C) | 119.62 | -544679.59 | 9.86 |
| 31 | 32(H) | 0.43 | -25.48 | 0.49 |
| 32 | 31(H) | 0.43 | -24.90 | 0.47 |
| 33 | 17(C) | 119.59 | -544543.53 | 9.92 |
| 34 | 13(S) | 2588.26 | -143782267.80 | 82.65 |
| 35 | 23(O) | 295.27 | -2462877.34 | 18.36 |
| 36 | 41(H) | 0.43 | -25.30 | 0.48 |
| 37 | 38(H) | 0.44 | -25.68 | 0.48 |
| 38 | 40(C) | 119.52 | -544227.73 | 9.91 |
| 39 | 42(H) | 0.43 | -25.30 | 0.48 |
| 40 | 39(H) | 0.44 | -25.68 | 0.48 |
| 41 | 24(C) | 119.49 | -544106.07 | 9.93 |
| 42 | 43(H) | 0.43 | -25.26 | 0.50 |
| 43 | 37(H) | 0.438617805 | -25.77224108 | 0.483769033 |

**Table S40.** QTAIM topological parameters of **2h (**DMSO phase**)** at nuclear critical points (NCPs), including Electron Density ρ (a.u.), Laplacian of electron density ∇²ρ (a.u), and Average local ionization energy (ALIE) (a.u.) values for the corresponding nuclei, describing the electron distribution and local ionization characteristics of the molecule.

| CP | Corresponding nucleus | Electron Density ρ (a.u.) | Laplacian ∇²ρ (a.u.) | ALIE (a.u.) |
| --- | --- | --- | --- | --- |
| 1 | 34(H) | 0.44 | -25.61 | 0.47 |
| 2 | 35(H) | 0.43 | -25.55 | 0.47 |
| 3 | 22(C) | 119.50 | -544145.43 | 9.91 |
| 4 | 36(H) | 0.43 | -25.55 | 0.47 |
| 5 | 21(O) | 295.23 | -2462586.32 | 18.37 |
| 6 | 33(H) | 0.43 | -25.23 | 0.48 |
| 7 | 30(H) | 0.43 | -25.09 | 0.48 |
| 8 | 12(O) | 295.55 | -2465284.39 | 18.30 |
| 9 | 20(C) | 119.60 | -544577.77 | 9.93 |
| 10 | 19(C) | 119.58 | -544504.59 | 9.87 |
| 11 | 14(C) | 119.63 | -544733.83 | 9.88 |
| 12 | 2(C) | 119.70 | -545057.28 | 9.97 |
| 13 | 26(H) | 0.43 | -25.27 | 0.48 |
| 14 | 25(H) | 0.43 | -25.13 | 0.49 |
| 15 | 15(C) | 119.57 | -544420.59 | 9.89 |
| 16 | 7(C) | 119.64 | -544744.50 | 9.87 |
| 17 | 8(C) | 119.63 | -544694.48 | 9.87 |
| 18 | 3(C) | 119.62 | -544649.27 | 9.90 |
| 19 | 18(C) | 119.60 | -544572.08 | 9.87 |
| 20 | 32(H) | 0.43 | -25.21 | 0.49 |
| 21 | 1(N) | 193.94 | -1211124.96 | 13.88 |
| 22 | 6(C) | 119.56 | -544413.00 | 9.92 |
| 23 | 9(C) | 119.63 | -544717.29 | 9.87 |
| 24 | 27(H) | 0.43 | -25.28 | 0.48 |
| 25 | 16(C) | 119.61 | -544644.12 | 9.86 |
| 26 | 11(C) | 119.64 | -544742.96 | 9.88 |
| 27 | 10(C) | 119.62 | -544691.57 | 9.87 |
| 28 | 17(C) | 119.59 | -544548.27 | 9.93 |
| 29 | 4(N) | 194.40 | -1214101.80 | 13.78 |
| 30 | 29(H) | 0.43 | -25.13 | 0.49 |
| 31 | 5(C) | 119.66 | -544878.72 | 9.97 |
| 32 | 28(H) | 0.43 | -25.27 | 0.48 |
| 33 | 31(H) | 0.43 | -24.96 | 0.48 |
| 34 | 39(H) | 0.43 | -25.56 | 0.46 |
| 35 | 23(O) | 295.26 | -2462791.74 | 18.36 |
| 36 | 38(H) | 0.43 | -25.56 | 0.46 |
| 37 | 24(C) | 119.50 | -544133.99 | 9.91 |
| 38 | 13(S) | 2588.24 | -143781069.90 | 82.65 |
| 39 | 42(H) | 0.43 | -25.18 | 0.48 |
| 40 | 37(H) | 0.44 | -25.63 | 0.47 |
| 41 | 40(C) | 119.52 | -544232.52 | 9.90 |
| 42 | 41(H) | 0.43 | -25.18 | 0.48 |
| 43 | 43(H) | 0.43 | -24.99 | 0.48 |

**Table S41.** QTAIM topological parameters of **2i (**Gas phase**)** at nuclear critical points (NCPs), including Electron Density ρ (a.u.), Laplacian of electron density ∇²ρ (a.u), and Average local ionization energy (ALIE) (a.u.) values for the corresponding nuclei, describing the electron distribution and local ionization characteristics of the molecule.

| CP | Corresponding nucleus | Electron Density ρ (a.u.) | Laplacian ∇²ρ (a.u.) | ALIE (a.u.) |
| --- | --- | --- | --- | --- |
| 1 | 34(H) | 0.44 | -25.73 | 0.49 |
| 2 | 36(H) | 0.44 | -25.67 | 0.49 |
| 3 | 22(C) | 119.50 | -544117.60 | 9.94 |
| 4 | 35(H) | 0.44 | -25.68 | 0.49 |
| 5 | 12(O) | 295.61 | -2465818.19 | 18.30 |
| 6 | 21(O) | 295.24 | -2462619.00 | 18.38 |
| 7 | 30(H) | 0.43 | -25.02 | 0.49 |
| 8 | 26(H) | 0.43 | -25.45 | 0.49 |
| 9 | 27(H) | 0.43 | -25.47 | 0.49 |
| 10 | 8(C) | 119.62 | -544675.53 | 9.88 |
| 11 | 2(C) | 119.70 | -545069.77 | 9.97 |
| 12 | 9(C) | 119.62 | -544683.05 | 9.88 |
| 13 | 25(H) | 0.43 | -25.33 | 0.50 |
| 14 | 20(C) | 119.60 | -544574.41 | 9.94 |
| 15 | 33(H) | 0.43 | -25.48 | 0.50 |
| 16 | 7(C) | 119.63 | -544712.05 | 9.88 |
| 17 | 14(C) | 119.64 | -544783.83 | 9.89 |
| 18 | 10(C) | 119.62 | -544664.99 | 9.88 |
| 19 | 28(H) | 0.43 | -25.45 | 0.49 |
| 20 | 6(C) | 119.56 | -544408.04 | 9.93 |
| 21 | 11(C) | 119.62 | -544695.34 | 9.89 |
| 22 | 19(C) | 119.58 | -544465.11 | 9.88 |
| 23 | 3(C) | 119.61 | -544623.12 | 9.91 |
| 24 | 15(C) | 119.57 | -544432.21 | 9.89 |
| 25 | 1(N) | 193.95 | -1211201.08 | 13.89 |
| 26 | 29(H) | 0.43 | -25.39 | 0.50 |
| 27 | 18(C) | 119.59 | -544536.31 | 9.88 |
| 28 | 4(N) | 194.42 | -1214187.33 | 13.79 |
| 29 | 5(C) | 119.65 | -544807.21 | 9.99 |
| 30 | 16(C) | 119.62 | -544678.59 | 9.87 |
| 31 | 32(H) | 0.43 | -25.45 | 0.50 |
| 32 | 31(H) | 0.43 | -25.16 | 0.49 |
| 33 | 17(C) | 119.59 | -544542.99 | 9.93 |
| 34 | 13(S) | 2588.54 | -143798463.40 | 82.66 |
| 35 | 23(O) | 295.27 | -2462878.82 | 18.37 |
| 36 | 43(H) | 0.43 | -25.07 | 0.51 |
| 37 | 38(H) | 0.44 | -25.69 | 0.48 |
| 38 | 44(H) | 0.43 | -25.05 | 0.51 |
| 39 | 24(C) | 119.49 | -544110.15 | 9.93 |
| 40 | 39(H) | 0.44 | -25.68 | 0.48 |
| 41 | 42(C) | 119.57 | -544469.91 | 9.95 |
| 42 | 40(C) | 119.81 | -545540.12 | 10.00 |
| 43 | 37(H) | 0.44 | -25.73 | 0.49 |
| 44 | 41(O) | 295.76 | -2467007.62 | 18.33 |
| 45 | 45(Cl) | 3122.47 | -195968732.20 | 94.17 |

**Table S42.** QTAIM topological parameters of **2i (**DMSO phase**)** at nuclear critical points (NCPs), including Electron Density ρ (a.u.), Laplacian of electron density ∇²ρ (a.u), and Average local ionization energy (ALIE) (a.u.) values for the corresponding nuclei, describing the electron distribution and local ionization characteristics of the molecule.

| CP | Corresponding nucleus | Electron Density ρ (a.u.) | Laplacian ∇²ρ (a.u.) | ALIE (a.u.) |
| --- | --- | --- | --- | --- |
| 1 | 34(H) | 0.44 | -25.59 | 0.47 |
| 2 | 35(H) | 0.43 | -25.54 | 0.47 |
| 3 | 22(C) | 119.50 | -544149.54 | 9.91 |
| 4 | 36(H) | 0.43 | -25.54 | 0.47 |
| 5 | 21(O) | 295.23 | -2462579.17 | 18.37 |
| 6 | 33(H) | 0.43 | -25.21 | 0.49 |
| 7 | 30(H) | 0.43 | -25.05 | 0.49 |
| 8 | 12(O) | 295.55 | -2465328.84 | 18.30 |
| 9 | 20(C) | 119.60 | -544588.27 | 9.94 |
| 10 | 19(C) | 119.58 | -544508.10 | 9.87 |
| 11 | 14(C) | 119.65 | -544792.91 | 9.89 |
| 12 | 2(C) | 119.70 | -545050.17 | 9.98 |
| 13 | 15(C) | 119.57 | -544409.12 | 9.89 |
| 14 | 25(H) | 0.43 | -25.11 | 0.49 |
| 15 | 26(H) | 0.43 | -25.26 | 0.48 |
| 16 | 3(C) | 119.61 | -544617.34 | 9.91 |
| 17 | 18(C) | 119.60 | -544593.33 | 9.87 |
| 18 | 7(C) | 119.64 | -544752.75 | 9.88 |
| 19 | 8(C) | 119.63 | -544696.09 | 9.87 |
| 20 | 32(H) | 0.43 | -25.19 | 0.49 |
| 21 | 1(N) | 193.95 | -1211183.00 | 13.88 |
| 22 | 6(C) | 119.56 | -544408.58 | 9.93 |
| 23 | 9(C) | 119.63 | -544726.11 | 9.87 |
| 24 | 27(H) | 0.43 | -25.28 | 0.48 |
| 25 | 16(C) | 119.62 | -544650.97 | 9.87 |
| 26 | 11(C) | 119.64 | -544751.42 | 9.88 |
| 27 | 10(C) | 119.62 | -544694.17 | 9.87 |
| 28 | 17(C) | 119.59 | -544546.52 | 9.93 |
| 29 | 29(H) | 0.43 | -25.10 | 0.49 |
| 30 | 28(H) | 0.43 | -25.26 | 0.48 |
| 31 | 4(N) | 194.42 | -1214186.41 | 13.79 |
| 32 | 31(H) | 0.43 | -25.02 | 0.48 |
| 33 | 5(C) | 119.65 | -544827.00 | 9.99 |
| 34 | 39(H) | 0.43 | -25.56 | 0.46 |
| 35 | 23(O) | 295.26 | -2462802.71 | 18.36 |
| 36 | 38(H) | 0.43 | -25.56 | 0.46 |
| 37 | 24(C) | 119.50 | -544134.58 | 9.91 |
| 38 | 13(S) | 2588.50 | -143796403.80 | 82.67 |
| 39 | 44(H) | 0.42 | -24.80 | 0.50 |
| 40 | 43(H) | 0.42 | -24.78 | 0.50 |
| 41 | 37(H) | 0.44 | -25.62 | 0.47 |
| 42 | 42(C) | 119.59 | -544517.76 | 9.94 |
| 43 | 40(C) | 119.81 | -545543.85 | 10.00 |
| 44 | 45(Cl) | 3122.43 | -195966019.50 | 94.18 |
| 45 | 41(O) | 295.71 | -2466586.37 | 18.34 |

**Table S43.** QTAIM topological parameters of **3 (**Gas phase**)** at nuclear critical points (NCPs), including Electron Density ρ (a.u.), Laplacian of electron density ∇²ρ (a.u), and Average local ionization energy (ALIE) (a.u.) values for the corresponding nuclei, describing the electron distribution and local ionization characteristics of the molecule.

| CP | Corresponding nucleus | Electron Density ρ (a.u.) | Laplacian ∇²ρ (a.u.) | ALIE (a.u.) |
| --- | --- | --- | --- | --- |
| 1 | 32(H) | 0.43 | -25.36 | 0.50 |
| 2 | 8(S) | 2588.26 | -143782155.20 | 82.59 |
| 3 | 33(H) | 0.43 | -25.46 | 0.49 |
| 4 | 30(H) | 0.42 | -24.27 | 0.64 |
| 5 | 4(N) | 193.94 | -1211101.57 | 13.88 |
| 6 | 3(C) | 119.68 | -544945.16 | 10.01 |
| 7 | 9(C) | 119.62 | -544695.31 | 9.88 |
| 8 | 31(H) | 0.43 | -25.35 | 0.52 |
| 9 | 10(C) | 119.62 | -544654.98 | 9.88 |
| 10 | 2(N) | 193.97 | -1211323.88 | 13.90 |
| 11 | 5(C) | 119.50 | -544163.19 | 9.97 |
| 12 | 6(C) | 119.56 | -544419.51 | 9.93 |
| 13 | 23(N) | 195.12 | -1218707.87 | 13.76 |
| 14 | 1(C) | 119.70 | -545058.76 | 10.00 |
| 15 | 7(O) | 295.62 | -2465896.90 | 18.32 |
| 16 | 42(H) | 0.44 | -25.73 | 0.51 |
| 17 | 11(C) | 119.62 | -544682.00 | 9.88 |
| 18 | 22(C) | 119.92 | -546077.93 | 9.91 |
| 19 | 44(H) | 0.43 | -25.56 | 0.50 |
| 20 | 34(H) | 0.43 | -25.48 | 0.49 |
| 21 | 13(C) | 119.63 | -544731.05 | 9.88 |
| 22 | 38(H) | 0.42 | -24.71 | 0.55 |
| 23 | 12(C) | 119.62 | -544654.36 | 9.88 |
| 24 | 27(C) | 119.50 | -544147.30 | 9.95 |
| 25 | 26(O) | 295.19 | -2462275.35 | 18.40 |
| 26 | 14(C) | 119.50 | -544152.00 | 9.95 |
| 27 | 36(H) | 0.43 | -25.33 | 0.50 |
| 28 | 16(C) | 119.43 | -543842.63 | 10.00 |
| 29 | 37(H) | 0.43 | -25.57 | 0.52 |
| 30 | 35(H) | 0.43 | -25.45 | 0.49 |
| 31 | 17(C) | 119.58 | -544498.69 | 9.96 |
| 32 | 43(H) | 0.44 | -25.67 | 0.50 |
| 33 | 15(C) | 119.56 | -544400.52 | 9.91 |
| 34 | 24(C) | 119.91 | -546036.39 | 9.90 |
| 35 | 39(H) | 0.43 | -25.44 | 0.51 |
| 36 | 18(C) | 119.59 | -544528.09 | 9.89 |
| 37 | 21(C) | 119.59 | -544551.61 | 9.89 |
| 38 | 41(H) | 0.43 | -25.39 | 0.51 |
| 39 | 47(H) | 0.43 | -25.53 | 0.48 |
| 40 | 19(C) | 119.61 | -544617.18 | 9.89 |
| 41 | 20(C) | 119.59 | -544555.06 | 9.95 |
| 42 | 25(N) | 195.12 | -1218710.66 | 13.76 |
| 43 | 40(H) | 0.43 | -25.25 | 0.50 |
| 44 | 29(C) | 119.50 | -544135.60 | 9.93 |
| 45 | 28(O) | 295.26 | -2462848.25 | 18.38 |
| 46 | 46(H) | 0.44 | -25.67 | 0.49 |
| 47 | 45(H) | 0.44 | -25.74 | 0.49 |

**Table S44.** QTAIM topological parameters of **3 (**DMSO phase**)** at nuclear critical points (NCPs), including Electron Density ρ (a.u.), Laplacian of electron density ∇²ρ (a.u), and Average local ionization energy (ALIE) (a.u.) values for the corresponding nuclei, describing the electron distribution and local ionization characteristics of the molecule.

| CP | Corresponding nucleus | Electron Density ρ (a.u.) | Laplacian ∇²ρ (a.u.) | ALIE (a.u.) |
| --- | --- | --- | --- | --- |
| 1 | 32(H) | 0.43 | -25.13 | 0.49 |
| 2 | 8(S) | 2588.16 | -143776546.00 | 82.59 |
| 3 | 30(H) | 0.41 | -23.68 | 0.63 |
| 4 | 33(H) | 0.43 | -25.27 | 0.48 |
| 5 | 4(N) | 193.89 | -1210837.50 | 13.87 |
| 6 | 3(C) | 119.67 | -544939.60 | 10.00 |
| 7 | 9(C) | 119.63 | -544728.96 | 9.87 |
| 8 | 31(H) | 0.43 | -25.04 | 0.50 |
| 9 | 10(C) | 119.62 | -544685.03 | 9.87 |
| 10 | 2(N) | 193.97 | -1211290.35 | 13.89 |
| 11 | 5(C) | 119.51 | -544161.51 | 9.95 |
| 12 | 6(C) | 119.56 | -544423.42 | 9.92 |
| 13 | 23(N) | 195.03 | -1218089.07 | 13.76 |
| 14 | 1(C) | 119.70 | -545070.28 | 9.99 |
| 15 | 42(H) | 0.44 | -25.58 | 0.47 |
| 16 | 38(H) | 0.41 | -24.34 | 0.52 |
| 17 | 22(C) | 119.94 | -546188.79 | 9.90 |
| 18 | 44(H) | 0.43 | -25.55 | 0.47 |
| 19 | 11(C) | 119.63 | -544713.32 | 9.87 |
| 20 | 7(O) | 295.58 | -2465560.85 | 18.31 |
| 21 | 34(H) | 0.43 | -25.29 | 0.48 |
| 22 | 27(C) | 119.50 | -544150.30 | 9.92 |
| 23 | 16(C) | 119.44 | -543888.03 | 9.98 |
| 24 | 26(O) | 295.20 | -2462341.35 | 18.37 |
| 25 | 13(C) | 119.64 | -544750.58 | 9.87 |
| 26 | 14(C) | 119.50 | -544140.18 | 9.94 |
| 27 | 12(C) | 119.62 | -544683.17 | 9.87 |
| 28 | 36(H) | 0.43 | -25.15 | 0.49 |
| 29 | 37(H) | 0.43 | -25.39 | 0.49 |
| 30 | 43(H) | 0.43 | -25.53 | 0.47 |
| 31 | 17(C) | 119.59 | -544525.85 | 9.94 |
| 32 | 15(C) | 119.56 | -544414.07 | 9.89 |
| 33 | 35(H) | 0.43 | -25.27 | 0.48 |
| 34 | 24(C) | 119.94 | -546172.68 | 9.90 |
| 35 | 39(H) | 0.43 | -25.19 | 0.49 |
| 36 | 18(C) | 119.59 | -544539.27 | 9.87 |
| 37 | 21(C) | 119.59 | -544562.14 | 9.87 |
| 38 | 41(H) | 0.43 | -25.22 | 0.49 |
| 39 | 25(N) | 195.02 | -1218070.13 | 13.76 |
| 40 | 19(C) | 119.61 | -544620.87 | 9.87 |
| 41 | 47(H) | 0.43 | -25.52 | 0.46 |
| 42 | 20(C) | 119.59 | -544545.06 | 9.93 |
| 43 | 40(H) | 0.43 | -25.13 | 0.48 |
| 44 | 28(O) | 295.25 | -2462778.84 | 18.36 |
| 45 | 29(C) | 119.50 | -544140.61 | 9.91 |
| 46 | 46(H) | 0.43 | -25.54 | 0.46 |
| 47 | 45(H) | 0.44 | -25.62 | 0.47 |

**Table S45:** Topological parameters and energy density descriptors for compound **1** (Gas phase) at bond critical points (BCP), including atomic connectivity, electron density ρ(r) (a.u.), Lagrangian kinetic energy density G(r) (a.u.), potential energy density V(r) (a.u.), total energy density E(r) or H(r) (a.u.), Laplacian of electron density ∇²ρ(r) (a.u.), ellipticity ε (dimensionless), and bond type.

| CP | Connected Atoms | ρ(r) | G(r) | V(r) | H(r) | ∇²ρ(r) | ε | Bond Type |
| --- | --- | --- | --- | --- | --- | --- | --- | --- |
| 41 | 38(H ) -- 24(C) | 0.28 | 0.04 | -0.32 | -0.28 | -0.99 | 0.04 | Covalent bond |
| 42 | 23(O ) -- 24(C ) | 0.25 | 0.27 | -0.61 | -0.34 | -0.29 | 0.01 | Covalent bond |
| 43 | 32(H ) -- 16(C ) | 0.28 | 0.04 | -0.32 | -0.28 | -0.96 | 0.02 | Covalent bond |
| 44 | 24(C ) -- 39(H ) | 0.28 | 0.04 | -0.31 | -0.27 | -0.94 | 0.04 | Covalent bond |
| 45 | 23(O ) -- 17(C ) | 0.29 | 0.34 | -0.75 | -0.41 | -0.30 | 0.01 | Covalent bond |
| 46 | 31(H ) -- 14(C ) | 0.28 | 0.04 | -0.32 | -0.28 | -0.96 | 0.02 | Covalent bond |
| 47 | 24(C ) -- 40(H ) | 0.28 | 0.04 | -0.31 | -0.27 | -0.94 | 0.04 | Covalent bond |
| 48 | 16(C ) -- 17(C ) | 0.31 | 0.10 | -0.43 | -0.32 | -0.89 | 0.24 | Covalent bond |
| 49 | 12(O ) -- 2(C ) | 0.42 | 0.67 | -1.38 | -0.70 | -0.12 | 0.07 | Covalent bond |
| 50 | 27(H ) -- 8(C ) | 0.28 | 0.04 | -0.32 | -0.28 | -0.97 | 0.02 | Covalent bond |
| 51 | 16(C ) -- 15(C ) | 0.30 | 0.10 | -0.41 | -0.31 | -0.83 | 0.23 | Covalent bond |
| 52 | 26(H ) -- 7(C ) | 0.28 | 0.04 | -0.32 | -0.28 | -0.98 | 0.02 | Covalent bond |
| 53 | 8(C ) -- 7(C ) | 0.31 | 0.10 | -0.42 | -0.32 | -0.87 | 0.20 | Covalent bond |
| 54 | 14(C ) -- 15(C ) | 0.27 | 0.07 | -0.32 | -0.25 | -0.70 | 0.11 | Covalent bond |
| 55 | 17(C ) -- 18(C ) | 0.31 | 0.10 | -0.42 | -0.32 | -0.86 | 0.25 | Covalent bond |
| 56 | 14(C ) -- 3(C ) | 0.33 | 0.13 | -0.50 | -0.37 | -0.95 | 0.32 | Covalent bond |
| 57 | 8(C ) -- 9(C ) | 0.31 | 0.10 | -0.42 | -0.32 | -0.87 | 0.20 | Covalent bond |
| 58 | 2(C ) -- 3(C ) | 0.27 | 0.07 | -0.31 | -0.24 | -0.69 | 0.14 | Covalent bond |
| 59 | 2(C ) -- 1(N ) | 0.29 | 0.17 | -0.55 | -0.38 | -0.81 | 0.09 | Covalent bond |
| 60 | 28(H ) -- 9(C ) | 0.28 | 0.04 | -0.32 | -0.28 | -0.97 | 0.02 | Covalent bond |
| 62 | 7(C ) -- 6(C ) | 0.31 | 0.10 | -0.43 | -0.32 | -0.88 | 0.24 | Covalent bond |
| 64 | 15(C ) -- 20(C ) | 0.30 | 0.09 | -0.39 | -0.30 | -0.82 | 0.23 | Covalent bond |
| 65 | 18(C ) -- 33(H ) | 0.28 | 0.04 | -0.32 | -0.28 | -0.96 | 0.02 | Covalent bond |
| 66 | 9(C ) -- 10(C ) | 0.31 | 0.10 | -0.42 | -0.32 | -0.87 | 0.20 | Covalent bond |
| 67 | 6(C ) -- 1(N ) | 0.27 | 0.19 | -0.55 | -0.36 | -0.66 | 0.03 | Covalent bond |
| 68 | 3(C ) -- 4(N ) | 0.30 | 0.23 | -0.65 | -0.42 | -0.79 | 0.08 | Covalent bond |
| 70 | 6(C ) -- 11(C ) | 0.31 | 0.10 | -0.43 | -0.32 | -0.88 | 0.24 | Covalent bond |
| 71 | 18(C ) -- 19(C ) | 0.30 | 0.10 | -0.41 | -0.31 | -0.83 | 0.21 | Covalent bond |
| 73 | 10(C ) -- 11(C ) | 0.31 | 0.10 | -0.42 | -0.32 | -0.87 | 0.20 | Covalent bond |
| 74 | 20(C ) -- 19(C ) | 0.31 | 0.11 | -0.43 | -0.32 | -0.87 | 0.27 | Covalent bond |
| 75 | 10(C ) -- 29(H ) | 0.28 | 0.04 | -0.32 | -0.28 | -0.97 | 0.02 | Covalent bond |
| 76 | 20(C ) -- 21(O ) | 0.28 | 0.33 | -0.72 | -0.39 | -0.26 | 0.03 | Covalent bond |
| 77 | 1(N ) -- 5(C ) | 0.30 | 0.18 | -0.57 | -0.40 | -0.87 | 0.10 | Covalent bond |
| 78 | 11(C ) -- 30(H ) | 0.28 | 0.04 | -0.32 | -0.28 | -0.98 | 0.01 | Covalent bond |
| 79 | 4(N ) -- 5(C ) | 0.32 | 0.22 | -0.67 | -0.45 | -0.92 | 0.13 | Covalent bond |
| 80 | 19(C ) -- 34(H ) | 0.28 | 0.04 | -0.32 | -0.28 | -0.96 | 0.02 | Covalent bond |
| **81** | **21(O ) -- 25(H )** | **0.03** | **0.03** | **-0.02** | **0.00** | **0.12** | **0.14** | **H-Bond** |
| 82 | 21(O ) -- 22(C ) | 0.25 | 0.27 | -0.60 | -0.33 | -0.24 | 0.00 | Covalent bond |
| 83 | 5(C ) -- 13(S ) | 0.22 | 0.27 | -0.53 | -0.26 | 0.05 | 0.04 | Covalent bond |
| 84 | 37(H ) -- 22(C ) | 0.28 | 0.04 | -0.31 | -0.28 | -0.95 | 0.04 | Covalent bond |
| 85 | 22(C ) -- 35(H ) | 0.28 | 0.04 | -0.32 | -0.28 | -0.99 | 0.04 | Covalent bond |
| 86 | 22(C ) -- 36(H ) | 0.28 | 0.04 | -0.31 | -0.27 | -0.95 | 0.04 | Covalent bond |

**Table S46:** Topological parameters and energy density descriptors for compound **1** (DMSO phase) at bond critical points (BCP), including atomic connectivity, electron density ρ(r) (a.u.), Lagrangian kinetic energy density G(r) (a.u.), potential energy density V(r) (a.u.), total energy density E(r) or H(r) (a.u.), Laplacian of electron density ∇²ρ(r) (a.u.), ellipticity ε (dimensionless), and bond type.

| CP | Connected atoms | ρ(r) | G(r) | V(r) | H(r) | ∇²ρ(r) | ε | Bond Type |
| --- | --- | --- | --- | --- | --- | --- | --- | --- |
| 41 | 29(H ) -- 10(C ) | 0.28 | 0.04 | -0.32 | -0.28 | -0.98 | 0.02 | Covalent bond |
| 42 | 28(H ) -- 9(C ) | 0.28 | 0.04 | -0.32 | -0.28 | -0.98 | 0.01 | Covalent bond |
| 43 | 10(C ) -- 9(C ) | 0.31 | 0.10 | -0.42 | -0.32 | -0.86 | 0.19 | Covalent bond |
| 44 | 10(C ) -- 11(C ) | 0.31 | 0.10 | -0.42 | -0.32 | -0.86 | 0.20 | Covalent bond |
| 45 | 30(H ) -- 11(C ) | 0.28 | 0.03 | -0.31 | -0.28 | -0.98 | 0.02 | Covalent bond |
| 46 | 38(H ) -- 24(C ) | 0.28 | 0.04 | -0.32 | -0.28 | -0.99 | 0.04 | Covalent bond |
| 47 | 32(H ) -- 16(C ) | 0.28 | 0.04 | -0.31 | -0.28 | -0.97 | 0.02 | Covalent bond |
| 48 | 12(O ) -- 2(C ) | 0.41 | 0.65 | -1.35 | -0.69 | -0.15 | 0.07 | Covalent bond |
| 49 | 11(C ) -- 6(C ) | 0.31 | 0.10 | -0.43 | -0.32 | -0.88 | 0.24 | Covalent bond |
| 50 | 9(C ) -- 8(C ) | 0.31 | 0.10 | -0.42 | -0.32 | -0.86 | 0.19 | Covalent bond |
| 52 | 31(H ) -- 14(C ) | 0.28 | 0.04 | -0.32 | -0.28 | -0.97 | 0.02 | Covalent bond |
| 53 | 23(O ) -- 24(C ) | 0.25 | 0.27 | -0.59 | -0.33 | -0.25 | 0.00 | Covalent bond |
| 54 | 2(C ) -- 1(N ) | 0.30 | 0.18 | -0.57 | -0.39 | -0.82 | 0.09 | Covalent bond |
| 55 | 24(C ) -- 40(H ) | 0.28 | 0.04 | -0.31 | -0.27 | -0.95 | 0.04 | Covalent bond |
| 56 | 23(O ) -- 17(C ) | 0.29 | 0.33 | -0.75 | -0.41 | -0.32 | 0.01 | Covalent bond |
| 57 | 6(C ) -- 1(N ) | 0.27 | 0.19 | -0.54 | -0.36 | -0.67 | 0.03 | Covalent bond |
| 58 | 8(C ) -- 27(H ) | 0.28 | 0.04 | -0.32 | -0.28 | -0.98 | 0.02 | Covalent bond |
| 59 | 8(C ) -- 7(C ) | 0.31 | 0.10 | -0.42 | -0.32 | -0.86 | 0.20 | Covalent bond |
| 60 | 16(C ) -- 17(C ) | 0.31 | 0.10 | -0.43 | -0.32 | -0.89 | 0.24 | Covalent bond |
| 61 | 2(C ) -- 3(C ) | 0.27 | 0.07 | -0.31 | -0.25 | -0.70 | 0.15 | Covalent bond |
| 62 | 6(C ) -- 7(C ) | 0.31 | 0.10 | -0.43 | -0.32 | -0.88 | 0.24 | Covalent bond |
| 63 | 24(C ) -- 39(H ) | 0.28 | 0.04 | -0.31 | -0.27 | -0.95 | 0.04 | Covalent bond |
| 64 | 16(C ) -- 15(C ) | 0.30 | 0.10 | -0.41 | -0.31 | -0.83 | 0.22 | Covalent bond |
| 65 | 14(C ) -- 3(C ) | 0.33 | 0.13 | -0.50 | -0.37 | -0.95 | 0.31 | Covalent bond |
| 66 | 14(C ) -- 15(C ) | 0.28 | 0.07 | -0.33 | -0.25 | -0.71 | 0.11 | Covalent bond |
| 68 | 7(C ) -- 26(H ) | 0.28 | 0.03 | -0.31 | -0.28 | -0.98 | 0.02 | Covalent bond |
| 69 | 3(C ) -- 4(N ) | 0.30 | 0.22 | -0.63 | -0.41 | -0.77 | 0.10 | Covalent bond |
| 70 | 17(C ) -- 18(C ) | 0.31 | 0.10 | -0.42 | -0.32 | -0.86 | 0.24 | Covalent bond |
| 71 | 1(N ) -- 5(C ) | 0.31 | 0.19 | -0.59 | -0.41 | -0.89 | 0.11 | Covalent bond |
| 73 | 15(C ) -- 20(C ) | 0.30 | 0.09 | -0.39 | -0.30 | -0.82 | 0.23 | Covalent bond |
| 74 | 5(C ) -- 4(N ) | 0.33 | 0.23 | -0.70 | -0.47 | -0.94 | 0.15 | Covalent bond |
| 76 | 18(C ) -- 33(H ) | 0.28 | 0.04 | -0.32 | -0.28 | -0.98 | 0.02 | Covalent bond |
| 77 | 18(C ) -- 19(C ) | 0.30 | 0.10 | -0.41 | -0.31 | -0.83 | 0.21 | Covalent bond |
| 78 | 5(C ) -- 13(S ) | 0.21 | 0.25 | -0.50 | -0.25 | -0.02 | 0.06 | Covalent bond |
| 79 | 4(N ) -- 25(H ) | 0.33 | 0.04 | -0.55 | -0.51 | -1.86 | 0.04 | Covalent bond |
| 80 | 20(C ) -- 19(C ) | 0.31 | 0.11 | -0.43 | -0.32 | -0.86 | 0.26 | Covalent bond |
| 81 | 20(C ) -- 21(O ) | 0.28 | 0.34 | -0.74 | -0.41 | -0.27 | 0.03 | Covalent bond |
| **82** | **25(H ) -- 21(O )** | **0.03** | **0.02** | **-0.02** | **0.00** | **0.11** | **0.16** | **H-Bond** |
| 83 | 19(C ) -- 34(H ) | 0.28 | 0.04 | -0.32 | -0.28 | -0.98 | 0.02 | Covalent bond |
| 84 | 21(O ) -- 22(C ) | 0.24 | 0.27 | -0.58 | -0.32 | -0.20 | 0.00 | Covalent bond |
| 85 | 22(C ) -- 36(H ) | 0.28 | 0.04 | -0.31 | -0.28 | -0.96 | 0.04 | Covalent bond |
| 86 | 22(C ) -- 37(H ) | 0.28 | 0.04 | -0.31 | -0.28 | -0.96 | 0.04 | Covalent bond |
| 87 | 22(C ) -- 35(H ) | 0.28 | 0.03 | -0.32 | -0.28 | -0.99 | 0.04 | Covalent bond |

**Table S47:** Topological parameters and energy density descriptors for compound **2a** (Gas phase) at bond critical points (BCP), including atomic connectivity, electron density ρ(r) (a.u.), Lagrangian kinetic energy density G(r) (a.u.), potential energy density V(r) (a.u.), total energy density E(r) or H(r) (a.u.), Laplacian of electron density ∇²ρ(r) (a.u.), ellipticity ε (dimensionless), and bond type.

| CP | Connected atoms | ρ(r) | G(r) | V(r) | H(r) | ∇²ρ(r) | ε | Bond Type |
| --- | --- | --- | --- | --- | --- | --- | --- | --- |
| 54 | 48(H ) -- 29(C ) | 0.28 | 0.04 | -0.32 | -0.28 | -0.99 | 0.04 | Covalent bond |
| 55 | 49(H ) -- 29(C ) | 0.28 | 0.04 | -0.31 | -0.27 | -0.94 | 0.04 | Covalent bond |
| 56 | 29(C ) -- 50(H ) | 0.28 | 0.04 | -0.31 | -0.27 | -0.94 | 0.04 | Covalent bond |
| 57 | 29(C ) -- 28(O ) | 0.25 | 0.27 | -0.62 | -0.34 | -0.27 | 0.01 | Covalent bond |
| 58 | 40(H ) -- 19(C ) | 0.28 | 0.04 | -0.32 | -0.28 | -0.96 | 0.03 | Covalent bond |
| 59 | 28(O ) -- 20(C ) | 0.28 | 0.34 | -0.75 | -0.41 | -0.27 | 0.01 | Covalent bond |
| **60** | **28(O ) -- 37(H )** | **0.02** | **0.02** | **-0.01** | **0.00** | **0.08** | **0.91** | **H-Bond** |
| 61 | 19(C ) -- 20(C ) | 0.31 | 0.11 | -0.43 | -0.32 | -0.87 | 0.27 | Covalent bond |
| 63 | 19(C ) -- 18(C ) | 0.30 | 0.10 | -0.41 | -0.31 | -0.83 | 0.21 | Covalent bond |
| 64 | 20(C ) -- 15(C ) | 0.30 | 0.09 | -0.38 | -0.29 | -0.80 | 0.22 | Covalent bond |
| 65 | 37(H ) -- 14(C ) | 0.29 | 0.04 | -0.32 | -0.29 | -1.00 | 0.01 | Covalent bond |
| 66 | 44(H ) -- 24(C ) | 0.28 | 0.04 | -0.32 | -0.28 | -0.97 | 0.02 | Covalent bond |
| 67 | 39(H ) -- 18(C ) | 0.28 | 0.04 | -0.32 | -0.28 | -0.96 | 0.02 | Covalent bond |
| 68 | 43(H ) -- 23(C ) | 0.28 | 0.04 | -0.32 | -0.28 | -0.96 | 0.02 | Covalent bond |
| 69 | 24(C ) -- 23(C ) | 0.31 | 0.10 | -0.42 | -0.32 | -0.87 | 0.20 | Covalent bond |
| 70 | 41(H ) -- 21(C ) | 0.28 | 0.04 | -0.31 | -0.28 | -0.95 | 0.01 | Covalent bond |
| 71 | 24(C ) -- 25(C ) | 0.31 | 0.10 | -0.42 | -0.32 | -0.87 | 0.20 | Covalent bond |
| 73 | 45(H ) -- 25(C ) | 0.28 | 0.04 | -0.32 | -0.28 | -0.96 | 0.02 | Covalent bond |
| 74 | 23(C ) -- 22(C ) | 0.31 | 0.10 | -0.42 | -0.31 | -0.85 | 0.21 | Covalent bond |
| 76 | 15(C ) -- 14(C ) | 0.28 | 0.08 | -0.34 | -0.26 | -0.73 | 0.11 | Covalent bond |
| 77 | 42(H ) -- 21(C ) | 0.28 | 0.04 | -0.31 | -0.28 | -0.95 | 0.00 | Covalent bond |
| 78 | 25(C ) -- 26(C ) | 0.31 | 0.10 | -0.42 | -0.32 | -0.87 | 0.20 | Covalent bond |
| 79 | 22(C ) -- 21(C ) | 0.26 | 0.06 | -0.28 | -0.22 | -0.62 | 0.04 | Covalent bond |
| 80 | 22(C ) -- 27(C ) | 0.31 | 0.10 | -0.42 | -0.31 | -0.85 | 0.21 | Covalent bond |
| 81 | 26(C ) -- 27(C ) | 0.31 | 0.10 | -0.42 | -0.32 | -0.87 | 0.20 | Covalent bond |
| 82 | 26(C ) -- 46(H ) | 0.28 | 0.04 | -0.32 | -0.28 | -0.96 | 0.02 | Covalent bond |
| 83 | 18(C ) -- 17(C ) | 0.31 | 0.10 | -0.42 | -0.32 | -0.87 | 0.25 | Covalent bond |
| 84 | 27(C ) -- 47(H ) | 0.28 | 0.04 | -0.32 | -0.28 | -0.96 | 0.02 | Covalent bond |
| 85 | 14(C ) -- 3(C ) | 0.33 | 0.12 | -0.47 | -0.35 | -0.94 | 0.29 | Covalent bond |
| 86 | 15(C ) -- 16(C ) | 0.30 | 0.10 | -0.41 | -0.31 | -0.83 | 0.20 | Covalent bond |
| 87 | 4(N ) -- 3(C ) | 0.30 | 0.16 | -0.53 | -0.37 | -0.87 | 0.09 | Covalent bond |
| 88 | 21(C ) -- 13(S ) | 0.17 | 0.05 | -0.15 | -0.11 | -0.24 | 0.09 | Covalent bond |
| 89 | 17(C ) -- 16(C ) | 0.31 | 0.10 | -0.43 | -0.32 | -0.89 | 0.23 | Covalent bond |
| 90 | 17(C ) -- 30(O ) | 0.28 | 0.33 | -0.74 | -0.41 | -0.30 | 0.01 | Covalent bond |
| 91 | 4(N ) -- 5(C ) | 0.38 | 0.30 | -0.88 | -0.58 | -1.12 | 0.26 | Covalent bond |
| 93 | 53(H ) -- 31(C ) | 0.28 | 0.04 | -0.31 | -0.27 | -0.94 | 0.04 | Covalent bond |
| 94 | 16(C ) -- 38(H ) | 0.29 | 0.03 | -0.32 | -0.29 | -1.01 | 0.02 | Covalent bond |
| 95 | 3(C ) -- 2(C ) | 0.27 | 0.07 | -0.30 | -0.24 | -0.66 | 0.15 | Covalent bond |
| 97 | 52(H ) -- 31(C ) | 0.28 | 0.04 | -0.31 | -0.27 | -0.94 | 0.04 | Covalent bond |
| 98 | 5(C ) -- 13(S ) | 0.20 | 0.06 | -0.23 | -0.16 | -0.40 | 0.26 | Covalent bond |
| 99 | 31(C ) -- 30(O ) | 0.26 | 0.27 | -0.62 | -0.35 | -0.30 | 0.01 | Covalent bond |
| 100 | 5(C ) -- 1(N ) | 0.30 | 0.19 | -0.58 | -0.39 | -0.83 | 0.11 | Covalent bond |
| **101** | **38(H ) -- 12(O )** | **0.02** | **0.02** | **-0.02** | **0.00** | **0.09** | **0.02** | **H-Bond** |
| 102 | 2(C ) -- 1(N ) | 0.29 | 0.18 | -0.55 | -0.38 | -0.79 | 0.10 | Covalent bond |
| 103 | 31(C ) -- 51(H ) | 0.28 | 0.04 | -0.32 | -0.28 | -0.98 | 0.04 | Covalent bond |
| 104 | 2(C ) -- 12(O ) | 0.41 | 0.65 | -1.34 | -0.69 | -0.16 | 0.07 | Covalent bond |
| 105 | 1(N ) -- 6(C ) | 0.28 | 0.19 | -0.55 | -0.36 | -0.71 | 0.04 | Covalent bond |
| 106 | 36(H ) -- 11(C ) | 0.28 | 0.04 | -0.32 | -0.28 | -0.98 | 0.01 | Covalent bond |
| 107 | 6(C ) -- 11(C ) | 0.31 | 0.10 | -0.42 | -0.32 | -0.87 | 0.23 | Covalent bond |
| 108 | 6(C ) -- 7(C ) | 0.31 | 0.10 | -0.42 | -0.32 | -0.87 | 0.24 | Covalent bond |
| 109 | 32(H ) -- 7(C ) | 0.28 | 0.04 | -0.32 | -0.28 | -0.97 | 0.02 | Covalent bond |
| 110 | 11(C ) -- 10(C ) | 0.31 | 0.10 | -0.42 | -0.32 | -0.87 | 0.20 | Covalent bond |
| 112 | 7(C ) -- 8(C ) | 0.31 | 0.10 | -0.42 | -0.32 | -0.86 | 0.20 | Covalent bond |
| 113 | 10(C ) -- 35(H ) | 0.28 | 0.04 | -0.32 | -0.28 | -0.97 | 0.02 | Covalent bond |
| 114 | 10(C ) -- 9(C ) | 0.31 | 0.10 | -0.42 | -0.32 | -0.86 | 0.20 | Covalent bond |
| 115 | 8(C ) -- 9(C ) | 0.31 | 0.10 | -0.42 | -0.32 | -0.87 | 0.20 | Covalent bond |
| 116 | 8(C ) -- 33(H ) | 0.28 | 0.04 | -0.32 | -0.28 | -0.97 | 0.02 | Covalent bond |
| 117 | 9(C ) -- 34(H ) | 0.28 | 0.04 | -0.32 | -0.28 | -0.97 | 0.02 | Covalent bond |

**Table S48:** Topological parameters and energy density descriptors for compound **2a** (DMSO phase) at bond critical points (BCP), including atomic connectivity, electron density ρ(r) (a.u.), Lagrangian kinetic energy density G(r) (a.u.), potential energy density V(r) (a.u.), total energy density E(r) or H(r) (a.u.), Laplacian of electron density ∇²ρ(r) (a.u.), ellipticity ε (dimensionless), and bond type.

| CP | Connected atoms | ρ(r) | G(r) | V(r) | H(r) | ∇²ρ(r) | ε | Bond Type |
| --- | --- | --- | --- | --- | --- | --- | --- | --- |
| 54 | 48(H ) -- 29(C ) | 0.28 | 0.04 | -0.32 | -0.28 | -0.99 | 0.04 | Covalent bond |
| 55 | 50(H ) -- 29(C ) | 0.28 | 0.04 | -0.31 | -0.27 | -0.95 | 0.04 | Covalent bond |
| 56 | 49(H ) -- 29(C ) | 0.28 | 0.04 | -0.31 | -0.28 | -0.95 | 0.04 | Covalent bond |
| 57 | 29(C ) -- 28(O ) | 0.24 | 0.27 | -0.59 | -0.32 | -0.22 | 0.00 | Covalent bond |
| 58 | 40(H ) -- 19(C ) | 0.28 | 0.04 | -0.32 | -0.28 | -0.97 | 0.02 | Covalent bond |
| 59 | 28(O ) -- 20(C ) | 0.29 | 0.34 | -0.76 | -0.42 | -0.29 | 0.02 | Covalent bond |
| 60 | 19(C ) -- 20(C ) | 0.31 | 0.11 | -0.43 | -0.32 | -0.86 | 0.25 | Covalent bond |
| 61 | 19(C ) -- 18(C ) | 0.30 | 0.10 | -0.41 | -0.31 | -0.83 | 0.21 | Covalent bond |
| 62 | 37(H ) -- 14(C ) | 0.28 | 0.04 | -0.32 | -0.28 | -0.98 | 0.01 | Covalent bond |
| 63 | 20(C ) -- 15(C ) | 0.30 | 0.09 | -0.38 | -0.29 | -0.81 | 0.23 | Covalent bond |
| 64 | 44(H ) -- 24(C ) | 0.28 | 0.04 | -0.32 | -0.28 | -0.97 | 0.02 | Covalent bond |
| 65 | 43(H ) -- 23(C ) | 0.28 | 0.04 | -0.31 | -0.28 | -0.97 | 0.02 | Covalent bond |
| 66 | 41(H ) -- 21(C ) | 0.28 | 0.04 | -0.31 | -0.28 | -0.96 | 0.01 | Covalent bond |
| 67 | 24(C ) -- 23(C ) | 0.31 | 0.10 | -0.42 | -0.32 | -0.86 | 0.20 | Covalent bond |
| 68 | 39(H ) -- 18(C ) | 0.28 | 0.04 | -0.32 | -0.28 | -0.97 | 0.02 | Covalent bond |
| 70 | 14(C ) -- 15(C ) | 0.28 | 0.08 | -0.33 | -0.26 | -0.72 | 0.11 | Covalent bond |
| 71 | 24(C ) -- 25(C ) | 0.31 | 0.10 | -0.42 | -0.32 | -0.86 | 0.19 | Covalent bond |
| 72 | 23(C ) -- 22(C ) | 0.31 | 0.10 | -0.41 | -0.31 | -0.85 | 0.21 | Covalent bond |
| 73 | 42(H ) -- 21(C ) | 0.28 | 0.04 | -0.31 | -0.28 | -0.96 | 0.01 | Covalent bond |
| 74 | 45(H ) -- 25(C ) | 0.28 | 0.04 | -0.32 | -0.28 | -0.97 | 0.02 | Covalent bond |
| 76 | 22(C ) -- 21(C ) | 0.26 | 0.06 | -0.28 | -0.22 | -0.62 | 0.04 | Covalent bond |
| 77 | 25(C ) -- 26(C ) | 0.31 | 0.10 | -0.42 | -0.32 | -0.86 | 0.19 | Covalent bond |
| 78 | 22(C ) -- 27(C ) | 0.31 | 0.10 | -0.41 | -0.31 | -0.85 | 0.21 | Covalent bond |
| 79 | 14(C ) -- 3(C ) | 0.33 | 0.12 | -0.48 | -0.36 | -0.95 | 0.30 | Covalent bond |
| 80 | 18(C ) -- 17(C ) | 0.31 | 0.10 | -0.42 | -0.32 | -0.86 | 0.24 | Covalent bond |
| 81 | 15(C ) -- 16(C ) | 0.30 | 0.10 | -0.41 | -0.31 | -0.83 | 0.22 | Covalent bond |
| 82 | 26(C ) -- 27(C ) | 0.31 | 0.10 | -0.42 | -0.32 | -0.86 | 0.20 | Covalent bond |
| 83 | 4(N ) -- 3(C ) | 0.30 | 0.16 | -0.53 | -0.37 | -0.86 | 0.09 | Covalent bond |
| 84 | 26(C ) -- 46(H ) | 0.28 | 0.04 | -0.32 | -0.28 | -0.97 | 0.02 | Covalent bond |
| 85 | 27(C ) -- 47(H ) | 0.28 | 0.04 | -0.31 | -0.28 | -0.97 | 0.02 | Covalent bond |
| 86 | 21(C ) -- 13(S ) | 0.17 | 0.05 | -0.15 | -0.11 | -0.24 | 0.08 | Covalent bond |
| 87 | 17(C ) -- 16(C ) | 0.31 | 0.10 | -0.43 | -0.32 | -0.89 | 0.24 | Covalent bond |
| 88 | 4(N ) -- 5(C ) | 0.38 | 0.29 | -0.86 | -0.57 | -1.12 | 0.25 | Covalent bond |
| 89 | 17(C ) -- 30(O ) | 0.29 | 0.33 | -0.74 | -0.41 | -0.32 | 0.02 | Covalent bond |
| 91 | 3(C ) -- 2(C ) | 0.27 | 0.07 | -0.30 | -0.23 | -0.66 | 0.14 | Covalent bond |
| 93 | 5(C ) -- 13(S ) | 0.20 | 0.07 | -0.23 | -0.17 | -0.41 | 0.27 | Covalent bond |
| 94 | 16(C ) -- 38(H ) | 0.28 | 0.04 | -0.32 | -0.29 | -1.00 | 0.02 | Covalent bond |
| 95 | 52(H ) -- 31(C ) | 0.28 | 0.04 | -0.31 | -0.27 | -0.95 | 0.04 | Covalent bond |
| 96 | 5(C ) -- 1(N ) | 0.30 | 0.19 | -0.59 | -0.40 | -0.85 | 0.11 | Covalent bond |
| 97 | 53(H ) -- 31(C ) | 0.28 | 0.04 | -0.31 | -0.27 | -0.95 | 0.04 | Covalent bond |
| 98 | 30(O ) -- 31(C ) | 0.25 | 0.27 | -0.60 | -0.33 | -0.25 | 0.00 | Covalent bond |
| **99** | **38(H ) -- 12(O )** | **0.02** | **0.01** | **-0.01** | **0.00** | **0.06** | **0.18** | **H-Bond** |
| 100 | 2(C ) -- 1(N ) | 0.30 | 0.19 | -0.58 | -0.39 | -0.81 | 0.10 | Covalent bond |
| 101 | 2(C ) -- 12(O ) | 0.41 | 0.64 | -1.32 | -0.68 | -0.17 | 0.07 | Covalent bond |
| 102 | 31(C ) -- 51(H ) | 0.28 | 0.04 | -0.32 | -0.28 | -0.99 | 0.04 | Covalent bond |
| 104 | 1(N ) -- 6(C ) | 0.27 | 0.18 | -0.54 | -0.36 | -0.69 | 0.03 | Covalent bond |
| 104 | 36(H ) -- 11(C ) | 0.28 | 0.03 | -0.31 | -0.28 | -0.98 | 0.02 | Covalent bond |
| 105 | 6(C ) -- 11(C ) | 0.31 | 0.10 | -0.42 | -0.32 | -0.87 | 0.24 | Covalent bond |
| 106 | 6(C ) -- 7(C ) | 0.31 | 0.10 | -0.42 | -0.32 | -0.87 | 0.24 | Covalent bond |
| 107 | 11(C ) -- 10(C ) | 0.31 | 0.10 | -0.42 | -0.32 | -0.87 | 0.20 | Covalent bond |
| 108 | 32(H ) -- 7(C ) | 0.28 | 0.03 | -0.31 | -0.28 | -0.98 | 0.02 | Covalent bond |
| 110 | 10(C ) -- 35(H ) | 0.28 | 0.04 | -0.32 | -0.28 | -0.98 | 0.02 | Covalent bond |
| 111 | 7(C ) -- 8(C ) | 0.31 | 0.10 | -0.42 | -0.32 | -0.86 | 0.20 | Covalent bond |
| 112 | 10(C ) -- 9(C ) | 0.31 | 0.10 | -0.42 | -0.32 | -0.86 | 0.19 | Covalent bond |
| 113 | 8(C ) -- 9(C ) | 0.31 | 0.10 | -0.42 | -0.32 | -0.86 | 0.19 | Covalent bond |
| 114 | 8(C ) -- 33(H ) | 0.28 | 0.04 | -0.32 | -0.28 | -0.98 | 0.02 | Covalent bond |
| 115 | 9(C ) -- 34(H ) | 0.28 | 0.04 | -0.32 | -0.28 | -0.98 | 0.01 | Covalent bond |

**Table S49:** Topological parameters and energy density descriptors for compound **2b** (Gas phase) at bond critical points (BCP), including atomic connectivity, electron density ρ(r) (a.u.), Lagrangian kinetic energy density G(r) (a.u.), potential energy density V(r) (a.u.), total energy density E(r) or H(r) (a.u.), Laplacian of electron density ∇²ρ(r) (a.u.), ellipticity ε (dimensionless), and bond type.

| CP | Connected atoms | ρ(r) | G(r) | V(r) | H(r) | ∇²ρ(r) | ε | Bond Type |
| --- | --- | --- | --- | --- | --- | --- | --- | --- |
| 56 | 47(H ) -- 28(C ) | 0.28 | 0.04 | -0.32 | -0.28 | -0.97 | 0.02 | Covalent bond |
| 57 | 55(H ) -- 27(C ) | 0.28 | 0.04 | -0.32 | -0.28 | -0.97 | 0.01 | Covalent bond |
| 58 | 28(C ) -- 27(C ) | 0.31 | 0.10 | -0.42 | -0.32 | -0.86 | 0.19 | Covalent bond |
| 59 | 28(C ) -- 29(C ) | 0.31 | 0.10 | -0.43 | -0.32 | -0.88 | 0.20 | Covalent bond |
| 60 | 48(H ) -- 29(C ) | 0.28 | 0.04 | -0.32 | -0.28 | -0.99 | 0.01 | Covalent bond |
| 61 | 27(C ) -- 26(C ) | 0.31 | 0.10 | -0.42 | -0.32 | -0.87 | 0.20 | Covalent bond |
| 63 | 29(C ) -- 23(C ) | 0.31 | 0.10 | -0.41 | -0.31 | -0.85 | 0.18 | Covalent bond |
| 64 | 49(H ) -- 31(C ) | 0.28 | 0.04 | -0.32 | -0.28 | -0.99 | 0.04 | Covalent bond |
| 65 | 26(C ) -- 46(H ) | 0.28 | 0.04 | -0.32 | -0.28 | -0.97 | 0.02 | Covalent bond |
| 66 | 51(H ) -- 31(C ) | 0.28 | 0.04 | -0.31 | -0.27 | -0.94 | 0.04 | Covalent bond |
| 67 | 26(C ) -- 25(C ) | 0.31 | 0.10 | -0.42 | -0.32 | -0.87 | 0.19 | Covalent bond |
| 68 | 23(C ) -- 25(C ) | 0.31 | 0.10 | -0.41 | -0.31 | -0.84 | 0.18 | Covalent bond |
| 69 | 23(C ) -- 22(C ) | 0.27 | 0.07 | -0.30 | -0.23 | -0.67 | 0.10 | Covalent bond |
| 70 | 31(C ) -- 30(O ) | 0.25 | 0.27 | -0.61 | -0.34 | -0.26 | 0.01 | Covalent bond |
| 71 | 24(O ) -- 22(C ) | 0.41 | 0.68 | -1.36 | -0.68 | -0.01 | 0.05 | Covalent bond |
| 72 | 31(C ) -- 50(H ) | 0.28 | 0.04 | -0.31 | -0.27 | -0.94 | 0.04 | Covalent bond |
| 73 | 25(C ) -- 45(H ) | 0.28 | 0.04 | -0.32 | -0.28 | -0.99 | 0.01 | Covalent bond |
| **75** | **45(H ) -- 44(H )** | **0.01** | **0.01** | **-0.01** | **0.00** | **0.04** | **2.03** | **Van der Waal** |
| 76 | 22(C ) -- 21(C ) | 0.25 | 0.06 | -0.27 | -0.21 | -0.60 | 0.04 | Covalent bond |
| 77 | 44(H ) -- 21(C ) | 0.28 | 0.04 | -0.31 | -0.27 | -0.94 | 0.00 | Covalent bond |
| **78** | **45(H ) -- 4(N )** | **0.02** | **0.01** | **-0.01** | **0.00** | **0.05** | **0.10** | **H-bond** |
| **79** | **30(O ) -- 39(H )** | **0.02** | **0.02** | **-0.01** | **0.00** | **0.08** | **0.88** | **H-Bond** |
| 80 | 30(O ) -- 20(C ) | 0.29 | 0.34 | -0.75 | -0.41 | -0.27 | 0.01 | Covalent bond |
| 81 | 21(C ) -- 43(H ) | 0.28 | 0.04 | -0.31 | -0.28 | -0.96 | 0.01 | Covalent bond |
| 83 | 42(H ) -- 19(C ) | 0.28 | 0.04 | -0.32 | -0.28 | -0.96 | 0.03 | Covalent bond |
| **84** | **44(H ) -- 4(N )** | **0.02** | **0.01** | **-0.01** | **0.00** | **0.07** | **0.36** | **H-bond** |
| 85 | 20(C ) -- 19(C ) | 0.31 | 0.11 | -0.43 | -0.33 | -0.87 | 0.27 | Covalent bond |
| 86 | 39(H ) -- 14(C ) | 0.29 | 0.04 | -0.32 | -0.28 | -1.00 | 0.01 | Covalent bond |
| 88 | 21(C ) -- 13(S ) | 0.17 | 0.05 | -0.16 | -0.11 | -0.25 | 0.06 | Covalent bond |
| 89 | 20(C ) -- 15(C ) | 0.30 | 0.09 | -0.38 | -0.29 | -0.80 | 0.22 | Covalent bond |
| 90 | 19(C ) -- 18(C ) | 0.30 | 0.10 | -0.41 | -0.31 | -0.83 | 0.21 | Covalent bond |
| 91 | 14(C ) -- 15(C ) | 0.28 | 0.08 | -0.34 | -0.26 | -0.73 | 0.11 | Covalent bond |
| 92 | 14(C ) -- 3(C ) | 0.33 | 0.12 | -0.47 | -0.35 | -0.94 | 0.28 | Covalent bond |
| 94 | 4(N ) -- 3(C ) | 0.30 | 0.16 | -0.54 | -0.38 | -0.86 | 0.09 | Covalent bond |
| 95 | 4(N ) -- 5(C ) | 0.38 | 0.30 | -0.88 | -0.58 | -1.11 | 0.25 | Covalent bond |
| 96 | 18(C ) -- 41(H ) | 0.28 | 0.04 | -0.32 | -0.28 | -0.96 | 0.02 | Covalent bond |
| 97 | 13(S ) -- 5(C ) | 0.20 | 0.06 | -0.22 | -0.16 | -0.39 | 0.26 | Covalent bond |
| 98 | 15(C ) -- 16(C ) | 0.30 | 0.10 | -0.41 | -0.31 | -0.83 | 0.20 | Covalent bond |
| 99 | 18(C ) -- 17(C ) | 0.31 | 0.10 | -0.42 | -0.32 | -0.86 | 0.25 | Covalent bond |
| 101 | 3(C ) -- 2(C ) | 0.27 | 0.07 | -0.30 | -0.24 | -0.67 | 0.15 | Covalent bond |
| 102 | 5(C ) -- 1(N ) | 0.30 | 0.19 | -0.60 | -0.40 | -0.84 | 0.11 | Covalent bond |
| 104 | 16(C ) -- 17(C ) | 0.31 | 0.10 | -0.43 | -0.33 | -0.89 | 0.23 | Covalent bond |
| 105 | 16(C ) -- 40(H ) | 0.29 | 0.03 | -0.32 | -0.29 | -1.01 | 0.02 | Covalent bond |
| 106 | 17(C ) -- 32(O ) | 0.29 | 0.33 | -0.74 | -0.41 | -0.30 | 0.01 | Covalent bond |
| 107 | 2(C ) -- 1(N ) | 0.29 | 0.18 | -0.55 | -0.37 | -0.79 | 0.10 | Covalent bond |
| 108 | 2(C ) -- 12(O ) | 0.41 | 0.65 | -1.34 | -0.69 | -0.15 | 0.08 | Covalent bond |
| 109 | 53(H ) -- 33(C ) | 0.28 | 0.04 | -0.31 | -0.27 | -0.94 | 0.04 | Covalent bond |
| **110** | **40(H ) -- 12(O )** | **0.02** | **0.02** | **-0.02** | **0.00** | **0.09** | **0.02** | **H-bond** |
| 111 | 1(N ) -- 6(C ) | 0.28 | 0.19 | -0.55 | -0.36 | -0.69 | 0.03 | Covalent bond |
| 112 | 38(H ) -- 11(C ) | 0.28 | 0.04 | -0.32 | -0.28 | -0.98 | 0.01 | Covalent bond |
| 113 | 32(O ) -- 33(C ) | 0.25 | 0.27 | -0.62 | -0.35 | -0.30 | 0.01 | Covalent bond |
| 114 | 33(C ) -- 54(H ) | 0.28 | 0.04 | -0.31 | -0.27 | -0.94 | 0.04 | Covalent bond |
| 115 | 6(C ) -- 11(C ) | 0.31 | 0.10 | -0.42 | -0.32 | -0.88 | 0.23 | Covalent bond |
| 116 | 33(C ) -- 52(H ) | 0.28 | 0.04 | -0.32 | -0.28 | -0.99 | 0.04 | Covalent bond |
| 117 | 6(C ) -- 7(C ) | 0.31 | 0.10 | -0.42 | -0.32 | -0.87 | 0.24 | Covalent bond |
| 118 | 11(C ) -- 10(C ) | 0.31 | 0.10 | -0.42 | -0.32 | -0.87 | 0.20 | Covalent bond |
| 119 | 34(H ) -- 7(C ) | 0.28 | 0.04 | -0.32 | -0.28 | -0.97 | 0.02 | Covalent bond |
| 121 | 10(C ) -- 37(H ) | 0.28 | 0.04 | -0.32 | -0.28 | -0.97 | 0.02 | Covalent bond |
| 122 | 7(C ) -- 8(C ) | 0.31 | 0.10 | -0.42 | -0.32 | -0.86 | 0.20 | Covalent bond |
| 123 | 10(C ) -- 9(C ) | 0.31 | 0.10 | -0.42 | -0.32 | -0.86 | 0.20 | Covalent bond |
| 124 | 8(C ) -- 9(C ) | 0.31 | 0.10 | -0.42 | -0.32 | -0.87 | 0.20 | Covalent bond |
| 125 | 8(C ) -- 35(H ) | 0.28 | 0.04 | -0.32 | -0.28 | -0.97 | 0.02 | Covalent bond |
| 126 | 9(C ) -- 36(H ) | 0.28 | 0.04 | -0.32 | -0.28 | -0.97 | 0.02 | Covalent bond |

**Table S50:** Topological parameters and energy density descriptors for compound **2b** (DMSO phase) at bond critical points (BCP), including atomic connectivity, electron density ρ(r) (a.u.), Lagrangian kinetic energy density G(r) (a.u.), potential energy density V(r) (a.u.), total energy density E(r) or H(r) (a.u.), Laplacian of electron density ∇²ρ(r) (a.u.), ellipticity ε (dimensionless), and bond type.

| CP | Connected atoms | ρ(r) | G(r) | V(r) | H(r) | ∇²ρ(r) | ε | Bond Type |
| --- | --- | --- | --- | --- | --- | --- | --- | --- |
| 56 | 43(H ) -- 21(C ) | 0.28 | 0.04 | -0.31 | -0.28 | -0.96 | 0.01 | Covalent bond |
| 57 | 44(H ) -- 21(C ) | 0.28 | 0.04 | -0.31 | -0.27 | -0.95 | 0.01 | Covalent bond |
| 58 | 24(O ) -- 22(C ) | 0.40 | 0.67 | -1.34 | -0.67 | -0.03 | 0.04 | Covalent bond |
| 59 | 49(H ) -- 31(C ) | 0.28 | 0.03 | -0.32 | -0.28 | -0.99 | 0.04 | Covalent bond |
| 60 | 21(C ) -- 22(C ) | 0.26 | 0.06 | -0.27 | -0.21 | -0.62 | 0.03 | Covalent bond |
| 61 | 51(H ) -- 31(C ) | 0.28 | 0.04 | -0.31 | -0.28 | -0.96 | 0.04 | Covalent bond |
| 62 | 22(C ) -- 23(C ) | 0.27 | 0.06 | -0.30 | -0.23 | -0.67 | 0.09 | Covalent bond |
| 63 | 45(H ) -- 25(C ) | 0.28 | 0.04 | -0.32 | -0.28 | -0.99 | 0.01 | Covalent bond |
| 64 | 21(C ) -- 13(S ) | 0.17 | 0.05 | -0.15 | -0.11 | -0.23 | 0.08 | Covalent bond |
| 65 | 31(C ) -- 30(O ) | 0.24 | 0.27 | -0.59 | -0.32 | -0.21 | 0.00 | Covalent bond |
| 66 | 23(C ) -- 25(C ) | 0.30 | 0.10 | -0.41 | -0.31 | -0.84 | 0.19 | Covalent bond |
| 67 | 31(C ) -- 50(H ) | 0.28 | 0.04 | -0.31 | -0.28 | -0.96 | 0.04 | Covalent bond |
| 68 | 23(C ) -- 29(C ) | 0.30 | 0.10 | -0.41 | -0.31 | -0.84 | 0.18 | Covalent bond |
| **69** | **45(H ) -- 4(N )** | **0.01** | **0.01** | **-0.01** | **0.00** | **0.04** | **0.70** | **H-bond** |
| 70 | 25(C ) -- 26(C ) | 0.31 | 0.10 | -0.42 | -0.32 | -0.87 | 0.19 | Covalent bond |
| 71 | 48(H ) -- 29(C ) | 0.28 | 0.04 | -0.32 | -0.28 | -0.98 | 0.01 | Covalent bond |
| **74** | **30(O ) -- 39(H )** | **0.02** | **0.02** | **-0.01** | **0.00** | **0.08** | **1.10** | **H-bond** |
| 75 | 26(C ) -- 46(H ) | 0.28 | 0.04 | -0.32 | -0.28 | -0.98 | 0.02 | Covalent bond |
| 77 | 29(C ) -- 28(C ) | 0.31 | 0.10 | -0.43 | -0.32 | -0.88 | 0.20 | Covalent bond |
| 78 | 39(H ) -- 14(C ) | 0.29 | 0.03 | -0.32 | -0.28 | -1.00 | 0.01 | Covalent bond |
| 79 | 26(C ) -- 27(C ) | 0.31 | 0.10 | -0.42 | -0.32 | -0.87 | 0.19 | Covalent bond |
| 80 | 30(O ) -- 20(C ) | 0.29 | 0.35 | -0.77 | -0.42 | -0.29 | 0.01 | Covalent bond |
| 81 | 13(S ) -- 5(C ) | 0.20 | 0.06 | -0.21 | -0.15 | -0.37 | 0.17 | Covalent bond |
| 82 | 28(C ) -- 27(C ) | 0.31 | 0.10 | -0.41 | -0.31 | -0.86 | 0.19 | Covalent bond |
| 83 | 4(N ) -- 5(C ) | 0.38 | 0.32 | -0.90 | -0.59 | -1.08 | 0.28 | Covalent bond |
| 84 | 28(C ) -- 47(H ) | 0.28 | 0.04 | -0.32 | -0.28 | -0.98 | 0.02 | Covalent bond |
| 85 | 4(N ) -- 3(C ) | 0.30 | 0.16 | -0.54 | -0.38 | -0.86 | 0.09 | Covalent bond |
| 86 | 27(C ) -- 55(H ) | 0.28 | 0.04 | -0.32 | -0.28 | -0.98 | 0.01 | Covalent bond |
| 87 | 42(H ) -- 19(C ) | 0.28 | 0.04 | -0.32 | -0.28 | -0.97 | 0.02 | Covalent bond |
| 88 | 14(C ) -- 3(C ) | 0.32 | 0.12 | -0.47 | -0.35 | -0.93 | 0.27 | Covalent bond |
| 89 | 20(C ) -- 19(C ) | 0.31 | 0.11 | -0.43 | -0.32 | -0.86 | 0.25 | Covalent bond |
| 91 | 20(C ) -- 15(C ) | 0.29 | 0.09 | -0.38 | -0.29 | -0.80 | 0.22 | Covalent bond |
| 92 | 14(C ) -- 15(C ) | 0.28 | 0.08 | -0.34 | -0.26 | -0.74 | 0.11 | Covalent bond |
| 93 | 5(C ) -- 1(N ) | 0.30 | 0.20 | -0.60 | -0.40 | -0.83 | 0.13 | Covalent bond |
| **96** | **28(C ) -- 38(H )** | **0.01** | **0.00** | **0.00** | **0.00** | **0.02** | **0.87** | **Van der Waal** |
| 97 | 3(C ) -- 2(C ) | 0.27 | 0.07 | -0.31 | -0.24 | -0.67 | 0.15 | Covalent bond |
| 98 | 19(C ) -- 18(C ) | 0.30 | 0.10 | -0.41 | -0.31 | -0.84 | 0.21 | Covalent bond |
| 100 | 15(C ) -- 16(C ) | 0.30 | 0.10 | -0.40 | -0.30 | -0.82 | 0.20 | Covalent bond |
| 101 | 1(N ) -- 2(C ) | 0.30 | 0.19 | -0.57 | -0.39 | -0.81 | 0.11 | Covalent bond |
| 103 | 1(N ) -- 6(C ) | 0.27 | 0.19 | -0.55 | -0.36 | -0.68 | 0.03 | Covalent bond |
| 104 | 2(C ) -- 12(O ) | 0.40 | 0.63 | -1.30 | -0.68 | -0.19 | 0.07 | Covalent bond |
| 105 | 38(H ) -- 11(C ) | 0.28 | 0.04 | -0.32 | -0.28 | -0.99 | 0.01 | Covalent bond |
| 106 | 18(C ) -- 41(H ) | 0.28 | 0.04 | -0.32 | -0.28 | -0.98 | 0.02 | Covalent bond |
| 107 | 18(C ) -- 17(C ) | 0.31 | 0.10 | -0.42 | -0.32 | -0.86 | 0.23 | Covalent bond |
| 108 | 16(C ) -- 17(C ) | 0.31 | 0.10 | -0.43 | -0.33 | -0.89 | 0.24 | Covalent bond |
| 109 | 6(C ) -- 11(C ) | 0.31 | 0.10 | -0.43 | -0.32 | -0.88 | 0.23 | Covalent bond |
| 110 | 16(C ) -- 40(H ) | 0.29 | 0.03 | -0.32 | -0.29 | -1.00 | 0.02 | Covalent bond |
| 111 | 6(C ) -- 7(C ) | 0.31 | 0.10 | -0.42 | -0.32 | -0.87 | 0.24 | Covalent bond |
| 112 | **40(H ) -- 12(O )** | **0.03** | **0.02** | **-0.02** | **0.00** | **0.09** | **0.03** | **H-bond** |
| 113 | 34(H ) -- 7(C ) | 0.28 | 0.04 | -0.31 | -0.28 | -0.97 | 0.02 | Covalent bond |
| 114 | 11(C ) -- 10(C ) | 0.31 | 0.10 | -0.42 | -0.32 | -0.87 | 0.20 | Covalent bond |
| 115 | 17(C ) -- 32(O ) | 0.29 | 0.33 | -0.74 | -0.41 | -0.32 | 0.02 | Covalent bond |
| 117 | 7(C ) -- 8(C ) | 0.31 | 0.10 | -0.42 | -0.32 | -0.86 | 0.20 | Covalent bond |
| 118 | 10(C ) -- 37(H ) | 0.28 | 0.04 | -0.32 | -0.28 | -0.98 | 0.02 | Covalent bond |
| 119 | 10(C ) -- 9(C ) | 0.31 | 0.10 | -0.42 | -0.32 | -0.86 | 0.19 | Covalent bond |
| 120 | 8(C ) -- 9(C ) | 0.31 | 0.10 | -0.42 | -0.32 | -0.86 | 0.20 | Covalent bond |
| 121 | 8(C ) -- 35(H ) | 0.28 | 0.04 | -0.32 | -0.28 | -0.98 | 0.02 | Covalent bond |
| 122 | 53(H ) -- 33(C ) | 0.28 | 0.04 | -0.31 | -0.27 | -0.95 | 0.04 | Covalent bond |
| 123 | 32(O ) -- 33(C ) | 0.25 | 0.27 | -0.59 | -0.33 | -0.25 | 0.00 | Covalent bond |
| 124 | 9(C ) -- 36(H ) | 0.28 | 0.04 | -0.32 | -0.28 | -0.98 | 0.02 | Covalent bond |
| 125 | 33(C ) -- 54(H ) | 0.28 | 0.04 | -0.31 | -0.27 | -0.95 | 0.04 | Covalent bond |
| 126 | 33(C ) -- 52(H ) | 0.28 | 0.04 | -0.32 | -0.28 | -0.99 | 0.04 | Covalent bond |

**Table S51:** Topological parameters and energy density descriptors for compound **2c** (Gas phase) at bond critical points (BCP), including atomic connectivity, electron density ρ(r) (a.u.), Lagrangian kinetic energy density G(r) (a.u.), potential energy density V(r) (a.u.), total energy density E(r) or H(r) (a.u.), Laplacian of electron density ∇²ρ(r) (a.u.), ellipticity ε (dimensionless), and bond type.

| CP | Connected atoms | ρ(r) | G(r) | V(r) | H(r) | ∇²ρ(r) | ε | Bond Type |
| --- | --- | --- | --- | --- | --- | --- | --- | --- |
| 56 | 47(H ) -- 28(C ) | 0.28 | 0.04 | -0.32 | -0.28 | -0.98 | 0.02 | Covalent bond |
| 57 | 55(Cl) -- 27(C ) | 0.20 | 0.07 | -0.21 | -0.15 | -0.31 | 0.06 | Covalent bond |
| 58 | 28(C ) -- 27(C ) | 0.31 | 0.10 | -0.42 | -0.32 | -0.87 | 0.22 | Covalent bond |
| 59 | 28(C ) -- 29(C ) | 0.31 | 0.11 | -0.43 | -0.32 | -0.88 | 0.21 | Covalent bond |
| 60 | 27(C ) -- 26(C ) | 0.31 | 0.11 | -0.43 | -0.32 | -0.88 | 0.23 | Covalent bond |
| 61 | 48(H ) -- 29(C ) | 0.28 | 0.03 | -0.32 | -0.28 | -0.99 | 0.01 | Covalent bond |
| 63 | 26(C ) -- 46(H ) | 0.28 | 0.04 | -0.32 | -0.28 | -0.98 | 0.02 | Covalent bond |
| 64 | 51(H ) -- 31(C ) | 0.28 | 0.04 | -0.31 | -0.27 | -0.94 | 0.04 | Covalent bond |
| 65 | 29(C ) -- 23(C ) | 0.31 | 0.10 | -0.41 | -0.31 | -0.85 | 0.18 | Covalent bond |
| 66 | 26(C ) -- 25(C ) | 0.31 | 0.10 | -0.42 | -0.32 | -0.86 | 0.20 | Covalent bond |
| 67 | 49(H ) -- 31(C ) | 0.28 | 0.04 | -0.32 | -0.28 | -0.99 | 0.04 | Covalent bond |
| 68 | 23(C ) -- 25(C ) | 0.31 | 0.10 | -0.41 | -0.31 | -0.85 | 0.18 | Covalent bond |
| 69 | 31(C ) -- 30(O ) | 0.25 | 0.27 | -0.61 | -0.34 | -0.26 | 0.01 | Covalent bond |
| 70 | 23(C ) -- 22(C ) | 0.27 | 0.07 | -0.30 | -0.23 | -0.67 | 0.10 | Covalent bond |
| 71 | 31(C ) -- 50(H ) | 0.28 | 0.04 | -0.31 | -0.27 | -0.94 | 0.04 | Covalent bond |
| 72 | 25(C ) -- 45(H ) | 0.28 | 0.04 | -0.32 | -0.28 | -1.00 | 0.01 | Covalent bond |
| 73 | 24(O ) -- 22(C ) | 0.41 | 0.68 | -1.36 | -0.68 | -0.02 | 0.05 | Covalent bond |
| 75 | 42(H ) -- 19(C ) | 0.28 | 0.04 | -0.32 | -0.28 | -0.96 | 0.03 | Covalent bond |
| **76** | **45(H ) -- 44(H )** | **0.01** | **0.01** | **-0.01** | **0.00** | **0.04** | **2.16** | **Van der Waal** |
| 77 | 30(O ) -- 20(C ) | 0.29 | 0.34 | -0.75 | -0.41 | -0.27 | 0.01 | Covalent bond |
| 78 | 22(C ) -- 21(C ) | 0.25 | 0.06 | -0.27 | -0.21 | -0.60 | 0.04 | Covalent bond |
| **79** | **30(O ) -- 39(H )** | **0.02** | **0.02** | **-0.01** | **0.00** | **0.08** | **0.87** | **H-bond** |
| **80** | **45(H ) -- 4(N )** | **0.02** | **0.01** | **-0.01** | **0.00** | **0.05** | **0.10** | **H-bond** |
| 83 | 19(C ) -- 20(C ) | 0.31 | 0.11 | -0.43 | -0.33 | -0.87 | 0.27 | Covalent bond |
| 84 | 44(H ) -- 21(C ) | 0.28 | 0.04 | -0.31 | -0.27 | -0.94 | 0.00 | Covalent bond |
| 85 | 19(C ) -- 18(C ) | 0.30 | 0.10 | -0.41 | -0.31 | -0.83 | 0.21 | Covalent bond |
| 86 | 39(H ) -- 14(C ) | 0.29 | 0.04 | -0.32 | -0.28 | -1.00 | 0.01 | Covalent bond |
| 87 | 20(C ) -- 15(C ) | 0.30 | 0.09 | -0.38 | -0.29 | -0.80 | 0.22 | Covalent bond |
| **88** | **44(H ) -- 4(N )** | **0.02** | **0.01** | **-0.01** | **0.00** | **0.06** | **0.37** | **H-bond** |
| 89 | 21(C ) -- 43(H ) | 0.28 | 0.04 | -0.31 | -0.28 | -0.96 | 0.01 | Covalent bond |
| 90 | 41(H ) -- 18(C ) | 0.28 | 0.04 | -0.32 | -0.28 | -0.96 | 0.02 | Covalent bond |
| 92 | 14(C ) -- 15(C ) | 0.28 | 0.08 | -0.34 | -0.26 | -0.73 | 0.11 | Covalent bond |
| 94 | 21(C ) -- 13(S ) | 0.17 | 0.05 | -0.16 | -0.11 | -0.25 | 0.07 | Covalent bond |
| 95 | 14(C ) -- 3(C ) | 0.33 | 0.12 | -0.47 | -0.35 | -0.94 | 0.28 | Covalent bond |
| 96 | 18(C ) -- 17(C ) | 0.31 | 0.10 | -0.42 | -0.32 | -0.86 | 0.25 | Covalent bond |
| 97 | 15(C ) -- 16(C ) | 0.30 | 0.10 | -0.41 | -0.31 | -0.83 | 0.20 | Covalent bond |
| 98 | 4(N ) -- 3(C ) | 0.30 | 0.16 | -0.54 | -0.38 | -0.86 | 0.09 | Covalent bond |
| 99 | 17(C ) -- 16(C ) | 0.31 | 0.10 | -0.43 | -0.33 | -0.89 | 0.23 | Covalent bond |
| 100 | 4(N ) -- 5(C ) | 0.38 | 0.30 | -0.88 | -0.58 | -1.11 | 0.25 | Covalent bond |
| 101 | 53(H ) -- 33(C ) | 0.28 | 0.04 | -0.31 | -0.27 | -0.94 | 0.04 | Covalent bond |
| 102 | 17(C ) -- 32(O ) | 0.29 | 0.33 | -0.74 | -0.41 | -0.30 | 0.02 | Covalent bond |
| 103 | 13(S ) -- 5(C ) | 0.20 | 0.06 | -0.22 | -0.16 | -0.39 | 0.26 | Covalent bond |
| 105 | 3(C ) -- 2(C ) | 0.27 | 0.07 | -0.30 | -0.24 | -0.67 | 0.15 | Covalent bond |
| 107 | 16(C ) -- 40(H ) | 0.29 | 0.03 | -0.32 | -0.29 | -1.01 | 0.02 | Covalent bond |
| 108 | 5(C ) -- 1(N ) | 0.30 | 0.19 | -0.60 | -0.40 | -0.84 | 0.12 | Covalent bond |
| 109 | 33(C ) -- 32(O ) | 0.25 | 0.27 | -0.62 | -0.35 | -0.30 | 0.01 | Covalent bond |
| **110** | **40(H ) -- 12(O )** | **0.02** | **0.02** | **-0.02** | **0.00** | **0.09** | **0.02** | **H-bond** |
| 111 | 2(C ) -- 1(N ) | 0.29 | 0.18 | -0.55 | -0.37 | -0.78 | 0.10 | Covalent bond |
| 112 | 2(C ) -- 12(O ) | 0.41 | 0.65 | -1.35 | -0.69 | -0.15 | 0.08 | Covalent bond |
| 113 | 33(C ) -- 54(H ) | 0.28 | 0.04 | -0.31 | -0.27 | -0.94 | 0.04 | Covalent bond |
| 114 | 38(H ) -- 11(C ) | 0.28 | 0.04 | -0.32 | -0.28 | -0.98 | 0.01 | Covalent bond |
| 115 | 33(C ) -- 52(H ) | 0.28 | 0.04 | -0.32 | -0.28 | -0.99 | 0.04 | Covalent bond |
| 116 | 1(N ) -- 6(C ) | 0.28 | 0.19 | -0.55 | -0.36 | -0.69 | 0.03 | Covalent bond |
| 117 | 11(C ) -- 6(C ) | 0.31 | 0.10 | -0.42 | -0.32 | -0.88 | 0.23 | Covalent bond |
| 118 | 11(C ) -- 10(C ) | 0.31 | 0.10 | -0.42 | -0.32 | -0.87 | 0.20 | Covalent bond |
| 119 | 37(H ) -- 10(C ) | 0.28 | 0.04 | -0.32 | -0.28 | -0.97 | 0.02 | Covalent bond |
| 120 | 6(C ) -- 7(C ) | 0.31 | 0.10 | -0.42 | -0.32 | -0.87 | 0.24 | Covalent bond |
| 122 | 10(C ) -- 9(C ) | 0.31 | 0.10 | -0.42 | -0.32 | -0.86 | 0.20 | Covalent bond |
| 123 | 7(C ) -- 34(H ) | 0.28 | 0.04 | -0.32 | -0.28 | -0.97 | 0.02 | Covalent bond |
| 124 | 7(C ) -- 8(C ) | 0.31 | 0.10 | -0.42 | -0.32 | -0.86 | 0.20 | Covalent bond |
| 125 | 9(C ) -- 8(C ) | 0.31 | 0.10 | -0.42 | -0.32 | -0.87 | 0.20 | Covalent bond |
| 126 | 9(C ) -- 36(H ) | 0.28 | 0.04 | -0.32 | -0.28 | -0.97 | 0.02 | Covalent bond |
| 127 | 8(C ) -- 35(H ) | 0.28 | 0.04 | -0.32 | -0.28 | -0.97 | 0.02 | Covalent bond |

**Table S52:** Topological parameters and energy density descriptors for compound **2c** (DMSO phase) at bond critical points (BCP), including atomic connectivity, electron density ρ(r) (a.u.), Lagrangian kinetic energy density G(r) (a.u.), potential energy density V(r) (a.u.), total energy density E(r) or H(r) (a.u.), Laplacian of electron density ∇²ρ(r) (a.u.), ellipticity ε (dimensionless), and bond type.

| CP | Connected atoms | ρ(r) | G(r) | V(r) | H(r) | ∇²ρ(r) | ε | Bond Type |
| --- | --- | --- | --- | --- | --- | --- | --- | --- |
| 56 | 43(H ) -- 21(C ) | 0.14 | 0.04 | -0.31 | -0.27 | -0.95 | 0.00 | Covalent bond |
| 57 | 45(H ) -- 25(C ) | 0.14 | 0.03 | -0.32 | -0.28 | -0.99 | 0.01 | Covalent bond |
| 58 | 24(O ) -- 22(C ) | 0.20 | 0.67 | -1.35 | -0.68 | -0.02 | 0.04 | Covalent bond |
| 59 | 44(H ) -- 21(C ) | 0.14 | 0.04 | -0.31 | -0.28 | -0.97 | 0.01 | Covalent bond |
| 60 | 22(C ) -- 21(C ) | 0.13 | 0.06 | -0.27 | -0.21 | -0.61 | 0.03 | Covalent bond |
| 61 | 22(C ) -- 23(C ) | 0.13 | 0.06 | -0.30 | -0.23 | -0.68 | 0.08 | Covalent bond |
| 62 | 25(C ) -- 23(C ) | 0.15 | 0.10 | -0.41 | -0.31 | -0.84 | 0.19 | Covalent bond |
| 63 | 25(C ) -- 26(C ) | 0.15 | 0.10 | -0.42 | -0.32 | -0.86 | 0.20 | Covalent bond |
| 64 | 46(H ) -- 26(C ) | 0.14 | 0.03 | -0.32 | -0.28 | -0.99 | 0.02 | Covalent bond |
| 65 | 49(H ) -- 31(C ) | 0.14 | 0.04 | -0.32 | -0.28 | -0.99 | 0.04 | Covalent bond |
| 66 | 21(C ) -- 13(S ) | 0.08 | 0.05 | -0.15 | -0.11 | -0.23 | 0.08 | Week covalent |
| 57 | 23(C ) -- 29(C ) | 0.15 | 0.10 | -0.41 | -0.31 | -0.84 | 0.19 | Covalent bond |
| 68 | 26(C ) -- 27(C ) | 0.16 | 0.10 | -0.43 | -0.32 | -0.87 | 0.22 | Covalent bond |
| 70 | 51(H ) -- 31(C ) | 0.14 | 0.04 | -0.31 | -0.28 | -0.96 | 0.04 | Covalent bond |
| **71** | **46(H ) -- 30(O )** | **0.00** | **0.00** | **0.00** | **0.00** | **0.02** | **0.17** | **Van der Waal** |
| **73** | **23(C ) -- 4(N )** | **0.00** | **0.01** | **0.00** | **0.00** | **0.03** | **14.06** | **Van der Waal** |
| 74 | 31(C ) -- 30(O ) | 0.12 | 0.27 | -0.59 | -0.32 | -0.21 | 0.00 | Covalent bond |
| 75 | 29(C ) -- 48(H ) | 0.14 | 0.03 | -0.32 | -0.28 | -0.99 | 0.01 | Covalent bond |
| 76 | 29(C ) -- 28(C ) | 0.15 | 0.10 | -0.42 | -0.32 | -0.87 | 0.20 | Covalent bond |
| 77 | 27(C ) -- 55(Cl) | 0.10 | 0.07 | -0.21 | -0.14 | -0.30 | 0.06 | Covalent bond |
| 78 | 27(C ) -- 28(C ) | 0.16 | 0.10 | -0.43 | -0.32 | -0.87 | 0.22 | Covalent bond |
| 79 | 31(C ) -- 50(H ) | 0.14 | 0.04 | -0.31 | -0.28 | -0.96 | 0.04 | Covalent bond |
| **81** | **26(C ) -- 14(C )** | **0.00** | **0.00** | **0.00** | **0.00** | **0.02** | **0.17** | **Van der Waal** |
| 82 | 28(C ) -- 47(H ) | 0.14 | 0.03 | -0.32 | -0.28 | -0.98 | 0.02 | Covalent bond |
| 83 | 13(S ) -- 5(C ) | 0.10 | 0.06 | -0.21 | -0.15 | -0.37 | 0.16 | Covalent bond |
| **84** | **30(O ) -- 39(H )** | **0.01** | **0.02** | **-0.01** | **0.00** | **0.08** | **1.41** | **H-bond** |
| **88** | **55(Cl) -- 20(C )** | **0.00** | **0.00** | **0.00** | **0.00** | **0.02** | **1.93** | **Halogen bond** |
| 89 | 4(N ) -- 5(C ) | 0.19 | 0.32 | -0.91 | -0.59 | -1.07 | 0.28 | Covalent bond |
| 90 | 39(H ) -- 14(C ) | 0.14 | 0.03 | -0.32 | -0.29 | -1.00 | 0.01 | Covalent bond |
| 91 | 30(O ) -- 20(C ) | 0.15 | 0.35 | -0.77 | -0.42 | -0.28 | 0.01 | Covalent bond |
| **92** | **48(H ) -- 38(H )** | **0.00** | **0.00** | **0.00** | **0.00** | **0.02** | **0.15** | **Van der Waal** |
| 94 | 4(N ) -- 3(C ) | 0.15 | 0.16 | -0.54 | -0.38 | -0.87 | 0.10 | Covalent bond |
| 95 | 5(C ) -- 1(N ) | 0.15 | 0.20 | -0.60 | -0.40 | -0.82 | 0.14 | Covalent bond |
| 96 | 14(C ) -- 3(C ) | 0.16 | 0.12 | -0.47 | -0.35 | -0.94 | 0.27 | Covalent bond |
| 98 | 42(H ) -- 19(C ) | 0.14 | 0.04 | -0.32 | -0.28 | -0.97 | 0.02 | Covalent bond |
| 99 | 20(C ) -- 19(C ) | 0.16 | 0.11 | -0.43 | -0.32 | -0.87 | 0.25 | Covalent bond |
| 100 | 20(C ) -- 15(C ) | 0.15 | 0.09 | -0.38 | -0.29 | -0.80 | 0.22 | Covalent bond |
| 101 | 14(C ) -- 15(C ) | 0.14 | 0.08 | -0.34 | -0.27 | -0.75 | 0.11 | Covalent bond |
| 102 | 3(C ) -- 2(C ) | 0.14 | 0.07 | -0.31 | -0.24 | -0.68 | 0.15 | Covalent bond |
| 103 | 1(N ) -- 2(C ) | 0.15 | 0.18 | -0.57 | -0.38 | -0.81 | 0.10 | Covalent bond |
| 104 | 38(H ) -- 11(C ) | 0.14 | 0.04 | -0.32 | -0.28 | -0.99 | 0.02 | Covalent bond |
| 105 | 1(N ) -- 6(C ) | 0.14 | 0.19 | -0.56 | -0.37 | -0.69 | 0.03 | Covalent bond |
| 106 | 19(C ) -- 18(C ) | 0.15 | 0.10 | -0.41 | -0.31 | -0.84 | 0.21 | Covalent bond |
| 107 | 6(C ) -- 11(C ) | 0.16 | 0.10 | -0.42 | -0.32 | -0.87 | 0.23 | Covalent bond |
| 109 | 15(C ) -- 16(C ) | 0.15 | 0.10 | -0.40 | -0.31 | -0.83 | 0.20 | Covalent bond |
| 111 | 6(C ) -- 7(C ) | 0.15 | 0.10 | -0.42 | -0.32 | -0.87 | 0.24 | Covalent bond |
| 112 | 2(C ) -- 12(O ) | 0.20 | 0.63 | -1.31 | -0.68 | -0.19 | 0.07 | Covalent bond |
| 113 | 11(C ) -- 10(C ) | 0.15 | 0.10 | -0.42 | -0.32 | -0.86 | 0.20 | Covalent bond |
| 114 | 34(H ) -- 7(C ) | 0.14 | 0.04 | -0.31 | -0.28 | -0.97 | 0.02 | Covalent bond |
| 116 | 10(C ) -- 37(H ) | 0.14 | 0.04 | -0.32 | -0.28 | -0.98 | 0.02 | Covalent bond |
| 117 | 7(C ) -- 8(C ) | 0.15 | 0.10 | -0.42 | -0.32 | -0.86 | 0.20 | Covalent bond |
| 118 | 10(C ) -- 9(C ) | 0.15 | 0.10 | -0.42 | -0.32 | -0.86 | 0.19 | Covalent bond |
| 119 | 18(C ) -- 41(H ) | 0.14 | 0.04 | -0.32 | -0.28 | -0.98 | 0.02 | Covalent bond |
| 120 | 18(C ) -- 17(C ) | 0.15 | 0.10 | -0.42 | -0.32 | -0.86 | 0.23 | Covalent bond |
| 121 | 16(C ) -- 17(C ) | 0.16 | 0.10 | -0.43 | -0.33 | -0.89 | 0.24 | Covalent bond |
| 122 | 8(C ) -- 9(C ) | 0.15 | 0.10 | -0.42 | -0.32 | -0.86 | 0.20 | Covalent bond |
| 123 | 16(C ) -- 40(H ) | 0.14 | 0.03 | -0.32 | -0.29 | -1.00 | 0.02 | Covalent bond |
| **124** | **12(O ) -- 40(H )** | **0.01** | **0.02** | **-0.02** | **0.00** | **0.09** | **0.05** | **H-bond** |
| 125 | 8(C ) -- 35(H ) | 0.14 | 0.04 | -0.32 | -0.28 | -0.98 | 0.02 | Covalent bond |
| 126 | 9(C ) -- 36(H ) | 0.14 | 0.04 | -0.32 | -0.28 | -0.98 | 0.02 | Covalent bond |
| 127 | 17(C ) -- 32(O ) | 0.14 | 0.33 | -0.74 | -0.41 | -0.32 | 0.02 | Covalent bond |
| 128 | 53(H ) -- 33(C ) | 0.14 | 0.04 | -0.31 | -0.27 | -0.95 | 0.04 | Covalent bond |
| 129 | 32(O ) -- 33(C ) | 0.12 | 0.27 | -0.60 | -0.33 | -0.26 | 0.00 | Covalent bond |
| 130 | 33(C ) -- 54(H ) | 0.14 | 0.04 | -0.31 | -0.27 | -0.95 | 0.04 | Covalent bond |
| 131 | 33(C ) -- 52(H ) | 0.14 | 0.04 | -0.32 | -0.28 | -0.99 | 0.04 | Covalent bond |

**Table S53:** Topological parameters and energy density descriptors for compound **2d** (Gas phase) at bond critical points (BCP), including atomic connectivity, electron density ρ(r) (a.u.), Lagrangian kinetic energy density G(r) (a.u.), potential energy density V(r) (a.u.), total energy density E(r) or H(r) (a.u.), Laplacian of electron density ∇²ρ(r) (a.u.), ellipticity ε (dimensionless), and bond type.

| CP | Connected atoms | ρ(r) | G(r) | V(r) | H(r) | ∇²ρ(r) | ε | Bond Type |
| --- | --- | --- | --- | --- | --- | --- | --- | --- |
| 56 | 51(H ) -- 31(C ) | 0.28 | 0.04 | -0.31 | -0.27 | -0.94 | 0.04 | Covalent bond |
| 57 | 49(H ) -- 31(C ) | 0.28 | 0.04 | -0.32 | -0.28 | -0.99 | 0.04 | Covalent bond |
| 58 | 31(C ) -- 50(H ) | 0.28 | 0.04 | -0.31 | -0.27 | -0.94 | 0.04 | Covalent bond |
| 59 | 31(C ) -- 30(O ) | 0.25 | 0.27 | -0.61 | -0.34 | -0.26 | 0.01 | Covalent bond |
| 60 | 42(H ) -- 19(C ) | 0.28 | 0.04 | -0.32 | -0.28 | -0.96 | 0.03 | Covalent bond |
| 61 | 30(O ) -- 20(C ) | 0.29 | 0.34 | -0.75 | -0.41 | -0.27 | 0.01 | Covalent bond |
| 62 | 55(Br) -- 27(C ) | 0.16 | 0.05 | -0.15 | -0.09 | -0.16 | 0.07 | Covalent bond |
| 63 | 19(C ) -- 20(C ) | 0.31 | 0.11 | -0.43 | -0.33 | -0.87 | 0.27 | Covalent bond |
| 64 | 19(C ) -- 18(C ) | 0.30 | 0.10 | -0.41 | -0.31 | -0.83 | 0.21 | Covalent bond |
| 65 | 46(H ) -- 26(C ) | 0.28 | 0.04 | -0.32 | -0.28 | -0.98 | 0.02 | Covalent bond |
| **66** | **30(O ) -- 39(H )** | **0.02** | **0.02** | **-0.01** | **0.00** | **0.08** | **0.87** | **H-bond** |
| 67 | 27(C ) -- 26(C ) | 0.31 | 0.11 | -0.43 | -0.32 | -0.87 | 0.22 | Covalent bond |
| 69 | 41(H ) -- 18(C ) | 0.28 | 0.04 | -0.32 | -0.28 | -0.96 | 0.02 | Covalent bond |
| 70 | 27(C ) -- 28(C ) | 0.31 | 0.10 | -0.42 | -0.32 | -0.86 | 0.21 | Covalent bond |
| 71 | 47(H ) -- 28(C ) | 0.28 | 0.04 | -0.32 | -0.28 | -0.98 | 0.02 | Covalent bond |
| 72 | 20(C ) -- 15(C ) | 0.30 | 0.09 | -0.38 | -0.29 | -0.80 | 0.22 | Covalent bond |
| 73 | 26(C ) -- 25(C ) | 0.31 | 0.10 | -0.42 | -0.32 | -0.86 | 0.20 | Covalent bond |
| 75 | 28(C ) -- 29(C ) | 0.31 | 0.11 | -0.43 | -0.32 | -0.88 | 0.21 | Covalent bond |
| 77 | 39(H ) -- 14(C ) | 0.29 | 0.04 | -0.32 | -0.28 | -1.00 | 0.01 | Covalent bond |
| 78 | 25(C ) -- 45(H ) | 0.28 | 0.04 | -0.32 | -0.28 | -1.00 | 0.01 | Covalent bond |
| 79 | 18(C ) -- 17(C ) | 0.31 | 0.10 | -0.42 | -0.32 | -0.86 | 0.25 | Covalent bond |
| 80 | 25(C ) -- 23(C ) | 0.31 | 0.10 | -0.41 | -0.31 | -0.85 | 0.18 | Covalent bond |
| 81 | 29(C ) -- 23(C ) | 0.31 | 0.10 | -0.41 | -0.31 | -0.85 | 0.18 | Covalent bond |
| 82 | 29(C ) -- 48(H ) | 0.28 | 0.03 | -0.32 | -0.28 | -0.99 | 0.01 | Covalent bond |
| 83 | 15(C ) -- 14(C ) | 0.28 | 0.08 | -0.34 | -0.26 | -0.73 | 0.11 | Covalent bond |
| **84** | **45(H ) -- 44(H )** | **0.01** | **0.01** | **-0.01** | **0.00** | **0.04** | **2.20** | **Van der Waal** |
| 86 | 23(C ) -- 22(C ) | 0.27 | 0.07 | -0.30 | -0.23 | -0.67 | 0.10 | Covalent bond |
| 87 | 15(C ) -- 16(C ) | 0.30 | 0.10 | -0.41 | -0.31 | -0.83 | 0.20 | Covalent bond |
| **88** | **45(H ) -- 4(N )** | **0.02** | **0.01** | **-0.01** | **0.00** | **0.05** | **0.10** | **H-Bond** |
| 90 | 53(H ) -- 33(C ) | 0.28 | 0.04 | -0.31 | -0.27 | -0.94 | 0.04 | Covalent bond |
| 91 | 17(C ) -- 16(C ) | 0.31 | 0.10 | -0.43 | -0.33 | -0.89 | 0.23 | Covalent bond |
| 92 | 22(C ) -- 24(O ) | 0.41 | 0.68 | -1.36 | -0.68 | -0.02 | 0.05 | Covalent bond |
| 93 | 17(C ) -- 32(O ) | 0.29 | 0.33 | -0.74 | -0.41 | -0.30 | 0.02 | Covalent bond |
| 94 | 14(C ) -- 3(C ) | 0.33 | 0.12 | -0.47 | -0.35 | -0.94 | 0.28 | Covalent bond |
| 95 | 44(H ) -- 21(C ) | 0.28 | 0.04 | -0.31 | -0.27 | -0.94 | 0.00 | Covalent bond |
| **96** | **44(H ) -- 4(N )** | **0.02** | **0.01** | **-0.01** | **0.00** | **0.07** | **0.36** | **H-bond** |
| 97 | 54(H ) -- 33(C ) | 0.28 | 0.04 | -0.31 | -0.27 | -0.94 | 0.04 | Covalent bond |
| 98 | 22(C ) -- 21(C ) | 0.25 | 0.06 | -0.27 | -0.21 | -0.60 | 0.04 | Covalent bond |
| 99 | 33(C ) -- 32(O ) | 0.25 | 0.27 | -0.62 | -0.35 | -0.30 | 0.01 | Covalent bond |
| 101 | 4(N ) -- 3(C ) | 0.30 | 0.16 | -0.54 | -0.38 | -0.86 | 0.09 | Covalent bond |
| 102 | 21(C ) -- 43(H ) | 0.28 | 0.04 | -0.31 | -0.28 | -0.96 | 0.01 | Covalent bond |
| 103 | 16(C ) -- 40(H ) | 0.29 | 0.03 | -0.32 | -0.29 | -1.01 | 0.02 | Covalent bond |
| 105 | 33(C ) -- 52(H ) | 0.28 | 0.04 | -0.32 | -0.28 | -0.99 | 0.04 | Covalent bond |
| 106 | 3(C ) -- 2(C ) | 0.27 | 0.07 | -0.31 | -0.24 | -0.67 | 0.15 | Covalent bond |
| 107 | 21(C ) -- 13(S ) | 0.17 | 0.05 | -0.16 | -0.11 | -0.25 | 0.07 | Covalent bond |
| 108 | 4(N ) -- 5(C ) | 0.38 | 0.30 | -0.88 | -0.58 | -1.11 | 0.25 | Covalent bond |
| **110** | **40(H ) -- 12(O )** | **0.02** | **0.02** | **-0.02** | **0.00** | **0.09** | **0.02** | **H-bond** |
| 111 | 5(C ) -- 13(S ) | 0.20 | 0.06 | -0.22 | -0.16 | -0.38 | 0.26 | Covalent bond |
| 112 | 5(C ) -- 1(N ) | 0.30 | 0.19 | -0.60 | -0.40 | -0.85 | 0.12 | Covalent bond |
| 113 | 2(C ) -- 12(O ) | 0.41 | 0.65 | -1.35 | -0.69 | -0.15 | 0.08 | Covalent bond |
| 114 | 2(C ) -- 1(N ) | 0.29 | 0.18 | -0.55 | -0.37 | -0.78 | 0.10 | Covalent bond |
| 115 | 1(N ) -- 6(C ) | 0.28 | 0.19 | -0.55 | -0.36 | -0.69 | 0.03 | Covalent bond |
| 116 | 38(H ) -- 11(C ) | 0.28 | 0.04 | -0.32 | -0.28 | -0.98 | 0.01 | Covalent bond |
| 117 | 6(C ) -- 11(C ) | 0.31 | 0.10 | -0.42 | -0.32 | -0.88 | 0.23 | Covalent bond |
| 118 | 6(C ) -- 7(C ) | 0.31 | 0.10 | -0.42 | -0.32 | -0.87 | 0.24 | Covalent bond |
| 119 | 11(C ) -- 10(C ) | 0.31 | 0.10 | -0.42 | -0.32 | -0.87 | 0.20 | Covalent bond |
| 120 | 34(H ) -- 7(C ) | 0.28 | 0.04 | -0.32 | -0.28 | -0.97 | 0.02 | Covalent bond |
| 122 | 10(C ) -- 37(H ) | 0.28 | 0.04 | -0.32 | -0.28 | -0.97 | 0.02 | Covalent bond |
| 123 | 7(C ) -- 8(C ) | 0.31 | 0.10 | -0.42 | -0.32 | -0.86 | 0.20 | Covalent bond |
| 124 | 10(C ) -- 9(C ) | 0.31 | 0.10 | -0.42 | -0.32 | -0.86 | 0.20 | Covalent bond |
| 125 | 8(C ) -- 9(C ) | 0.31 | 0.10 | -0.42 | -0.32 | -0.87 | 0.20 | Covalent bond |
| 126 | 8(C ) -- 35(H ) | 0.28 | 0.04 | -0.32 | -0.28 | -0.97 | 0.02 | Covalent bond |
| 127 | 9(C ) -- 36(H ) | 0.28 | 0.04 | -0.32 | -0.28 | -0.97 | 0.02 | Covalent bond |

**Table S54:** Topological parameters and energy density descriptors for compound **2d** (DMSO phase) at bond critical points (BCP), including atomic connectivity, electron density ρ(r) (a.u.), Lagrangian kinetic energy density G(r) (a.u.), potential energy density V(r) (a.u.), total energy density E(r) or H(r) (a.u.), Laplacian of electron density ∇²ρ(r) (a.u.), ellipticity ε (dimensionless), and bond type.

| CP | Connected atoms | ρ(r) | G(r) | V(r) | H(r) | ∇²ρ(r) | ε | Bond Type |
| --- | --- | --- | --- | --- | --- | --- | --- | --- |
| 56 | 49(H ) -- 31(C ) | 0.28 | 0.04 | -0.32 | -0.28 | -0.99 | 0.04 | Covalent bond |
| 57 | 51(H ) -- 31(C ) | 0.28 | 0.04 | -0.31 | -0.28 | -0.96 | 0.04 | Covalent bond |
| 58 | 45(H ) -- 25(C ) | 0.28 | 0.03 | -0.32 | -0.28 | -0.99 | 0.01 | Covalent bond |
| **59** | **45(H ) -- 44(H )** | **0.01** | **0.01** | **-0.01** | **0.00** | **0.04** | **2.43** | **Van der Waal** |
| 60 | 44(H ) -- 21(C ) | 0.28 | 0.04 | -0.32 | -0.28 | -0.97 | 0.01 | Covalent bond |
| 61 | 31(C ) -- 50(H ) | 0.28 | 0.04 | -0.31 | -0.28 | -0.96 | 0.04 | Covalent bond |
| 62 | 43(H ) -- 21(C ) | 0.28 | 0.04 | -0.31 | -0.27 | -0.95 | 0.00 | Covalent bond |
| 64 | 31(C ) -- 30(O ) | 0.24 | 0.27 | -0.59 | -0.32 | -0.21 | 0.00 | Covalent bond |
| 65 | 46(H ) -- 26(C ) | 0.28 | 0.03 | -0.32 | -0.28 | -0.99 | 0.02 | Covalent bond |
| 66 | 25(C ) -- 26(C ) | 0.31 | 0.10 | -0.42 | -0.32 | -0.86 | 0.20 | Covalent bond |
| 67 | 21(C ) -- 22(C ) | 0.25 | 0.06 | -0.27 | -0.21 | -0.61 | 0.04 | Covalent bond |
| 68 | 25(C ) -- 23(C ) | 0.31 | 0.10 | -0.41 | -0.31 | -0.85 | 0.19 | Covalent bond |
| 69 | 21(C ) -- 13(S ) | 0.17 | 0.05 | -0.15 | -0.11 | -0.23 | 0.08 | Covalent bond |
| **70** | **30(O ) -- 39(H )** | **0.02** | **0.02** | **-0.01** | **0.00** | **0.08** | **1.10** | **H-bond** |
| 71 | 26(C ) -- 27(C ) | 0.31 | 0.10 | -0.42 | -0.32 | -0.86 | 0.21 | Covalent bond |
| 73 | 22(C ) -- 23(C ) | 0.27 | 0.06 | -0.30 | -0.23 | -0.67 | 0.09 | Covalent bond |
| 74 | 22(C ) -- 24(O ) | 0.40 | 0.67 | -1.35 | -0.68 | -0.02 | 0.04 | Covalent bond |
| **75** | **23(C ) -- 4(N )** | **0.01** | **0.01** | **0.00** | **0.00** | **0.03** | **5.91** | **Van der Waal** |
| 76 | 39(H ) -- 14(C ) | 0.29 | 0.03 | -0.32 | -0.29 | -1.01 | 0.01 | Covalent bond |
| 78 | 30(O ) -- 20(C ) | 0.29 | 0.35 | -0.77 | -0.42 | -0.28 | 0.01 | Covalent bond |
| **79** | **26(C ) -- 14(C )** | **0.01** | **0.00** | **0.00** | **0.00** | **0.02** | **0.51** | **Van der Waal** |
| 82 | 13(S ) -- 5(C ) | 0.20 | 0.06 | -0.21 | -0.15 | -0.36 | 0.15 | Covalent bond |
| 83 | 23(C ) -- 29(C ) | 0.31 | 0.10 | -0.41 | -0.31 | -0.84 | 0.19 | Covalent bond |
| 84 | 27(C ) -- 55(Br) | 0.16 | 0.05 | -0.15 | -0.09 | -0.16 | 0.07 | Covalent bond |
| 85 | 4(N ) -- 5(C ) | 0.38 | 0.32 | -0.91 | -0.59 | -1.07 | 0.29 | Covalent bond |
| 86 | 42(H ) -- 19(C ) | 0.28 | 0.04 | -0.32 | -0.28 | -0.97 | 0.02 | Covalent bond |
| 87 | 20(C ) -- 19(C ) | 0.31 | 0.11 | -0.43 | -0.32 | -0.87 | 0.25 | Covalent bond |
| 88 | 4(N ) -- 3(C ) | 0.30 | 0.16 | -0.54 | -0.38 | -0.86 | 0.10 | Covalent bond |
| 90 | 20(C ) -- 15(C ) | 0.30 | 0.09 | -0.38 | -0.29 | -0.80 | 0.22 | Covalent bond |
| 91 | 27(C ) -- 28(C ) | 0.31 | 0.10 | -0.42 | -0.32 | -0.86 | 0.21 | Covalent bond |
| 92 | 14(C ) -- 3(C ) | 0.32 | 0.12 | -0.47 | -0.35 | -0.93 | 0.27 | Covalent bond |
| **93** | **55(Br) -- 15(C )** | **0.01** | **0.00** | **0.00** | **0.00** | **0.02** | **3.93** | **Halogen bond** |
| 94 | 14(C ) -- 15(C ) | 0.28 | 0.08 | -0.35 | -0.27 | -0.75 | 0.11 | Covalent bond |
| 96 | 29(C ) -- 28(C ) | 0.31 | 0.10 | -0.42 | -0.32 | -0.87 | 0.20 | Covalent bond |
| 98 | 5(C ) -- 1(N ) | 0.30 | 0.20 | -0.60 | -0.40 | -0.82 | 0.14 | Covalent bond |
| 100 | 29(C ) -- 48(H ) | 0.28 | 0.03 | -0.32 | -0.28 | -0.99 | 0.01 | Covalent bond |
| 102 | 19(C ) -- 18(C ) | 0.31 | 0.10 | -0.41 | -0.31 | -0.84 | 0.20 | Covalent bond |
| 103 | 3(C ) -- 2(C ) | 0.27 | 0.07 | -0.31 | -0.24 | -0.68 | 0.15 | Covalent bond |
| 105 | 15(C ) -- 16(C ) | 0.30 | 0.10 | -0.40 | -0.30 | -0.83 | 0.20 | Covalent bond |
| 106 | 28(C ) -- 47(H ) | 0.28 | 0.03 | -0.32 | -0.28 | -0.99 | 0.02 | Covalent bond |
| 109 | 1(N ) -- 2(C ) | 0.30 | 0.18 | -0.57 | -0.39 | -0.81 | 0.10 | Covalent bond |
| 110 | 18(C ) -- 41(H ) | 0.28 | 0.04 | -0.32 | -0.28 | -0.98 | 0.02 | Covalent bond |
| 111 | 48(H ) -- 38(H ) | 0.01 | 0.00 | 0.00 | 0.00 | 0.02 | 0.06 | Van der Waal |
| 112 | 18(C ) -- 17(C ) | 0.31 | 0.10 | -0.42 | -0.32 | -0.86 | 0.23 | Covalent bond |
| 113 | 1(N ) -- 6(C ) | 0.28 | 0.19 | -0.56 | -0.36 | -0.69 | 0.03 | Covalent bond |
| 114 | 2(C ) -- 12(O ) | 0.40 | 0.63 | -1.30 | -0.67 | -0.19 | 0.07 | Covalent bond |
| 115 | 16(C ) -- 17(C ) | 0.31 | 0.10 | -0.43 | -0.33 | -0.90 | 0.24 | Covalent bond |
| 116 | 34(H ) -- 7(C ) | 0.28 | 0.04 | -0.31 | -0.28 | -0.97 | 0.02 | Covalent bond |
| 117 | 16(C ) -- 40(H ) | 0.29 | 0.03 | -0.32 | -0.29 | -1.00 | 0.02 | Covalent bond |
| 118 | 6(C ) -- 7(C ) | 0.31 | 0.10 | -0.42 | -0.32 | -0.87 | 0.24 | Covalent bond |
| **119** | **40(H ) -- 12(O )** | **0.03** | **0.02** | **-0.02** | **0.00** | **0.09** | **0.03** | **H-bond** |
| 120 | 6(C ) -- 11(C ) | 0.31 | 0.10 | -0.42 | -0.32 | -0.87 | 0.23 | Covalent bond |
| 121 | 17(C ) -- 32(O ) | 0.29 | 0.33 | -0.74 | -0.41 | -0.32 | 0.02 | Covalent bond |
| 122 | 38(H ) -- 11(C ) | 0.28 | 0.04 | -0.32 | -0.28 | -0.99 | 0.02 | Covalent bond |
| 123 | 7(C ) -- 8(C ) | 0.31 | 0.10 | -0.42 | -0.32 | -0.86 | 0.20 | Covalent bond |
| 125 | 11(C ) -- 10(C ) | 0.31 | 0.10 | -0.42 | -0.32 | -0.86 | 0.20 | Covalent bond |
| 126 | 8(C ) -- 35(H ) | 0.28 | 0.04 | -0.32 | -0.28 | -0.98 | 0.02 | Covalent bond |
| 127 | 8(C ) -- 9(C ) | 0.31 | 0.10 | -0.42 | -0.32 | -0.86 | 0.20 | Covalent bond |
| 128 | 53(H ) -- 33(C ) | 0.28 | 0.04 | -0.31 | -0.27 | -0.95 | 0.04 | Covalent bond |
| 129 | 32(O ) -- 33(C ) | 0.25 | 0.27 | -0.60 | -0.33 | -0.26 | 0.00 | Covalent bond |
| 130 | 10(C ) -- 9(C ) | 0.31 | 0.10 | -0.42 | -0.32 | -0.86 | 0.19 | Covalent bond |
| 131 | 54(H ) -- 33(C ) | 0.28 | 0.04 | -0.31 | -0.27 | -0.95 | 0.04 | Covalent bond |
| 132 | 10(C ) -- 37(H ) | 0.28 | 0.04 | -0.32 | -0.28 | -0.98 | 0.02 | Covalent bond |
| 133 | 9(C ) -- 36(H ) | 0.28 | 0.04 | -0.32 | -0.28 | -0.98 | 0.02 | Covalent bond |
| 134 | 33(C ) -- 52(H ) | 0.28 | 0.04 | -0.32 | -0.28 | -0.99 | 0.04 | Covalent bond |

**Table S55:** Topological parameters and energy density descriptors for compound **2e** (Gas phase) at bond critical points (BCP), including atomic connectivity, electron density ρ(r) (a.u.), Lagrangian kinetic energy density G(r) (a.u.), potential energy density V(r) (a.u.), total energy density E(r) or H(r) (a.u.), Laplacian of electron density ∇²ρ(r) (a.u.), ellipticity ε (dimensionless), and bond type.

| CP | Connected atoms | ρ(r) | G(r) | V(r) | H(r) | ∇²ρ(r) | ε | Bond Type |
| --- | --- | --- | --- | --- | --- | --- | --- | --- |
| 56 | 34(O ) -- 32(N ) | 0.51 | 0.39 | -1.05 | -0.67 | -1.13 | 0.11 | Covalent bond |
| 57 | 33(O ) -- 32(N ) | 0.51 | 0.39 | -1.05 | -0.67 | -1.12 | 0.11 | Covalent bond |
| 58 | 52(H ) -- 29(C ) | 0.28 | 0.04 | -0.31 | -0.27 | -0.94 | 0.04 | Covalent bond |
| 59 | 50(H ) -- 29(C ) | 0.28 | 0.04 | -0.32 | -0.28 | -0.99 | 0.04 | Covalent bond |
| 60 | 32(N ) -- 25(C ) | 0.26 | 0.14 | -0.45 | -0.31 | -0.68 | 0.12 | Covalent bond |
| 61 | 29(C ) -- 51(H ) | 0.28 | 0.04 | -0.31 | -0.27 | -0.94 | 0.04 | Covalent bond |
| 62 | 47(H ) -- 24(C ) | 0.28 | 0.03 | -0.32 | -0.28 | -1.00 | 0.01 | Covalent bond |
| 63 | 29(C ) -- 28(O ) | 0.25 | 0.27 | -0.61 | -0.34 | -0.26 | 0.01 | Covalent bond |
| 64 | 25(C ) -- 24(C ) | 0.31 | 0.10 | -0.43 | -0.33 | -0.88 | 0.21 | Covalent bond |
| 65 | 43(H ) -- 19(C ) | 0.28 | 0.04 | -0.32 | -0.28 | -0.96 | 0.03 | Covalent bond |
| 66 | 25(C ) -- 26(C ) | 0.31 | 0.10 | -0.43 | -0.32 | -0.88 | 0.21 | Covalent bond |
| 67 | 24(C ) -- 23(C ) | 0.31 | 0.10 | -0.43 | -0.32 | -0.87 | 0.19 | Covalent bond |
| 68 | 48(H ) -- 26(C ) | 0.28 | 0.03 | -0.32 | -0.28 | -0.99 | 0.01 | Covalent bond |
| 69 | 28(O ) -- 20(C ) | 0.28 | 0.34 | -0.75 | -0.41 | -0.27 | 0.01 | Covalent bond |
| 71 | 19(C ) -- 20(C ) | 0.31 | 0.11 | -0.43 | -0.33 | -0.87 | 0.27 | Covalent bond |
| 72 | 23(C ) -- 46(H ) | 0.28 | 0.03 | -0.32 | -0.28 | -1.00 | 0.02 | Covalent bond |
| 73 | 19(C ) -- 18(C ) | 0.30 | 0.10 | -0.41 | -0.31 | -0.83 | 0.21 | Covalent bond |
| **74** | **28(O ) -- 40(H )** | **0.02** | **0.02** | **-0.01** | **0.00** | **0.08** | **0.86** | **H-bond** |
| 76 | 26(C ) -- 27(C ) | 0.31 | 0.10 | -0.43 | -0.32 | -0.88 | 0.21 | Covalent bond |
| 77 | 42(H ) -- 18(C ) | 0.28 | 0.04 | -0.32 | -0.28 | -0.96 | 0.02 | Covalent bond |
| 78 | 23(C ) -- 22(C ) | 0.31 | 0.10 | -0.41 | -0.31 | -0.86 | 0.20 | Covalent bond |
| 79 | 20(C ) -- 15(C ) | 0.30 | 0.09 | -0.38 | -0.29 | -0.80 | 0.22 | Covalent bond |
| 81 | 27(C ) -- 22(C ) | 0.31 | 0.10 | -0.41 | -0.31 | -0.85 | 0.20 | Covalent bond |
| 82 | 40(H ) -- 14(C ) | 0.29 | 0.04 | -0.32 | -0.28 | -1.00 | 0.01 | Covalent bond |
| **83** | **46(H ) -- 4(N )** | **0.01** | **0.01** | **-0.01** | **0.00** | **0.04** | **0.16** | **H-bond** |
| 84 | 18(C ) -- 17(C ) | 0.31 | 0.10 | -0.42 | -0.32 | -0.86 | 0.25 | Covalent bond |
| 85 | 15(C ) -- 14(C ) | 0.28 | 0.08 | -0.34 | -0.26 | -0.73 | 0.11 | Covalent bond |
| 86 | 27(C ) -- 49(H ) | 0.28 | 0.04 | -0.32 | -0.28 | -0.96 | 0.02 | Covalent bond |
| 88 | 15(C ) -- 16(C ) | 0.30 | 0.10 | -0.41 | -0.31 | -0.83 | 0.20 | Covalent bond |
| 89 | 22(C ) -- 21(C ) | 0.26 | 0.06 | -0.28 | -0.22 | -0.63 | 0.03 | Covalent bond |
| 90 | 54(H ) -- 31(C ) | 0.28 | 0.04 | -0.31 | -0.27 | -0.94 | 0.04 | Covalent bond |
| 91 | 17(C ) -- 16(C ) | 0.31 | 0.10 | -0.43 | -0.33 | -0.89 | 0.23 | Covalent bond |
| 92 | 14(C ) -- 3(C ) | 0.33 | 0.12 | -0.47 | -0.35 | -0.94 | 0.29 | Covalent bond |
| 93 | 17(C ) -- 30(O ) | 0.29 | 0.33 | -0.74 | -0.41 | -0.30 | 0.01 | Covalent bond |
| 94 | 45(H ) -- 21(C ) | 0.28 | 0.04 | -0.31 | -0.28 | -0.96 | 0.01 | Covalent bond |
| 95 | 31(C ) -- 55(H ) | 0.28 | 0.04 | -0.31 | -0.27 | -0.94 | 0.04 | Covalent bond |
| 96 | 4(N ) -- 3(C ) | 0.30 | 0.16 | -0.54 | -0.38 | -0.86 | 0.09 | Covalent bond |
| 97 | 31(C ) -- 30(O ) | 0.25 | 0.27 | -0.62 | -0.35 | -0.30 | 0.01 | Covalent bond |
| 98 | 21(C ) -- 44(H ) | 0.28 | 0.04 | -0.31 | -0.27 | -0.94 | 0.01 | Covalent bond |
| 99 | 16(C ) -- 41(H ) | 0.29 | 0.03 | -0.32 | -0.29 | -1.01 | 0.02 | Covalent bond |
| 101 | 31(C ) -- 53(H ) | 0.28 | 0.04 | -0.32 | -0.28 | -0.99 | 0.04 | Covalent bond |
| 102 | 21(C ) -- 13(S ) | 0.17 | 0.05 | -0.16 | -0.11 | -0.24 | 0.06 | Covalent bond |
| 103 | 4(N ) -- 5(C ) | 0.38 | 0.30 | -0.88 | -0.58 | -1.11 | 0.26 | Covalent bond |
| 104 | 3(C ) -- 2(C ) | 0.27 | 0.07 | -0.31 | -0.24 | -0.67 | 0.15 | Covalent bond |
| **106** | **41(H ) -- 12(O )** | **0.02** | **0.02** | **-0.02** | **0.00** | **0.09** | **0.02** | **H-bond** |
| 107 | 5(C ) -- 13(S ) | 0.20 | 0.06 | -0.22 | -0.16 | -0.39 | 0.26 | Covalent bond |
| 108 | 5(C ) -- 1(N ) | 0.30 | 0.19 | -0.59 | -0.40 | -0.84 | 0.12 | Covalent bond |
| 109 | 2(C ) -- 12(O ) | 0.41 | 0.65 | -1.34 | -0.69 | -0.15 | 0.08 | Covalent bond |
| 110 | 2(C ) -- 1(N ) | 0.29 | 0.18 | -0.55 | -0.37 | -0.79 | 0.10 | Covalent bond |
| 111 | 35(H ) -- 7(C ) | 0.28 | 0.04 | -0.32 | -0.28 | -0.97 | 0.02 | Covalent bond |
| 112 | 1(N ) -- 6(C ) | 0.28 | 0.19 | -0.55 | -0.36 | -0.70 | 0.03 | Covalent bond |
| 113 | 7(C ) -- 6(C ) | 0.31 | 0.10 | -0.42 | -0.32 | -0.87 | 0.24 | Covalent bond |
| 114 | 7(C ) -- 8(C ) | 0.31 | 0.10 | -0.42 | -0.32 | -0.87 | 0.20 | Covalent bond |
| 115 | 6(C ) -- 11(C ) | 0.31 | 0.10 | -0.42 | -0.32 | -0.88 | 0.24 | Covalent bond |
| 116 | 36(H ) -- 8(C ) | 0.28 | 0.04 | -0.32 | -0.28 | -0.97 | 0.02 | Covalent bond |
| 118 | 11(C ) -- 39(H ) | 0.28 | 0.04 | -0.32 | -0.28 | -0.98 | 0.01 | Covalent bond |
| 119 | 8(C ) -- 9(C ) | 0.31 | 0.10 | -0.42 | -0.32 | -0.87 | 0.20 | Covalent bond |
| 120 | 11(C ) -- 10(C ) | 0.31 | 0.10 | -0.42 | -0.32 | -0.87 | 0.20 | Covalent bond |
| 121 | 9(C ) -- 10(C ) | 0.31 | 0.10 | -0.42 | -0.32 | -0.87 | 0.20 | Covalent bond |
| 122 | 9(C ) -- 37(H ) | 0.28 | 0.04 | -0.32 | -0.28 | -0.97 | 0.02 | Covalent bond |
| 123 | 10(C ) -- 38(H ) | 0.28 | 0.04 | -0.32 | -0.28 | -0.97 | 0.02 | Covalent bond |

**Table S56:** Topological parameters and energy density descriptors for compound **2e** (DMSO phase) at bond critical points (BCP), including atomic connectivity, electron density ρ(r) (a.u.), Lagrangian kinetic energy density G(r) (a.u.), potential energy density V(r) (a.u.), total energy density E(r) or H(r) (a.u.), Laplacian of electron density ∇²ρ(r) (a.u.), ellipticity ε (dimensionless), and bond type.

| CP | Connected atoms | ρ(r) | G(r) | V(r) | H(r) | ∇²ρ(r) | ε | Bond Type |
| --- | --- | --- | --- | --- | --- | --- | --- | --- |
| 56 | 34(O ) -- 32(N ) | 0.50 | 0.38 | -1.04 | -0.66 | -1.11 | 0.11 | Covalent bond |
| 57 | 33(O ) -- 32(N ) | 0.50 | 0.38 | -1.04 | -0.66 | -1.11 | 0.11 | Covalent bond |
| 58 | 32(N ) -- 25(C ) | 0.26 | 0.16 | -0.48 | -0.32 | -0.66 | 0.13 | Covalent bond |
| 59 | 47(H ) -- 24(C ) | 0.28 | 0.03 | -0.32 | -0.28 | -1.00 | 0.01 | Covalent bond |
| 60 | 25(C ) -- 24(C ) | 0.31 | 0.10 | -0.43 | -0.32 | -0.88 | 0.21 | Covalent bond |
| 61 | 25(C ) -- 26(C ) | 0.31 | 0.10 | -0.42 | -0.32 | -0.87 | 0.21 | Covalent bond |
| 62 | 52(H ) -- 29(C ) | 0.28 | 0.04 | -0.31 | -0.28 | -0.96 | 0.04 | Covalent bond |
| 63 | 50(H ) -- 29(C ) | 0.28 | 0.04 | -0.32 | -0.28 | -0.99 | 0.04 | Covalent bond |
| 64 | 48(H ) -- 26(C ) | 0.28 | 0.03 | -0.32 | -0.28 | -1.00 | 0.01 | Covalent bond |
| 65 | 24(C ) -- 23(C ) | 0.31 | 0.10 | -0.43 | -0.32 | -0.87 | 0.20 | Covalent bond |
| 67 | 29(C ) -- 28(O ) | 0.24 | 0.27 | -0.59 | -0.32 | -0.22 | 0.00 | Covalent bond |
| 68 | 29(C ) -- 51(H ) | 0.28 | 0.04 | -0.31 | -0.28 | -0.96 | 0.04 | Covalent bond |
| 69 | 26(C ) -- 27(C ) | 0.31 | 0.11 | -0.43 | -0.33 | -0.88 | 0.20 | Covalent bond |
| 70 | 23(C ) -- 46(H ) | 0.28 | 0.03 | -0.32 | -0.28 | -1.00 | 0.02 | Covalent bond |
| 71 | 23(C ) -- 22(C ) | 0.31 | 0.10 | -0.41 | -0.31 | -0.86 | 0.19 | Covalent bond |
| 72 | 43(H ) -- 19(C ) | 0.28 | 0.04 | -0.32 | -0.28 | -0.97 | 0.02 | Covalent bond |
| 73 | 27(C ) -- 22(C ) | 0.31 | 0.10 | -0.41 | -0.31 | -0.85 | 0.19 | Covalent bond |
| 74 | 28(O ) -- 20(C ) | 0.29 | 0.35 | -0.76 | -0.42 | -0.29 | 0.01 | Covalent bond |
| 75 | 27(C ) -- 49(H ) | 0.28 | 0.04 | -0.32 | -0.28 | -0.98 | 0.02 | Covalent bond |
| 76 | 19(C ) -- 20(C ) | 0.31 | 0.11 | -0.43 | -0.32 | -0.86 | 0.25 | Covalent bond |
| **77** | **28(O ) -- 40(H )** | **0.02** | **0.02** | **-0.01** | **0.00** | **0.08** | **1.00** | **H-bond** |
| **79** | **23(C ) -- 4(N )** | **0.01** | **0.01** | **0.00** | **0.00** | **0.03** | **1.42** | **Van der Waal** |
| 80 | 19(C ) -- 18(C ) | 0.30 | 0.10 | -0.41 | -0.31 | -0.83 | 0.21 | Covalent bond |
| 81 | 22(C ) -- 21(C ) | 0.26 | 0.06 | -0.28 | -0.22 | -0.63 | 0.03 | Covalent bond |
| 83 | 20(C ) -- 15(C ) | 0.29 | 0.09 | -0.38 | -0.29 | -0.80 | 0.23 | Covalent bond |
| 84 | 40(H ) -- 14(C ) | 0.28 | 0.04 | -0.32 | -0.28 | -1.00 | 0.01 | Covalent bond |
| 85 | 42(H ) -- 18(C ) | 0.28 | 0.04 | -0.32 | -0.28 | -0.98 | 0.02 | Covalent bond |
| 87 | 21(C ) -- 45(H ) | 0.28 | 0.04 | -0.31 | -0.28 | -0.96 | 0.01 | Covalent bond |
| 88 | 15(C ) -- 14(C ) | 0.28 | 0.08 | -0.34 | -0.26 | -0.73 | 0.11 | Covalent bond |
| 89 | 21(C ) -- 44(H ) | 0.28 | 0.04 | -0.31 | -0.27 | -0.94 | 0.01 | Covalent bond |
| 90 | 18(C ) -- 17(C ) | 0.31 | 0.10 | -0.42 | -0.32 | -0.86 | 0.24 | Covalent bond |
| 91 | 15(C ) -- 16(C ) | 0.30 | 0.10 | -0.40 | -0.30 | -0.82 | 0.20 | Covalent bond |
| 92 | 14(C ) -- 3(C ) | 0.32 | 0.12 | -0.47 | -0.35 | -0.93 | 0.28 | Covalent bond |
| 93 | 21(C ) -- 13(S ) | 0.17 | 0.05 | -0.16 | -0.11 | -0.24 | 0.05 | Covalent bond |
| 94 | 54(H ) -- 31(C ) | 0.28 | 0.04 | -0.31 | -0.27 | -0.95 | 0.04 | Covalent bond |
| 95 | 17(C ) -- 16(C ) | 0.31 | 0.10 | -0.43 | -0.33 | -0.89 | 0.24 | Covalent bond |
| 96 | 4(N ) -- 3(C ) | 0.30 | 0.16 | -0.53 | -0.37 | -0.86 | 0.09 | Covalent bond |
| 97 | 17(C ) -- 30(O ) | 0.29 | 0.33 | -0.74 | -0.41 | -0.32 | 0.02 | Covalent bond |
| 98 | 4(N ) -- 5(C ) | 0.38 | 0.30 | -0.88 | -0.58 | -1.11 | 0.26 | Covalent bond |
| 100 | 16(C ) -- 41(H ) | 0.29 | 0.03 | -0.32 | -0.29 | -1.00 | 0.02 | Covalent bond |
| 101 | 3(C ) -- 2(C ) | 0.27 | 0.07 | -0.30 | -0.23 | -0.66 | 0.15 | Covalent bond |
| 102 | 31(C ) -- 30(O ) | 0.25 | 0.27 | -0.60 | -0.33 | -0.26 | 0.00 | Covalent bond |
| 104 | 5(C ) -- 13(S ) | 0.20 | 0.06 | -0.23 | -0.16 | -0.40 | 0.25 | Covalent bond |
| 105 | 31(C ) -- 55(H ) | 0.28 | 0.04 | -0.31 | -0.27 | -0.95 | 0.04 | Covalent bond |
| 106 | 5(C ) -- 1(N ) | 0.30 | 0.19 | -0.60 | -0.40 | -0.84 | 0.12 | Covalent bond |
| **107** | **41(H ) -- 12(O )** | **0.02** | **0.02** | **-0.02** | **0.00** | **0.09** | **0.03** | **H-bond** |
| 108 | 31(C ) -- 53(H ) | 0.28 | 0.04 | -0.32 | -0.28 | -0.99 | 0.04 | Covalent bond |
| 109 | 2(C ) -- 1(N ) | 0.30 | 0.19 | -0.58 | -0.39 | -0.82 | 0.11 | Covalent bond |
| 110 | 2(C ) -- 12(O ) | 0.40 | 0.63 | -1.31 | -0.68 | -0.19 | 0.07 | Covalent bond |
| 111 | 35(H ) -- 7(C ) | 0.28 | 0.03 | -0.32 | -0.28 | -0.98 | 0.02 | Covalent bond |
| 112 | 1(N ) -- 6(C ) | 0.27 | 0.19 | -0.54 | -0.36 | -0.69 | 0.03 | Covalent bond |
| 113 | 7(C ) -- 6(C ) | 0.31 | 0.10 | -0.42 | -0.32 | -0.87 | 0.24 | Covalent bond |
| 114 | 7(C ) -- 8(C ) | 0.31 | 0.10 | -0.42 | -0.32 | -0.87 | 0.20 | Covalent bond |
| 115 | 36(H ) -- 8(C ) | 0.28 | 0.04 | -0.32 | -0.28 | -0.98 | 0.02 | Covalent bond |
| 116 | 6(C ) -- 11(C ) | 0.31 | 0.10 | -0.42 | -0.32 | -0.87 | 0.24 | Covalent bond |
| 118 | 8(C ) -- 9(C ) | 0.31 | 0.10 | -0.42 | -0.32 | -0.86 | 0.19 | Covalent bond |
| 119 | 11(C ) -- 39(H ) | 0.28 | 0.04 | -0.31 | -0.28 | -0.98 | 0.02 | Covalent bond |
| 120 | 11(C ) -- 10(C ) | 0.31 | 0.10 | -0.42 | -0.32 | -0.86 | 0.20 | Covalent bond |
| 121 | 9(C ) -- 10(C ) | 0.31 | 0.10 | -0.42 | -0.32 | -0.86 | 0.19 | Covalent bond |
| 122 | 9(C ) -- 37(H ) | 0.28 | 0.04 | -0.32 | -0.28 | -0.98 | 0.01 | Covalent bond |
| 123 | 10(C ) -- 38(H ) | 0.28 | 0.04 | -0.32 | -0.28 | -0.98 | 0.02 | Covalent bond |

**Table S57:** Topological parameters and energy density descriptors for compound **2f** (Gas phase) at bond critical points (BCP), including atomic connectivity, electron density ρ(r) (a.u.), Lagrangian kinetic energy density G(r) (a.u.), potential energy density V(r) (a.u.), total energy density E(r) or H(r) (a.u.), Laplacian of electron density ∇²ρ(r) (a.u.), ellipticity ε (dimensionless), and bond type.

| CP | Connected atoms | ρ(r) | G(r) | V(r) | H(r) | ∇²ρ(r) | ε | Bond Type |
| --- | --- | --- | --- | --- | --- | --- | --- | --- |
| 47 | 38(H ) -- 21(C ) | 0.27 | 0.04 | -0.31 | -0.27 | -0.92 | 0.01 | Covalent bond |
| 48 | 21(C ) -- 26(C ) | 0.25 | 0.06 | -0.26 | -0.21 | -0.60 | 0.07 | Covalent bond |
| 49 | 21(C ) -- 37(H ) | 0.28 | 0.04 | -0.31 | -0.28 | -0.96 | 0.01 | Covalent bond |
| 50 | 45(O ) -- 26(C ) | 0.31 | 0.35 | -0.81 | -0.46 | -0.44 | 0.02 | Covalent bond |
| 51 | 21(C ) -- 13(S ) | 0.18 | 0.05 | -0.17 | -0.12 | -0.28 | 0.07 | Covalent bond |
| 52 | 26(C ) -- 27(O ) | 0.42 | 0.69 | -1.42 | -0.72 | -0.10 | 0.08 | Covalent bond |
| 53 | 45(O ) -- 46(H ) | 0.34 | 0.06 | -0.74 | -0.67 | -2.45 | 0.01 | Covalent bond |
| 54 | 42(H ) -- 25(C ) | 0.28 | 0.04 | -0.32 | -0.28 | -0.99 | 0.04 | Covalent bond |
| 55 | 46(H ) -- 24(O ) | 0.03 | 0.03 | -0.02 | 0.00 | 0.11 | 0.07 | Covalent bond |
| 56 | 44(H ) -- 25(C ) | 0.28 | 0.04 | -0.31 | -0.27 | -0.95 | 0.04 | Covalent bond |
| 58 | 25(C ) -- 24(O ) | 0.25 | 0.27 | -0.60 | -0.34 | -0.26 | 0.01 | Covalent bond |
| **59** | **26(C ) -- 4(N )** | **0.01** | **0.01** | **-0.01** | **0.00** | **0.06** | **5.81** | **Van der Waal** |
| 60 | 13(S ) -- 5(C ) | 0.20 | 0.06 | -0.23 | -0.16 | -0.40 | 0.27 | Covalent bond |
| 63 | 25(C ) -- 43(H ) | 0.28 | 0.04 | -0.31 | -0.27 | -0.95 | 0.04 | Covalent bond |
| **64** | **27(O ) -- 34(H )** | **0.01** | **0.01** | **0.00** | **0.00** | **0.03** | **1.22** | **H-bond** |
| 65 | 31(H ) -- 10(C ) | 0.28 | 0.04 | -0.32 | -0.28 | -0.97 | 0.02 | Covalent bond |
| 66 | 32(H ) -- 11(C ) | 0.28 | 0.04 | -0.32 | -0.28 | -0.97 | 0.02 | Covalent bond |
| 67 | 10(C ) -- 11(C ) | 0.31 | 0.10 | -0.42 | -0.32 | -0.86 | 0.20 | Covalent bond |
| 68 | 5(C ) -- 4(N ) | 0.38 | 0.29 | -0.87 | -0.58 | -1.13 | 0.24 | Covalent bond |
| 69 | 24(O ) -- 17(C ) | 0.27 | 0.32 | -0.70 | -0.38 | -0.27 | 0.01 | Covalent bond |
| 70 | 5(C ) -- 1(N ) | 0.30 | 0.19 | -0.58 | -0.40 | -0.85 | 0.11 | Covalent bond |
| 71 | 10(C ) -- 9(C ) | 0.31 | 0.10 | -0.42 | -0.32 | -0.87 | 0.20 | Covalent bond |
| **72** | **34(H ) -- 4(N )** | **0.02** | **0.01** | **-0.01** | **0.00** | **0.06** | **0.16** | **H-bond** |
| 73 | 11(C ) -- 6(C ) | 0.31 | 0.10 | -0.42 | -0.32 | -0.86 | 0.24 | Covalent bond |
| 74 | 34(H ) -- 16(C ) | 0.29 | 0.04 | -0.32 | -0.29 | -1.01 | 0.02 | Covalent bond |
| 75 | 30(H ) -- 9(C ) | 0.28 | 0.04 | -0.32 | -0.28 | -0.97 | 0.02 | Covalent bond |
| 76 | 17(C ) -- 16(C ) | 0.31 | 0.10 | -0.44 | -0.33 | -0.90 | 0.24 | Covalent bond |
| 78 | 6(C ) -- 1(N ) | 0.28 | 0.19 | -0.56 | -0.37 | -0.70 | 0.04 | Covalent bond |
| 79 | 17(C ) -- 18(C ) | 0.31 | 0.11 | -0.43 | -0.32 | -0.87 | 0.26 | Covalent bond |
| 80 | 9(C ) -- 8(C ) | 0.31 | 0.10 | -0.42 | -0.32 | -0.86 | 0.20 | Covalent bond |
| 83 | 35(H ) -- 18(C ) | 0.28 | 0.04 | -0.32 | -0.28 | -0.96 | 0.02 | Covalent bond |
| 84 | 6(C ) -- 7(C ) | 0.31 | 0.10 | -0.42 | -0.32 | -0.87 | 0.23 | Covalent bond |
| 85 | 4(N ) -- 3(C ) | 0.31 | 0.17 | -0.56 | -0.39 | -0.90 | 0.07 | Covalent bond |
| 86 | 8(C ) -- 7(C ) | 0.31 | 0.10 | -0.42 | -0.32 | -0.87 | 0.20 | Covalent bond |
| 87 | 16(C ) -- 15(C ) | 0.31 | 0.10 | -0.41 | -0.31 | -0.85 | 0.21 | Covalent bond |
| 88 | 8(C ) -- 29(H ) | 0.28 | 0.04 | -0.32 | -0.28 | -0.97 | 0.02 | Covalent bond |
| 89 | 1(N ) -- 2(C ) | 0.28 | 0.17 | -0.52 | -0.36 | -0.76 | 0.09 | Covalent bond |
| 91 | 7(C ) -- 28(H ) | 0.28 | 0.04 | -0.32 | -0.28 | -0.98 | 0.02 | Covalent bond |
| 92 | 3(C ) -- 2(C ) | 0.27 | 0.07 | -0.31 | -0.24 | -0.68 | 0.14 | Covalent bond |
| 93 | 18(C ) -- 19(C ) | 0.30 | 0.10 | -0.41 | -0.31 | -0.82 | 0.21 | Covalent bond |
| 94 | 3(C ) -- 14(C ) | 0.33 | 0.12 | -0.49 | -0.37 | -0.97 | 0.30 | Covalent bond |
| 95 | 15(C ) -- 14(C ) | 0.28 | 0.07 | -0.33 | -0.25 | -0.72 | 0.10 | Covalent bond |
| 96 | 2(C ) -- 12(O ) | 0.41 | 0.67 | -1.37 | -0.70 | -0.12 | 0.07 | Covalent bond |
| 97 | 15(C ) -- 20(C ) | 0.30 | 0.09 | -0.39 | -0.30 | -0.83 | 0.23 | Covalent bond |
| 98 | 19(C ) -- 20(C ) | 0.31 | 0.11 | -0.43 | -0.32 | -0.86 | 0.26 | Covalent bond |
| 99 | 19(C ) -- 36(H ) | 0.28 | 0.04 | -0.32 | -0.28 | -0.96 | 0.03 | Covalent bond |
| 100 | 14(C ) -- 33(H ) | 0.28 | 0.03 | -0.32 | -0.28 | -0.99 | 0.01 | Covalent bond |
| 101 | 20(C ) -- 22(O ) | 0.29 | 0.34 | -0.76 | -0.41 | -0.28 | 0.02 | Covalent bond |
| 102 | 22(O ) -- 23(C ) | 0.25 | 0.27 | -0.61 | -0.34 | -0.27 | 0.01 | Covalent bond |
| 103 | 40(H ) -- 23(C ) | 0.28 | 0.04 | -0.31 | -0.27 | -0.94 | 0.04 | Covalent bond |
| 104 | 23(C ) -- 41(H ) | 0.28 | 0.04 | -0.31 | -0.27 | -0.94 | 0.04 | Covalent bond |
| 105 | 23(C ) -- 39(H ) | 0.28 | 0.04 | -0.32 | -0.28 | -0.99 | 0.04 | Covalent bond |
| **106** | **45(O ) -- 34(H )** | **0.01** | **0.01** | **0.00** | **0.00** | **0.03** | **1.24** | **H-bond** |

**Table S58:** Topological parameters and energy density descriptors for compound **2f** (DMSO phase) at bond critical points (BCP), including atomic connectivity, electron density ρ(r) (a.u.), Lagrangian kinetic energy density G(r) (a.u.), potential energy density V(r) (a.u.), total energy density E(r) or H(r) (a.u.), Laplacian of electron density ∇²ρ(r) (a.u.), ellipticity ε (dimensionless), and bond type.

| CP | Connected atoms | ρ(r) | G(r) | V(r) | H(r) | ∇²ρ(r) | ε | Bond Type |
| --- | --- | --- | --- | --- | --- | --- | --- | --- |
| 47 | 42(H ) -- 25(C ) | 0.28 | 0.03 | -0.32 | -0.28 | -0.99 | 0.04 | Covalent bond |
| 48 | 25(C ) -- 43(H ) | 0.28 | 0.04 | -0.31 | -0.28 | -0.96 | 0.04 | Covalent bond |
| 49 | 25(C ) -- 44(H ) | 0.28 | 0.04 | -0.31 | -0.28 | -0.96 | 0.04 | Covalent bond |
| 50 | 25(C ) -- 24(O ) | 0.24 | 0.27 | -0.58 | -0.32 | -0.21 | 0.00 | Covalent bond |
| 51 | 46(H ) -- 45(O ) | 0.34 | 0.06 | -0.73 | -0.66 | -2.41 | 0.01 | Covalent bond |
| 52 | 38(H ) -- 21(C ) | 0.27 | 0.04 | -0.31 | -0.27 | -0.93 | 0.01 | Covalent bond |
| 53 | 27(O ) -- 26(C ) | 0.42 | 0.68 | -1.38 | -0.71 | -0.13 | 0.07 | Covalent bond |
| 54 | 46(H ) -- 24(O ) | 0.03 | 0.03 | -0.03 | 0.00 | 0.11 | 0.06 | Covalent bond |
| 55 | 26(C ) -- 45(O ) | 0.31 | 0.36 | -0.82 | -0.46 | -0.43 | 0.03 | Covalent bond |
| 56 | 26(C ) -- 21(C ) | 0.25 | 0.06 | -0.27 | -0.21 | -0.61 | 0.07 | Covalent bond |
| 57 | 21(C ) -- 13(S ) | 0.18 | 0.05 | -0.17 | -0.12 | -0.28 | 0.07 | Covalent bond |
| 58 | 21(C ) -- 37(H ) | 0.28 | 0.04 | -0.31 | -0.28 | -0.96 | 0.02 | Covalent bond |
| **60** | **27(O ) -- 34(H )** | **0.01** | **0.01** | **0.00** | **0.00** | **0.03** | **1.48** | **H-bond** |
| 62 | 24(O ) -- 17(C ) | 0.28 | 0.32 | -0.70 | -0.39 | -0.29 | 0.01 | Covalent bond |
| **64** | **26(C ) -- 4(N )** | **0.01** | **0.01** | **-0.01** | **0.00** | **0.05** | **3.30** | **Van der Waal** |
| 66 | 34(H ) -- 16(C ) | 0.29 | 0.04 | -0.32 | -0.29 | -1.00 | 0.02 | Covalent bond |
| 67 | 17(C ) -- 16(C ) | 0.32 | 0.10 | -0.44 | -0.33 | -0.90 | 0.25 | Covalent bond |
| 68 | 17(C ) -- 18(C ) | 0.31 | 0.10 | -0.42 | -0.32 | -0.86 | 0.24 | Covalent bond |
| 69 | 13(S ) -- 5(C ) | 0.20 | 0.06 | -0.23 | -0.17 | -0.41 | 0.27 | Covalent bond |
| 70 | 35(H ) -- 18(C ) | 0.28 | 0.04 | -0.32 | -0.28 | -0.98 | 0.02 | Covalent bond |
| **71** | **34(H ) -- 4(N )** | **0.02** | **0.01** | **-0.01** | **0.00** | **0.06** | **0.18** | **Van der Waal** |
| 72 | 32(H ) -- 11(C ) | 0.28 | 0.04 | -0.31 | -0.28 | -0.98 | 0.02 | Covalent bond |
| 73 | 31(H ) -- 10(C ) | 0.28 | 0.04 | -0.32 | -0.28 | -0.98 | 0.02 | Covalent bond |
| 74 | 5(C ) -- 4(N ) | 0.38 | 0.29 | -0.87 | -0.57 | -1.13 | 0.24 | Covalent bond |
| 76 | 16(C ) -- 15(C ) | 0.30 | 0.10 | -0.41 | -0.31 | -0.83 | 0.22 | Covalent bond |
| 78 | 18(C ) -- 19(C ) | 0.30 | 0.10 | -0.41 | -0.31 | -0.82 | 0.21 | Covalent bond |
| 79 | 5(C ) -- 1(N ) | 0.30 | 0.19 | -0.59 | -0.40 | -0.86 | 0.11 | Covalent bond |
| 80 | 11(C ) -- 10(C ) | 0.31 | 0.10 | -0.42 | -0.32 | -0.86 | 0.20 | Covalent bond |
| 81 | 11(C ) -- 6(C ) | 0.31 | 0.10 | -0.42 | -0.32 | -0.87 | 0.24 | Covalent bond |
| 82 | 4(N ) -- 3(C ) | 0.31 | 0.17 | -0.56 | -0.39 | -0.89 | 0.08 | Covalent bond |
| 84 | 10(C ) -- 9(C ) | 0.31 | 0.10 | -0.42 | -0.32 | -0.86 | 0.19 | Covalent bond |
| 85 | 1(N ) -- 6(C ) | 0.27 | 0.19 | -0.55 | -0.36 | -0.69 | 0.03 | Covalent bond |
| 87 | 15(C ) -- 14(C ) | 0.28 | 0.07 | -0.33 | -0.25 | -0.72 | 0.10 | Covalent bond |
| 88 | 15(C ) -- 20(C ) | 0.30 | 0.09 | -0.39 | -0.30 | -0.82 | 0.23 | Covalent bond |
| 89 | 19(C ) -- 20(C ) | 0.31 | 0.10 | -0.42 | -0.32 | -0.86 | 0.25 | Covalent bond |
| 90 | 19(C ) -- 36(H ) | 0.28 | 0.04 | -0.32 | -0.28 | -0.97 | 0.02 | Covalent bond |
| 91 | 3(C ) -- 14(C ) | 0.33 | 0.12 | -0.49 | -0.36 | -0.97 | 0.30 | Covalent bond |
| 92 | 9(C ) -- 30(H ) | 0.28 | 0.04 | -0.32 | -0.28 | -0.98 | 0.01 | Covalent bond |
| 93 | 1(N ) -- 2(C ) | 0.29 | 0.18 | -0.55 | -0.38 | -0.79 | 0.09 | Covalent bond |
| 94 | 3(C ) -- 2(C ) | 0.27 | 0.07 | -0.31 | -0.24 | -0.68 | 0.15 | Covalent bond |
| 95 | 6(C ) -- 7(C ) | 0.31 | 0.10 | -0.42 | -0.32 | -0.87 | 0.24 | Covalent bond |
| 96 | 9(C ) -- 8(C ) | 0.31 | 0.10 | -0.42 | -0.32 | -0.86 | 0.19 | Covalent bond |
| 97 | 20(C ) -- 22(O ) | 0.29 | 0.35 | -0.77 | -0.42 | -0.29 | 0.02 | Covalent bond |
| 98 | 7(C ) -- 8(C ) | 0.31 | 0.10 | -0.42 | -0.32 | -0.87 | 0.20 | Covalent bond |
| 99 | 2(C ) -- 12(O ) | 0.41 | 0.65 | -1.34 | -0.69 | -0.15 | 0.06 | Covalent bond |
| 100 | 14(C ) -- 33(H ) | 0.28 | 0.03 | -0.32 | -0.28 | -0.99 | 0.01 | Covalent bond |
| 101 | 7(C ) -- 28(H ) | 0.28 | 0.03 | -0.31 | -0.28 | -0.98 | 0.02 | Covalent bond |
| 102 | 8(C ) -- 29(H ) | 0.28 | 0.04 | -0.32 | -0.28 | -0.98 | 0.02 | Covalent bond |
| 103 | 22(O ) -- 23(C ) | 0.24 | 0.27 | -0.59 | -0.32 | -0.22 | 0.00 | Covalent bond |
| 104 | 41(H ) -- 23(C ) | 0.28 | 0.04 | -0.31 | -0.28 | -0.96 | 0.04 | Covalent bond |
| 105 | 40(H ) -- 23(C ) | 0.28 | 0.04 | -0.31 | -0.28 | -0.96 | 0.04 | Covalent bond |
| 106 | 23(C ) -- 39(H ) | 0.28 | 0.04 | -0.32 | -0.28 | -0.99 | 0.04 | Covalent bond |
| **107** | **45(O ) -- 34(H )** | **0.01** | **0.01** | **0.00** | **0.00** | **0.03** | **1.30** | **H-bond** |

**Table S59:** Topological parameters and energy density descriptors for compound **2g** (DMSO phase) at bond critical points (BCP), including atomic connectivity, electron density ρ(r) (a.u.), Lagrangian kinetic energy density G(r) (a.u.), potential energy density V(r) (a.u.), total energy density E(r) or H(r) (a.u.), Laplacian of electron density ∇²ρ(r) (a.u.), ellipticity ε (dimensionless), and bond type.

| CP | Connected atoms | ρ(r) | G(r) | V(r) | H(r) | ∇²ρ(r) | ε | Bond Type |
| --- | --- | --- | --- | --- | --- | --- | --- | --- |
| 53 | 50(H ) -- 49(C ) | 0.27 | 0.04 | -0.31 | -0.27 | -0.90 | 0.01 | Covalent bond |
| 54 | 51(H ) -- 49(C ) | 0.28 | 0.04 | -0.31 | -0.27 | -0.92 | 0.01 | Covalent bond |
| 55 | 52(H ) -- 49(C ) | 0.27 | 0.04 | -0.31 | -0.27 | -0.89 | 0.01 | Covalent bond |
| 56 | 49(C ) -- 46(C ) | 0.25 | 0.06 | -0.26 | -0.21 | -0.59 | 0.04 | Covalent bond |
| 57 | 46(C ) -- 45(O ) | 0.23 | 0.24 | -0.53 | -0.30 | -0.25 | 0.05 | Covalent bond |
| 58 | 46(C ) -- 48(H ) | 0.29 | 0.03 | -0.32 | -0.29 | -1.01 | 0.04 | Covalent bond |
| 59 | 46(C ) -- 47(H ) | 0.28 | 0.04 | -0.32 | -0.28 | -0.98 | 0.03 | Covalent bond |
| 60 | 38(H ) -- 21(C ) | 0.27 | 0.04 | -0.31 | -0.27 | -0.92 | 0.01 | Covalent bond |
| 61 | 27(O ) -- 26(C ) | 0.42 | 0.69 | -1.41 | -0.72 | -0.09 | 0.08 | Covalent bond |
| 62 | 45(O ) -- 26(C ) | 0.31 | 0.35 | -0.81 | -0.46 | -0.43 | 0.01 | Covalent bond |
| 63 | 26(C ) -- 21(C ) | 0.25 | 0.06 | -0.27 | -0.21 | -0.61 | 0.07 | Covalent bond |
| 64 | 42(H ) -- 25(C ) | 0.28 | 0.04 | -0.32 | -0.28 | -0.98 | 0.04 | Covalent bond |
| **65** | **48(H ) -- 24(O )** | **0.01** | **0.01** | **-0.01** | **0.00** | **0.04** | **0.04** | **H-bond** |
| 66 | 43(H ) -- 25(C ) | 0.28 | 0.04 | -0.31 | -0.27 | -0.94 | 0.04 | Covalent bond |
| 67 | 21(C ) -- 37(H ) | 0.28 | 0.04 | -0.31 | -0.28 | -0.96 | 0.01 | Covalent bond |
| 68 | 21(C ) -- 13(S ) | 0.18 | 0.05 | -0.17 | -0.12 | -0.29 | 0.08 | Covalent bond |
| 69 | 25(C ) -- 24(O ) | 0.25 | 0.27 | -0.61 | -0.34 | -0.30 | 0.01 | Covalent bond |
| **71** | **45(O ) -- 34(H )** | **0.01** | **0.01** | **0.00** | **0.00** | **0.02** | **0.09** | **H-bond** |
| 72 | 25(C ) -- 44(H ) | 0.28 | 0.04 | -0.31 | -0.27 | -0.94 | 0.04 | Covalent bond |
| 74 | 13(S ) -- 5(C ) | 0.20 | 0.06 | -0.23 | -0.17 | -0.41 | 0.27 | Covalent bond |
| 75 | 24(O ) -- 17(C ) | 0.28 | 0.33 | -0.73 | -0.40 | -0.30 | 0.02 | Covalent bond |
| 76 | 32(H ) -- 11(C ) | 0.28 | 0.04 | -0.32 | -0.28 | -0.97 | 0.02 | Covalent bond |
| 77 | 34(H ) -- 16(C ) | 0.28 | 0.03 | -0.32 | -0.28 | -1.00 | 0.02 | Covalent bond |
| **78** | **34(H ) -- 4(N )** | **0.02** | **0.01** | **-0.01** | **0.00** | **0.06** | **0.06** | **H-bond** |
| 79 | 31(H ) -- 10(C ) | 0.28 | 0.04 | -0.32 | -0.28 | -0.97 | 0.02 | Covalent bond |
| 80 | 17(C ) -- 16(C ) | 0.31 | 0.10 | -0.43 | -0.33 | -0.89 | 0.24 | Covalent bond |
| 81 | 5(C ) -- 4(N ) | 0.38 | 0.28 | -0.85 | -0.56 | -1.13 | 0.24 | Covalent bond |
| 82 | 17(C ) -- 18(C ) | 0.31 | 0.10 | -0.42 | -0.32 | -0.86 | 0.25 | Covalent bond |
| 83 | 11(C ) -- 10(C ) | 0.31 | 0.10 | -0.42 | -0.32 | -0.86 | 0.20 | Covalent bond |
| 84 | 35(H ) -- 18(C ) | 0.28 | 0.04 | -0.32 | -0.28 | -0.96 | 0.02 | Covalent bond |
| 85 | 5(C ) -- 1(N ) | 0.31 | 0.19 | -0.60 | -0.40 | -0.86 | 0.11 | Covalent bond |
| 87 | 11(C ) -- 6(C ) | 0.31 | 0.10 | -0.42 | -0.32 | -0.86 | 0.24 | Covalent bond |
| 88 | 16(C ) -- 15(C ) | 0.30 | 0.10 | -0.41 | -0.31 | -0.83 | 0.21 | Covalent bond |
| 89 | 4(N ) -- 3(C ) | 0.31 | 0.17 | -0.56 | -0.39 | -0.89 | 0.08 | Covalent bond |
| 90 | 10(C ) -- 9(C ) | 0.31 | 0.10 | -0.42 | -0.32 | -0.87 | 0.20 | Covalent bond |
| 93 | 18(C ) -- 19(C ) | 0.30 | 0.10 | -0.41 | -0.31 | -0.83 | 0.21 | Covalent bond |
| 94 | 1(N ) -- 6(C ) | 0.28 | 0.19 | -0.55 | -0.36 | -0.70 | 0.03 | Covalent bond |
| 96 | 3(C ) -- 14(C ) | 0.33 | 0.12 | -0.49 | -0.37 | -0.97 | 0.29 | Covalent bond |
| 97 | 9(C ) -- 30(H ) | 0.28 | 0.04 | -0.32 | -0.28 | -0.97 | 0.02 | Covalent bond |
| 98 | 15(C ) -- 14(C ) | 0.28 | 0.08 | -0.34 | -0.26 | -0.73 | 0.11 | Covalent bond |
| 99 | 1(N ) -- 2(C ) | 0.29 | 0.17 | -0.53 | -0.36 | -0.76 | 0.08 | Covalent bond |
| 100 | 3(C ) -- 2(C ) | 0.27 | 0.07 | -0.31 | -0.24 | -0.68 | 0.15 | Covalent bond |
| 101 | 15(C ) -- 20(C ) | 0.30 | 0.09 | -0.38 | -0.29 | -0.82 | 0.22 | Covalent bond |
| 102 | 6(C ) -- 7(C ) | 0.31 | 0.10 | -0.42 | -0.32 | -0.87 | 0.23 | Covalent bond |
| 103 | 19(C ) -- 20(C ) | 0.31 | 0.11 | -0.43 | -0.32 | -0.87 | 0.26 | Covalent bond |
| 104 | 9(C ) -- 8(C ) | 0.31 | 0.10 | -0.42 | -0.32 | -0.86 | 0.20 | Covalent bond |
| 105 | 19(C ) -- 36(H ) | 0.28 | 0.04 | -0.32 | -0.28 | -0.96 | 0.03 | Covalent bond |
| 106 | 2(C ) -- 12(O ) | 0.41 | 0.67 | -1.37 | -0.70 | -0.12 | 0.07 | Covalent bond |
| 107 | 7(C ) -- 8(C ) | 0.31 | 0.10 | -0.42 | -0.32 | -0.87 | 0.20 | Covalent bond |
| 108 | 14(C ) -- 33(H ) | 0.28 | 0.04 | -0.32 | -0.28 | -1.00 | 0.01 | Covalent bond |
| 109 | 20(C ) -- 22(O ) | 0.29 | 0.34 | -0.75 | -0.41 | -0.27 | 0.01 | Covalent bond |
| 110 | 7(C ) -- 28(H ) | 0.28 | 0.04 | -0.32 | -0.28 | -0.98 | 0.02 | Covalent bond |
| 111 | 8(C ) -- 29(H ) | 0.28 | 0.04 | -0.32 | -0.28 | -0.97 | 0.02 | Covalent bond |
| 112 | 41(H ) -- 23(C ) | 0.28 | 0.04 | -0.31 | -0.27 | -0.94 | 0.04 | Covalent bond |
| 113 | 22(O ) -- 23(C ) | 0.25 | 0.27 | -0.62 | -0.34 | -0.27 | 0.01 | Covalent bond |
| 114 | 23(C ) -- 40(H ) | 0.28 | 0.04 | -0.31 | -0.27 | -0.94 | 0.04 | Covalent bond |
| 115 | 23(C ) -- 39(H ) | 0.28 | 0.04 | -0.32 | -0.28 | -0.99 | 0.04 | Covalent bond |

**Table S60:** Topological parameters and energy density descriptors for compound **2g** (DMSO phase) at bond critical points (BCP), including atomic connectivity, electron density ρ(r) (a.u.), Lagrangian kinetic energy density G(r) (a.u.), potential energy density V(r) (a.u.), total energy density E(r) or H(r) (a.u.), Laplacian of electron density ∇²ρ(r) (a.u.), ellipticity ε (dimensionless), and bond type.

| CP | Connected atoms | ρ(r) | G(r) | V(r) | H(r) | ∇²ρ(r) | ε | Bond Type |
| --- | --- | --- | --- | --- | --- | --- | --- | --- |
| 53 | 50(H ) -- 49(C ) | 0.27 | 0.04 | -0.31 | -0.27 | -0.91 | 0.01 | Covalent bond |
| 54 | 51(H ) -- 49(C ) | 0.27 | 0.04 | -0.31 | -0.27 | -0.91 | 0.01 | Covalent bond |
| 55 | 52(H ) -- 49(C ) | 0.27 | 0.04 | -0.31 | -0.27 | -0.90 | 0.01 | Covalent bond |
| 56 | 49(C ) -- 46(C ) | 0.25 | 0.06 | -0.27 | -0.21 | -0.60 | 0.04 | Covalent bond |
| 57 | 46(C ) -- 45(O ) | 0.23 | 0.23 | -0.53 | -0.29 | -0.23 | 0.05 | Covalent bond |
| 58 | 46(C ) -- 48(H ) | 0.29 | 0.03 | -0.32 | -0.29 | -1.02 | 0.04 | Covalent bond |
| 59 | 46(C ) -- 47(H ) | 0.28 | 0.04 | -0.32 | -0.28 | -0.99 | 0.04 | Covalent bond |
| 60 | 38(H ) -- 21(C ) | 0.27 | 0.04 | -0.31 | -0.27 | -0.93 | 0.01 | Covalent bond |
| 61 | 27(O ) -- 26(C ) | 0.42 | 0.68 | -1.38 | -0.71 | -0.11 | 0.07 | Covalent bond |
| 62 | 45(O ) -- 26(C ) | 0.31 | 0.36 | -0.82 | -0.46 | -0.42 | 0.01 | Covalent bond |
| 63 | 26(C ) -- 21(C ) | 0.25 | 0.06 | -0.27 | -0.21 | -0.61 | 0.07 | Covalent bond |
| 64 | 42(H ) -- 25(C ) | 0.28 | 0.04 | -0.32 | -0.28 | -0.99 | 0.04 | Covalent bond |
| **65** | **48(H ) -- 24(O )** | **0.01** | **0.01** | **-0.01** | **0.00** | **0.04** | **0.03** | **H-bond** |
| 66 | 21(C ) -- 37(H ) | 0.28 | 0.04 | -0.31 | -0.28 | -0.96 | 0.02 | Covalent bond |
| 67 | 43(H ) -- 25(C ) | 0.28 | 0.04 | -0.31 | -0.27 | -0.95 | 0.04 | Covalent bond |
| 68 | 21(C ) -- 13(S ) | 0.18 | 0.05 | -0.17 | -0.12 | -0.29 | 0.06 | Covalent bond |
| 69 | 25(C ) -- 24(O ) | 0.25 | 0.27 | -0.60 | -0.33 | -0.25 | 0.00 | Covalent bond |
| **71** | **45(O ) -- 34(H )** | **0.01** | **0.00** | **0.00** | **0.00** | **0.02** | **0.12** | **H-bond** |
| 72 | 25(C ) -- 44(H ) | 0.28 | 0.04 | -0.31 | -0.27 | -0.95 | 0.04 | Covalent bond |
| 74 | 13(S ) -- 5(C ) | 0.21 | 0.07 | -0.23 | -0.17 | -0.41 | 0.27 | Covalent bond |
| 75 | 24(O ) -- 17(C ) | 0.29 | 0.33 | -0.74 | -0.41 | -0.31 | 0.02 | Covalent bond |
| **76** | **34(H ) -- 4(N )** | **0.02** | **0.01** | **-0.01** | **0.00** | **0.06** | **0.05** | **H-bond** |
| 77 | 34(H ) -- 16(C ) | 0.28 | 0.04 | -0.32 | -0.28 | -0.99 | 0.02 | Covalent bond |
| 78 | 32(H ) -- 11(C ) | 0.28 | 0.03 | -0.31 | -0.28 | -0.98 | 0.02 | Covalent bond |
| 79 | 17(C ) -- 16(C ) | 0.31 | 0.10 | -0.43 | -0.32 | -0.89 | 0.24 | Covalent bond |
| 80 | 5(C ) -- 4(N ) | 0.38 | 0.28 | -0.84 | -0.56 | -1.13 | 0.23 | Covalent bond |
| 81 | 17(C ) -- 18(C ) | 0.31 | 0.10 | -0.42 | -0.32 | -0.86 | 0.24 | Covalent bond |
| 82 | 31(H ) -- 10(C ) | 0.28 | 0.04 | -0.32 | -0.28 | -0.98 | 0.02 | Covalent bond |
| 83 | 35(H ) -- 18(C ) | 0.28 | 0.04 | -0.32 | -0.28 | -0.97 | 0.02 | Covalent bond |
| 84 | 5(C ) -- 1(N ) | 0.31 | 0.19 | -0.60 | -0.41 | -0.87 | 0.11 | Covalent bond |
| 86 | 11(C ) -- 10(C ) | 0.31 | 0.10 | -0.42 | -0.32 | -0.86 | 0.20 | Covalent bond |
| 87 | 16(C ) -- 15(C ) | 0.30 | 0.10 | -0.40 | -0.30 | -0.82 | 0.21 | Covalent bond |
| 88 | 11(C ) -- 6(C ) | 0.31 | 0.10 | -0.42 | -0.32 | -0.87 | 0.24 | Covalent bond |
| 89 | 4(N ) -- 3(C ) | 0.31 | 0.17 | -0.56 | -0.39 | -0.89 | 0.08 | Covalent bond |
| 92 | 10(C ) -- 9(C ) | 0.31 | 0.10 | -0.42 | -0.32 | -0.86 | 0.19 | Covalent bond |
| 93 | 18(C ) -- 19(C ) | 0.30 | 0.10 | -0.41 | -0.31 | -0.83 | 0.21 | Covalent bond |
| 94 | 1(N ) -- 6(C ) | 0.27 | 0.19 | -0.54 | -0.36 | -0.69 | 0.03 | Covalent bond |
| 96 | 9(C ) -- 30(H ) | 0.28 | 0.04 | -0.32 | -0.28 | -0.98 | 0.01 | Covalent bond |
| 97 | 3(C ) -- 14(C ) | 0.33 | 0.12 | -0.48 | -0.36 | -0.96 | 0.29 | Covalent bond |
| 98 | 15(C ) -- 14(C ) | 0.28 | 0.08 | -0.34 | -0.26 | -0.73 | 0.11 | Covalent bond |
| 99 | 1(N ) -- 2(C ) | 0.29 | 0.18 | -0.56 | -0.38 | -0.80 | 0.09 | Covalent bond |
| 100 | 6(C ) -- 7(C ) | 0.31 | 0.10 | -0.42 | -0.32 | -0.87 | 0.24 | Covalent bond |
| 101 | 3(C ) -- 2(C ) | 0.27 | 0.07 | -0.31 | -0.24 | -0.68 | 0.15 | Covalent bond |
| 102 | 15(C ) -- 20(C ) | 0.30 | 0.09 | -0.38 | -0.29 | -0.81 | 0.23 | Covalent bond |
| 103 | 9(C ) -- 8(C ) | 0.31 | 0.10 | -0.42 | -0.32 | -0.86 | 0.19 | Covalent bond |
| 104 | 19(C ) -- 20(C ) | 0.31 | 0.11 | -0.43 | -0.32 | -0.86 | 0.25 | Covalent bond |
| 105 | 19(C ) -- 36(H ) | 0.28 | 0.04 | -0.32 | -0.28 | -0.97 | 0.02 | Covalent bond |
| 106 | 7(C ) -- 8(C ) | 0.31 | 0.10 | -0.42 | -0.32 | -0.87 | 0.20 | Covalent bond |
| 107 | 2(C ) -- 12(O ) | 0.41 | 0.65 | -1.33 | -0.69 | -0.16 | 0.06 | Covalent bond |
| 108 | 7(C ) -- 28(H ) | 0.28 | 0.03 | -0.31 | -0.28 | -0.98 | 0.02 | Covalent bond |
| 109 | 20(C ) -- 22(O ) | 0.29 | 0.35 | -0.76 | -0.42 | -0.29 | 0.01 | Covalent bond |
| 110 | 14(C ) -- 33(H ) | 0.28 | 0.04 | -0.32 | -0.28 | -0.99 | 0.01 | Covalent bond |
| 111 | 8(C ) -- 29(H ) | 0.28 | 0.04 | -0.32 | -0.28 | -0.98 | 0.02 | Covalent bond |
| 112 | 41(H ) -- 23(C ) | 0.28 | 0.04 | -0.31 | -0.28 | -0.96 | 0.04 | Covalent bond |
| 113 | 22(O ) -- 23(C ) | 0.24 | 0.27 | -0.59 | -0.32 | -0.22 | 0.00 | Covalent bond |
| 114 | 23(C ) -- 40(H ) | 0.28 | 0.04 | -0.31 | -0.27 | -0.95 | 0.04 | Covalent bond |
| 115 | 23(C ) -- 39(H ) | 0.28 | 0.04 | -0.32 | -0.28 | -0.99 | 0.04 | Covalent bond |

**Table S61:** Topological parameters and energy density descriptors for compound **2h** (Gas phase) at bond critical points (BCP), including atomic connectivity, electron density ρ(r) (a.u.), Lagrangian kinetic energy density G(r) (a.u.), potential energy density V(r) (a.u.), total energy density E(r) or H(r) (a.u.), Laplacian of electron density ∇²ρ(r) (a.u.), ellipticity ε (dimensionless), and bond type.

| CP | Connected atoms | ρ(r) | G(r) | V(r) | H(r) | ∇²ρ(r) | ε | Bond Type |
| --- | --- | --- | --- | --- | --- | --- | --- | --- |
| 44 | 34(H ) -- 22(C ) | 0.28 | 0.04 | -0.32 | -0.28 | -0.99 | 0.04 | Covalent bond |
| 45 | 36(H ) -- 22(C ) | 0.28 | 0.04 | -0.31 | -0.27 | -0.94 | 0.04 | Covalent bond |
| 46 | 22(C ) -- 35(H ) | 0.28 | 0.04 | -0.31 | -0.27 | -0.94 | 0.04 | Covalent bond |
| 47 | 22(C ) -- 21(O ) | 0.25 | 0.27 | -0.62 | -0.34 | -0.27 | 0.01 | Covalent bond |
| 48 | 26(H ) -- 8(C ) | 0.28 | 0.04 | -0.32 | -0.28 | -0.97 | 0.02 | Covalent bond |
| 49 | 12(O ) -- 2(C ) | 0.41 | 0.67 | -1.37 | -0.70 | -0.12 | 0.07 | Covalent bond |
| 50 | 30(H ) -- 14(C ) | 0.28 | 0.04 | -0.32 | -0.28 | -0.99 | 0.01 | Covalent bond |
| 51 | 27(H ) -- 9(C ) | 0.28 | 0.04 | -0.32 | -0.28 | -0.97 | 0.02 | Covalent bond |
| 52 | 21(O ) -- 20(C ) | 0.29 | 0.34 | -0.75 | -0.41 | -0.27 | 0.01 | Covalent bond |
| 53 | 8(C ) -- 9(C ) | 0.31 | 0.10 | -0.42 | -0.32 | -0.86 | 0.20 | Covalent bond |
| 54 | 8(C ) -- 7(C ) | 0.31 | 0.10 | -0.42 | -0.32 | -0.87 | 0.20 | Covalent bond |
| 55 | 25(H ) -- 7(C ) | 0.28 | 0.04 | -0.32 | -0.28 | -0.98 | 0.02 | Covalent bond |
| 56 | 9(C ) -- 10(C ) | 0.31 | 0.10 | -0.42 | -0.32 | -0.87 | 0.20 | Covalent bond |
| 58 | 7(C ) -- 6(C ) | 0.31 | 0.10 | -0.42 | -0.32 | -0.87 | 0.23 | Covalent bond |
| 59 | 2(C ) -- 1(N ) | 0.29 | 0.17 | -0.53 | -0.36 | -0.76 | 0.08 | Covalent bond |
| 60 | 2(C ) -- 3(C ) | 0.27 | 0.07 | -0.31 | -0.24 | -0.68 | 0.15 | Covalent bond |
| 61 | 33(H ) -- 19(C ) | 0.28 | 0.04 | -0.32 | -0.28 | -0.96 | 0.03 | Covalent bond |
| 62 | 20(C ) -- 19(C ) | 0.31 | 0.11 | -0.43 | -0.32 | -0.87 | 0.27 | Covalent bond |
| 63 | 14(C ) -- 3(C ) | 0.33 | 0.12 | -0.49 | -0.36 | -0.97 | 0.29 | Covalent bond |
| 64 | 20(C ) -- 15(C ) | 0.30 | 0.09 | -0.38 | -0.29 | -0.82 | 0.22 | Covalent bond |
| 65 | 10(C ) -- 28(H ) | 0.28 | 0.04 | -0.32 | -0.28 | -0.97 | 0.02 | Covalent bond |
| 66 | 10(C ) -- 11(C ) | 0.31 | 0.10 | -0.42 | -0.32 | -0.86 | 0.20 | Covalent bond |
| 67 | 14(C ) -- 15(C ) | 0.28 | 0.08 | -0.34 | -0.26 | -0.73 | 0.11 | Covalent bond |
| 68 | 6(C ) -- 11(C ) | 0.31 | 0.10 | -0.42 | -0.32 | -0.87 | 0.24 | Covalent bond |
| 69 | 6(C ) -- 1(N ) | 0.28 | 0.19 | -0.55 | -0.37 | -0.70 | 0.03 | Covalent bond |
| 70 | 11(C ) -- 29(H ) | 0.28 | 0.04 | -0.32 | -0.28 | -0.97 | 0.02 | Covalent bond |
| 72 | 3(C ) -- 4(N ) | 0.31 | 0.17 | -0.56 | -0.39 | -0.89 | 0.08 | Covalent bond |
| 73 | 19(C ) -- 18(C ) | 0.30 | 0.10 | -0.41 | -0.31 | -0.83 | 0.21 | Covalent bond |
| 75 | 15(C ) -- 16(C ) | 0.30 | 0.10 | -0.41 | -0.31 | -0.84 | 0.21 | Covalent bond |
| 76 | 1(N ) -- 5(C ) | 0.30 | 0.19 | -0.58 | -0.40 | -0.85 | 0.11 | Covalent bond |
| 78 | 4(N ) -- 5(C ) | 0.38 | 0.28 | -0.85 | -0.56 | -1.13 | 0.24 | Covalent bond |
| 79 | 18(C ) -- 32(H ) | 0.28 | 0.04 | -0.32 | -0.28 | -0.96 | 0.02 | Covalent bond |
| 80 | 18(C ) -- 17(C ) | 0.31 | 0.10 | -0.42 | -0.32 | -0.86 | 0.25 | Covalent bond |
| **81** | **4(N ) -- 31(H )** | **0.02** | **0.01** | **-0.01** | **0.00** | **0.06** | **0.05** | **H-bond** |
| 82 | 16(C ) -- 17(C ) | 0.31 | 0.10 | -0.43 | -0.33 | -0.89 | 0.24 | Covalent bond |
| 83 | 16(C ) -- 31(H ) | 0.28 | 0.03 | -0.32 | -0.28 | -1.00 | 0.02 | Covalent bond |
| 84 | 5(C ) -- 13(S ) | 0.20 | 0.07 | -0.23 | -0.17 | -0.41 | 0.27 | Covalent bond |
| 85 | 17(C ) -- 23(O ) | 0.28 | 0.33 | -0.73 | -0.40 | -0.30 | 0.01 | Covalent bond |
| 86 | 13(S ) -- 40(C ) | 0.18 | 0.05 | -0.17 | -0.12 | -0.28 | 0.07 | Covalent bond |
| 87 | 41(H ) -- 40(C ) | 0.28 | 0.04 | -0.31 | -0.27 | -0.94 | 0.01 | Covalent bond |
| 88 | 38(H ) -- 24(C ) | 0.28 | 0.04 | -0.31 | -0.27 | -0.94 | 0.04 | Covalent bond |
| 89 | 23(O ) -- 24(C ) | 0.26 | 0.27 | -0.62 | -0.35 | -0.30 | 0.01 | Covalent bond |
| 90 | 40(C ) -- 42(H ) | 0.28 | 0.04 | -0.31 | -0.27 | -0.94 | 0.01 | Covalent bond |
| 91 | 39(H ) -- 24(C ) | 0.28 | 0.04 | -0.31 | -0.27 | -0.94 | 0.04 | Covalent bond |
| 0 | 40(C ) -- 43(H ) | 0.28 | 0.04 | -0.31 | -0.27 | -0.93 | 0.01 | Covalent bond |
| 93 | 24(C ) -- 37(H ) | 0.28 | 0.04 | -0.32 | -0.28 | -0.98 | 0.04 | Covalent bond |

**Table S62:** Topological parameters and energy density descriptors for compound **2h** (DMSO phase) at bond critical points (BCP), including atomic connectivity, electron density ρ(r) (a.u.), Lagrangian kinetic energy density G(r) (a.u.), potential energy density V(r) (a.u.), total energy density E(r) or H(r) (a.u.), Laplacian of electron density ∇²ρ(r) (a.u.), ellipticity ε (dimensionless), and bond type.

| CP | Connected atoms | ρ(r) | G(r) | V(r) | H(r) | ∇²ρ(r) | ε | Bond Type |
| --- | --- | --- | --- | --- | --- | --- | --- | --- |
| 44 | 34(H ) -- 22(C ) | 0.28 | 0.04 | -0.32 | -0.28 | -0.99 | 0.04 | Covalent bond |
| 45 | 35(H ) -- 22(C ) | 0.28 | 0.04 | -0.31 | -0.27 | -0.95 | 0.04 | Covalent bond |
| 46 | 22(C ) -- 36(H ) | 0.28 | 0.04 | -0.31 | -0.27 | -0.95 | 0.04 | Covalent bond |
| 47 | 22(C ) -- 21(O ) | 0.24 | 0.27 | -0.59 | -0.32 | -0.22 | 0.00 | Covalent bond |
| 48 | 21(O ) -- 20(C ) | 0.29 | 0.34 | -0.76 | -0.42 | -0.29 | 0.02 | Covalent bond |
| 49 | 33(H ) -- 19(C ) | 0.28 | 0.04 | -0.32 | -0.28 | -0.97 | 0.02 | Covalent bond |
| 50 | 30(H ) -- 14(C ) | 0.28 | 0.04 | -0.32 | -0.28 | -0.99 | 0.01 | Covalent bond |
| 51 | 20(C ) -- 19(C ) | 0.31 | 0.11 | -0.43 | -0.32 | -0.86 | 0.25 | Covalent bond |
| 52 | 12(O ) -- 2(C ) | 0.41 | 0.64 | -1.33 | -0.68 | -0.16 | 0.06 | Covalent bond |
| 53 | 20(C ) -- 15(C ) | 0.30 | 0.09 | -0.38 | -0.29 | -0.81 | 0.23 | Covalent bond |
| 54 | 14(C ) -- 15(C ) | 0.28 | 0.08 | -0.34 | -0.26 | -0.73 | 0.11 | Covalent bond |
| 55 | 19(C ) -- 18(C ) | 0.30 | 0.10 | -0.41 | -0.31 | -0.83 | 0.21 | Covalent bond |
| 56 | 14(C ) -- 3(C ) | 0.33 | 0.12 | -0.48 | -0.36 | -0.96 | 0.29 | Covalent bond |
| 57 | 26(H ) -- 8(C ) | 0.28 | 0.04 | -0.32 | -0.28 | -0.98 | 0.02 | Covalent bond |
| 58 | 25(H ) -- 7(C ) | 0.28 | 0.03 | -0.31 | -0.28 | -0.98 | 0.02 | Covalent bond |
| 59 | 2(C ) -- 3(C ) | 0.27 | 0.07 | -0.31 | -0.24 | -0.68 | 0.15 | Covalent bond |
| 60 | 2(C ) -- 1(N ) | 0.29 | 0.18 | -0.57 | -0.38 | -0.80 | 0.09 | Covalent bond |
| 62 | 7(C ) -- 8(C ) | 0.31 | 0.10 | -0.42 | -0.32 | -0.87 | 0.20 | Covalent bond |
| 63 | 15(C ) -- 16(C ) | 0.30 | 0.10 | -0.40 | -0.30 | -0.82 | 0.21 | Covalent bond |
| 64 | 7(C ) -- 6(C ) | 0.31 | 0.10 | -0.42 | -0.32 | -0.87 | 0.24 | Covalent bond |
| 65 | 8(C ) -- 9(C ) | 0.31 | 0.10 | -0.42 | -0.32 | -0.86 | 0.19 | Covalent bond |
| 66 | 18(C ) -- 32(H ) | 0.28 | 0.04 | -0.32 | -0.28 | -0.97 | 0.02 | Covalent bond |
| 67 | 3(C ) -- 4(N ) | 0.31 | 0.17 | -0.55 | -0.39 | -0.89 | 0.08 | Covalent bond |
| 69 | 1(N ) -- 6(C ) | 0.27 | 0.18 | -0.54 | -0.36 | -0.69 | 0.03 | Covalent bond |
| 71 | 9(C ) -- 27(H ) | 0.28 | 0.04 | -0.32 | -0.28 | -0.98 | 0.01 | Covalent bond |
| 72 | 18(C ) -- 17(C ) | 0.31 | 0.10 | -0.42 | -0.32 | -0.86 | 0.24 | Covalent bond |
| 74 | 9(C ) -- 10(C ) | 0.31 | 0.10 | -0.42 | -0.32 | -0.86 | 0.19 | Covalent bond |
| 75 | 6(C ) -- 11(C ) | 0.31 | 0.10 | -0.42 | -0.32 | -0.87 | 0.24 | Covalent bond |
| 76 | 16(C ) -- 17(C ) | 0.31 | 0.10 | -0.43 | -0.33 | -0.89 | 0.24 | Covalent bond |
| 77 | 11(C ) -- 10(C ) | 0.31 | 0.10 | -0.42 | -0.32 | -0.86 | 0.20 | Covalent bond |
| 78 | 1(N ) -- 5(C ) | 0.30 | 0.19 | -0.59 | -0.40 | -0.86 | 0.11 | Covalent bond |
| 79 | 16(C ) -- 31(H ) | 0.28 | 0.04 | -0.32 | -0.28 | -0.99 | 0.02 | Covalent bond |
| 80 | 11(C ) -- 29(H ) | 0.28 | 0.03 | -0.31 | -0.28 | -0.98 | 0.02 | Covalent bond |
| 81 | 10(C ) -- 28(H ) | 0.28 | 0.04 | -0.32 | -0.28 | -0.98 | 0.02 | Covalent bond |
| **82** | **4(N ) -- 31(H )** | **0.02** | **0.01** | **-0.01** | **0.00** | **0.06** | **0.04** | **H-bond** |
| 83 | 4(N ) -- 5(C ) | 0.37 | 0.28 | -0.83 | -0.56 | -1.13 | 0.23 | Covalent bond |
| 84 | 17(C ) -- 23(O ) | 0.29 | 0.33 | -0.74 | -0.41 | -0.32 | 0.02 | Covalent bond |
| 85 | 5(C ) -- 13(S ) | 0.21 | 0.07 | -0.24 | -0.17 | -0.42 | 0.28 | Covalent bond |
| 86 | 39(H ) -- 24(C ) | 0.28 | 0.04 | -0.31 | -0.27 | -0.95 | 0.04 | Covalent bond |
| 87 | 23(O ) -- 24(C ) | 0.25 | 0.27 | -0.60 | -0.33 | -0.26 | 0.00 | Covalent bond |
| 88 | 38(H ) -- 24(C ) | 0.28 | 0.04 | -0.31 | -0.27 | -0.95 | 0.04 | Covalent bond |
| 89 | 24(C ) -- 37(H ) | 0.28 | 0.04 | -0.32 | -0.28 | -0.99 | 0.04 | Covalent bond |
| 90 | 13(S ) -- 40(C ) | 0.18 | 0.05 | -0.17 | -0.12 | -0.28 | 0.06 | Covalent bond |
| 91 | 42(H ) -- 40(C ) | 0.28 | 0.04 | -0.31 | -0.27 | -0.94 | 0.01 | Covalent bond |
| 92 | 40(C ) -- 41(H ) | 0.28 | 0.04 | -0.31 | -0.27 | -0.94 | 0.01 | Covalent bond |
| 93 | 40(C ) -- 43(H ) | 0.28 | 0.04 | -0.31 | -0.27 | -0.94 | 0.01 | Covalent bond |

**Table S63:** Topological parameters and energy density descriptors for compound **2i** (Gas phase) at bond critical points (BCP), including atomic connectivity, electron density ρ(r) (a.u.), Lagrangian kinetic energy density G(r) (a.u.), potential energy density V(r) (a.u.), total energy density E(r) or H(r) (a.u.), Laplacian of electron density ∇²ρ(r) (a.u.), ellipticity ε (dimensionless), and bond type.

| CP | Connected atoms | ρ(r) | G(r) | V(r) | H(r) | ∇²ρ(r) | ε | Bond Type |
| --- | --- | --- | --- | --- | --- | --- | --- | --- |
| 46 | 34(H ) -- 22(C ) | 0.28 | 0.04 | -0.32 | -0.28 | -0.99 | 0.04 | Covalent bond |
| 47 | 36(H ) -- 22(C ) | 0.28 | 0.04 | -0.31 | -0.27 | -0.94 | 0.04 | Covalent bond |
| 48 | 22(C ) -- 35(H ) | 0.28 | 0.04 | -0.31 | -0.27 | -0.94 | 0.04 | Covalent bond |
| 49 | 22(C ) -- 21(O ) | 0.25 | 0.27 | -0.61 | -0.34 | -0.26 | 0.00 | Covalent bond |
| 50 | 30(H ) -- 14(C ) | 0.28 | 0.03 | -0.32 | -0.28 | -1.00 | 0.01 | Covalent bond |
| 51 | 12(O ) -- 2(C ) | 0.41 | 0.67 | -1.37 | -0.70 | -0.12 | 0.07 | Covalent bond |
| 52 | 26(H ) -- 8(C ) | 0.28 | 0.04 | -0.32 | -0.28 | -0.97 | 0.02 | Covalent bond |
| 53 | 21(O ) -- 20(C ) | 0.29 | 0.34 | -0.76 | -0.41 | -0.27 | 0.01 | Covalent bond |
| 54 | 27(H ) -- 9(C ) | 0.28 | 0.04 | -0.32 | -0.28 | -0.97 | 0.02 | Covalent bond |
| 55 | 8(C ) -- 9(C ) | 0.31 | 0.10 | -0.42 | -0.32 | -0.87 | 0.20 | Covalent bond |
| 56 | 8(C ) -- 7(C ) | 0.31 | 0.10 | -0.42 | -0.32 | -0.87 | 0.20 | Covalent bond |
| 57 | 25(H ) -- 7(C ) | 0.28 | 0.04 | -0.32 | -0.28 | -0.98 | 0.02 | Covalent bond |
| 58 | 9(C ) -- 10(C ) | 0.31 | 0.10 | -0.42 | -0.32 | -0.87 | 0.20 | Covalent bond |
| 60 | 2(C ) -- 1(N ) | 0.28 | 0.17 | -0.53 | -0.36 | -0.76 | 0.08 | Covalent bond |
| 61 | 33(H ) -- 19(C ) | 0.28 | 0.04 | -0.32 | -0.28 | -0.96 | 0.03 | Covalent bond |
| 62 | 2(C ) -- 3(C ) | 0.27 | 0.07 | -0.31 | -0.24 | -0.69 | 0.16 | Covalent bond |
| 63 | 7(C ) -- 6(C ) | 0.31 | 0.10 | -0.42 | -0.32 | -0.88 | 0.24 | Covalent bond |
| 64 | 20(C ) -- 19(C ) | 0.31 | 0.11 | -0.43 | -0.32 | -0.86 | 0.26 | Covalent bond |
| 65 | 10(C ) -- 28(H ) | 0.28 | 0.04 | -0.32 | -0.28 | -0.97 | 0.02 | Covalent bond |
| 66 | 20(C ) -- 15(C ) | 0.30 | 0.09 | -0.38 | -0.29 | -0.82 | 0.22 | Covalent bond |
| 67 | 14(C ) -- 3(C ) | 0.33 | 0.12 | -0.48 | -0.36 | -0.97 | 0.29 | Covalent bond |
| 68 | 10(C ) -- 11(C ) | 0.31 | 0.10 | -0.42 | -0.32 | -0.87 | 0.20 | Covalent bond |
| 69 | 14(C ) -- 15(C ) | 0.28 | 0.08 | -0.34 | -0.26 | -0.74 | 0.11 | Covalent bond |
| 70 | 6(C ) -- 11(C ) | 0.31 | 0.10 | -0.42 | -0.32 | -0.87 | 0.24 | Covalent bond |
| 71 | 6(C ) -- 1(N ) | 0.27 | 0.19 | -0.55 | -0.36 | -0.69 | 0.03 | Covalent bond |
| 72 | 11(C ) -- 29(H ) | 0.28 | 0.04 | -0.32 | -0.28 | -0.97 | 0.02 | Covalent bond |
| 74 | 3(C ) -- 4(N ) | 0.30 | 0.17 | -0.56 | -0.39 | -0.87 | 0.08 | Covalent bond |
| 75 | 19(C ) -- 18(C ) | 0.30 | 0.10 | -0.41 | -0.31 | -0.83 | 0.21 | Covalent bond |
| 77 | 15(C ) -- 16(C ) | 0.30 | 0.10 | -0.41 | -0.31 | -0.84 | 0.21 | Covalent bond |
| 78 | 1(N ) -- 5(C ) | 0.30 | 0.19 | -0.59 | -0.40 | -0.85 | 0.13 | Covalent bond |
| 80 | 4(N ) -- 5(C ) | 0.38 | 0.29 | -0.85 | -0.57 | -1.13 | 0.25 | Covalent bond |
| 81 | 18(C ) -- 32(H ) | 0.28 | 0.04 | -0.32 | -0.28 | -0.96 | 0.02 | Covalent bond |
| 82 | 18(C ) -- 17(C ) | 0.31 | 0.10 | -0.42 | -0.32 | -0.86 | 0.25 | Covalent bond |
| 83 | 4(N ) -- 31(H ) | 0.02 | 0.01 | -0.01 | 0.00 | 0.06 | 0.05 | H-bond |
| 84 | 16(C ) -- 17(C ) | 0.31 | 0.10 | -0.43 | -0.33 | -0.90 | 0.24 | Covalent bond |
| 85 | 16(C ) -- 31(H ) | 0.29 | 0.04 | -0.32 | -0.29 | -1.00 | 0.02 | Covalent bond |
| 86 | 5(C ) -- 13(S ) | 0.20 | 0.06 | -0.21 | -0.15 | -0.37 | 0.22 | Covalent bond |
| 87 | 17(C ) -- 23(O ) | 0.29 | 0.33 | -0.74 | -0.41 | -0.31 | 0.02 | Covalent bond |
| 88 | 4(N ) -- 42(C ) | 0.01 | 0.01 | -0.01 | 0.00 | 0.06 | 4.24 | Van der Waal |
| 90 | 13(S ) -- 40(C ) | 0.17 | 0.05 | -0.16 | -0.10 | -0.22 | 0.18 | Covalent bond |
| 91 | 43(H ) -- 42(C ) | 0.28 | 0.03 | -0.31 | -0.28 | -0.97 | 0.03 | Covalent bond |
| 92 | 38(H ) -- 24(C ) | 0.28 | 0.04 | -0.31 | -0.27 | -0.94 | 0.04 | Covalent bond |
| 94 | 23(O ) -- 24(C ) | 0.25 | 0.27 | -0.61 | -0.34 | -0.29 | 0.00 | Covalent bond |
| 95 | 44(H ) -- 42(C ) | 0.28 | 0.03 | -0.31 | -0.28 | -0.97 | 0.03 | Covalent bond |
| 96 | 24(C ) -- 39(H ) | 0.28 | 0.04 | -0.31 | -0.27 | -0.94 | 0.04 | Covalent bond |
| 97 | 42(C ) -- 40(C ) | 0.25 | 0.06 | -0.27 | -0.21 | -0.61 | 0.08 | Covalent bond |
| 98 | 23(O ) -- 45(Cl) | 0.00 | 0.00 | 0.00 | 0.00 | 0.01 | 0.02 | Halogen bond |
| 99 | 40(C ) -- 41(O ) | 0.43 | 0.76 | -1.49 | -0.73 | 0.11 | 0.06 | Covalent bond |
| 100 | 24(C ) -- 37(H ) | 0.28 | 0.04 | -0.32 | -0.28 | -0.99 | 0.04 | Covalent bond |
| 101 | 42(C ) -- 45(Cl) | 0.19 | 0.06 | -0.19 | -0.12 | -0.25 | 0.01 | Covalent bond |

**Table S64:** Topological parameters and energy density descriptors for compound **2i** (DMSO phase) at bond critical points (BCP), including atomic connectivity, electron density ρ(r) (a.u.), Lagrangian kinetic energy density G(r) (a.u.), potential energy density V(r) (a.u.), total energy density E(r) or H(r) (a.u.), Laplacian of electron density ∇²ρ(r) (a.u.), ellipticity ε (dimensionless), and bond type.

| CP | Connected atoms | ρ(r) | G(r) | V(r) | H(r) | ∇²ρ(r) | ε | Bond Type |
| --- | --- | --- | --- | --- | --- | --- | --- | --- |
| 46 | 34(H ) -- 22(C ) | 0.28 | 0.04 | -0.32 | -0.28 | -0.99 | 0.04 | Covalent bond |
| 47 | 35(H ) -- 22(C ) | 0.28 | 0.04 | -0.31 | -0.28 | -0.96 | 0.04 | Covalent bond |
| 48 | 22(C ) -- 36(H ) | 0.28 | 0.04 | -0.31 | -0.28 | -0.96 | 0.04 | Covalent bond |
| 49 | 22(C ) -- 21(O ) | 0.24 | 0.27 | -0.59 | -0.32 | -0.22 | 0.00 | Covalent bond |
| 0 | 21(O ) -- 20(C ) | 0.29 | 0.35 | -0.77 | -0.42 | -0.29 | 0.01 | Covalent bond |
| 51 | 33(H ) -- 19(C ) | 0.28 | 0.04 | -0.32 | -0.28 | -0.97 | 0.02 | Covalent bond |
| 52 | 30(H ) -- 14(C ) | 0.28 | 0.03 | -0.32 | -0.28 | -1.00 | 0.01 | Covalent bond |
| 53 | 20(C ) -- 19(C ) | 0.31 | 0.11 | -0.43 | -0.32 | -0.86 | 0.25 | Covalent bond |
| 54 | 12(O ) -- 2(C ) | 0.41 | 0.65 | -1.33 | -0.69 | -0.16 | 0.06 | Covalent bond |
| 55 | 20(C ) -- 15(C ) | 0.30 | 0.09 | -0.38 | -0.29 | -0.81 | 0.23 | Covalent bond |
| 56 | 14(C ) -- 15(C ) | 0.28 | 0.08 | -0.34 | -0.26 | -0.74 | 0.11 | Covalent bond |
| 57 | 19(C ) -- 18(C ) | 0.30 | 0.10 | -0.41 | -0.31 | -0.84 | 0.21 | Covalent bond |
| 58 | 14(C ) -- 3(C ) | 0.33 | 0.12 | -0.48 | -0.36 | -0.96 | 0.28 | Covalent bond |
| 59 | 2(C ) -- 3(C ) | 0.27 | 0.07 | -0.31 | -0.24 | -0.69 | 0.16 | Covalent bond |
| 60 | 2(C ) -- 1(N ) | 0.29 | 0.18 | -0.56 | -0.38 | -0.79 | 0.09 | Covalent bond |
| 62 | 25(H ) -- 7(C ) | 0.28 | 0.03 | -0.31 | -0.28 | -0.98 | 0.02 | Covalent bond |
| 63 | 26(H ) -- 8(C ) | 0.28 | 0.04 | -0.32 | -0.28 | -0.98 | 0.02 | Covalent bond |
| 64 | 7(C ) -- 8(C ) | 0.31 | 0.10 | -0.42 | -0.32 | -0.87 | 0.20 | Covalent bond |
| 65 | 15(C ) -- 16(C ) | 0.30 | 0.10 | -0.40 | -0.30 | -0.82 | 0.21 | Covalent bond |
| 66 | 18(C ) -- 32(H ) | 0.28 | 0.04 | -0.32 | -0.28 | -0.98 | 0.02 | Covalent bond |
| 67 | 7(C ) -- 6(C ) | 0.31 | 0.10 | -0.42 | -0.32 | -0.87 | 0.24 | Covalent bond |
| 68 | 8(C ) -- 9(C ) | 0.31 | 0.10 | -0.42 | -0.32 | -0.86 | 0.19 | Covalent bond |
| 69 | 3(C ) -- 4(N ) | 0.30 | 0.17 | -0.56 | -0.39 | -0.87 | 0.08 | Covalent bond |
| 71 | 1(N ) -- 6(C ) | 0.27 | 0.19 | -0.54 | -0.36 | -0.68 | 0.02 | Covalent bond |
| 73 | 9(C ) -- 27(H ) | 0.28 | 0.04 | -0.32 | -0.28 | -0.98 | 0.01 | Covalent bond |
| 74 | 18(C ) -- 17(C ) | 0.31 | 0.10 | -0.42 | -0.32 | -0.86 | 0.23 | Covalent bond |
| 76 | 6(C ) -- 11(C ) | 0.31 | 0.10 | -0.42 | -0.32 | -0.87 | 0.24 | Covalent bond |
| 77 | 9(C ) -- 10(C ) | 0.31 | 0.10 | -0.42 | -0.32 | -0.86 | 0.19 | Covalent bond |
| 78 | 11(C ) -- 10(C ) | 0.31 | 0.10 | -0.42 | -0.32 | -0.87 | 0.19 | Covalent bond |
| 79 | 16(C ) -- 17(C ) | 0.31 | 0.10 | -0.43 | -0.33 | -0.89 | 0.24 | Covalent bond |
| 80 | 11(C ) -- 29(H ) | 0.28 | 0.03 | -0.31 | -0.28 | -0.98 | 0.02 | Covalent bond |
| 81 | 10(C ) -- 28(H ) | 0.28 | 0.04 | -0.32 | -0.28 | -0.98 | 0.02 | Covalent bond |
| 82 | 1(N ) -- 5(C ) | 0.31 | 0.19 | -0.59 | -0.40 | -0.87 | 0.13 | Covalent bond |
| 83 | 16(C ) -- 31(H ) | 0.28 | 0.04 | -0.32 | -0.28 | -0.99 | 0.02 | Covalent bond |
| **84** | **4(N ) -- 31(H )** | **0.02** | **0.01** | **-0.01** | **0.00** | **0.06** | **0.05** | **H-bond** |
| 85 | 4(N ) -- 5(C ) | 0.38 | 0.29 | -0.86 | -0.57 | -1.13 | 0.25 | Covalent bond |
| 86 | 17(C ) -- 23(O ) | 0.29 | 0.33 | -0.75 | -0.41 | -0.32 | 0.02 | Covalent bond |
| 87 | 5(C ) -- 13(S ) | 0.20 | 0.06 | -0.21 | -0.15 | -0.37 | 0.21 | Covalent bond |
| 88 | 39(H ) -- 24(C ) | 0.28 | 0.04 | -0.31 | -0.27 | -0.95 | 0.04 | Covalent bond |
| **89** | **4(N ) -- 42(C )** | **0.01** | **0.01** | **-0.01** | **0.00** | **0.05** | **6.72** | **Van der Waal** |
| 90 | 23(O ) -- 24(C ) | 0.25 | 0.27 | -0.60 | -0.33 | -0.25 | 0.00 | Covalent bond |
| 91 | 38(H ) -- 24(C ) | 0.28 | 0.04 | -0.31 | -0.27 | -0.95 | 0.04 | Covalent bond |
| 94 | 44(H ) -- 42(C ) | 0.28 | 0.03 | -0.31 | -0.28 | -0.98 | 0.03 | Covalent bond |
| 95 | 24(C ) -- 37(H ) | 0.28 | 0.04 | -0.32 | -0.28 | -0.99 | 0.04 | Covalent bond |
| 96 | 43(H ) -- 42(C ) | 0.28 | 0.03 | -0.31 | -0.28 | -0.99 | 0.03 | Covalent bond |
| **97** | **23(O ) -- 45(Cl)** | **0.00** | **0.00** | **0.00** | **0.00** | **0.00** | **0.01** | **Halogen bond** |
| 98 | 13(S ) -- 40(C ) | 0.17 | 0.05 | -0.16 | -0.11 | -0.24 | 0.18 | Covalent bond |
| 99 | 42(C ) -- 40(C ) | 0.26 | 0.06 | -0.28 | -0.22 | -0.62 | 0.08 | Covalent bond |
| 100 | 42(C ) -- 45(Cl) | 0.18 | 0.06 | -0.18 | -0.12 | -0.24 | 0.01 | Covalent bond |
| 101 | 40(C ) -- 41(O ) | 0.42 | 0.74 | -1.45 | -0.72 | 0.07 | 0.06 | Covalent bond |

**Table S65:** Topological parameters and energy density descriptors for compound **3** (Gas phase) at bond critical points (BCP), including atomic connectivity, electron density ρ(r) (a.u.), Lagrangian kinetic energy density G(r) (a.u.), potential energy density V(r) (a.u.), total energy density E(r) or H(r) (a.u.), Laplacian of electron density ∇²ρ(r) (a.u.), ellipticity ε (dimensionless), and bond type.

| CP | Connected atoms | ρ(r) | G(r) | V(r) | H(r) | ∇²ρ(r) | ε | Bond Type |
| --- | --- | --- | --- | --- | --- | --- | --- | --- |
| 48 | 32(H ) -- 9(C ) | 0.28 | 0.04 | -0.32 | -0.28 | -0.98 | 0.01 | Covalent bond |
| 49 | 8(S ) -- 3(C ) | 0.22 | 0.28 | -0.54 | -0.26 | 0.08 | 0.06 | Covalent bond |
| 50 | 30(H ) -- 4(N ) | 0.34 | 0.05 | -0.54 | -0.49 | -1.76 | 0.05 | Covalent bond |
| 51 | 4(N ) -- 3(C ) | 0.33 | 0.21 | -0.66 | -0.45 | -0.97 | 0.13 | Covalent bond |
| 52 | 33(H ) -- 10(C ) | 0.28 | 0.04 | -0.32 | -0.28 | -0.97 | 0.02 | Covalent bond |
| 53 | 3(C ) -- 2(N ) | 0.30 | 0.17 | -0.56 | -0.39 | -0.86 | 0.08 | Covalent bond |
| 54 | 9(C ) -- 10(C ) | 0.31 | 0.10 | -0.42 | -0.32 | -0.86 | 0.20 | Covalent bond |
| 55 | 31(H ) -- 5(C ) | 0.28 | 0.04 | -0.31 | -0.27 | -0.96 | 0.03 | Covalent bond |
| 56 | 9(C ) -- 6(C ) | 0.31 | 0.10 | -0.43 | -0.32 | -0.88 | 0.24 | Covalent bond |
| 58 | 4(N ) -- 5(C ) | 0.27 | 0.14 | -0.47 | -0.33 | -0.73 | 0.02 | Covalent bond |
| 59 | 2(N ) -- 6(C ) | 0.27 | 0.20 | -0.55 | -0.36 | -0.65 | 0.03 | Covalent bond |
| 60 | 2(N ) -- 1(C ) | 0.31 | 0.19 | -0.59 | -0.40 | -0.86 | 0.10 | Covalent bond |
| 61 | 5(C ) -- 1(C ) | 0.26 | 0.06 | -0.27 | -0.21 | -0.62 | 0.08 | Covalent bond |
| **62** | **8(S ) -- 44(H )** | **0.00** | **0.00** | **0.00** | **0.00** | **0.01** | **0.00** | **Van der Waal** |
| 63 | 10(C ) -- 11(C ) | 0.31 | 0.10 | -0.42 | -0.32 | -0.87 | 0.20 | Covalent bond |
| 64 | 1(C ) -- 7(O ) | 0.42 | 0.69 | -1.40 | -0.71 | -0.09 | 0.07 | Covalent bond |
| **65** | **30(H ) -- 26(O )** | **0.02** | **0.01** | **-0.01** | **0.00** | **0.05** | **0.37** | **H-bond** |
| 68 | 23(N ) -- 22(C ) | 0.48 | 0.83 | -1.69 | -0.86 | -0.14 | 0.00 | Covalent bond |
| 69 | 6(C ) -- 13(C ) | 0.31 | 0.10 | -0.43 | -0.32 | -0.87 | 0.23 | Covalent bond |
| 70 | 5(C ) -- 14(C ) | 0.23 | 0.06 | -0.24 | -0.18 | -0.49 | 0.02 | Covalent bond |
| 72 | 11(C ) -- 34(H ) | 0.28 | 0.04 | -0.32 | -0.28 | -0.97 | 0.02 | Covalent bond |
| 73 | 42(H ) -- 27(C ) | 0.28 | 0.04 | -0.32 | -0.28 | -0.98 | 0.04 | Covalent bond |
| 74 | 11(C ) -- 12(C ) | 0.31 | 0.10 | -0.42 | -0.32 | -0.86 | 0.20 | Covalent bond |
| 75 | 44(H ) -- 27(C ) | 0.28 | 0.04 | -0.31 | -0.28 | -0.96 | 0.04 | Covalent bond |
| 76 | 22(C ) -- 16(C ) | 0.26 | 0.07 | -0.32 | -0.24 | -0.68 | 0.03 | Covalent bond |
| 77 | 13(C ) -- 12(C ) | 0.31 | 0.10 | -0.42 | -0.32 | -0.87 | 0.20 | Covalent bond |
| 78 | 38(H ) -- 16(C ) | 0.28 | 0.04 | -0.31 | -0.27 | -0.96 | 0.01 | Covalent bond |
| **79** | **38(H ) -- 26(O )** | **0.02** | **0.02** | **-0.01** | **0.00** | **0.08** | **0.07** | **H-bond** |
| 80 | 13(C ) -- 36(H ) | 0.28 | 0.04 | -0.32 | -0.28 | -0.99 | 0.01 | Covalent bond |
| 82 | 27(C ) -- 26(O ) | 0.24 | 0.27 | -0.59 | -0.33 | -0.23 | 0.01 | Covalent bond |
| 83 | 14(C ) -- 16(C ) | 0.23 | 0.05 | -0.23 | -0.18 | -0.48 | 0.04 | Covalent bond |
| 86 | 14(C ) -- 37(H ) | 0.28 | 0.04 | -0.31 | -0.28 | -0.95 | 0.01 | Covalent bond |
| 87 | 12(C ) -- 35(H ) | 0.28 | 0.04 | -0.32 | -0.28 | -0.97 | 0.02 | Covalent bond |
| 88 | 14(C ) -- 15(C ) | 0.25 | 0.06 | -0.26 | -0.20 | -0.58 | 0.04 | Covalent bond |
| 89 | 27(C ) -- 43(H ) | 0.28 | 0.04 | -0.31 | -0.28 | -0.95 | 0.04 | Covalent bond |
| 90 | 26(O ) -- 17(C ) | 0.28 | 0.32 | -0.71 | -0.39 | -0.28 | 0.03 | Covalent bond |
| 91 | 16(C ) -- 24(C ) | 0.26 | 0.07 | -0.31 | -0.24 | -0.68 | 0.04 | Covalent bond |
| 92 | 17(C ) -- 15(C ) | 0.31 | 0.10 | -0.41 | -0.31 | -0.85 | 0.28 | Covalent bond |
| **93** | **13(C ) -- 47(H )** | **0.00** | **0.00** | **0.00** | **0.00** | **0.01** | **0.51** | **Van der Waal** |
| **94** | **36(H ) -- 20(C )** | **0.01** | **0.00** | **0.00** | **0.00** | **0.02** | **1.96** | **Van der Waal** |
| 96 | 17(C ) -- 18(C ) | 0.31 | 0.10 | -0.42 | -0.32 | -0.85 | 0.24 | Covalent bond |
| 97 | 15(C ) -- 21(C ) | 0.30 | 0.10 | -0.41 | -0.31 | -0.82 | 0.23 | Covalent bond |
| 98 | 39(H ) -- 18(C ) | 0.28 | 0.04 | -0.32 | -0.28 | -0.97 | 0.02 | Covalent bond |
| 100 | 21(C ) -- 41(H ) | 0.28 | 0.04 | -0.32 | -0.28 | -0.97 | 0.02 | Covalent bond |
| 101 | 24(C ) -- 25(N ) | 0.48 | 0.83 | -1.70 | -0.86 | -0.13 | 0.00 | Covalent bond |
| 102 | 18(C ) -- 19(C ) | 0.31 | 0.11 | -0.43 | -0.32 | -0.86 | 0.22 | Covalent bond |
| 103 | 21(C ) -- 20(C ) | 0.31 | 0.10 | -0.42 | -0.32 | -0.86 | 0.25 | Covalent bond |
| 104 | 47(H ) -- 29(C ) | 0.28 | 0.04 | -0.31 | -0.27 | -0.95 | 0.04 | Covalent bond |
| 105 | 19(C ) -- 20(C ) | 0.31 | 0.10 | -0.42 | -0.32 | -0.89 | 0.24 | Covalent bond |
| 106 | 20(C ) -- 28(O ) | 0.29 | 0.34 | -0.76 | -0.42 | -0.31 | 0.02 | Covalent bond |
| 107 | 19(C ) -- 40(H ) | 0.28 | 0.04 | -0.32 | -0.28 | -0.97 | 0.02 | Covalent bond |
| 108 | 29(C ) -- 28(O ) | 0.25 | 0.27 | -0.60 | -0.33 | -0.27 | 0.01 | Covalent bond |
| 109 | 29(C ) -- 46(H ) | 0.28 | 0.04 | -0.31 | -0.27 | -0.94 | 0.04 | Covalent bond |
| 110 | 29(C ) -- 45(H ) | 0.28 | 0.04 | -0.32 | -0.28 | -0.99 | 0.04 | Covalent bond |

**Table S66:** Topological parameters and energy density descriptors for compound **3** (DMSO phase) at bond critical points (BCP), including atomic connectivity, electron density ρ(r) (a.u.), Lagrangian kinetic energy density G(r) (a.u.), potential energy density V(r) (a.u.), total energy density E(r) or H(r) (a.u.), Laplacian of electron density ∇²ρ(r) (a.u.), ellipticity ε (dimensionless), and bond type.

| CP | Connected atoms | ρ(r) | G(r) | V(r) | H(r) | ∇²ρ(r) | ε | Bond Type |
| --- | --- | --- | --- | --- | --- | --- | --- | --- |
| 48 | 32(H ) -- 9(C ) | 0.28 | 0.04 | -0.31 | -0.28 | -0.98 | 0.02 | Covalent bond |
| 49 | 8(S ) -- 3(C ) | 0.21 | 0.25 | -0.51 | -0.25 | -0.01 | 0.07 | Covalent bond |
| 50 | 30(H ) -- 4(N ) | 0.34 | 0.05 | -0.54 | -0.50 | -1.82 | 0.04 | Covalent bond |
| 51 | 33(H ) -- 10(C ) | 0.28 | 0.04 | -0.32 | -0.28 | -0.98 | 0.02 | Covalent bond |
| 52 | 4(N ) -- 3(C ) | 0.34 | 0.25 | -0.73 | -0.49 | -0.96 | 0.16 | Covalent bond |
| 53 | 9(C ) -- 10(C ) | 0.31 | 0.10 | -0.42 | -0.32 | -0.86 | 0.20 | Covalent bond |
| 54 | 3(C ) -- 2(N ) | 0.30 | 0.17 | -0.56 | -0.39 | -0.88 | 0.08 | Covalent bond |
| 55 | 31(H ) -- 5(C ) | 0.28 | 0.03 | -0.31 | -0.28 | -0.97 | 0.03 | Covalent bond |
| 56 | 9(C ) -- 6(C ) | 0.31 | 0.10 | -0.43 | -0.32 | -0.88 | 0.24 | Covalent bond |
| 57 | 4(N ) -- 5(C ) | 0.27 | 0.15 | -0.48 | -0.33 | -0.72 | 0.02 | Covalent bond |
| **59** | **8(S ) -- 44(H )** | **0.00** | **0.00** | **0.00** | **0.00** | **0.01** | **0.11** | **Van der Waal** |
| 61 | 2(N ) -- 6(C ) | 0.27 | 0.19 | -0.55 | -0.36 | -0.66 | 0.03 | Covalent bond |
| 62 | 2(N ) -- 1(C ) | 0.31 | 0.19 | -0.60 | -0.41 | -0.86 | 0.09 | Covalent bond |
| 63 | 5(C ) -- 1(C ) | 0.26 | 0.06 | -0.27 | -0.21 | -0.62 | 0.08 | Covalent bond |
| **64** | **4(N ) -- 26(O )** | **0.01** | **0.01** | **-0.01** | **0.00** | **0.04** | **0.69** | **Van der Waal** |
| 65 | 10(C ) -- 11(C ) | 0.31 | 0.10 | -0.42 | -0.32 | -0.86 | 0.19 | Covalent bond |
| 66 | 1(C ) -- 7(O ) | 0.41 | 0.68 | -1.38 | -0.70 | -0.10 | 0.07 | Covalent bond |
| 68 | 23(N ) -- 22(C ) | 0.48 | 0.82 | -1.69 | -0.86 | -0.17 | 0.00 | Covalent bond |
| 69 | 42(H ) -- 27(C ) | 0.28 | 0.04 | -0.32 | -0.28 | -0.99 | 0.04 | Covalent bond |
| 71 | 5(C ) -- 14(C ) | 0.23 | 0.06 | -0.24 | -0.18 | -0.50 | 0.02 | Covalent bond |
| 72 | 38(H ) -- 16(C ) | 0.28 | 0.03 | -0.31 | -0.27 | -0.97 | 0.01 | Covalent bond |
| 73 | 6(C ) -- 13(C ) | 0.31 | 0.10 | -0.43 | -0.32 | -0.88 | 0.24 | Covalent bond |
| 74 | 44(H ) -- 27(C ) | 0.28 | 0.04 | -0.31 | -0.28 | -0.96 | 0.04 | Covalent bond |
| 75 | 11(C ) -- 34(H ) | 0.28 | 0.04 | -0.32 | -0.28 | -0.98 | 0.01 | Covalent bond |
| **76** | **38(H ) -- 26(O )** | **0.02** | **0.01** | **-0.01** | **0.00** | **0.07** | **0.25** | **H-bond** |
| 77 | 22(C ) -- 16(C ) | 0.26 | 0.08 | -0.32 | -0.25 | -0.68 | 0.04 | Covalent bond |
| 78 | 27(C ) -- 26(O ) | 0.24 | 0.27 | -0.59 | -0.32 | -0.20 | 0.00 | Covalent bond |
| 79 | 11(C ) -- 12(C ) | 0.31 | 0.10 | -0.42 | -0.32 | -0.86 | 0.19 | Covalent bond |
| 81 | 16(C ) -- 14(C ) | 0.23 | 0.05 | -0.23 | -0.17 | -0.47 | 0.04 | Covalent bond |
| 82 | 13(C ) -- 12(C ) | 0.31 | 0.10 | -0.42 | -0.32 | -0.87 | 0.20 | Covalent bond |
| 83 | 13(C ) -- 36(H ) | 0.28 | 0.03 | -0.32 | -0.28 | -0.99 | 0.01 | Covalent bond |
| 84 | 27(C ) -- 43(H ) | 0.28 | 0.04 | -0.31 | -0.28 | -0.96 | 0.04 | Covalent bond |
| 85 | 14(C ) -- 37(H ) | 0.28 | 0.04 | -0.31 | -0.28 | -0.97 | 0.01 | Covalent bond |
| 87 | 16(C ) -- 24(C ) | 0.26 | 0.07 | -0.32 | -0.25 | -0.69 | 0.04 | Covalent bond |
| 88 | 26(O ) -- 17(C ) | 0.28 | 0.33 | -0.74 | -0.41 | -0.29 | 0.03 | Covalent bond |
| 89 | 14(C ) -- 15(C ) | 0.25 | 0.06 | -0.26 | -0.20 | -0.58 | 0.04 | Covalent bond |
| 90 | 12(C ) -- 35(H ) | 0.28 | 0.04 | -0.32 | -0.28 | -0.98 | 0.02 | Covalent bond |
| 91 | 17(C ) -- 15(C ) | 0.31 | 0.10 | -0.41 | -0.31 | -0.85 | 0.27 | Covalent bond |
| 92 | 17(C ) -- 18(C ) | 0.31 | 0.10 | -0.42 | -0.31 | -0.85 | 0.24 | Covalent bond |
| **93** | **36(H ) -- 20(C )** | **0.01** | **0.00** | **0.00** | **0.00** | **0.02** | **0.58** | **Van der Waal** |
| 94 | 15(C ) -- 21(C ) | 0.30 | 0.10 | -0.41 | -0.31 | -0.82 | 0.23 | Covalent bond |
| **95** | **13(C ) -- 47(H )** | **0.00** | **0.00** | **0.00** | **0.00** | **0.01** | **0.35** | **Van der Waal** |
| 96 | 39(H ) -- 18(C ) | 0.28 | 0.04 | -0.32 | -0.28 | -0.98 | 0.02 | Covalent bond |
| 97 | 24(C ) -- 25(N ) | 0.48 | 0.82 | -1.69 | -0.86 | -0.17 | 0.00 | Covalent bond |
| 100 | 21(C ) -- 41(H ) | 0.28 | 0.04 | -0.32 | -0.28 | -0.98 | 0.02 | Covalent bond |
| 101 | 18(C ) -- 19(C ) | 0.31 | 0.11 | -0.42 | -0.32 | -0.86 | 0.22 | Covalent bond |
| 102 | 21(C ) -- 20(C ) | 0.31 | 0.10 | -0.42 | -0.32 | -0.86 | 0.24 | Covalent bond |
| 103 | 19(C ) -- 20(C ) | 0.31 | 0.10 | -0.42 | -0.32 | -0.88 | 0.24 | Covalent bond |
| 104 | 19(C ) -- 40(H ) | 0.28 | 0.04 | -0.31 | -0.28 | -0.97 | 0.02 | Covalent bond |
| 105 | 47(H ) -- 29(C ) | 0.28 | 0.04 | -0.31 | -0.27 | -0.95 | 0.04 | Covalent bond |
| 106 | 20(C ) -- 28(O ) | 0.29 | 0.33 | -0.75 | -0.41 | -0.32 | 0.02 | Covalent bond |
| 107 | 28(O ) -- 29(C ) | 0.25 | 0.27 | -0.59 | -0.33 | -0.25 | 0.00 | Covalent bond |
| 108 | 29(C ) -- 46(H ) | 0.28 | 0.04 | -0.31 | -0.27 | -0.95 | 0.04 | Covalent bond |
| 109 | 29(C ) -- 45(H ) | 0.28 | 0.04 | -0.32 | -0.28 | -0.99 | 0.04 | Covalent bond |
